# Stabilization Effects in Phosphinyl Radicals: The Scope of the Donor, Acceptor, and Captodative Functionalization

**DOI:** 10.1021/acs.inorgchem.5c03515

**Published:** 2025-11-17

**Authors:** Pelin Kaymak, Zoltán Benkő

**Affiliations:** † Department of Inorganic and Analytical Chemistry, Faculty of Chemical Technology and Biotechnology, 61810 Budapest University of Technology and Economics, H-1111 Budapest, Hungary; ‡ HUN-REN-BME Computation Driven Chemistry Research Group, H-1111 Budapest, Hungary

## Abstract

Accessing stable phosphinyl radicals continues to pose a major synthetic challenge. To aid the effective design of such radicals and to understand their behaviors, this computational study systematically explores the possibilities and limitations of electronic and steric effects governing the stability of phosphinyl radicals, with a particular focus on captodative substitutions. To decipher the delocalization effects, the electronic structures of various radicals have been scrutinized by their spin distributions and using natural bonding orbital analyses. The best stabilization effects are primarily offered by π-donor groups; however, the most efficient π-donors are exceptions and suffer from a saturation effect. To compensate for this disadvantageous phenomenon, various captodative substitutions have been assessed, and several examples were found to benefit from extra stabilization. As electronic effects alone are insufficient to prevent the dimerization, additional protection arising from sterically encumbered substituents is also necessary, and steric effects were found to prevail over electronic effects in stabilizing the monomeric radical. Among the studied substituents, spherical groups placed at the donor and acceptor sites provide better steric protection than planar groups. Embedding the N-centers into cyclic frameworks further enhances stability. Integrating the results of our systematic investigations, we propose several potential candidates for synthetic purposes.

## Graphical abstract

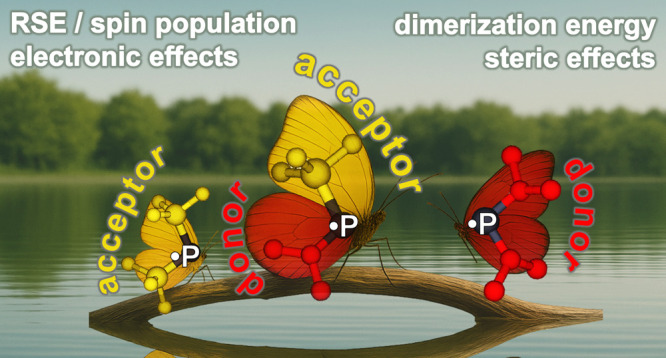

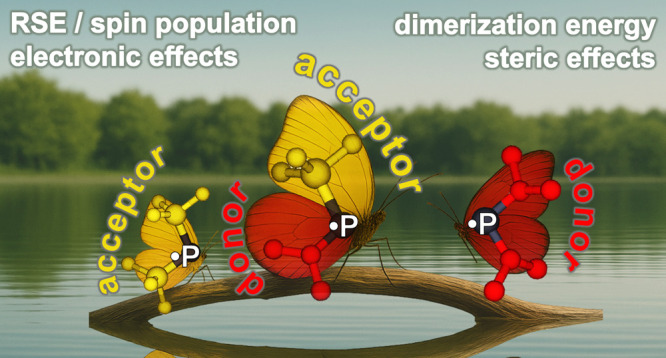

## 1 Introduction

Owing to their particular properties and versatile reactivities, radicals containing main-group elements are of great importance in various fields of experimental and theoretical chemistry.^1–3^ Among these, P-containing radicals are valuable tools serving as intermediates in numerous synthetic pathways, may have a crucial role in constructing complex organophosphorus compounds, and facilitate the development of new methodologies in synthesis by enabling bond formation processes.^4,5^ In addition to these applications, phosphonyl (R_2_P^•^=O) radicals are highly active components in photoinitiated polymerization reactions,^6–10^ allowing important applications such as surface modifications and coatings,^11,12^ 3D printing technologies,^13,14^ and the manufacturing of optical devices.^2,15–17^ Despite the difficulties in their experimental accessibility stemming from their high reactivities,^5^ the investigations on phosphorus-centered radicals continue to inspire research and development across multiple scientific disciplines.

In the past decades, notable advancements have been achieved in isolating and characterizing various stable phosphorus-centered radicals, encompassing neutral, cationic, and anionic forms.^1,18–20^ The main classes of neutral phosphorus-centered radicals are phosphinyl (R_2_P^•^), phosphonyl (R_2_P^•^=O), and phosphoranyl (R_4_P^•^); the subject of the present work is restricted to the phosphinyl type. Lacking the beneficial stabilization of the charge, however, the task of accessing stable neutral phosphinyl radicals (R_2_P^•^) remains challenging as a consequence of their intrinsic propensity to dimerize, especially in the solid state. Therefore, the majority of such radicals have commonly been obtained by a “trial and error” approach. After the discovery of the first persistent dialkylphosphinyl radical (Figure 1a) by Lappert et al.^21^ several stable or persistent phosphinyl radicals have been delineated.^22–33^ For example, sterically congested diphosphines (R_2_PPR_2_, Figure 1b) were found to undergo reversible dissociation delivering persistent phosphinyl radicals in both gas-phase and solution environments, sometimes initiated photochemically.^34–36^ Extending the scope of organic backbones, a unique phosphinyl radical embedded into a five-membered ring exhibiting four trimethylsilyl (TMS) groups in the α positions to protect the P-center (Figure 1c) was proven to be stable as a monomer even in the solid state.^24^ As a further important milestone, iminato (nitridovanadium trisanilide, −N=VR′_3_) substituents were utilized to stabilize a phosphinyl radical that is monomeric in the solid state, allowing for its successful isolation and crystallographic characterization (Figure 1d).^37^ Later on, to tune the stability of such bisiminato radicals, substituting either or both of the VR′_3_ moieties with bulky imidazol-2-ylidene groups afforded further stable radicals (Figure 1e and 1f).^38^ To complement the so-called “carbene-supported″ NPN radical (Figure 1f), its analogue exhibiting a PPP core was also accessed successfully in the past decade (Figure 1g).^39^ Recently, the isolation and characterization of a stable heteroleptic diarylphosphinyl radical have also been presented.^40^

**Figure 1:**
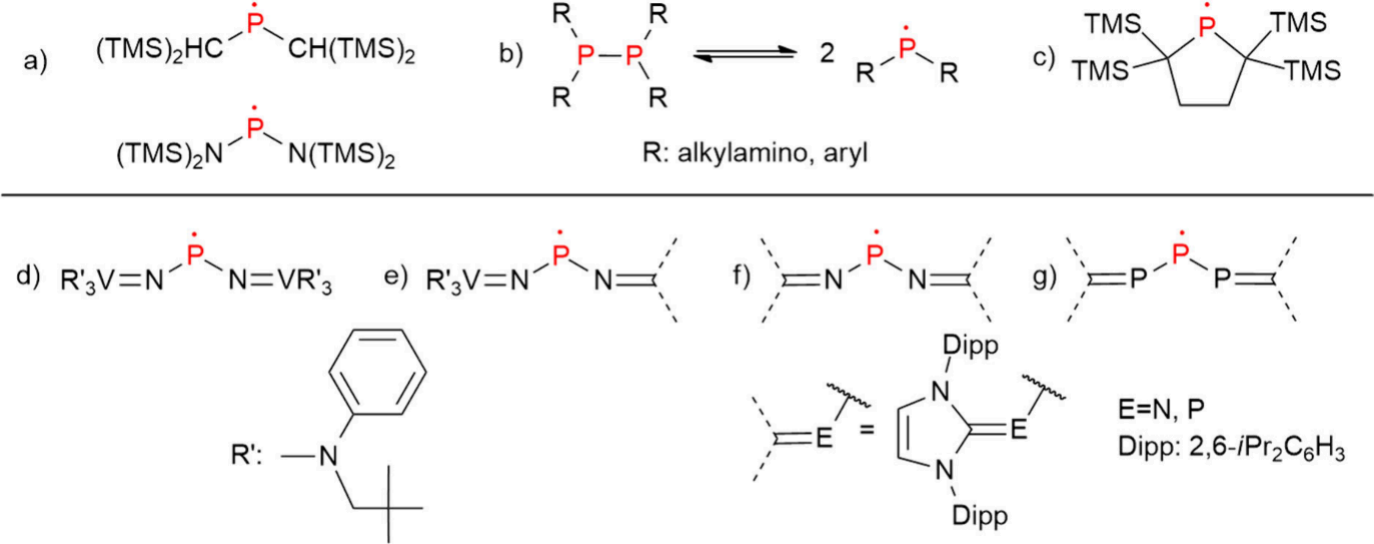
(a,c–g) Selected examples of persistent and stable phosphinyl radicals; (b) equilibrium between diphosphine and the corresponding phosphinyl radicals.

Recently, the reduction of carbene-stabilized phosphenium cations has been shown to deliver stable radicals, which are better described as C-center radicals exhibiting flanking phosphinyl substituents (so-called α-radical phosphines) instead of phosphinyl radicals stabilized by carbenes.^20,41,42^

Methods for enhancing the stability of a phosphinyl radical often integrate both electronic and steric aspects.^3,43^ Electronic stabilization relies on introducing adjacent functional groups that facilitate the delocalization of the lone electron over neighboring centers. In contrast, steric stabilization can be achieved by creating a congested environment around the radical center using bulky substituents, generating steric repulsion that prevents the formation of the intrinsically strong covalent P-P bond. It is often challenging to differentiate between electronic and steric effects, and experimental research commonly aims at using both types due to their pronounced influence on stability.^25^

A combined experimental and theoretical study on the stability aspects of [CH­(TMS)_2_]_2_P^•^ radical (as well as its arsenic analogue) established the ’Jack-in-the-box’ model,^35^ highlighting the importance of steric effects and conformational changes. While these radicals dimerize in the solid state, they stay monomeric in the solution environment or in the gas phase, and the dissociation of the diphosphine into the two radicals is accompanied by substantial conformational changes of the ligand around the P-center. The asymmetric and flexible substituents act as molecular springs, and the steric strain energy stored in the dimer is released upon dissolution or evaporation, contributing significantly to the thermodynamic stability of the monomeric radical.

Complementing the experimental investigations, a handful of purely computational studies have also emerged to gain insights into the stabilization effects of P-centered radicals. Krenske and Coote have reported that hydroxyphosphinyl radicals of X­(HO)­P^•^ type are thermodynamically more stable (up to 54.3 kJ/mol or 13.0 kcal/mol) than the corresponding phosphonyl isomers XHP­(O)^•^.^44^ This stability order is unusual in phosphorus chemistry, and the higher stabilities of the hydroxyphosphinyl species are primarily attributable to the greater p character in the singly occupied molecular orbital (SOMO) at their P-atoms compared to the analogous phosphonyl tautomers. Additionally, the electronegativity of the substituent X has been suggested to play a role in determining the degree of stabilization, which is further tuned by the capability of the substituent for π-interactions with the radical center. Indeed, π-acceptor groups such as CN or Ph enhance spin delocalization. As to the π-donor substituents, replacing one of the H atoms in H_2_P^•^ by a hydroxyl group [giving H­(HO)­P^•^] affords a moderate stabilization of 19.6 kJ/mol (4.7 kcal/mol). However, the replacement of the second H by a different substituent X, leading to X­(HO)­P^•^, only has a minor effect on the stability. In general, when the P-center interacts with a π-donor substituent, the energy of the π-orbital decreases, while the SOMO gets destabilized, diminishing the ability of the unpaired electron to engage with another π-donor substituent. This competition leads to a saturation effect, and therefore, further significant stabilization in the X­(HO)­P^•^ type of radicals is only expected if X is a better π-donor than OH.

To understand the effect of amino substituents, the homolytic dissociation of sterically strained tetraamino-diphosphines [(R_2_N)_2_P-P­(NR_2_)_2_] leading to persistent radicals [(R_2_N)_2_P^•^] has been analyzed in a combined experimental-computational study.^45^ To compare the delocalization effects, the rotation barriers of the NH_2_ substituent in the parent diaminophosphinyl radical (H_2_N)_2_P^•^ and its cationic counterpart (H_2_N)_2_P^+^ have been explored. The rotation barrier is larger (ΔE^⧧^ = 17.8 kJ/mol or 4.3 kcal/mol) for the cation compared to the corresponding radical (ΔE^⧧^ = 2.0 kJ/mol or 0.5 kcal/mol), in line with the differing interactions: In comparison with the LUMO of the cation, the additional electron in the antibonding π*-type SOMO of the radical reduces the bond order and parallelly the stabilization provided by π-delocalization. As a consequence, the interaction between the lone electron at the P-atom and the lone pair at N in the (R_2_N)_2_P^•^ type of radicals is best described as hyperconjugation rather than strong π-delocalization.

Complementing these theoretical studies, the effect of π-delocalization on the stability of phosphinyl radicals exhibiting all-carbon backbones has also been scrutinized recently in our group, underlining the impact of the ring size on the delocalization.^46^

The preceding examples underscore that the primary stabilization mechanisms of acyclic phosphinyl radicals have only been investigated for rather specific substituents without targeting a more general understanding. Consequently, we embarked on a systematic investigation to elucidate the magnitudes of electronic effects that may contribute to stability and find their limitations in comparison with steric effects. More generally, our study also aims to decipher the specific factors governing the stability of phosphinyl radicals exhibiting versatile substitution patterns, in order to propose realistic targets for eventual synthetic realization.

## 2 Results and Discussion

The paper is organized as follows: First, the electronic effects will be scrutinized on a relative scale employing radicals with a wide range of model functional groups attached to the P-center. By combining selected donor and acceptor substituents, the captodative effects on the stabilities of phosphinyl radicals will then be analyzed. Finally, the global stability of selected radicals decorated by sterically demanding substituents will be screened using dimerization energies.

### Electronic Effects

To quantify the electronic effects, a hypothetical isodesmic reaction equation tailored for phosphinyl radicals (Scheme 1) was employed to compute radical stabilization energies (RSEs). These RSE values gauge the stability of the radical RR′P^•^ in question relative to the reference radical H_2_P^•^: a lower (more negative) RSE value is consistent with a greater stability on this relative scale. Detailed explanations of the RSE concept can be found in the literature.^47,48^ RSEs were first determined at the (U)­ωB97X-D/6-311G** level of theory that was previously used successfully to assess the stability of other P-centered radicals.^46,49^ To test the reliability of this level, two further DFT functionals (M06-2X and B3LYP-D3) were also employed. In addition, the effect of a larger basis set (aug-cc-pVQZ) was tested. To obtain precise RSE values, single-point energy calculations were conducted at the (U)­DF-CCSD­(T)/aug-cc-pVTZ//(U)­ωB97X-D/6-311G** level (Table 1). As all of these levels of theory show highly similar results (see Supporting Information, SI), in the following, the “gold standard” coupled cluster results will only be discussed in detail (for DFT results, see Tables S1 and S2 in the Supporting Information). It is well-known in the literature^47^ that the energies of isodesmic reactions are generally insensitive to the level of theory (the errors on the two sides of the equation cancel out each other), aligning with our observations. To assess the delocalization effects, spin populations were obtained using the Mulliken partitioning scheme at the ωB97X-D/6-311G** level.

**Scheme 1:**
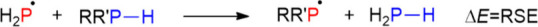
Isodesmic Reaction Equation for Determining RSE

**Table 1:** RSE Values Obtained at the (U)­DF-CCSD­(T)/aug-cc-pVTZ//(U)­ωB97X-D/6-311G** Level in kcal/mol, and ρ_spin_(P) Spin Populations at P for RHP^•^ and R_2_P^•^ Type Radicals with R = EH_*n*_ (*n* = 0–3) and R= EMe_*n*_ (*n* = 1–3)

	R = EH_*n*_				R = EMe_*n*_			
	RHP^•^		R_2_P^•^		RHP^•^		R_2_P^•^	
E	RSE	ρ_spin_(P)	RSE	ρ_spin_(P)	RSE	ρ_spin_(P)	RSE	ρ_spin_(P)
F	–3.8	0.938	–7.1	0.901				
Cl	–4.9	0.937	–9.6	0.891				
Br	–4.9	0.924	–9.6	0.875				
O	–5.8	0.913	–5.4	0.862	–6.9	0.902	–6.6	0.861
S	–7.9	0.900	–7.8	0.841	–6.2	0.871	–8.6	0.816
Se	–7.8	0.878	–8.5	0.820	–6.2	0.848	–9.1	0.799
N	–6.5	0.817	–5.2	0.843	–8.1	0.786	–5.3	0.810
P	–3.8	0.940	–6.8	0.905	–3.9	0.922	–6.0	0.866
As	–2.7	0.963	–5.6	0.941	–2.6	0.974	–4.9	0.950
C	–1.2	1.000	–2.2	1.000	–0.6	1.030	–0.7	1.002
Si	–0.7	0.990	–1.5	0.983	–0.5	0.997	–1.2	0.939
Ge	–0.4	0.998	–1.0	0.997	–0.6	1.018	–1.3	0.979
B	2.5	0.903	4.9	0.850	1.8	0.962	2.9	0.991
Al	0.5	0.963	–0.4	0.978	0.4	0.997	–0.2	0.969
Ga	1.2	0.978	0.7	0.996	0.5	1.007	0.0	0.979

In the present study, we categorize the phosphinyl radicals into three classes based on their substituents: (i) the RHP^•^ type, distinguished by the presence of a hydrogen substituent alongside an R substituent; (ii) the R_2_P^•^ type, with two identical substituents, and (iii) the R^A^R^D^P^•^ type, exhibiting substitution by two electronically different groups (R^A^: Acceptor, R^D^: Donor), which may enable push–pull (captodative) stabilization effects. To attain versatility, we systematically examined a diverse range of simple functional groups of both EH_*n*_ and EMe_*n*_ type (with H or methyl substituents at the E centers, respectively) exhibiting central atoms E spanning the first three rows of the main groups from 13 to 17 (see Table 1). Some of the substituents (such as involving B, Al, and Ga atoms) are rather of theoretical importance as their inclusion seems hard to achieve synthetically. Nevertheless, as these groups are strongly electron-withdrawing, their incorporation in our study expands the scope of the substituents. Apart from these few unusual examples, the majority of substituents (e.g., OR, SR, SeR, NR_2_, PR_2_, etc.) are synthetically accessible in a straightforward manner. Since unsaturated functional groups were subjects of a previous study, we exclude them here as possibilities.^46^

The parent EH_*n*_ substituents have been employed in our study as simple models because the H-atom is expected to cause negligible electronic influence on the E center. On the other hand, the methyl substitution in EMe_*n*_ serves as a general model for saturated organic frameworks. We begin our discussion with the RHP^•^ and R_2_P^•^ type radicals. In our hypothesis, the comparison of the stabilization in these couples may enable a thorough understanding of possible additive, saturation, and synergistic effects.

### RHP^•^ Type Radicals

First, we discuss the electronic effects in the asymmetric RHP^•^ type model radicals, focusing on the parent groups (R = EH_*n*_ type), and the substituents will be presented in a decreasing order of stabilization (increasing RSE). Then, we only highlight the differences for the methylated analogues.

The **RSE values** (Table 1) encompass a relatively wide range between −7.9 and + 2.5 kcal/mol. The lowest RSEs are obtained for the π-donor thioxyl (SH) and selenoxyl (SeH) substituents, having similar RSE values of −7.9 and −7.8 kcal/mol, respectively. Compared to its heavier congeners in Group 16, however, the hydroxyl group affords a somewhat lower stabilization (less negative RSE of −5.8 kcal/mol). While R = NH_2_ (also a good π-donor) offers slightly better stabilization (RSE= −6.5 kcal/mol) than the hydroxyl group, the heavier PH_2_ and AsH_2_ substituents have rather limited effects (RSE = −3.8 and −2.7 kcal/mol), in line with the high pyramidality at these pnictogen centers (sum of bond angles: 293.5° and 285.0°, respectively in PH_2_ and AsH_2_; versus 357.6° in NH_2_). The halogen substituents also act stabilizing, but their effects are less pronounced. Among them, the “heavier” Cl and Br (RSE = −4.9 kcal/mol) provide slightly better stabilization than F (RSE = −3.8 kcal/mol). The RSEs of P-radicals with substituents containing Group 14 elements indicate negligible stabilization effects (RSE = −1.2, −0.7, and −0.4 for R = CH_3_, SiH_3_, and GeH_3_, respectively). In contrast, the positive RSE values caused by the substituents with Group 13 elements (BH_2_, AlH_2_, GaH_2_) outline destabilization, consistent with the marked π-acceptor (Lewis acidic) properties of these groups.

The **spin populations** at the P-center [ρ_spin_(P)] range between 0.817 and 1.000. The lowest ρ_spin_ with a value of 0.817 is provided by the NH_2_ group, while those of Group 16 substituents are higher but decline when descending the group (OH, SH, and SeH with ρ_spin_(P) = 0.913, 0.900, and 0.878, respectively) in line with the increasing stabilization effect. A similar trend is found for halogens, although the values are slightly even higher (ρ_spin_(P) = 0.938, 0.937, and 0.924, respectively, for R = F, Cl, and Br), parallel with the less effective stabilization outlined by higher RSEs. Considering all of the studied substituents, the highest spin populations (around unity) were found for those with tetrel (Group 14) elements in accord with their negligible RSE values. Notably, the spin population at the P-center obtained with the boryl group is almost the smallest [ρ_spin_(P) = 0.903]. This observation indicates that π-acceptor substituents can effectively abstract spin density from the 3p orbital of the P-center; however, this does not result in energetic stabilization (an explanation for this unusual inconsistency will be given below). A loose trend can be found between the RSE values and the spin populations (see Figure S1) and, in general, the lower ρ_spin_ is consistent with a lower RSE (higher stabilization). However, the correlation is poor (R^2^ = 0.402) because several groups, including the above-mentioned amino and boryl substituents, are outliers. We also attempted to search for possible correlation with other descriptors, such as the electronegativity^50^ or the ionization potential^51^ of the central atom E, but we could not find any meaningful relationship (see Figures S2 and S3), contrasting the outcome of a previous study.^44^

Compared to the parent substituents, the RSE values obtained for the methylated R = EMe_*n*_ analogues in the RHP^•^ category span similarly wide between −8.1 and 1.8 kcal/mol (see Table 1). In general, switching EH_*n*_ to EMe_*n*_ substituents has a minor effect on the tendency (see Figure S4). As an exception, the NMe_2_ group leads to some extra stabilization with RSE of −8.1 kcal/mol, which is lower compared to the R = NH_2_ analogue (RSE = −6.5 kcal/mol), indicating a better hyperconjugation triggered by the methyl groups.

### R_2_P^•^ type radicals

In the following, we will briefly discuss the effect of a second R substituent of the same kind on the stability of the phosphinyl radical with the formula R_2_P^•^, and focus only on the differences, because the trends align with those of the RHP^•^ type for the majority of the substituents. In contrast to single substitutions with the chalcogen-containing groups affording the lowest RSEs, for the R_2_P^•^ type, the halogens (especially chlorine and bromine) act the most stabilizing with the lowest RSE reaching −9.6 kcal/mol (for both Cl_2_P^•^ and Br_2_P^•^). However, the monovalent nature of halogens, unfortunately, prohibits further functionalization and thus excludes synthetic relevance. Among substituents with Group 15 elements, two PH_2_ groups provide the lowest RSE (−6.8 kcal/mol); even less compared to the NH_2_ substituents (−5.2 kcal/mol). The unusually low stabilizing effect observed for two NH_2_ groups results from a competition, which will be discussed below. Considering the elements in a given row of the periodic table, the RSE values show a decreasing tendency from left to right, indicating a gradual enhancement in stability. For the substituents containing Group 15–17 elements, the RSE increases with the electronegativity as well as ionization energy of the element E; however, for Groups 13 and 14, no relationship can be observed (see Figures S5 and S6).

Replacing the hydrogens by methyl groups in the disubstituted radicals, giving (Me_*n*_E)_2_P^•^, the range of RSE lies between −9.1 and 2.9 kcal/mol. The greatest stabilization is observed for the SeMe substituent (RSE = −9.1 kcal/mol); and the corresponding RSE is similar to those found for the Cl_2_P^•^ or Br_2_P^•^ radicals exhibiting the lowest values in the R_2_P^•^ family. In general, the RSEs follow similar trends as the parent analogues (with R = EH_*n*_).

### Comparing RHP^•^ and R_2_P^•^

To analyze the relation between the mono- and disubstitution, we plotted the RSEs of the radicals with two identical substituents (R_2_P^•^) as a function of the RSE obtained with only one R functional group (RHP^•^) attached to the P-center (Figure 2). A point on the blue RSE­(R_2_P^•^) = 2RSE­(RHP^•^) line corresponds to pure additivity. In contrast, the red RSE­(R_2_P^•^)=RSE­(RHP^•^) line indicates that the stability just remains the same when the second R substituent is introduced, consistent with a saturation effect.

**Figure 2:**
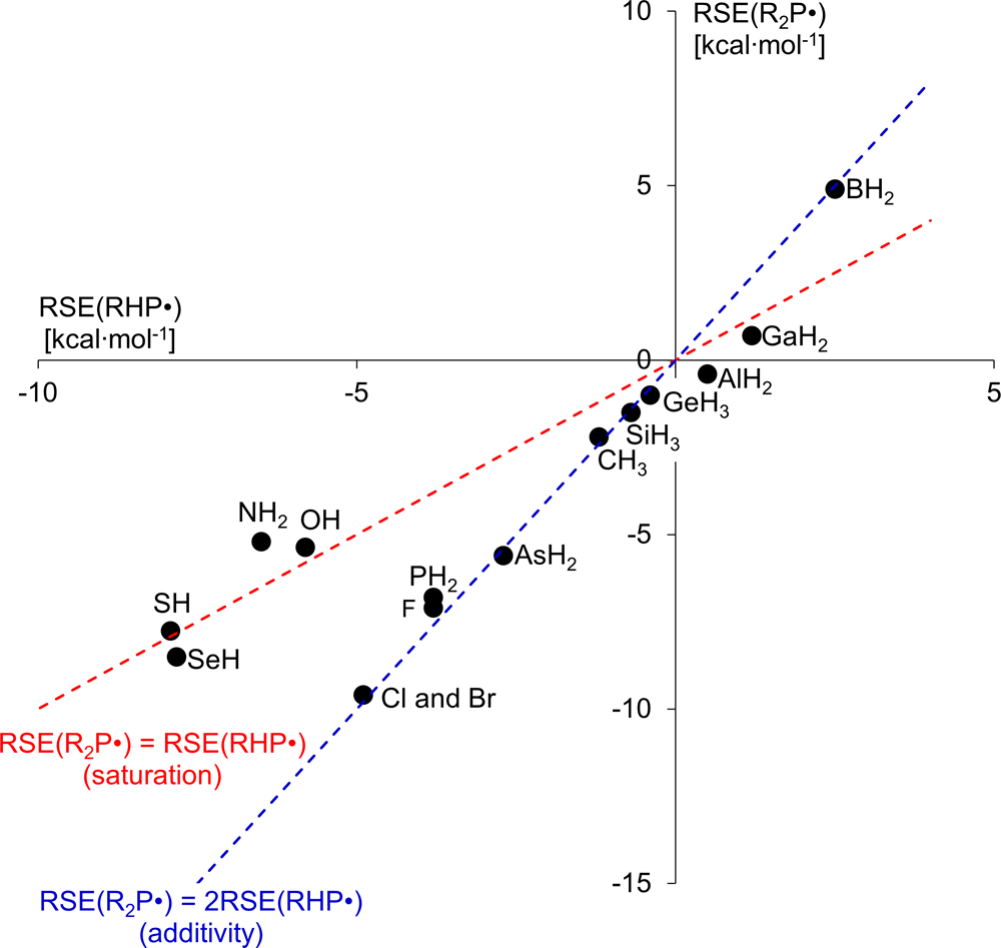
Relation between RSEs of monosubstituted RHP^•^ and disubstituted R_2_P^•^ type radicals for R = EH_*n*_.

Based on the diagram, no meaningful synergistic effect that is |RSE­(R_2_P^•^)| > 2|RSE­(RHP^•^)| can be found. Actually, the majority of the data points follow the RSE­(R_2_P^•^) = 2RSE­(RHP^•^) “additivity’ line, meaning that the identical substitution at both sides of the P-center leads to an additive effect, e.g., for halogens or Group 14/15 elements (except nitrogen). In the “destabilizing” direction, two boryl substituents also exert a twice larger RSE. In contrast to these additive effects, the chalcoxyl (OH, SH, and SeH) and amino (NH_2_) groups follow the “saturation” line RSE­(R_2_P^•^) = RSE­(RHP^•^), implying that the RSE of the radical remains practically unaffected by a second, identical R substituent. The data point for the amino (NH_2_) group even slightly surpasses the red line, indicating that a single substitution at the P-center provides more stabilization than the symmetrical disubstitution, in line with previous observations.^44^

To gain deeper insights into these effects, we have quantified the donor–acceptor interactions for all studied substituents in both the RHP^•^ and R_2_P^•^ types of radicals using the interaction energies ΔE^(2)^ originating from second-order perturbation theory based on the Fock matrix in NBO basis (for the complete data set see Table S3). In each radical, one meaningful donor–acceptor interaction (that is, ΔE^(2)^ > 3 kcal/mol) was only detected, and two representative examples visualizing these interactions together with the corresponding Kohn–Sham SOMOs are shown in Figure 3. However, the analysis of the results reveals remarkable differences between the π-donor and π-acceptor R substituents: In the case of π-donor groups such as halogens, amino, and chalcoxyl substituents, the primary stabilizing interactions arise in the β-series of electrons as donations from the lone electron pair at the donor atoms (halogen, N, O, S, or Se; see Figure 3a for LP@N) into the empty orbital of practically pure 3p character at phosphorus (in the following, we denote this orbital as LP*@P). The arising stabilization energy offered by this donation is denoted as ΔE^(2)^_D_. In marked contrast, π-withdrawing groups (e.g., boryl or silyl) affect the distribution of the α-electrons: The empty p-orbital (see Figure 3b for LP*@B) or σ*-antibonding orbital (e.g., for silyl groups) at the central atom in these substituents effectively abstracts the α-lone electron occupying the 3p orbital at P (in the following, LP@P); ΔE^(2)^_BD_ gives the stabilization energy stemming from this back-donation.

**Figure 3:**
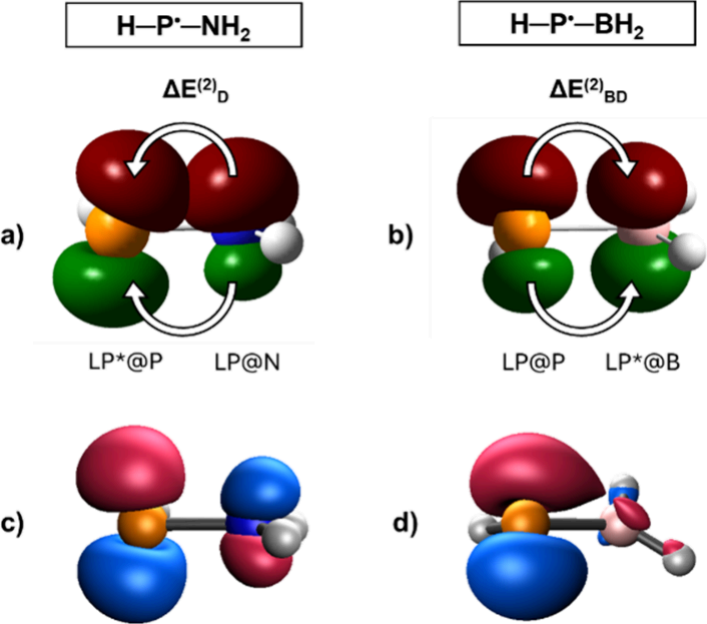
(a) Donation from the lone pair at N (LP@N) into the empty 3p orbital at P (LP*@P) in H–P^•^–NH_2_ giving ΔE^(2)^_D_; (b) Back-donation from the lone pair at P (LP@P) into the vacant 2p orbital at B (LP*@B) in H–P^•^–BH_2_ giving ΔE^(2)^_BD_; (c) SOMO of H–P^•^–NH_2_; (d) SOMO of H–P^•^–BH_2_ (contour values of 0.2)

In order to better understand the addition and saturation effects, we will assess and compare the ΔE^(2)^ interaction energies of both types of radicals. While in the RHP^•^ type radicals one major donation was only found (arising between the R group and the P-center), in the case of R_2_P^•^ radicals, we obtained the total interaction energies by summing the two (almost identical) increments corresponding to the symmetrical interactions toward the R substituents (see Table S3). For clarity, these interaction energies will be referred to as ΔE^(2)^(RHP^•^) and ΔE^(2)^(R_2_P^•^), respectively.

The π-donor substituents can be divided further into two larger categories depending on the additivity of ΔE^(2)^ interaction energies. For halogens (considering the halogen lone pair → LP*@P donation) the total interaction energies found in the R_2_P^•^ type are nearly double of the ΔE^(2)^ energies obtained for RHP^•^ [ΔE^(2)^_D_(RHP^•^) = 25.6, 24.4, and 25.3 kcal/mol, ΔE^(2)^_D_(R_2_P^•^) = 43.8, 45.0, and 46.8 kcal/mol for F, Cl, and Br, respectively]. These values are practically independent of the type of halogen, in line with their similar RSE values (Table 1). Moreover, the additivity of the interaction energies, namely ΔE^(2)^_D_(R_2_P^•^) ≈ 2• ΔE^(2)^_D_(RHP^•^), is nicely consistent with the additivity of RSE shown in Figure 2.

Unlike halogens, for other π-donors, particularly for the OH and NH_2_ substituents, the total interaction energies calculated for the disubstituted radicals [ΔE^(2)^_D_(R_2_P^•^) = 25.8 and 49.8 kcal/mol for (HO)_2_P^•^ and (H_2_N)_2_P^•^, respectively] are even lower than those found in the corresponding monosubstituted species [ΔE^(2)^_D_(RHP^•^) = 40.9 and 62.1 kcal/mol for (HO)­HP^•^ and (H_2_N)­HP^•^, respectively]. Similar statements can be made for the SH and SeH groups. These results clearly show that the origin of the saturation effect lies in the competing donations of two different lone pairs toward the same empty 3p-orbital at P (LP*@P). Furthermore, these findings align with the trends of RSEs, reinforcing the concept that the second substituent remains ineffective in stabilizing the radicals.

In the case of π-withdrawing substituents, particularly BH_2_ but also AlH_2_, GaH_2_, SiH_3_, and GeH_3_ to lesser extent, the donation from the half-filled 3p orbital at P (LP@P) into the empty 2p-orbital at B affords a significant interaction energy of ΔE^(2)^_BD_ = 35.6 kcal/mol for (H_2_B)­HP^•^ and somewhat less than double of this value, 47.0 kcal/mol for (H_2_B)_2_P^•^, signifying a limited additivity. These remarkable donor–acceptor interactions would hint at significant stabilization in these radicals; however, the positive RSE values indicate clear destabilization. As the isodesmic reaction compares the stabilities of the radicals R_2_P^•^ and H_2_P^•^ relative to the corresponding phosphines R_2_PH and PH_3_, we inspected the stabilizing interactions in the phosphine counterparts. Taking the analogous boryl-substituted phosphines as references, the NBO analyses reveal donations from the lone electron pair at P into the empty 2p orbital at B accompanied by significant ΔE^(2)^_BD_ energies [ΔE^(2)^_BD_ = 35.6 and 173.0 kcal/mol for (H_2_B)­HP^•^ and (H_2_B)_2_PH, respectively], as expected considering the remarkable Lewis acidity of the boron centers. The comparison of these ΔE^(2)^_BD_ interaction energies explains that at least the same amount of stabilization is lost in the phosphine than that gained in the radical counterpart when the RSE is obtained, and this deficiency in stabilization is reflected in the positive RSE.

### Captodative Substitutions

It is clear from the above results that the most promising π-donor substituents have severe limitations in double substitution. Therefore, we turned our attention toward the possibility of enhancing the stability by using an additional π-acceptor group. The captodative or push–pull effect operates through the synergistic communication of donor and acceptor groups within a molecule.^52–54^ This section probes such effects on the stability of the R^A^R^D^P^•^ type phosphinyl radicals. One of the substituents (R^A^) is chosen as π-electron-accepting (in our consideration, BR′_2_, AlR′_2_, GaR′_2_, SiR′_3,_ and GeR′_3_), while the other (R^D^) is a π-electron-donating group (namely NR′_2_, PR′_2_, AsR′_2_, OR′, SR′, and SeR′). The analogues with R′ = H and R′ = Me at the main group centers have also been involved for each combination. The RSEs of these two kinds of radicals are highly similar (for parent substituents ranging between −13.3 and −1.9 kcal/mol, see Table S4, and for the methylated groups between −11.0 and −2.2 kcal/mol, see Table 2, with a regression coefficient of R^2^= 0.900, see Figure S7). Hence, we only concentrate on the methylated congeners in the following discussion.

**Table 2:** RSE Values of R^A^R^D^P^•^ Radicals Represented in a Matrix, Calculated at the (U)­DF-CCSD­(T)/aug-cc-pVTZ//(U)­ωB97X-D/6-311G** Level in kcal/mol ^a^

	NMe_2_	PMe_2_	AsMe_2_	OMe	SMe	SeMe
BMe_2_	–9.5 (0.531)	–3.4 (0.737)	–2.2 (0.868)	–8.8 (0.728)	–9.5 (0.691)	–9.0 (0.657)
AlMe_2_	–11.0 (0.625)	–5.6 (0.761)	–3.7 (0.863)	–8.7 (0.819)	–11.0 (0.773)	–10.9 (0.740)
GaMe_2_	–10.4 (0.612)	–5.2 (0.771)	–3.2 (0.869)	–8.2 (0.811)	–10.4 (0.769)	–10.6 (0.736)
SiMe_3_	–9.7 (0.700)	–4.5 (0.860)	–3.2 (0.916)	–7.5 (0.856)	–9.0 (0.822)	–8.7 (0.797)
GeMe_3_	–9.1 (0.735)	–4.5 (0.885)	–3.2 (0.938)	–7.5 (0.881)	–9.1 (0.845)	–8.8 (0.820)

a The spin populations at P are given in parentheses.

The most stable radicals are obtained by combining the π-electron-donating NMe_2_, SMe, or SeMe groups with any of the π-acceptors; the similar yet remarkable RSEs range in a window of −11.0 to −2.2 kcal/mol. Regardless of the pull group, the combinations with the R^D^ = OMe group lead to moderately stabilized radicals, while the R^D^ = PMe_2_ and AsMe_2_ substituents act as the least stabilizing, with RSEs spreading between −5.6 and −2.2 kcal/mol. These results underline that, generally, the push part is the governing factor, while the nature of the pull side has a minor effect on stability. Nevertheless, it is important to highlight that most captodative substitutions are clearly more effective than the symmetrical substitutions by π-donor groups, employed commonly to stabilize phosphinyl radicals in experimental studies.^19,50–53^

To identify possible additive or synergistic relationships, it is instructive to compare the RSEs of the R^A^R^D^P^•^ type radicals with the sum of RSE values obtained for the respective radicals containing one donor and one acceptor group separately: RSE­(R^A^HP^•^) + RSE­(R^D^HP^•^). The black dashed line in Figure 4 indicates additivity, and the points left from this line demonstrate a synergistic effect, that is, a more substantial stabilization is gained than simple additivity.

**Figure 4:**
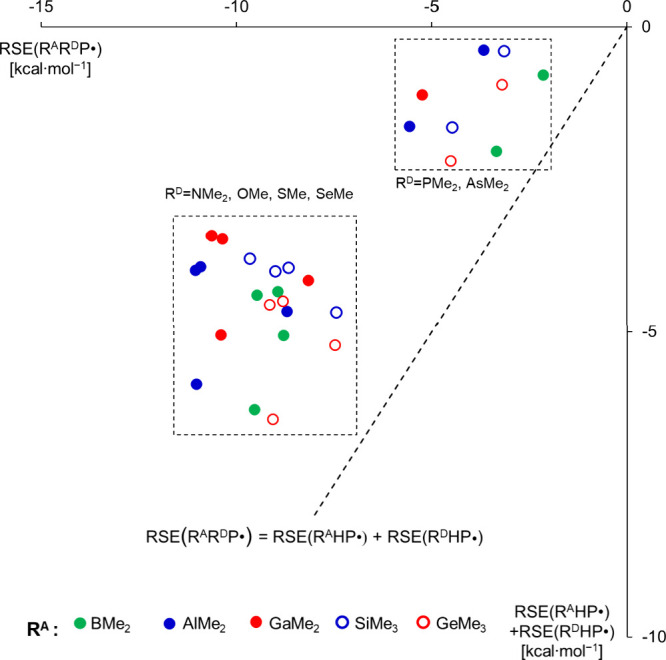
Comparison of RSEs of radicals exhibiting captodative substitutions [RSE­(R^A^R^D^P^•^)] with the sum of constituents, RSE­(R^A^HP^•^) + RSE­(R^D^HP^•^). A representation highlighting the different donor groups is shown in Figure S8.

Surprisingly, two well-separated clusters can be identified, depending on the push groups but independent of the nature of the pull substituents: (i) NMe_2_, OMe, SMe, and SeMe, and (ii) PMe_2_ and AsMe_2_.

All of the radicals containing the R^D^ = NMe_2_, OMe, SMe, and SeMe substituents form a distinct group: the RSEs are generally similar to that offered by the push group alone, and remain substantially negative in a moderately wide range between −11.0 and −7.5 kcal/mol, indicating significant electronic stabilization effects. In this cluster, remarkable synergistic effects can be observed (data points are shifted to the left from the diagonal). The radicals in the second cluster (R^D^= PMe_2_ and AsMe_2_) benefit from substantial extra stabilization, but the RSE values stay rather moderate compared to the former cases. These results highlight that the pyramidal arrangement around the P- or As-centers (see above) limits the donor ability of these groups, even if combined with a pull substituent.

To gain more insights into the nature of captodative effects, we visualized the Kohn–Sham singly occupied molecular orbitals (SOMOs) for two selected radicals (Me_2_B)­(Me_2_N)­P^•^ and (Me_3_Si)­(Me_2_N)­P^•^ and their monosubstituted constituents (Figure 5). The SOMO of the radical with captodative substitution can be interpreted as a direct combination of the orbital contributions obtained for the separated donor and acceptor counterparts. The extension of the lobe at the P-center toward the electron-deficient B or Si-centers indicates the electron-withdrawing character of these groups. The orbital energies also align between those of the constituents [although being closer to that of (Me_2_N)­HP^•^, in line with the chief role of the push group]. In addition, the decreased spin populations on the phosphorus atom [0.531 and 0.700 in (Me_2_B)­(Me_2_N)­P^•^ and (Me_3_Si)­(Me_2_N)­P^•^, respectively] reveal that the unpaired electron is effectively delocalized across the B/Si-, P-, and N-centers, contributing to the stability of these radicals. The delocalization effects are also reflected in the bond distances: the B–P bond length shortens from 1.932 Å in the monosubstituted counterpart (Me_2_B)­HP^•^ to 1.850 Å in the captodatively substituted radical. Parallely, the N–P bond also contracts but to a smaller extent, from 1.699 Å to 1.691 Å. Moreover, the B- and N-centers reside in planar coordination environments, agreeing with the strong π-accepting and π-donating properties, respectively, and the partial double bond character of the bonds.

**Figure 5:**
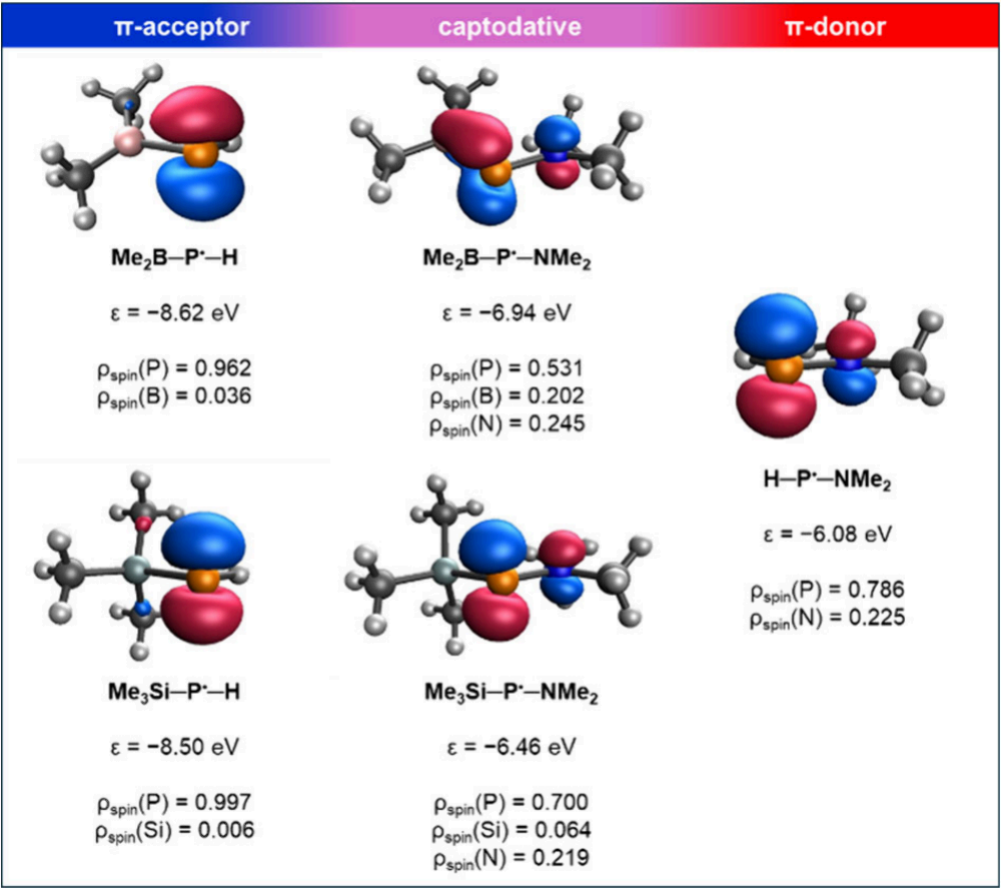
SOMOs of selected radicals (contour value: 0.2) with their orbital energies as well as their spin populations at the P-center.

To further bolster these findings, donor–acceptor interaction energies ΔE^(2)^ were calculated as detailed above, and the obtained values were compared with the individual components in the R^D^HP^•^ and R^A^HP^•^ type radicals. Compared to the interaction energies of 28.7 kcal/mol in (Me_2_B)­HP^•^ and 134.6 kcal/mol in (Me_2_N)­HP^•^, captodative substitutions induce additional stabilization: In (Me_2_B)­(Me_2_N)­P^•^, the unpaired electron at P (LP@P) is more efficiently abstracted by the empty 2p orbital at B (ΔE^(2)^_BD_ = 74.3 kcal/mol), and parallelly the donation from the lone pair at N into the half-empty 3p orbital at P (LP*@P) is also more effective, with an ΔE^(2)^_D_ of 197.3 kcal/mol. Remarkable but somewhat smaller ΔE^(2)^ energies are characteristic for the silicon-containing counterpart [from P lone electron to σ* (Si-C): ΔE^(2)^_BD_ = 11.5 kcal/mol and N lone pair into LP*@P: ΔE^(2)^_D_ = 133.9 kcal/mol]. These ΔE^(2)^ energies are consistent with the remarkable RSEs and low spin populations at the P-center determined for these radicals and support the effectiveness of the captodative type substitution.

### Dimerization

While the RSE value provides a useful relative scale, the dimerization energy calculated from total electronic energies (Δ*E*_dim_ according to the following equation: 2 R_2_P^•^ → R_2_P-PR_2_) can be referred to as a general thermodynamic measure for the global stability of a radical. Knowing that the entropy of an association reaction obtained in the gas-phase calculations is significantly overestimated compared to those determinable experimentally in a real solution environment,^55,56^ in our methodology, we will consider a radical to be stable against dimerization if it has a positive dimerization energy (Δ*E*_dim_ > 0).^46,49^ Note that this is a much stricter criterion than the positive dimerization Gibbs free energy, Δ*G*_dim_ > 0 (as obtained for the same dimerization equation given above, for more details see the Computational Details). The radicals with Δ*E*_dim_ < 0 but Δ*G*_dim_ > 0 would likely lead to (partial) dimerization under experimental conditions (since the dimerization Gibbs free energy in a real solution is expected to lie between Δ*E*_dim_ and Δ*G*_dim_). However, they might be detected as persistent species, especially at higher temperatures. In addition, the Δ*E*_dim_ values reported in this section have not been corrected for the basis set superposition error (BSSE), as it virtually overstabilizes the dimer in the calculations, and this would shift the Δ*E*_dim_ values to a less negative direction. Thus, the reported dimerization energies correspond to the “worst-case scenarios″. Nevertheless, the BSSE is typically in a few kcal/mol range (1.5 to 5.1 kcal/mol) even for bulky substituents studied in this work.

We determined the dimerization energies (and Gibbs free energies) for all of the parent and methylated phosphinyl radicals introduced above, exhibiting versatile functionalizations (including either donor or acceptor substituents, as well as their combinations presented above) at the ωB97X-D/6-311G** level of theory (Tables S5 and S6), taking various possible conformations determined through systematic conformational searches into account. Notably, the same DFT level of theory was previously found to deliver highly similar results to the state-of-the-art LNO-CCSD­(T) method, which was thoroughly tested for dimerization reactions of a set of similarly bulky phosphinyl radicals.^46^ In few cases of captodative substitutions [e.g., for (H_2_B)­(H_2_N)­P^•^, (H_2_Al)­(H_2_N)­P^•^, and (Me_2_Ga)­(MeO)­P^•^, etc.], the geometry optimizations of the dimers ended up in highly distorted geometries with additional bonds formed between the donor and acceptor sites beside the P-P bond, offering extra stabilization to the dimers. For clarity, we excluded these cases from our evaluation.

To benchmark our calculations, we obtained the dimerization energies and Gibbs free energies for the radicals shown in Figure 1a, which are persistent but dimerize in the solid state. The Δ*G*_dim_ = −4.8 (Δ*E*_dim_ = −30.7 kcal/mol kcal/mol, BSSE = 2.9 kcal/mol) for the (TMS_2_CH)_2_P^•^ as well as Δ*G*_dim_ = + 9.0 kcal/mol (Δ*E*_dim_ = −19.6 kcal/mol and BSSE = 2.9 kcal/mol) for (TMS_2_N)_2_P^•^ values are consistent with the persistent nature of those radicals.

The Δ*E*_dim_ energies and Δ*G*_dim_ Gibbs free energies correlate linearly with RSE (Figure 6), outlining that the RSE values are, in general, realistic measures for estimating the thermodynamic stability of the radical. Extrapolating the graph to Δ*E*_dim_ = 0 gives a critical RSE value of −32.2 kcal/mol, which is expected to provide the necessary stabilization that prohibits dimerization. The slope of the regression line (1.96) indicates that practically double the amount of RSE is transferred to the dimerization energy in the form of an increase, because each of the two radicals contributes individually to the change in dimerization energy, illuminating the importance of electronic effects. However, it is obvious that the major factor affecting the thermodynamics of dimerization is the remarkable intrinsic strength of the P-P bond (bond dissociation energy in H_2_P-PH_2_:^57^ 61.2 kcal/mol, cf. the intercept of the trend line is −63.1 kcal/mol). In earlier reports, the RSE of the vanadium-supported radicals in Figure 1d and 1e were calculated to be −26.3 and −26.5 kcal/mol, respectively, at a slightly different DFT level of theory. These remarkably low values unveil that the extreme redox-activity of the transition metal centers is needed to approach the critical RSE value. The optimal “target″ RSE value also highlights that the electronic effects of main group substituents without steric congestion are insufficient to hamper the dimerization of simple phosphinyl radicals.

**Figure 6:**
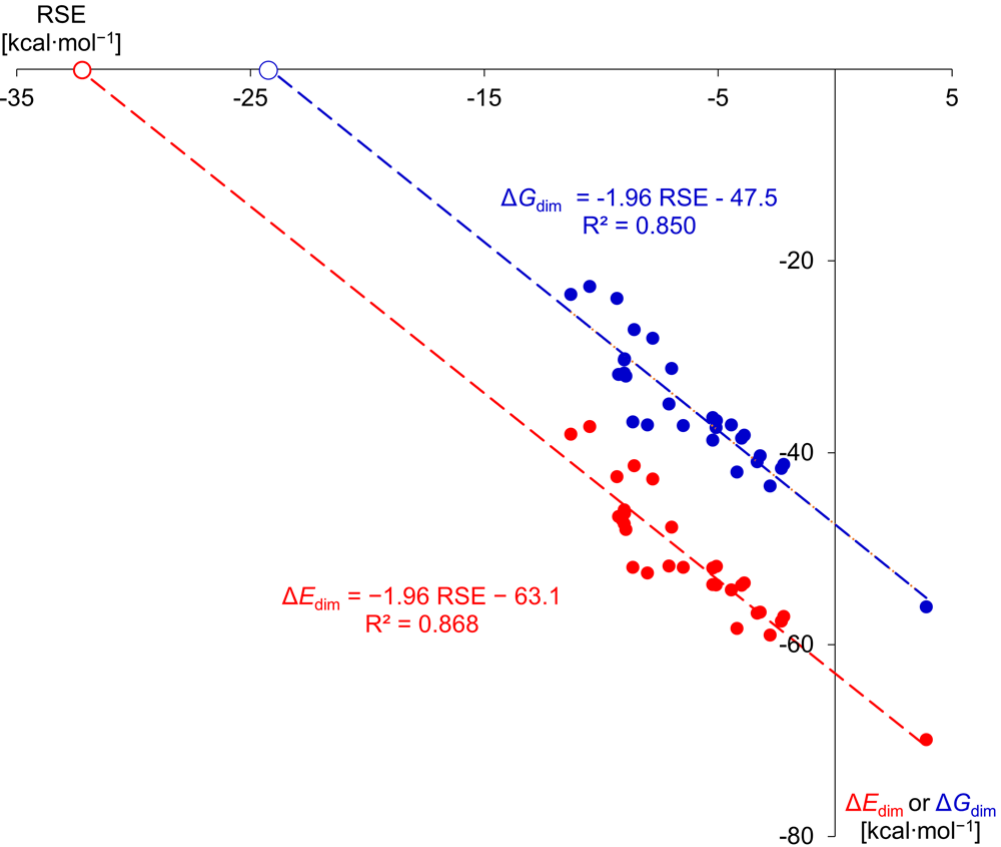
Plot of ΔE_dim_ against RSE (the unfilled red and blue markers represent the extrapolated values to ΔE_dim_ = 0 and ΔG_dim_ = 0, respectively)

### Steric Effects

To spot the possibilities and limitations of steric effects, we will only focus on captodative substitutions that further tune the stability of the radical. To our knowledge, phosphinyl radicals benefiting from such captodative substitutions have not been reported so far, although this effect may offer new possibilities in the synthetic realization.

The results discussed above reveal that the stabilization effects are governed predominantly by the push side, especially in the case of amino- and chalcogen-containing groups. Among these, the amino groups are special because, unlike chalcogens, nitrogen can accommodate two substituents, and also provides more stabilization than the heavier pnictogens since its lone electron pair is more accessible electronically due to lower pyramidalization.

In addition, the nature of the pull group is also important in terms of possible additional tuning by steric protection. Owing to the prominent electron deficiency (Lewis acidity) of the B-, Al-, or Ga-centers, in these cases, the geometry optimizations resulted in dimers that are overstabilized by additional dative bonds forming between the push and pull groups (see above). Therefore, these pull substituents were not considered further. On the other hand, silicon, the diagonal sibling of boron, is also capable of π-withdrawal (via the σ* Si-R′ orbitals) but is less Lewis acidic. Importantly, in the optimized structures of the dimers, no N→Si dative bonds are detectable, even with the smallest substituents. As a further advantage, silyl (SiR′_3_) groups are synthetically accessible with various R′ substituents, and, in contrast to the triel elements (B, Al, and Ga), silicon has the ability to accommodate three bulky groups, which can better shield the P-center. In this respect, germyl (GeR′_3_) groups could also be utilized; however, they are less available experimentally than the silyl counterparts.

Based on all of these considerations, we narrowed down the possibilities to the most feasible congener, (R′_3_Si)­(R_2_N)­P^•^, to delve into a systematic investigation of steric effects. Importantly, halogen-substituted phosphines decorated simultaneously by a silyl and an amino substituent are synthetically accessible through halogenation of bis­(trialkylsilyl)­phosphines (e.g., using hexachloroethane as a reagent),^58–62^ and even commercially available examples can also be found. Available phosphines with the general formula XP­(SiR′_3_)­(NR_2_) [X = Cl, Br, I] signify the prospective synthetic accessibility of the corresponding radicals through reduction (note that strong reductants such as alkaline metals, potassium graphite, or sodium naphthalenide are commonly used for the reduction of halophosphines).^35,38,42,45^

A set of sterically demanding groups (R and R′) was selected considering their synthetic accessibility, volume, and shape: As planar (or 2D) groups phenyl (**Ph**) and pentafluorophenyl (**PFP**), and as spherical (or 3D) groups tertiary butyl (*t* **Bu**), trimethylsilyl (**TMS**), and perfluoro­(trimethylsilyl) (**PFS**) were chosen (Figure 7a). Note that both silyl and amino groups are synthetically accessible with the majority of these bulky substituents. Taking a total of 25 combinations of these bulky groups, the dimerization energies of the radicals were calculated at the ωB97X-D/6-311G** level of theory (Table 3).

**Figure 7:**
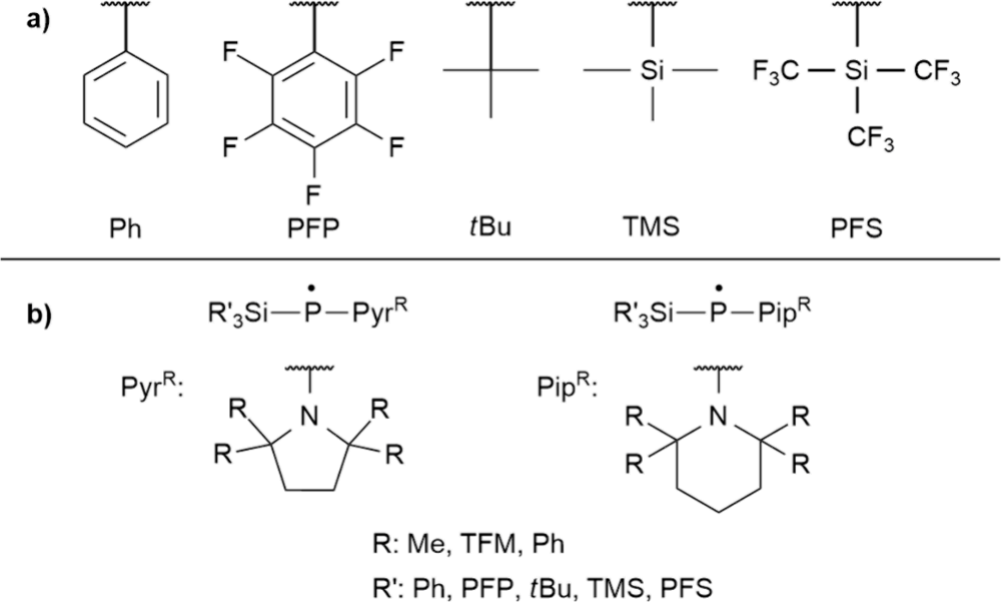
a) Sterically demanding bulky substituents involved in our study at both N and Si centers of the R′_3_Si-P^•^-NR_2_ radicals, and b) cyclic substituents encapsulating the N-center.

**Table 3:** Δ*E*_dim_, Δ*G*_dim_, Δ*E*_prep_, Δ*E*_int_, and Δ*E*_disp_ Values Obtained at (U)­ωB97X-D/6-311G** Level of Theory in kcal/mol, for (R′_3_Si)­(R_2_N)­P^•^

Radical	R′	R	Δ*E*_dim_	Δ*G*_dim_	Δ*E*_int_	Δ*E*_prep_	Δ*E*_disp_
**1**	Ph	Ph	–57.3	–37.1	–78.3	21.1	–35.2
**2**	Ph	PFP	–54.8	–31.4	–88.7	33.9	–36.3
**3**	Ph	*t*Bu	–51.9	–26.2	–64.2	12.3	–34.2
**4**	Ph	TMS	–54.5	–27.4	–69.3	14.7	–37.2
**5**	Ph	PFS	–29.3	–1.5	–67.2	38.0	–38.7
							
**6**	PFP	Ph	–51.3	–27.8	–80.4	29.1	–38.1
**7**	PFP	PFP	–37.3	–12.5	–78.9	41.5	–37.5
**8**	PFP	*t*Bu	–33.2	–8.8	–59.2	26.0	–35.7
**9**	PFP	TMS	–48.4	–22.8	–64.4	16.0	–38.6
**10**	PFP	PFS	–12.3	17.8	–65.4	53.1	–41.5
							
**11**	*t*Bu	Ph	–41.8	–20.0	–59.5	17.8	–31.9
**12**	*t*Bu	PFP	–32.0	–10.8	–50.3	18.2	–32.1
**13**	*t*Bu	*t*Bu	–1.8	24.6	–34.0	32.2	–30.5
**14**	*t*Bu	TMS	–17.2	9.1	–47.7	30.4	–35.2
**15**	*t*Bu	PFS	–^a^	–^a^	–^a^	–^a^	–^a^
							
**16**	TMS	Ph	–52.7	–23.5	–67.6	14.9	–35.1
**17**	TMS	PFP	–44.9	–18.3	–71.1	26.2	–38.4
**18**	TMS	*t*Bu	–17.7	11.8	–46.0	28.3	–31.9
**19**	TMS	TMS	–29.1	3.3	–55.9	26.8	–37.8
**20**	TMS	PFS	17.6	51.2	–50.6	68.2	–40.0
							
**21**	PFS	Ph	–18.2	8.6	–49.0	30.8	–34.6
**22**	PFS	PFP	–12.8	18.5	–57.0	43.1	–37.6
**23**	PFS	*t*Bu	–^a^	–^a^	–^a^	–^a^	–^a^
**24**	PFS	TMS	–^a^	–^a^	–^a^	–^a^	–^a^
**25**	PFS	PFS	–^a^	–^a^	–^a^	–^a^	–^a^

a No dimer could be optimized.

To map the conformational space, possible relative arrangements of the substituents were scanned through a conformer-rotamer sampling tool (for details, see the Computational Details section), and the most stable structures were subjected to further geometry optimizations at the DFT level. In a few cases (radicals **23**, **24**, and **25**), however, no minima corresponding to the dimeric forms could be located on the potential energy surface. Although it is difficult to prove that a radical does not form a dimer, that is, no corresponding minimum can be found on the potential energy surface, we made several attempts to locate such minima. First, we tested a large number of starting geometries, but the geometry optimizations resulted in bond cleavage either at the P-P bond or at a functional group, and the molecule lost its integrity in all these cases. Alternatively, we also endeavored to obtain reasonable starting geometries for the dimers using geometrical constraints (by fixing the P-P bond distance). However, after releasing these restrictions, the optimization runs resulted in the dissociation of the dimer in all of these cases. Finally, to exclude the possibility of dimer formation, we also performed relaxed scan calculations in which the distance between the P-centers of two separated radicals was gradually decreased. However, in these cases, we again detected the cleavage of a covalent bond but in a different moiety.

Among these combinations, the dimerization energies occupy a wide range from Δ*E*_dim_ = −57.3 to + 17.6 kcal/mol. In general, radicals containing a planar (2D) phenyl or pentafluorophenyl group either at one or both sides exhibit highly to moderately exothermic dimerization energies (−57.3 to −29.3 kcal/mol for radicals **1**–**9**, **11**, **12**, **16**, and **17**), indicating a considerable affinity of the radical toward dimerization. The limited steric effects of these planar groups likely arise from their moderate volumes (V = 117.3 Å^3^ and V = 128.0 Å^3^ for Ph and PFP, respectively), and they can undergo rotation for repositioning more easily than the spherical groups, providing less shielding to the reactive P-center. Interestingly, if the same planar groups are explicitly combined with the PFS group (regardless whether on the pull or push side), such as in radicals **10**, **21**, and **22**, significantly less negative Δ*E*_dim_ energies (−18.2 to −12.8 kcal/mol) can be achieved, and the positive Δ*G*_dim_ values (+8.6 to + 18.5 kcal/mol) suggest that these radicals could possibly be persistent, especially at higher temperature. Overall, however, the 2D-type substitution seems inferior.

On the other hand, radicals with spherical (3D) substituents on both sides exhibit less exothermic dimerization reactions compared to the former cases. In several instances, positive dimerization Gibbs free energies were obtained (radicals **13**, **14**, and **18**–**22**) or the dimeric structures underwent spontaneous dissociation during the optimization (radicals **15**, and **23**–**25**).

In general, the dimerization energies of radicals containing the *t*Bu substituent (e.g., **13** and **14**) are less negative (by at least 10 kcal/mol) compared to the corresponding ones featuring TMS groups (e.g., **18** or **19**). This difference in Δ*E*_dim_ arises from the fact that the *t*Bu group is more compact (V = 112.8 Å^3^ ^49^ ) than the TMS (V = 130.9 Å^3^ ^49^), protecting the reactive center more effectively. Even though the Δ*E*_dim_ values are negative in these cases, the positive Δ*G*_dim_ values indicate likely reversible dimerization, and these radicals may be monomeric at high temperatures.

Highlighting the beneficial effect of fluorination in the bulky organic substituent, for the radicals exhibiting the PFS group on at least one side combined with any of the spherical groups (radical **15**, **23**, **24**, and **25**), no dimeric structures could be optimized. Additionally, a highly positive dimerization energy was obtained for radical **20** (Δ*E*_dim_ = +17.6 kcal/mol). Based on the results, all of these phosphinyl radicals seem synthetically accessible.

Our findings clearly show that planar substituents at either or both sides of the P-center do not provide enough steric bulk to stabilize the monomeric radicals effectively. In contrast, spherical groups offer a remarkable improvement in steric congestion. However, the conformational flexibility of these spherical groups may still help them to find the most appropriate position in the dimers, which leads to a lowering in relative energy. In addition, the N-centers are only tricoordinated, and are therefore less congested than the tetra-coordinated Si-centers. To solve this problem, we anticipate that the conformational flexibility of the push side can be reduced by applying cyclic backbones. Specifically, we explored 15 combinations carrying the previous groups at Si and 5-membered pyrrolidine (Pyr) or 6-membered piperidine (Pip) cores, modified with four methyl (Me), trifluoromethyl (TFM), or phenyl (Ph) groups at their α-positions (Figure 7b, and data in Table S7). Notably, such substituents can also be realized synthetically.

Conspicuously, for the majority of the radicals with these cyclic substituents, the dimer dissociated during the optimization runs, and the dimeric form could only be optimized in a few cases (see Table S7), depending on the substitutions at the Si-center. Remarkably, for all combinations exhibiting R′ = *t*Bu groups at the Si-center and any heterocyclic push groups (Pyr^R^, Pip^R^), no dimeric structures could be obtained, suggesting that these radicals would be monomeric also in the experiments. In contrast, employing the less compact TMS group at the Si acceptor site leads to a more complicated picture, since the feasibility of the dimerization also depends on the substituent attached to the N-heterocycle. Indeed, with the smallest Me groups on the ring (Pyr^Me^, Pip^Me^) the dimerization is still exothermic [for (TMS_3_Si)­(Pyr^Me^)­P^•^ and (TMS_3_Si)­(Pip^Me^)­P^•^, Δ*E*_dim_ = −35.9 and −25.0 kcal/mol, respectively]. However, changing the functional group at the α-position of the rings to TFM or Ph hinders the dimerization successfully, and all of these combinations afford radicals that are stable against dimerization. The fluorination of the TMS substituent also has a beneficial effect on the stability of the monomeric radical: when the Si-center is decorated by R = PFS groups, all of the combinations with any cyclic donor group result in the dissociation of the dimer.

Overall, the combinations that include either the *t*Bu or PFS groups at the pull side besides any N-heterocyclic substituent effectively protect the P-center of the radical against dimerization. Our results demonstrate that embedding the N-center into heterocyclic frameworks is a highly efficient way to cancel the thermodynamic driving force of the dimerization.

To gain more insights into the quantitative aspects of the dimerization process, the distortion-interaction model^63^ was applied to break down the components of dimerization energy into preparation (or distortion) energy (Δ*E*_prep_) and interaction energy (Δ*E*_int_). The preparation energy Δ*E*_prep_ stands for the energy required to deform the geometries of the two (formerly identical) monomers to achieve the suitable orientation to form the dimer. On the other hand, Δ*E*_int_ describes the energy release that stems from the interactions developing between two “prepared″ monomers upon dimerization. Among the stabilizing interactions, dispersion forces may provide a meaningful contribution to Δ*E*_int_; therefore, the dispersion energy (Δ*E*_disp_) was estimated using Grimme’s D3 model.^64^

Both the Δ*E*_int_ and Δ*E*_prep_ show an increasing trend with the rising Δ*E*_dim_ values (Figure 8). This indicates that stabilizing the radical in its monomeric form requires maximizing the preparation energy, and, in parallel, the interaction energy ought to be canceled out by Pauli repulsion developing between the bulky substituents. Interestingly, the dispersion energies are rather similar for all of the radicals, fluctuating around Δ*E*_disp_ ≈ −40 kcal/mol, giving a practically constant and substantial contribution to the interaction energies (Figure 8). This most likely arises from the structural similarity between the molecules (considering both the monomeric and dimeric forms), independent of the actual substituents. Nevertheless, the magnitude of dispersion energies implies a significant contribution that needs to be compensated by the preparation energy, which requires well-tailored substituents at both sides of the phosphinyl radical.

**Figure 8:**
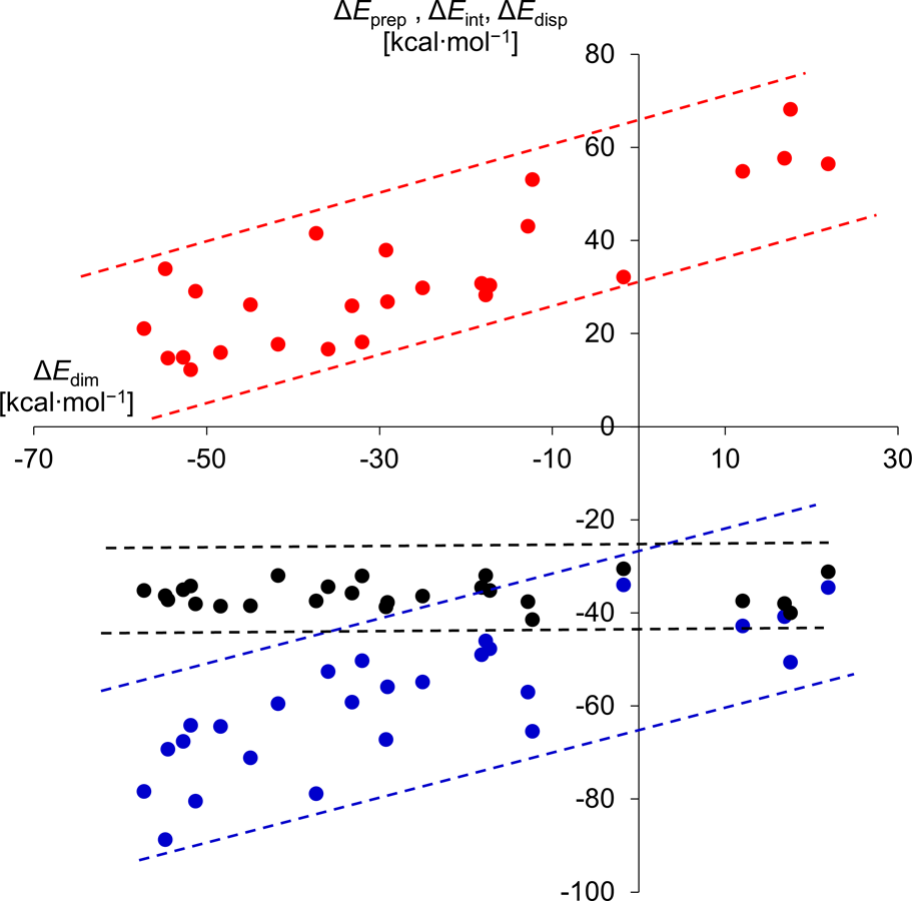
Plot of Δ*E*_int_ (blue), Δ*E*_prep_ (red), and Δ*E*_disp_ (black) as a function of Δ*E*_dim_.

## 3 Conclusion

To achieve a conceptual understanding of the stability of phosphinyl radicals, in this computational study, we utilized a systematic approach to explore various electronic and steric effects that influence the stability of phosphinyl radicals. We scrutinized the electronic effects of a large set of simple substituents using radical stabilization energies (RSEs) on a relative scale. The comparison of the RSE values reveals that the electronic stabilization effects are additive for the majority of the substituents, except for the most efficient π-donors (NR_2_, OR, SR, and SeR) that suffer from a saturation effect. The RSEs of radicals with captodative substitution outline effective stabilization, which is mainly driven by the donor side (particularly those incorporating amino or chalcogenyl functional groups) rather than by the acceptor side. Furthermore, the observed additivity and saturation effects depend on the differing strengths of stabilization interactions offered by π-donor and π-acceptor substituents: π-donors stabilize the radical through donation from the lone pair at N, O, S, or Se into the half-empty 3p orbital at phosphorus, whereas π-acceptor groups primarily alter the α-electron distribution via donation from the half-filled 3p-type α-orbital at P into the vacant p-orbital or σ* orbitals at the acceptor center.

On the basis of dimerization energies, however, the electronic effects alone are insufficient to hinder the dimerization, and bulky substituents are also necessary for stabilizing the monomeric radicals. To screen the possibility of steric protection, the (R′_3_Si)­(R_2_N)­P^•^ radical exhibiting captodative substitution has been selected for further investigations. Evaluation of the dimerization energies shows that substitutions by spherical groups provide better steric protection than planar groups, leading to either positive dimerization energies or the lack of local minima corresponding to the dimeric structures on the potential energy surface. Knowing that open-chain substituents can easily reposition to adopt the optimal orientation, we took a step further to increase the steric bulk at the less congested donor center (nitrogen) by introducing 5- or 6-membered N-heterocycles as π-donor sites. These cyclic structures can fix the position of the substituents in their α-positions to effectively shield the P-centers. Despite the substantial stabilizing effect of electronic communication offered by the captodative substitution pattern, the steric effects are clearly more decisive in stabilizing the monomeric radicals, as the high strength of the P–P covalent bond and the weak secondary (dispersion) interactions arising between the ligands at the two P-centers need to be compensated by steric repulsion.

The presented computational analysis identifies several phosphinyl radicals that are expected to remain monomeric and, therefore, are promising candidates for experimental exploration in the future. Specifically, the phosphinyl radicals decorated by N-heterocyclic push groups (Pyr^R^ or Pip^R^) paired with *t*Bu or perfluorotrimethyl silyl substituents at the Si-center on the pull side effectively prevent dimer formation. Apart from these, a set of further realistic candidates exhibiting various acyclic protecting groups of high steric demand was identified. Importantly, as phosphines substituted by silyl, amino, and halogen functionalities simultaneously are known in the literature, the targeted phosphinyl radicals seem achievable synthetically.

Our study also highlights that achieving stable phosphinyl radicals presents significant difficulties, yet the captodative substitution strategy offers a promising pathway to overcome these obstacles. With fine-tunable steric protection using bulky functional groups, this methodology may enable the design of radicals with enhanced stability. While challenges remain, our findings may help future experimental research targeting the synthesis of stable phosphinyl radicals.

## 4 Computational Details

For DFT calculations, the Gaussian 16,^65^ while for coupled-cluster calculations, the MRCC 2022 ^66,67^ program packages were used. The ωB97X-D method combined with the 6-311G** basis set was applied for all geometry optimizations, which was previously employed successfully for studying similar radicals.^46^ In addition, we have tested this method using the functionals M06-2X and B3LYP-D3, which resulted in highly similar geometries, as well as the calculated RSE values also show excellent correlations. The effect of basis set was also examined employing the aug-cc-pVQZ basis set, which gave highly similar energetic data to those obtained with the smaller Pople-type basis set. Harmonic vibrational analyses were carried out at the (U)­ωB97X-D/6-311G** level, and in all the cases, all positive eigenvalues of the Hessian matrix indicated local minima. The spin populations were obtained using Mulliken population analysis at the UωB97X-D/6-311G** level. Single-point energy calculations were obtained at the (U)­DF-CCSD­(T)/aug-cc-pVTZ level of theory using the geometries optimized at the (U)­ωB97X-D/6-311G** level.

The dimerization energies were calculated at (U)­ωB97X-D/6-311G** level of theory, which was thoroughly tested previously using LNO-CCSD­(T) calculations delivering highly similar results to this DFT level. To search for the conformers with the lowest relative energy, possible conformers were scanned using the conformer-rotamer sampling tool of the CREST program package.^68^ Then, geometry optimizations were started from various initial geometries obtained from these conformational searches to find the most stable conformers. The dimerization energies were determined by comparing the most stable conformers of the monomers and dimers. The Δ*E*_dim_ values were calculated from the total electronic energies of components of the reaction, and the thermal corrections to Gibbs free energy were obtained at 298.15 K and 1 atm using standard procedures implemented in the program code by taking the translational, rotational, vibrational, and electronic entropy terms, as well as pΔ*V*contribution into account.

In several cases, the geometry optimizations led to disintegrated dimers, which were characterized by the cleavage of substituents or failure to form the P–P bond, indicating that these dimers are inaccessible on the potential energy hypersurface. To cross-check that geometries might not be disintegrated, certain characteristic bond distances were fixed (depending on the need, for example, P-P, P-E, or E-R bonds that were cleaved during the initial optimizations), and then these geometries were optimized with constraints. Following these preoptimizations, all restrictions were released and the geometries were reoptimized; however, fragmentation of the dimer was observed in all of these cases.

To obtain the distortion and interaction energies, single-point energy calculations were performed at the above-described DFT level on the separated geometries of the radicals arising from the dimeric structures. Dispersion contributions were estimated using Grimme’s D3 empirical dispersion correction model.^64^ The basis set superposition error (BSSE) was determined using the counterpoise correction, implemented in Gaussian 16.

In addition to gas-phase calculations, we examined the solvation effects of hexane, toluene, THF, and diethyl ether (commonly employed as a solvent in the experiments involving P-centered radicals) using the SMD model^69^ for a representative set of bulky substituted phosphinyl radicals (radical **1**, **5**, **9**, **13**, **17**, **20**, **34**, and **39**). The Δ*E*_dim_ values obtained in solution showed an excellent correlation with those calculated in the gas phase, with typical deviations of around 1 to 3 kcal/mol (R^2^ = 0.99 for all cases; see Table S8 and Figure S9).

Natural bond orbital analyses were performed using NBO version 7.0.^70^

Molecular orbitals were visualized using the IQmol software package.^71^

## Supplementary Material



## Notes

Additional figures and tables, xyz coordinates, and total energies of optimized structures (PDF)
