# Supplementary material for: Stabilization Effects in Phosphinyl Radicals: The Scope of the Donor, Acceptor, and Captodative Functionalization
Source: Inorg Chem. 2025 Nov 17;64(47):23202–14. doi: 10.1021/acs.inorgchem.5c03515 (PMC12673518; doi:10.1021/acs.inorgchem.5c03515)
Supplement: Supplementary file 1 [file ic5c03515_si_001.pdf]

Supporting Information

Stabilization effects in Phosphinyl Radicals: The scope of the donor, acceptor, and captodative functionalization

Pelin Kaymak[a], Zoltán Benkő [a,b,*]

[a] Department of Inorganic and Analytical Chemistry, Faculty of Chemical Technology and Biotechnology, Budapest University of Technology and Economics, H-1111 Budapest, Hungary; e-mail: benko.zoltan@vbk.bme.hu

[b] HUN-REN-BME Computation Driven Chemistry Research Group, H-1111 Budapest, Hungary.

LIST OF TABLES

Table S1: *RSE values at the (U)ωB97X-D/6-311G** level in kcal·mol^{-1}, for the RHP• and R_2P• type radicals with R=EH_n (n=0-3) and R= EMe_n (n=1-3)* S4

Table S2: *RSE values at the (U)M06-2X/6-311G**, (U)B3LYP-D3/6-311G** (U)ωB97X-D/aug-CC-pVQZ level in kcal·mol^{-1}, for the RHP• and R_2P• type radicals with R= EMe_n (n=1-3)* S4

Table S3: *The donor-acceptor interaction energies $ΔE^{(2)}$ obtained for the RHP• and R_2P• type radicals in kcal·mol^{-1}. For explanation, see the main text and Figure 3.* S5

Table S4: *RSE values of R^AHP•, R^DHP•, and R^AR^DP• are represented in a matrix for R=EH_n, calculated at the (U)DF-CCSD(T)/aug-cc-pVTZ//(U)ωB97X-D/6-311G** level in kcal·mol^{-1}. The spin populations at the P-center are given in parentheses* S5

Table S5*: The dimerization energies and Gibbs free energies of R_2P• type radicals for R=EH_n at the (U)ωB97X-D/6-311G** level of theory in kcal·mol^{-1}* S6

Table S6: *Dimerization energies of R^AR^DP• type (R= EH_n, EMe_n) obtained at the (U)ωB97X-D/6-311G** level of theory.* S7

Table S7: *Numbering of the radicals and $ΔE_{dim}$, $ΔG_{dim}$, $ΔE_{prep}$, $ΔE_{int}$, and $ΔE_{disp}$ values obtained at the (U)ωB97X-D/6-311G** level of theory, for R= tBu, TMS, and TFMS and R'= Pyr^{Me}, Pyr^{TFM}, Pyr^{Ph}, Pip^{Me}, Pip^{TFM}, and Pip^{Ph}* S8

Table S8: *$ΔE_{dim}$ values for different solvents obtained with the SMD solvation model at the (U)ωB97X-D/6-311G** level of theory* S8

LIST OF FIGURES

Figure S1: *Plot of RSE vs spin populations $ρ_{spin}(P)$ calculated at the (U)DF-CCSD(T)/aug-ccpVTZ//ωB97X-D/6-311G** level for the R_2P• (filled markers) and RHP• (unfilled markers) type radicals with R=EH_n* S9

Figure S2*: Plot of RSEs obtained at the (U)DF-CCSD(T)/aug-cc-pVTZ//(U)ωB97X-D/6-311G** level vs the Pauling electronegativity (EN) of the central atom E in the R substituent for the RHP• type radicals* S9

Figure S3: *Plot of RSEs obtained at the (U)DF-CCSD(T)/aug-cc-pVTZ//(U)ωB97X-D/6-311G** level vs the ionization energy (IE) of the central atom E in the R substituent for the RHP• type radicals* ... S10

Figure S4: *Correlation between the RSE values obtained for R=EH_n and R=EMe_n. Unfilled and filled markers correspond to the RHP• and R_2P• type of radicals. Energies were obtained at the (U)DF-CCSD(T)/aug-cc-pVTZ//(U)ωB97X-D/6-311G** level of theory.* S10

Figure S5*: RSEs obtained at the (U)DF-CCSD(T)/aug-cc-pVTZ//(U)ωB97X-D/6-311G** level vs the Pauling electronegativity (EN) of the central atom E in the R substituent for the R_2P• type radicals* . S11

Figure S6*: RSEs obtained at the (U)DF-CCSD(T)/aug-cc-pVTZ//(U)ωB97X-D/6-311G** level vs the ionization energy (IE) of the central atom E in the R substituent for the R_2P• type radicals* S11

Figure S7: *Relationship between the RSEs of R^AR^DP• for EH_n and EMe_n type substitution obtained at the (U)DF-CCSD(T)/aug-cc-pVTZ//(U)ωB97X-D/6-311G** level*...*S12*
Figure S8: *Comparison of RSEs of radicals exhibiting captodative substitutions [RSE(R^AR^DP•)] with the sum of constituents, RSE(R^AHP•)+RSE(R^DHP•)*..*S12*
Figure S9: *Comparison of dimerization energies calculated in the gas-phase and with SMD solvent models (U)ωB97X-D/6-311G** level*...*S13*

TOTAL ENERGIES AND OPTIMIZED GEOMETRIES IN CARTESIAN COORDINATES S13
1) RHP• type radicals and their corresponding phosphines for R=EH_n S13
2) RHP• type radicals and their corresponding phosphines R=EMe_n S18
3) R_2P• type radicals and their corresponding phosphines R=EH_n S24
4) R_2P• type radicals and their corresponding phosphines R=EMe_n.. S30
5) R^AR^DP• type radicals and their corresponding phosphines R=EH_n S39
6) R^AR^DP• type radicals and their corresponding phosphines R=EMe_n.. S51
7) $[R_2P•]_2$ dimers for R=EH_n.. S74
8) $[R^AR^DP•]_2$ dimers for R=EMe_n S83
9) Phosphinyl radicals of the formula (R'$_3$Si)(R_2N)P• and their dimers [(R'$_3$Si)(R_2N)P•]$_2$........... S98

Table S1: *RSE values at the (U)ωB97X-D/6-311G** level in kcal·mol^{-1}, for the RHP• and R_2P• type radicals with R=EH_n (n=0-3) and R= EMe_n (n=1-3)*

E	R= EH_n		R=EMe_n	
	RHP•	R_2P•	RHP•	R_2P•
F	-4.6	-8.6	-	-
Cl	-5.5	-10.5	-	-
Br	-6.0	-11.3	-	-
O	-6.6	-7.0	-8.2	-8.5
S	-8.4	-7.8	-6.9	-8.8
Se	-8.9	-9.3	-7.3	-10.1
N	-7.8	-5.1	-9.4	-6.3
P	-4.5	-7.1	-4.7	-6.7
As	-3.7	-6.5	-3.5	-5.8
C	-1.9	-3.2	-1.2	-1.6
Si	-1.2	-2.2	-1.0	-1.8
Ge	-1.3	-2.3	-1.0	-1.9
B	1.9	3.9	1.4	2.2
Al	3.2	-0.5	0.02	-0.7
Ga	0.3	-0.6	0.04	-0.8

Table S2: *RSE values at the (U)M06-2X/6-311G**, (U)B3LYP-D3/6-311G** (U)ωB97X-D/aug-CC-pVQZ level in kcal·mol^{-1}, for the RHP• and R_2P• type radicals with R= EMe_n (n=1-3)*

E	R=EMe_n					
	M06-2X/6-311G**		B3LYP-D3/6-311G**		ωB97X-D/aug-cc-pVQZ	
	RHP•	R_2P•	RHP•	R_2P•	RHP•	R_2P•
O	-7.0	-7.9	-8.2	-8.8	-7.9	-8.3
S	-5.8	-8.1	-7.2	-9.0	-7.4	-9.8
Se	-6.0	-9.3	-7.6	-10.2	-7.4	-10.3
N	-8.1	-6.2	-9.9	-6.8	-9.4	-6.2
P	-3.6	-5.5	-4.9	-7.1	-5.4	-7.8
As	-2.9	-4.9	-3.5	-5.7	-3.6	-5.7
C	-1.5	-2.3	-1.2	-1.4	-1.1	-1.4
Si	-0.9	-1.7	-1.1	-2.0	-0.9	-1.8
Ge	-0.8	-1.6	-1.2	-2.4	-0.9	-1.8
B	1.0	1.8	1.4	2.6	1.5	2.5
Al	-0.2	-0.4	-0.1	-1.0	0.1	-0.6
Ga	0.1	-0.2	-0.1	-1.3	0.3	-0.4

Table S3: *The donor-acceptor interaction energies $\Delta E^{(2)}$ obtained for the RHP• and R_2P• type radicals in kcal·mol^{-1}. For explanation, see the main text and Figure 3.*

R	$\Delta E^{(2)}_D$ (RHP•)	$\Delta E^{(2)}_D$ (R_2P•)
**F**	25.6	43.8
**Cl**	24.4	45.0
**Br**	25.3	46.8
**OH**	40.9	25.8
**SH**	41.8	65.8
**SeH**	41.6	66.0
**NH_2**	62.1	49.8
**PH_2**	11.4	18.6
**AsH_2**	6.6	23.3
**CH_3**	6.1	13.7
	$\Delta E^{(2)}_{BD}$ (RHP•)	$\Delta E^{(2)}_{BD}$ (R_2P•)
**BH_2**	35.6	47.0
**AlH_2**	13.9	14.3
**GaH_2**	3.5	11.8
**SiH_3**	7.7	14.9
**GeH_3**	6.9	13.0

Table S4: *RSE values of R^AHP•, R^DHP•, and R^AR^DP• are represented in a matrix for R=EH_n, calculated at the (U)DF-CCSD(T)/aug-cc-pVTZ//(U)ωB97X-D/6-311G** level in kcal·mol^{-1}. The spin populations at the P-center are given in parentheses.*

R^DHP• / R^AHP•		**O**	**S**	**Se**	**N**	**P**	**As**
		-5.8	-7.9	-7.8	-6.5	-3.8	-2.7
**B**	2.5	-9.7 *(0.656)*	-10.6 (0.642)	-10.2 (0.600)	-13.3 (0.498)	-2.8 (0.734)	-1.9 (0.794)
**Al**	0.5	-9.6 (0.803)	-11.3 (0.786)	-10.8 (0.753)	-12.2 (0.666)	-5.5 (0.833)	-4.1 (0.882)
**Ga**	1.2	-8.9 (0.786)	-10.7 (0.773)	-10.3 (0.741)	-11.9 (0.645)	-4.6 (0.826)	-3.3 (0.880)
**Si**	-0.7	-7.5 (0.868)	-8.5 (0.859)	-7.9 (0.836)	-8.0 (0.790)	-4.6 (0.922)	-3.0 (0.950)
**Ge**	-0.4	-7.0 (0.881)	-8.2 (0.871)	-7.7 (0.849)	-7.6 (0.807)	-4.4 (0.933)	-2.9 (0.960)

Table S5*: The dimerization energies and Gibbs free energies of R_2P• type radicals for $R=EH_n$ at the (U)ωB97X-D/6-311G** level of theory in kcal·mol^{-1}*

R	ΔE_{dim}	ΔG_{dim}
F	-41.4	-27.2
Cl	-37.3	-22.7
Br	-38.0	-23.5
OH	-47.7	-31.2
SH	-42.7	-28.1
SeH	-42.5	-23.9
NH_2	-53.8	-37.4
PH_2	-51.8	-34.9
AsH_2	-51.9	-37.2
CH_3	-56.6	-40.3
SiH_3	-57.0	-41.2
GeH_3	-57.5	-41.6
BH_2	-69.9	-56.1
AlH_2	-60.8*	-67.8 [a]
GaH_2	-63.9*	-46.8 [a]

[a]: A dative bond was found between the donor and acceptor sites.

Table S6: *Dimerization energies of R^AR^DP• type (R= EH_n, EMe_n) obtained at the (U)ωB97X-D/6-311G** level of theory in kcal·mol^{-1}.*

		**Δ*E*dim**	
Acceptor center	Donor center	EH_n	EMe_n
B	O	-69.9 [a]	-54.4
	S	-52.6 [a]	-48.4
	Se	-55.0 [a]	-47.5
	N	-50.7 [a]	-49.9
	P	-56.7	-63.5
	As	-59.0	-64.8
Al	O	-95.5 [a]	-98.5 [a]
	S	-72.3 [a]	-63.0 [a]
	Se	-70.2 [a]	-60.1 [a]
	N	-78.0 [a]	-80.2 [a]
	P	-78.4 [a]	-83.2 [a]
	As	-54.3	-57.4
Ga	O	-74.9 [a]	-78.1 [a]
	S	-64.3 [a]	-48.6
	Se	-62.9 [a]	-55.8 [a]
	N	-63.9 [a]	-43.3
	P	-53.8	-75.4 [a]
	As	-58.3	-56.8
Si	O	-51.9	-54.7
	S	-46.6	-49.4
	Se	-45.9	-47.8
	N	-47.4	-49.5
	P	-51.8	-57.6
	As	-53.6	-58.9
Ge	O	-52.5	-55.2
	S	-47.0	-49.6
	Se	-46.3	-48.1
	N	-48.0	-49.9
	P	-52.0	-57.8
	As	-53.8	-59.1

[a]: A dative bond was found between the donor and acceptor sites.

Table S7: *Numbering of the radicals and ΔE_{dim}, ΔG_{dim}, ΔE_{prep}, ΔE_{int}, and ΔE_{disp} values obtained at the (U)ωB97X-D/6-311G** level of theory in kcal·mol^{-1}, for R= tBu, TMS, and TFMS and R'= Pyr^{Me}, Pyr^{TFM}, Pyr^{Ph}, Pip^{Me}, Pip^{TFM}, and Pip^{Ph} .*

**Radical**	**R'**	**R**	ΔE_{dim}	ΔG_{dim}	ΔE_{int}	ΔE_{prep}	ΔE_{disp}
**26**		Pyr^{Me}	— [a]	— [a]	— [a]	— [a]	— [a]
**27**		Pip^{Me}	— [a]	— [a]	— [a]	— [a]	— [a]
**28**		Pyr^{TFM}	— [a]	— [a]	— [a]	— [a]	— [a]
**29**	*t*Bu	Pip^{TFM}	— [a]	— [a]	— [a]	— [a]	— [a]
**30**		Pyr^{Ph}	— [a]	— [a]	— [a]	— [a]	— [a]
**31**		Pip^{Ph}	— [a]	— [a]	— [a]	— [a]	— [a]
**32**		Pyr^{Me}	-35.9	-6.9	-52.6	16.6	-34.4
**33**		Pip^{Me}	-25.0	1.9	-54.9	29.9	-36.4
**34**		Pyr^{TFM}	21.9	52.2	-34.6	56.5	-31.2
**35**	TMS	Pip^{TFM}	16.9	51.4	-40.8	57.7	-38
**36**		Pyr^{Ph}	— [a]	— [a]	— [a]	— [a]	— [a]
**37**		Pip^{Ph}	— [a]	— [a]	— [a]	— [a]	— [a]
**38**		Pyr^{Me}	— [a]	— [a]	— [a]	— [a]	— [a]
**39**		Pip^{Me}	12.1	44.4	-42.8	54.8	-37.4
**40**		Pyr^{TFM}	— [a]	— [a]	— [a]	— [a]	— [a]
**41**	PFS	Pip^{TFM}	— [a]	— [a]	— [a]	— [a]	— [a]
**42**		Pyr^{Ph}	— [a]	— [a]	— [a]	— [a]	— [a]
**43**		Pip^{Ph}	— [a]	— [a]	— [a]	— [a]	— [a]

[a]: No dimer could be optimized.

Table S8: *ΔE_{dim} values for different solvents obtained with the SMD solvation model at the (U)ωB97X-D/6-311G** level of theory in kcal·mol^{-1}*

	**Solvent**			
**Radical**	**Hexane**	**Toulene**	**THF**	**Diethyl ether**
**1**	-52.2	-51.8	-51.6	-51.3
**5**	-27.4	-27.2	-27.6	-26.5
**9**	-46.7	-46.9	-47.5	-46.5
**13**	1.4	1.7	1.9	2.3
**17**	-43.4	-43.6	-44.2	-43.1
**20**	16.2	15.8	14.3	15.6
**34**	21.6	21.4	21.2	22.2
**39**	14.3	14.6	14.7	19.3

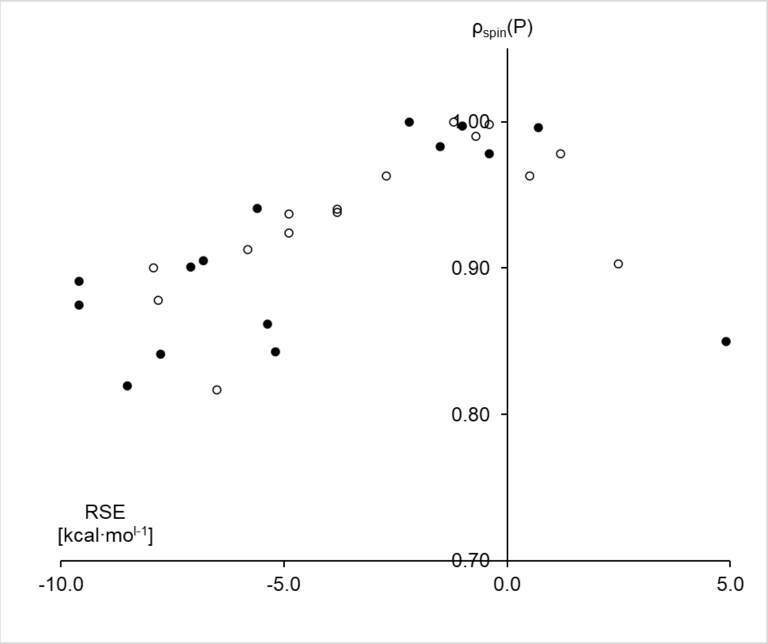

Figure S1: *Plot of RSE vs spin populations ρ_{spin}(P) calculated at the (U)DF-CCSD(T)/aug-ccpVTZ//ωB97X-D/6-311G** level for the R_2P• (filled markers) and RHP• (unfilled markers) type radicals with R=EH_n*

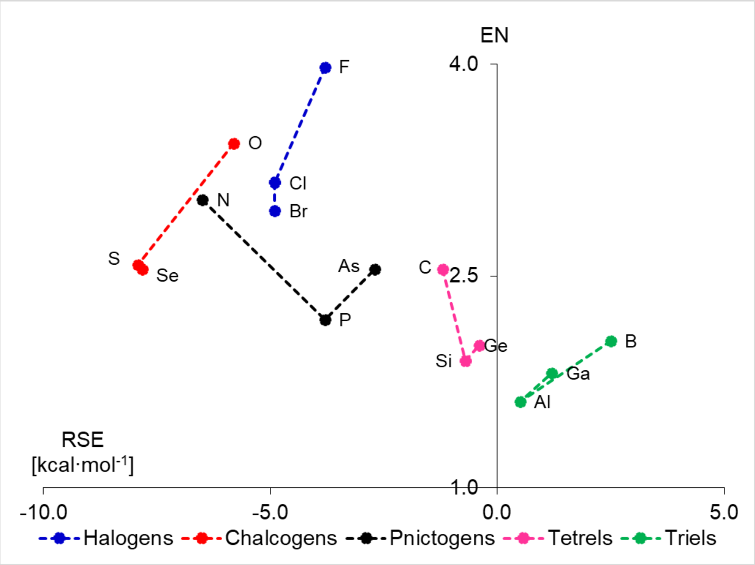

***Figure S2**: Plot of RSEs obtained at the (U)DF-CCSD(T)/aug-cc-pVTZ//(U)ωB97X-D/6-311G** level vs the Pauling electronegativity (EN) of the central atom E in the R substituent for the RHP• type radicals*

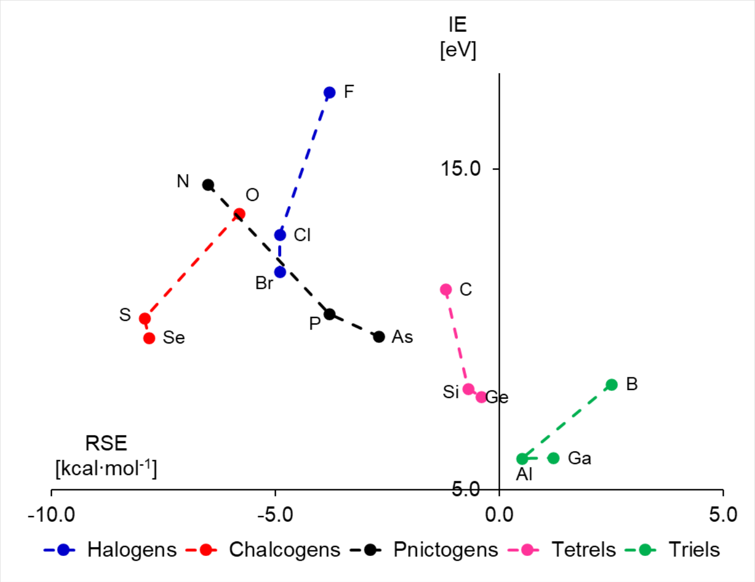

Figure S3: *Plot of RSEs obtained at the (U)DF-CCSD(T)/aug-cc-pVTZ//(U)ωB97X-D/6-311G** level vs the ionization energy (IE) of the central atom E in the R substituent for the RHP• type radicals*

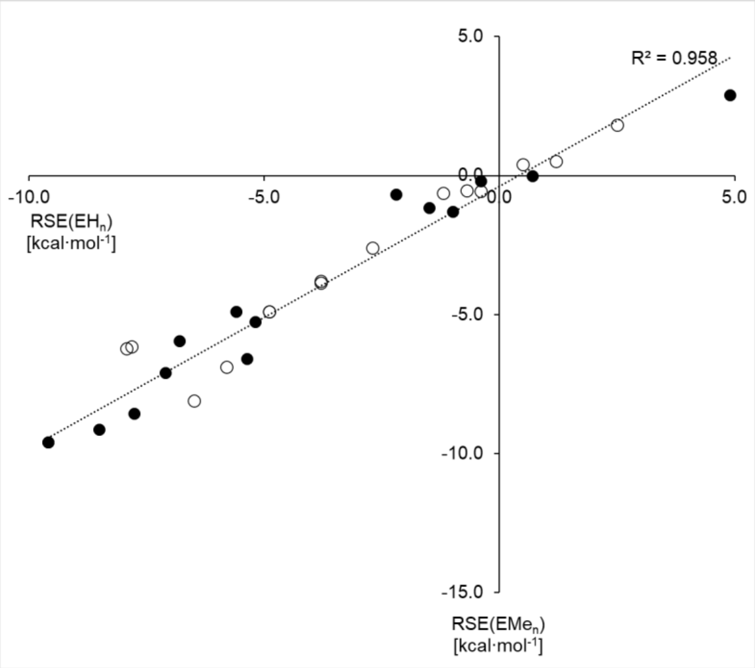

Figure S4: *Correlation between the RSE values obtained for R=EH$_n$ and R=EMe$_n$. Unfilled and filled markers correspond to the RHP• and R$_2$P• type of radicals. Energies were obtained at the (U)DF-CCSD(T)/aug-cc-pVTZ//(U)ωB97X-D/6-311G** level of theory.*

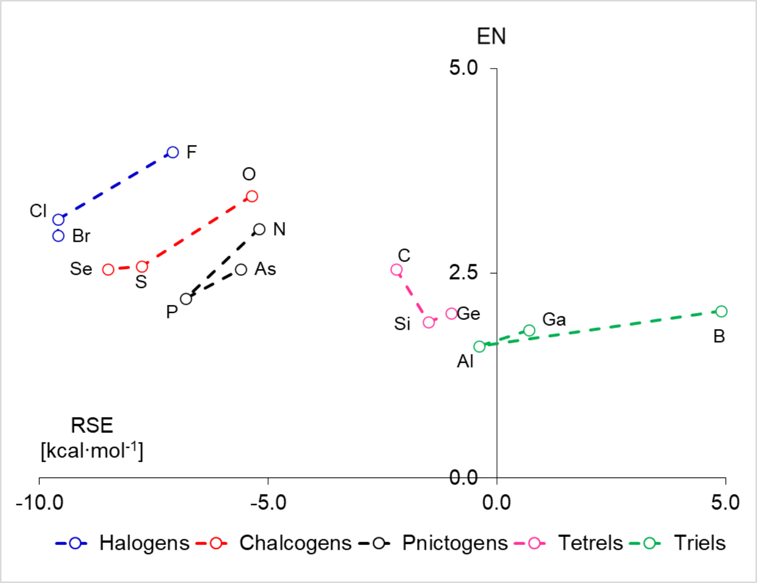

***Figure S5**: RSEs obtained at the (U)DF-CCSD(T)/aug-cc-pVTZ//(U)ωB97X-D/6-311G** level vs the Pauling electronegativity (EN) of the central atom E in the R substituent for the R_2P• type radicals*

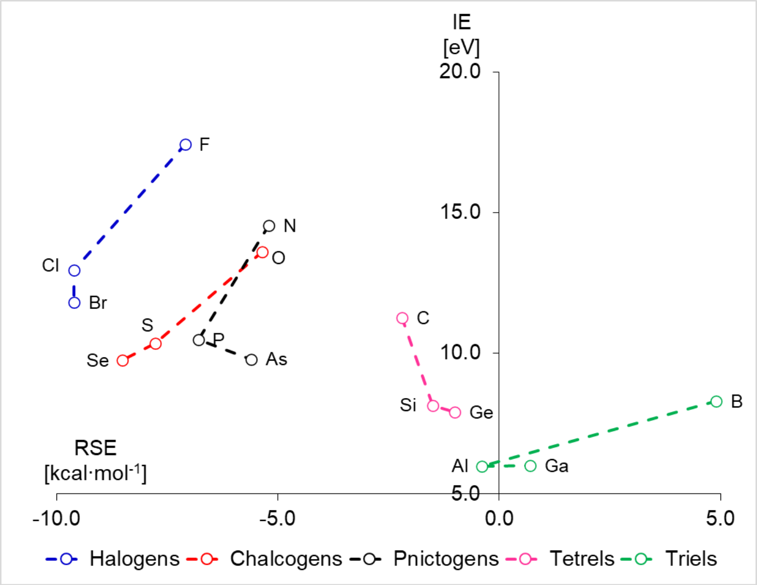

***Figure S6**: RSEs obtained at the (U)DF-CCSD(T)/aug-cc-pVTZ//(U)ωB97X-D/6-311G** level vs the ionization energy (IE) of the central atom E in the R substituent for the R_2P• type radicals*

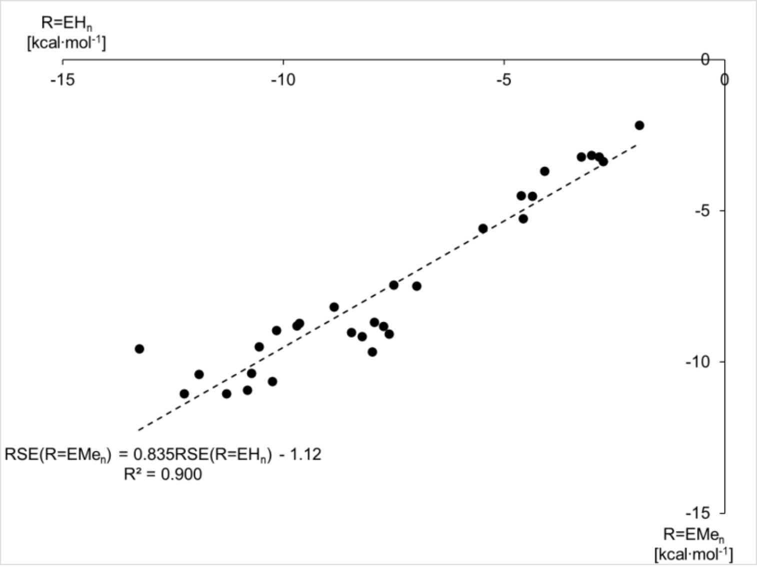

Figure S7: *Relationship between the RSEs of R^AR^DP• for EH_n and EMe_n type substitution obtained at the (U)DF-CCSD(T)/aug-cc-pVTZ//(U)ωB97X-D/6-311G** level*

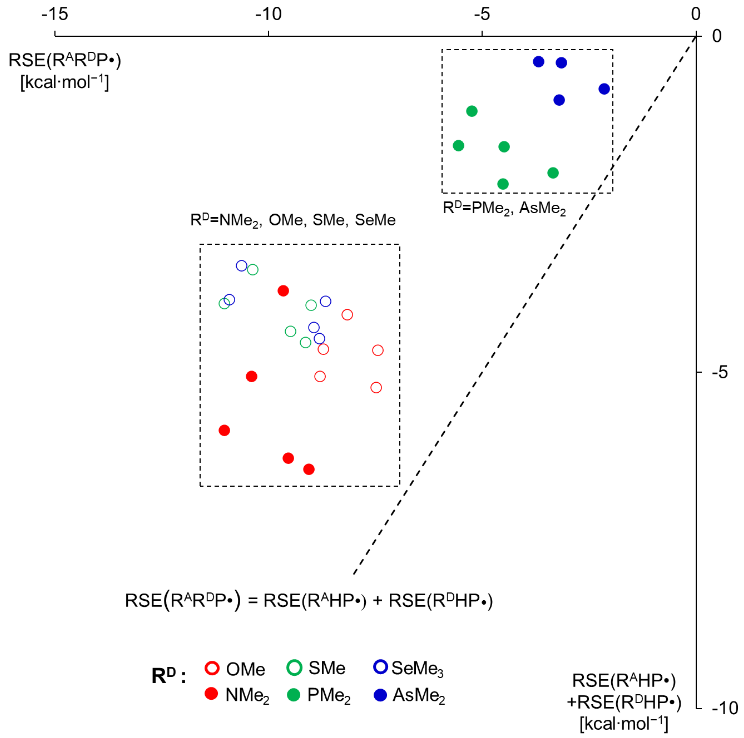

Figure S8: *Comparison of RSEs of radicals exhibiting captodative substitutions [RSE(R^AR^DP•)] with the sum of constituents, RSE(R^AHP•)+RSE(R^DHP•)*

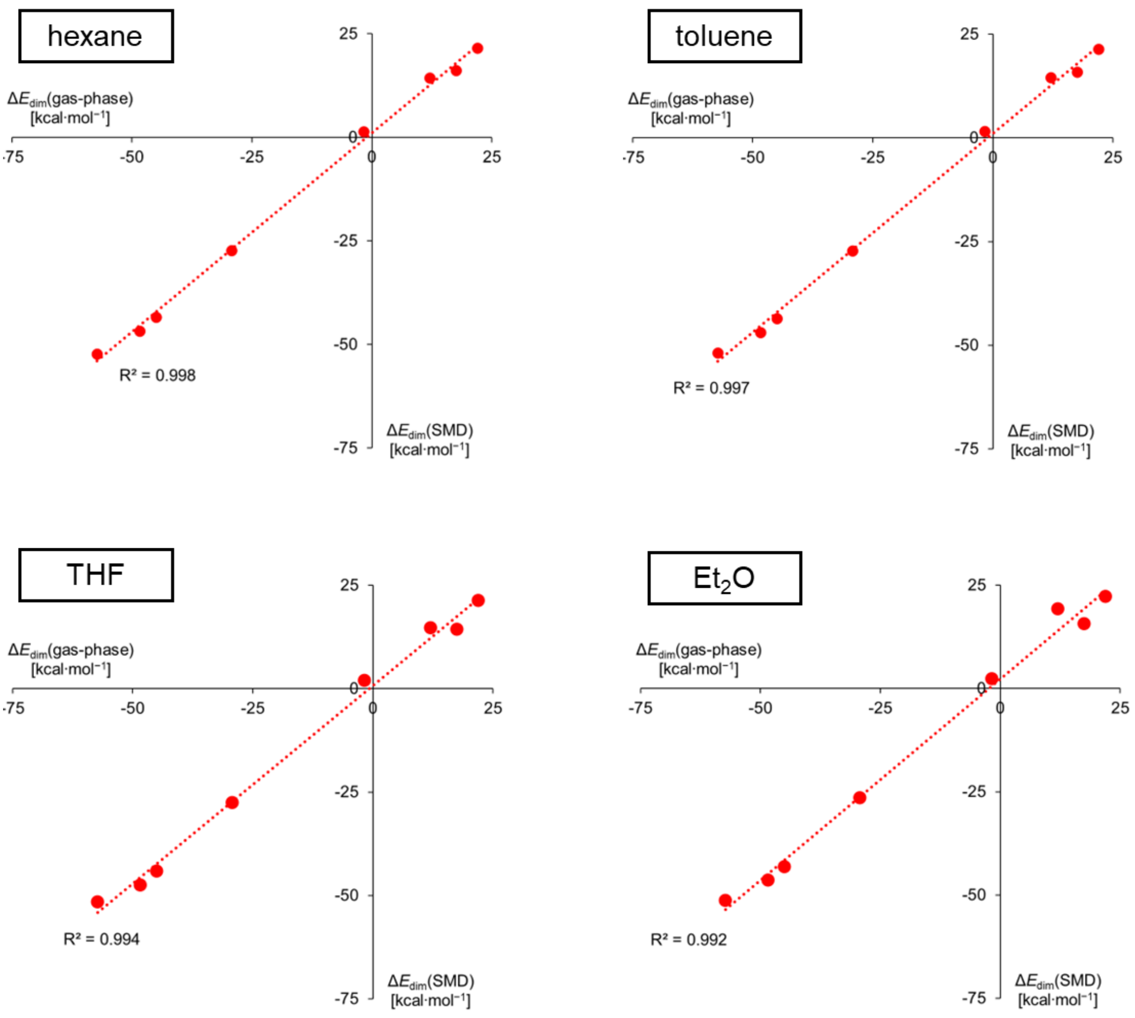

Figure S9: *Comparison of dimerization energies calculated in the gas-phase and with SMD solvent models (U)ωB97X-D/6-311G** level*

TOTAL ENERGIES AND OPTIMIZED GEOMETRIES IN CARTESIAN COORDINATES

Total energies and optimized geometries of all studied radicals, their corresponding phosphines, and dimers are provided in Cartesian coordinates.

1) RHP• type radicals and their corresponding phosphines for R=EHn

FHP•

3

E= -441.7633574

P	0.000000	0.000000	0.000000
F	0.000000	0.000000	1.622274
H	1.420285	0.000000	-0.159787

FPH2

4

E= -442.3965142

P	0.000000	0.000000	0.000000
F	0.000000	0.000000	1.625762
H	1.409070	0.000000	-0.203201
H	-0.072535	1.407040	-0.202785

ClHP•

3

E= -802.1363899

P	0.000000	0.000000	0.000000
Cl	0.000000	0.000000	2.062409
H	1.416877	0.000000	-0.131589

ClPH₂

4

E= -802.7681546

P	0.000000	0.000000	0.000000
Cl	0.000000	0.000000	2.088616
H	1.407524	0.000000	-0.167762
H	-0.084273	1.405728	-0.168847

BrHP•

3

E= -2916.1077531

P	0.000000	0.000000	0.000000
Br	0.000000	0.000000	2.215952
H	1.416757	0.000000	-0.122480

BrPH₂

4

E= -2916.7387349

P	0.000000	0.000000	0.000000
Br	0.000000	0.000000	2.249358
H	1.408633	0.000000	-0.158999
H	-0.084075	1.406803	-0.160179

(HO)HP•

4

E= -417.7416126

O	0.000000	0.000000	0.000000
P	0.000000	0.000000	1.661342
H	0.898624	0.000000	-0.334820
H	-1.417064	-0.001954	1.758519

(HO)PH₂

6

E= -493.6200821

O	0.000000	0.000000	0.000000
P	0.000000	0.000000	1.652472
O	1.596569	0.000000	2.079435
H	-0.131196	-1.402152	1.753181
H	-0.253516	0.860431	-0.335411
H	1.854763	0.860786	2.410407

(HS)HP•

4

E= -740.7266331

S	0.000000	0.000000	0.000000
P	0.000000	0.000000	2.108111
H	1.337457	0.000000	-0.134581
H	-1.419518	0.000223	2.144018

N	0.000000	0.000000	0.000000
P	0.000000	0.000000	1.696959
H	1.417616	0.000000	1.812263
H	-0.873811	0.083323	-0.491034
H	0.812440	0.190795	-0.560726

(HS)PH$_2$

5

E= -741.3537978

S	0.000000	0.000000	0.000000
P	0.000000	0.000000	2.139225
H	1.397459	0.000000	2.376190
H	0.887783	-0.982805	-0.238084
H	-0.141513	-1.390269	2.375913

(HSe)HP•

4

E= -2744.1071651

P	0.000000	0.000000	0.000000
Se	0.000000	0.000000	2.258504
H	1.492958	0.000000	-0.145176
H	-1.464248	0.076738	2.301202

(HSe)PH$_2$

5

E= -2744.737807

P	0.002422	0.509269	-0.075167
Se	-0.425574	-0.611480	1.863149
H	1.381769	0.189704	-0.155939
H	-1.152407	0.518633	2.447465
H	-0.382415	-0.529387	-0.960732

(H$_2$N)HP•

5

E= -397.8756537

(H$_2$N)PH$_2$

6

E= -398.5037195

N	0.000000	0.000000	0.000000
P	0.000000	0.000000	1.703600
H	1.387071	0.000000	2.045324
H	-0.270776	0.869896	-0.426968
H	0.724455	-0.469909	-0.518747
H	-0.141157	-1.392327	1.941156

(H$_2$P)HP•

5

E= -684.4687591

P	0.000000	0.000000	0.000000
P	0.000000	0.000000	2.190272
H	1.420975	0.000000	2.248810
H	-1.301431	0.523330	-0.184895
H	0.676392	1.219842	-0.255897

(H$_2$P)PH$_2$

6

E= -685.1021707

P	0.000000	0.000000	0.000000
P	0.000000	0.000000	2.243585

H 1.415001 0.000000 2.351395
H -1.414949 0.001259 -0.107732
H 0.080515 1.412695 -0.107660
H -0.079334 -1.412794 2.351272

($(H_2As)HP$•)

5
E= -2579.0196194
As 0.000000 0.000000 0.000000
P 0.000000 0.000000 2.314254
H 1.422647 0.000000 2.362815
H -1.394729 0.582799 -0.126056
H 0.681511 1.347175 -0.175618

$(H_2As)PH_2$

6
E= -2579.6542627
As 0.000000 0.000000 0.000000
P 0.000000 0.000000 2.355677
H 1.417749 0.000000 2.442763
H -1.512362 0.136066 -0.078676
H 0.180481 1.508406 -0.066992
H -0.074070 -1.415224 2.451792

$(H_3C)HP$•

6
E= -381.8233931
C 0.000000 0.000000 0.000000
P 0.000000 0.000000 1.852029
H 1.417949 0.000000 1.989286
H 0.510692 -0.883547 -0.393377
H -1.026475 0.000000 -0.369230
H 0.510692 0.883547 -0.393377

$(H_3C)PH_2$

7
E= -382.4609182
C 0.000000 0.000000 0.000000
P 0.000000 0.000000 1.860012
H 1.406502 0.000000 2.049529
H 0.676313 -0.734635 -0.437021
H -1.013762 -0.198984 -0.352765
H 0.282069 0.993897 -0.352638
H -0.116092 -1.401870 2.049401

$(H_3Si)HP$•

6
E= -633.2119586
Si 0.000000 0.000000 0.000000
P 0.000000 0.000000 2.259554
H 1.424098 0.000000 2.306871
H 0.698699 -1.205561 -0.515987
H -1.397959 0.000000 -0.491723
H 0.698699 1.205561 -0.515987

$(H_3Si)PH_2$

7
E= -633.8505366
Si 0.000000 0.000000 0.000000
P 0.000000 0.000000 2.265321

H	1.414649	0.000000	2.380508
H	0.925798	-1.001382	-0.585076
H	-1.379415	-0.289080	-0.462073
H	0.396649	1.352578	-0.461552
H	-0.111084	-1.410507	2.380849

(H_3Ge)HP•

6

E=-2420.7492232

Ge	0.000000	0.000000	0.000000
P	0.000000	0.000000	2.324057
H	1.423583	0.000000	2.371954
H	0.722529	-1.247983	-0.523781
H	-1.446052	0.000000	-0.503597
H	0.722529	1.247983	-0.523781

(H_3Ge)PH_2

7

E= -2421.3877026

Ge	0.000000	0.000000	0.000000
P	0.000000	0.000000	2.329454
H	1.415514	0.000000	2.433676
H	0.966585	-1.037443	-0.582642
H	-1.422532	-0.305406	-0.479516
H	0.405834	1.397528	-0.478747
H	-0.100920	-1.412050	2.434109

(H_2B)HP•

5

E= -367.9363757

B	0.000000	0.000000	0.000000
P	0.000000	0.000000	1.887216
H	1.416701	0.000000	2.028147
H	-0.788737	-0.654102	-0.613813
H	0.737518	0.734692	-0.589000

(H_2B)PH_2

6

E= -368.5799343

B	0.000000	0.000000	0.000000
P	0.000000	0.000000	1.865970
H	1.363323	0.000000	2.223377
H	-0.870752	-0.566835	-0.584894
H	0.773967	0.690719	-0.587556
H	-0.356349	-1.316485	2.221280

(H_2Al)HP•

5

E= -585.5352041

Al	0.000000	0.000000	0.000000
P	0.000000	0.000000	2.336679
H	1.425187	0.000000	2.409624
H	-1.187838	-0.716239	-0.766176
H	1.147928	0.761700	-0.788031

(H_2Al)PH_2

6

E= -586.1761414

Al	0.000000	0.000000	0.000000
P	0.000000	0.000000	2.322811

H	1.409063	0.000000	2.482407
H	-1.076667	-0.870779	-0.769305
H	0.993659	0.965144	-0.768730
H	-0.167666	-1.398825	2.482680

(H_2Ga)HP•

5

E= -2267.9879126

Ga	0.000000	0.000000	0.000000
P	0.000000	0.000000	2.320827
H	1.426017	0.000000	2.341212
H	-0.780939	-1.103000	-0.786133
H	0.744741	1.149435	-0.757003

(H_2Ga)PH_2

6

E= -2268.6289308

Ga	0.000000	0.000000	0.000000
P	0.000000	0.000000	2.310750
H	1.409136	0.000000	2.465698
H	-1.060220	0.860082	-0.757342
H	0.978745	-0.951123	-0.758214
H	-0.163561	1.399492	2.465162

2) **RHP• type radicals and their corresponding phosphines R=EMe_n**

(MeO)HP•

7

E= -457.037378

P	-0.012136	0.002408	-0.010320
H	0.030772	-0.135958	1.404830
O	1.624912	0.184463	-0.146301
C	2.157171	0.370842	-1.448833
H	3.236560	0.470042	-1.340113
H	1.939406	-0.487766	-2.093474
H	1.758980	1.279145	-1.914210

(MeO)PH_2

8

E= -457.664843

C	-0.021839	-0.014730	0.008930
O	0.075225	0.368907	1.368651
P	1.526921	0.075677	2.123080
H	2.099649	1.386298	2.116581
H	-1.077199	0.030730	-0.260930
H	0.535728	0.668927	-0.643155
H	0.345394	-1.035547	-0.153961
H	1.038887	0.226427	3.445999

(MeS)HP•

7

E= -780.033877

C	-0.019579	0.071121	-0.065651
S	-0.293681	-0.057726	1.729557
P	1.558623	0.415754	2.596852
H	2.230727	0.635960	1.360594
H	-0.965196	-0.172337	-0.550127
H	0.273081	1.085372	-0.335719
H	0.743117	-0.637314	-0.387671

(MeS)PH_2

8

E= -780.663341

C	-0.108734	-0.106299	-0.177006
S	-0.254201	0.630475	1.485047
P	1.757926	0.387964	2.153327
H	1.965615	1.698808	2.652767
H	-1.100741	-0.035800	-0.624309
H	0.598540	0.445874	-0.795121
H	0.181192	-1.155160	-0.121950
H	1.483169	-0.159172	3.432440

(MeSe)HP•

7

E= -2783.418865

C	-1.454153	1.133745	0.000047
Se	-0.383857	-0.503995	-0.000018
P	1.697771	0.294585	0.000053
H	1.286353	1.657146	-0.000655
H	-2.501032	0.830739	0.000071
H	-1.237911	1.713341	0.895472
H	-1.237900	1.713354	-0.895364

(MeSe)PH_2

8

E= -2784.047695

C	-0.118187	-0.128056	-0.211447
Se	-0.367228	0.620224	1.581968
P	1.793970	0.426542	2.252305
H	1.950345	1.732270	2.784076
H	-1.098988	-0.106710	-0.687138
H	0.577205	0.481609	-0.784966
H	0.233320	-1.156054	-0.149418
H	1.552329	-0.163136	3.519591

(Me_2N)HP•

11

E= -476.482717

C	-0.030389	0.018870	0.045258
N	0.022365	-0.022813	1.491593
C	1.363793	0.002774	2.041430
P	-1.259055	-0.469055	2.513662
H	-2.257159	-0.407474	1.501116
H	0.576408	0.851759	-0.327079
H	0.355304	-0.909577	-0.399042
H	-1.056019	0.164334	-0.293070
H	1.889099	0.908362	1.717977
H	1.323359	0.002753	3.131767
H	1.948008	-0.868927	1.716674

(Me_2N)PH_2

12

E= -477.108224

C	-0.043346	0.028988	-0.020037
N	0.083070	-0.159061	1.416980
P	1.672017	-0.051155	2.041719
C	-0.804381	-1.181515	1.937972
H	1.333932	0.002043	3.420132
H	-1.826338	-0.997207	1.591765
H	-0.511605	-2.196744	1.620168

H	-0.807321	-1.154747	3.029638
H	-1.072447	0.312293	-0.262769
H	0.615983	0.833597	-0.350430
H	0.201698	-0.878348	-0.596248
H	2.070713	-1.430885	2.121709

(Me$_2$P)HP•

11

E= -763.106644

C	-0.083720	0.109741	-0.169235
P	-0.138007	0.601395	1.617150
C	1.539132	0.068019	2.186757
P	-1.410240	-0.878155	2.558366
H	-2.463250	-0.644784	1.628703
H	0.626564	0.756804	-0.689590
H	0.213935	-0.931925	-0.307523
H	-1.067971	0.259882	-0.616601
H	2.287060	0.748959	1.774560
H	1.590827	0.133884	3.275389
H	1.781382	-0.952812	1.882309

(Me$_2$P)PH$_2$

12

E= -763.739702

C	0.016652	-0.021294	-0.022847
P	-0.019150	0.014777	1.830964
C	1.806405	0.012197	2.157337
P	-0.341532	2.214128	2.061840
H	-0.439067	2.185845	3.478471
H	2.202781	-0.976171	1.911360

H	2.344743	0.764159	1.573964
H	1.993036	0.186938	3.219347
H	0.349933	-1.010867	-0.345584
H	-0.991103	0.131206	-0.415789
H	0.682444	0.732999	-0.451030
H	-1.749502	2.161265	1.883011

(Me$_2$As)HP•

11

E= -2657.653910

C	-0.093742	0.119224	-0.232303
As	-0.172976	0.730890	1.645371
C	1.592279	0.074602	2.227465
P	-1.460102	-0.941876	2.565623
H	-2.536478	-0.675321	1.670457
H	0.628580	0.742568	-0.763483
H	0.208076	-0.926290	-0.301650
H	-1.073297	0.248423	-0.694448
H	2.361502	0.721769	1.801720
H	1.656814	0.130370	3.315205
H	1.765056	-0.953351	1.906329

(Me$_2$As)PH$_2$

12

E= -2658.288902

C	-0.027046	-0.160627	-0.317774
As	-0.104020	-0.257501	1.652248
C	1.839910	-0.120236	1.963390
P	-0.537655	2.030967	1.964662
H	-0.640975	1.941150	3.379330

H	2.304988	-1.060492	1.659292
H	2.281779	0.700800	1.396040
H	2.032457	0.028401	3.027371
H	0.373438	-1.104426	-0.694142
H	-1.033770	-0.031564	-0.719104
H	0.606994	0.660864	-0.655970
H	-1.941582	1.912098	1.778142

$(Me_3C)HP$•

15

E= -499.760491

C	-0.000621	0.000388	-0.001735
C	-0.005807	-0.002735	1.533203
C	1.438298	0.002606	2.055875
P	-0.898853	1.541707	2.115932
C	-0.737602	-1.251800	2.045968
H	-0.743904	1.262447	3.506434
H	0.514576	-0.890513	-0.376918
H	-1.016926	-0.004868	-0.406158
H	0.516114	0.878738	-0.399155
H	1.970290	-0.881255	1.684399
H	1.986380	0.887118	1.719028
H	1.469020	-0.021605	3.148651
H	-0.236673	-2.153629	1.674485
H	-0.739658	-1.294780	3.138599
H	-1.775325	-1.281610	1.701777

$(Me_3C)PH_2$

16

E= -500.399038

H	0.425100	-0.381225	0.021649
P	0.038704	0.053998	1.318669
C	1.751806	0.021698	2.100408
C	2.508310	-1.275629	1.799459
C	2.535606	1.220086	1.548289
C	1.572363	0.178526	3.616447
H	-0.372182	-1.242188	1.733413
H	3.497817	-1.254218	2.271506
H	1.972462	-2.148224	2.183051
H	2.652461	-1.413564	0.724371
H	2.551508	0.208850	4.106657
H	1.042988	1.102419	3.865789
H	1.013226	-0.659835	4.043054
H	3.526786	1.263431	2.012678
H	2.679041	1.141499	0.466273
H	2.025669	2.164986	1.756019

$(Me_3Si)HP$•

15

E= -751.196206

H	0.335047	-0.125249	-0.149675
P	-0.125461	0.271750	1.140866
Si	1.966190	0.170624	2.018026
C	1.868083	0.643234	3.832225
C	3.091250	1.365495	1.099579
C	2.637858	-1.577141	1.841716
H	2.860654	0.606345	4.292082
H	1.478025	1.656905	3.959038
H	1.216991	-0.038396	4.386498
H	3.639369	-1.639890	2.279931

H	2.002646	-2.306316	2.351434
H	2.711942	-1.868986	0.790642
H	4.098167	1.337417	1.528928
H	3.169623	1.101984	0.041357
H	2.727041	2.394042	1.166193

$(Me_3Si)PH_2$

16

E= -751.835200

H	0.283362	-0.408228	-0.067377
P	-0.165906	0.037450	1.205103
Si	1.899760	0.025023	2.157684
C	2.825820	-1.568581	1.794525
C	2.849283	1.488480	1.462924
C	1.665090	0.218712	4.010858
H	-0.513460	-1.266305	1.651338
H	3.818274	-1.552860	2.256648
H	2.286564	-2.436038	2.185055
H	2.958186	-1.711878	0.718537
H	2.635472	0.251233	4.516287
H	1.130802	1.141955	4.250603
H	1.099469	-0.617555	4.431365
H	3.842609	1.549556	1.918580
H	2.984694	1.399433	0.381315
H	2.330434	2.430214	1.661141

$(Me_3Ge)HP$•

15

E= -2538.718461

H	0.366993	-0.095806	-0.138402
P	-0.100250	0.301993	1.149361
Ge	2.050169	0.196176	2.056687
C	1.953658	0.688119	3.948940
C	3.226555	1.440321	1.100200
C	2.749595	-1.626959	1.873272
H	2.948745	0.646756	4.399250
H	1.564640	1.701867	4.065610
H	1.300309	0.003291	4.493716
H	3.751227	-1.682992	2.307967
H	2.108525	-2.344477	2.389174
H	2.812688	-1.910753	0.820688
H	4.231047	1.402188	1.530198
H	3.291926	1.172603	0.043553
H	2.856400	2.464521	1.177304

$(Me_3Ge)PH_2$

16

E= -2539.357359

H	0.234680	-0.417545	-0.096425
P	-0.232042	0.025332	1.170904
Ge	1.891388	0.026895	2.156147
C	2.855351	-1.636369	1.777577
C	2.883970	1.551773	1.432503
C	1.651514	0.232908	4.088197
H	-0.557775	-1.285660	1.612920
H	3.844457	-1.615079	2.243029
H	2.306974	-2.495593	2.169749
H	2.982637	-1.769835	0.701104
H	2.624279	0.268728	4.585283
H	1.113324	1.155610	4.314934

H	1.086993	-0.606584	4.500174
H	3.872750	1.608290	1.894900
H	3.016102	1.450991	0.352924
H	2.355850	2.486751	1.630668

(Me_2B)HP•

11

E= -446.599086

C	0.043029	-0.077904	-0.096183
B	-0.028825	0.063422	1.466457
C	1.289353	0.032915	2.322659
P	-1.754306	0.167446	2.329753
H	-1.327872	1.036127	3.376022
H	0.990697	0.275905	-0.513227
H	0.003802	-1.159502	-0.301954
H	-0.787653	0.370258	-0.646045
H	1.647010	1.074131	2.353228
H	1.169321	-0.284655	3.360875
H	2.087746	-0.550381	1.853102

(Me_2B)PH_2

12

E= -447.241814

C	-0.039954	0.039133	0.060312
B	0.026883	-0.037576	1.628755
C	1.418380	-0.003093	2.358477
P	-1.573685	-0.333138	2.634594
H	-2.498776	0.438438	1.892626
H	-1.380779	0.570874	3.705255
H	0.847484	-0.379399	-0.424853

H	-0.935239	-0.397393	-0.389289
H	-0.052701	1.109285	-0.195217
H	2.095330	0.718698	1.888294
H	1.388681	0.186389	3.433567
H	1.888631	-0.985985	2.210823

(Me_2Al)HP•

11

E= -664.193863

C	-0.012962	-0.028187	0.000771
Al	0.009763	-0.012517	1.957441
P	2.088238	0.125645	3.044999
C	-1.647789	-0.049730	2.999408
H	1.650079	-0.719675	4.108113
H	-0.992940	0.236708	-0.408136
H	0.728955	0.655115	-0.425363
H	0.235011	-1.026111	-0.381479
H	-2.325370	-0.828407	2.630397
H	-1.493659	-0.216115	4.068724
H	-2.186937	0.899572	2.890963

(Me_2Al)PH_2

12

E= -664.834400

C	-0.100835	0.029965	-0.318176
Al	0.009670	-0.050412	1.634509
C	1.724180	-0.045284	2.578202
P	-1.968211	-0.297928	2.857024
H	-2.735130	0.610510	2.080697
H	-1.631426	0.638835	3.869375

H	0.817200	-0.316869	-0.802827
H	-0.935393	-0.557212	-0.714336
H	-0.263791	1.063201	-0.649384
H	2.350244	0.794262	2.254675
H	1.625666	0.012148	3.665591
H	2.292082	-0.954983	2.347993

(Me_2Ga)HP•

11

E= -2346.637026

C	0.036768	-0.105044	-0.265277
Ga	0.021069	-0.020920	1.703834
C	1.679465	0.102005	2.763918
P	-2.036730	-0.112662	2.813229
H	-1.699340	1.028663	3.602167
H	1.049946	-0.089222	-0.672860
H	-0.461173	-1.018224	-0.606332
H	-0.521658	0.735184	-0.688850
H	2.079427	1.119149	2.684059
H	1.523662	-0.115827	3.821885
H	2.443142	-0.575341	2.371000

(Me_2Ga)PH_2

12

E= -2347.277603

C	0.004888	-0.010268	-0.009554
Ga	0.002068	-0.011059	1.960244
P	2.040086	0.078344	3.086294
C	-1.651052	0.011250	3.032589
H	2.696912	-0.868734	2.257642
H	1.689014	-0.852460	4.098665
H	-0.749835	0.675625	-0.404659
H	0.977507	0.257747	-0.426945
H	-0.250000	-1.012234	-0.372289
H	-2.548876	0.043747	2.411488
H	-1.703204	-0.877520	3.668973
H	-1.660403	0.879304	3.699071

3) **R_2P• type radicals and their corresponding phosphines R=EH_n**

F_2P•

3

E= -541.0436731

P	0.000000	0.000000	0.000000
F	0.000000	0.000000	1.603981
F	1.584097	0.000000	-0.251775

F_2PH

4

E= -541.670445

P	0.000000	0.000000	0.000000
F	0.000000	0.000000	1.606521
F	1.584978	0.000000	-0.264865
H	-0.166392	-1.412384	-0.141599

Cl_2P•

3

E= -1261.7711374

P 0.000000 0.000000 0.000000
Cl 0.000000 0.000000 2.055240
Cl 2.006546 0.000000 -0.444730

Cl_2PH

4

E= -1262.3948833

P 0.000000 0.000000 0.000000
Cl 0.000000 0.000000 2.073871
Cl 2.021230 0.000000 -0.464297
H -0.154975 1.402174 -0.123397

Br_2P•

3

E= -5489.7124541

P 0.000000 0.000000 0.000000
Br 0.000000 0.000000 2.215995
Br 2.153413 0.000000 -0.522915

Br_2PH

4

E= -5490.3349149

P 0.000000 0.000000 0.000000
Br 0.000000 0.000000 2.239912
Br 2.173408 0.000000 -0.542294
H -0.146643 1.404169 -0.114782

$(HO)_2P$•

5

E= -492.9913651

O 0.000000 0.000000 0.000000
P 0.000000 0.000000 1.648031
O 1.608480 0.000000 2.007009
H 0.480807 -0.727413 -0.404841
H 2.107987 0.728548 1.627663

$(HO)_2PH$

6

E= -493.6200821

O 0.000000 0.000000 0.000000
P 0.000000 0.000000 1.652472
O 1.596569 0.000000 2.079435
H -0.131196 -1.402152 1.753181
H -0.253516 0.860431 -0.335411
H 1.854763 0.860786 2.410407

$(HS)_2P$•

5

E= -1138.9407738

S 0.000000 0.000000 0.000000
P 0.000000 0.000000 2.122203
S 2.113549 0.000000 2.313658
H 2.070426 0.000000 3.656751
H -1.341507 0.000000 -0.078220

$(HS)_2PH$

6

E= -1139.5656726

S 0.000000 0.000000 0.000000
P 0.000000 0.000000 2.133906

S	2.030749	0.000000	2.789429
H	2.459084	1.143225	2.221931
H	0.671723	1.143186	-0.233272
H	-0.264268	1.375297	2.326392

(HSe)$_2$P•

5

E= -5145.706298

P	0.000000	0.000000	0.000000
Se	0.000000	0.000000	2.258505
Se	2.247901	0.000000	-0.218587
H	-1.466258	0.000000	2.301203
H	2.148489	0.000000	-1.682094

(HSe)$_2$PH

6

E= -5146.332571

P	-0.176346	0.332188	-0.228535
Se	-0.437536	0.472482	2.032795
Se	2.063207	0.424961	-0.650133
H	2.119067	-0.988332	-1.035512
H	-0.844313	-0.931679	2.141598
H	-0.352248	1.723174	-0.417604

(H$_2$N)$_2$P•

7

E= -453.2451577

N	0.000000	0.000000	0.000000
P	0.000000	0.000000	1.708114
N	1.700624	0.000000	1.868361
H	-0.892397	-0.161613	-0.441175
H	0.526945	0.729500	-0.462849
H	2.056047	0.161966	2.798198
H	2.210747	-0.730021	1.387804

(H$_2$N)$_2$PH

8

E= -453.877586

N	0.000000	0.000000	0.000000
P	0.000000	0.000000	1.703635
N	1.585895	0.000000	2.325907
H	-0.088039	-0.897083	-0.449477
H	0.567076	0.641911	-0.533925
H	2.289842	0.642338	1.993340
H	1.972524	-0.897230	2.570995
H	-0.200332	1.393480	1.840208

(H$_2$P)$_2$P•

7

E= -1026.431205

P	-0.001936	0.000418	-0.001868
P	0.002352	0.015115	2.197144
P	2.186546	0.000427	2.451975
H	2.167629	-0.400550	3.809951
H	2.484033	-1.295383	1.949014
H	-1.353205	-0.400619	-0.137802
H	0.531700	-1.295374	-0.240078

(H$_2$P)$_2$PH

8

E= -1027.0604719

P	0.000000	0.000000	0.000000
P	0.000000	0.000000	2.224152
P	2.202446	0.000000	-0.310592
H	-0.293348	1.362312	-0.254932
H	0.306360	1.363976	2.468638
H	-1.399181	0.187088	2.342442
H	2.124168	0.186992	-1.712606
H	2.487256	1.363989	-0.041438

$(H_2As)_2P\bullet$

7

E= -4815.5338275

As	0.000000	0.000000	0.000000
P	0.000000	0.000000	2.322620
As	2.309082	0.000000	2.566741
H	2.259847	0.552180	3.979041
H	2.559077	1.368910	1.950958
H	-1.455283	0.418093	-0.097810
H	0.506740	1.424429	-0.171749

$(H_2As)_2PH$

8

E= -4816.1640232

P	0.000000	0.000000	0.000000
As	0.000000	0.000000	2.345584
As	2.328812	0.000000	-0.280003
H	-0.239987	-1.378901	-0.212849
H	2.574379	-1.446724	0.118172
H	2.232588	-0.351482	-1.753965
H	-1.474903	-0.351507	2.426001
H	0.424667	-1.446717	2.541862

$(H_3C)_2P\bullet$

9

E= -421.1435529

C	0.000000	0.000000	0.000000
P	0.000000	0.000000	1.848907
C	1.830225	0.000000	2.111082
H	0.512080	-0.884737	-0.392997
H	-1.024907	0.000054	-0.375002
H	0.512159	0.884694	-0.392998
H	2.056107	0.000089	3.178807
H	2.291842	-0.884772	1.659941
H	2.291899	0.884659	1.659786

$(H_3C)_2PH$

10

E= -421.7789644

C	0.000000	0.000000	0.000000
P	0.000000	0.000000	1.854719
C	1.828067	0.000000	2.167853
H	0.681799	-0.739764	-0.425606
H	-1.009771	-0.197524	-0.365933
H	0.291131	0.991223	-0.355440
H	2.018350	-0.196740	3.225038
H	2.362679	-0.740151	1.568185
H	2.227453	0.991100	1.940148
H	-0.206776	-1.394054	2.028909

$(H_3Si)_2P$•

9

E= -923.9210751

Si	0.000000	0.000000	0.000000
P	0.000000	0.000000	2.256402
Si	2.248859	0.000000	2.439909
H	0.963836	-0.996723	-0.536599
H	-1.359076	-0.316142	-0.500216
H	0.400709	1.345782	-0.486595
H	2.636940	0.314998	3.835413
H	2.766916	-1.345292	2.078979
H	2.861804	0.997724	1.523806

$(H_3Si)_2PH$

10

E= -924.558012

Si	0.000000	0.000000	0.000000
P	0.000000	0.000000	2.259804
Si	2.239735	0.000000	2.560320
H	2.528701	0.266056	3.990204
H	2.938185	1.011458	1.726146
H	-1.378756	0.266056	-0.476549
H	0.919640	1.011451	-0.581340
H	0.426552	-1.348254	-0.448450
H	2.740926	-1.348254	2.197192
H	-0.157881	1.403344	2.397923

$(H_3Ge)_2P$•

9

E= -4498.9951557

Ge	0.000000	0.000000	0.000000
P	0.000000	0.000000	2.324842
Ge	2.315441	0.000000	2.533689
H	1.006985	-1.022428	-0.545756
H	-1.404055	-0.343388	-0.505901
H	0.397765	1.396088	-0.497718
H	2.693168	0.343413	3.977510
H	2.846929	-1.396070	2.182254
H	2.949403	1.022442	1.579785

$(H_3Ge)_2PH$

10

E= -4499.6319022

Ge	0.000000	0.000000	0.000000
P	0.000000	0.000000	2.327998
Ge	2.308398	0.000000	2.629445
H	2.604222	0.309534	4.100260
H	3.010422	1.031899	1.737327
H	-1.420131	0.309509	-0.483786
H	0.975512	1.031899	-0.580596
H	0.408095	-1.399838	-0.470760
H	2.828035	-1.399844	2.285768
H	-0.126656	1.410260	2.439186

$(H_2B)_2P$•

7

E= -393.3789834

B	0.000000	0.000000	0.000000
P	0.000000	0.000000	1.867320
B	1.862153	0.000000	2.005539

H -0.563192 -0.851374 -0.617072

H 0.534094 0.907436 -0.565532

H 2.465288 0.907353 1.514364

H 2.436337 -0.851058 2.613074

$(H_2B)_2PH$

8

E= -394.0256451

B 0.000000 0.000000 0.000000

P 0.000000 0.000000 1.833002

B 1.539336 0.000000 2.828233

H 1.021421 -0.291326 -0.537161

H -0.992629 0.249884 -0.606199

H 1.508988 0.249910 3.990936

H 2.545229 -0.291180 2.262472

H -1.159362 0.468248 2.464216

$(H_2Al)_2P\bullet$

7

E= -828.5791707

Al 0.000000 0.000000 0.000000

P 0.000000 0.000000 2.291634

Al 2.262025 0.000000 2.658731

H -0.041917 -1.385406 -0.764820

H -0.042543 1.385330 -0.764725

H 3.010305 1.385367 2.821896

H 3.009965 -1.385373 2.823827

$(H_2Al)_2PH$

8

E= -829.218801

Al -0.049909 0.017068 -0.049962

P -0.216501 -0.636259 2.133369

Al 1.707803 0.017289 3.177976

H 1.235242 -0.437397 -0.850757

H -1.243253 0.815773 -0.712061

H 1.616553 0.816364 4.539428

H 3.077784 -0.437414 2.533111

H -1.197934 0.230111 2.667689

$(H_2Ga)_2P\bullet$

7

E= -4193.482415

Ga 0.223103 -0.047196 0.476609

P 0.231874 -1.243567 2.432807

Ga 1.876422 -0.045289 3.490116

H 1.125754 -0.527485 -0.702063

H -0.765602 1.151555 0.333569

H 1.465188 1.154241 4.399518

H 3.355745 -0.524971 3.362756

$(H_2Ga)_2PH$

8

E= -4194.121902

Ga 0.141730 0.020701 0.416340

P -0.091144 -0.718550 2.565863

Ga 1.840996 0.022635 3.534715

H 1.396367 -0.450036 -0.378200

H -0.997574 0.879820 -0.210169

H 1.749362 0.883198 4.830732

H	3.189058	-0.448403	2.911921
H	-1.039353	0.196172	3.081999
H	0.814450	0.888022	-0.572526
H	0.774699	-0.829421	-0.089973
H	3.276378	1.820735	0.780925
H	4.222576	1.920596	2.281170
H	3.825679	0.327603	1.588905
H	0.483711	0.488791	3.541034

4) **R_2P• type radicals and their corresponding phosphines R=EMe_n**

$(MeO)_2P$•

11

E= -571.582003

C	0.081851	0.056431	0.039567
O	0.002459	-0.023874	1.460444
P	1.315847	0.036693	2.415569
O	2.072398	1.340347	1.729269
C	3.380530	1.664398	2.167595
H	-0.928802	-0.085493	-0.341010
H	0.461084	1.032367	-0.268413
H	0.735170	-0.728144	-0.357691
H	3.687742	2.559874	1.628620
H	3.403013	1.875801	3.243032
H	4.087469	0.856242	1.948156

$(MeO)_2PH$

12

E= -572.208910

C	0.254078	0.133247	-0.010902
O	0.095832	0.531608	1.340596
P	1.251857	0.079277	2.426776
O	2.282910	1.361518	2.543215
C	3.459426	1.349001	1.752041
H	-0.740174	0.037303	-0.448356

$(MeS)_2P$•

11

E= -1217.560424

C	-0.154672	-0.127793	-0.189378
S	-0.548351	-0.127588	1.597083
P	1.329622	0.037966	2.534051
S	2.010502	1.868468	1.723863
C	3.731111	1.824029	2.324099
H	-1.095837	-0.317956	-0.705318
H	0.243165	0.835792	-0.502486
H	0.554056	-0.921255	-0.424089
H	4.182357	2.772418	2.034381
H	3.762019	1.733636	3.409971
H	4.284789	1.006926	1.862961

$(MeS)_2PH$

12

E= -1218.186935

C	0.438270	-0.128866	0.038153
S	0.055448	0.644045	1.643880
P	1.890437	0.254032	2.654038
S	3.046640	2.043664	2.669506
C	4.221801	1.641352	1.335547

H	-0.497342	-0.161645	-0.520130
H	1.160679	0.462987	-0.523666
H	0.807693	-1.145824	0.171345
H	4.995637	2.408505	1.363401
H	4.682168	0.666972	1.500048
H	3.737106	1.668228	0.359763
H	1.345854	0.475927	3.939474

$(MeSe)_2P$•

11

E= -5224.329301

C	-0.073508	-0.124911	-0.181604
Se	-0.504309	-0.085914	1.735544
P	1.559913	0.076414	2.618913
Se	2.132767	2.103254	1.856588
C	4.030148	2.017543	2.337749
H	-1.024231	-0.233741	-0.703806
H	0.408230	0.804350	-0.477677
H	0.567086	-0.977514	-0.397556
H	4.452933	2.996136	2.112802
H	4.136241	1.811129	3.401473
H	4.532290	1.254558	1.746377

$(MeSe)_2PH$

12

E= -5224.953686

C	0.446356	-0.099007	0.017868
Se	0.035179	0.635689	1.786188
P	2.070087	0.221032	2.706671
Se	3.216802	2.178059	2.829849

C	4.409372	1.823037	1.317401
H	-0.494571	-0.128638	-0.531348
H	1.149737	0.544232	-0.507896
H	0.843794	-1.108056	0.112981
H	5.159682	2.613521	1.322729
H	4.897236	0.857772	1.441648
H	3.856851	1.857754	0.380144
H	1.596052	0.297779	4.038507

$(Me_2N)_2P$•

19

E= -610.456028

C	-0.000745	0.003836	-0.000828
N	-0.000513	-0.002024	1.448007
C	1.325692	-0.006326	2.039127
P	-1.345625	0.675944	2.251908
N	-1.199040	-0.147545	3.740398
C	-1.084587	-1.593316	3.810443
C	-1.802336	0.454072	4.912255
H	1.866292	-0.916612	1.754151
H	1.917939	0.860771	1.710453
H	1.241875	0.019223	3.125700
H	0.500016	-0.894094	-0.381562
H	-1.026705	0.000612	-0.374640
H	0.518424	0.881181	-0.414932
H	-0.432546	-1.883459	4.642754
H	-2.064790	-2.069919	3.961078
H	-0.650796	-1.971132	2.884485
H	-1.196999	0.238683	5.800609
H	-1.850492	1.538428	4.793122

H	-2.819725	0.078202	5.097715

$(Me_2N)_2PH$

20

E= -611.086507

C	0.003218	0.010803	-0.003545
N	0.004295	-0.003041	1.447954
C	1.335726	0.005814	2.021386
P	-1.356251	-0.669349	2.238698
N	-1.110753	-2.306287	2.663539
C	-1.610627	-3.322749	1.755810
C	-0.003826	-2.761158	3.481718
H	-1.012458	-0.160753	3.517723
H	-0.286974	-3.671684	4.022497
H	0.896330	-2.988756	2.889884
H	0.257025	-1.999750	4.219699
H	-1.941847	-4.202390	2.319918
H	-2.466195	-2.933974	1.200203
H	-0.850206	-3.655607	1.032034
H	1.905694	0.857453	1.632131
H	1.277735	0.108631	3.106959
H	1.902735	-0.909920	1.792736
H	0.528121	0.899544	-0.372746
H	0.496588	-0.873667	-0.436195
H	-1.023744	0.045554	-0.372305

$(Me_2P)_2P$•

19

E= -1183.706408

C	0.112129	-0.171277	-0.150583
P	0.012258	0.155342	1.668459
P	2.104696	-0.027071	2.220625
P	1.871091	-0.509342	4.361200
C	3.591876	-0.104730	4.914847
C	-0.840543	-1.384258	2.247727
C	2.011806	-2.363243	4.307562
H	-1.864421	-1.371188	1.866264
H	-0.344432	-2.294769	1.903311
H	-0.891260	-1.386557	3.338265
H	-0.889285	-0.084045	-0.578545
H	0.745746	0.583371	-0.620610
H	0.512437	-1.161317	-0.380168
H	2.108321	-2.729209	5.333333
H	1.104993	-2.794958	3.881416
H	2.872024	-2.705438	3.727313
H	3.711784	-0.433381	5.950493
H	4.360610	-0.581734	4.301376
H	3.737835	0.976475	4.884974

$(Me_2P)_2PH$

20

E= -1184.336249

C	-0.120387	-0.098346	0.055260
P	-0.020030	-0.070242	1.907548
C	1.822627	0.041227	2.091372
P	-0.469465	2.101842	2.194750
P	0.128058	2.307817	4.338053
C	1.692936	3.267382	4.074554
C	-1.005631	3.711057	4.765371
H	2.259897	-0.898866	1.746123
H	2.263195	0.862584	1.518257

H	2.081702	0.154926	3.146522
H	0.229341	-1.071902	-0.298540
H	-1.160601	0.013422	-0.258928
H	0.475472	0.687987	-0.415188
H	2.065395	3.604746	5.045322
H	2.454145	2.619412	3.634665
H	1.552144	4.134153	3.423882
H	-0.701926	4.118506	5.732950
H	-0.987655	4.515514	4.024971
H	-2.028021	3.341014	4.864412
H	-1.846000	1.917118	2.492609

$(Me_2As)_2P$•

19

E= -4972.803566

C	-0.059457	0.076049	-0.263585
As	0.156408	-0.819097	1.479519
C	1.859102	0.040204	2.001330
P	-1.316240	0.530101	2.659907
As	-0.730172	-0.192752	4.785083
C	-1.464009	-2.027252	4.707955
C	-2.238263	0.647363	5.737011
H	2.653459	-0.374284	1.376824
H	1.816393	1.121690	1.866440
H	2.074718	-0.187246	3.045863
H	0.673434	-0.339352	-0.958648
H	-1.059125	-0.123650	-0.651985
H	0.088900	1.153655	-0.181353
H	-1.426463	-2.450078	5.714245
H	-2.494427	-2.028602	4.350371
H	-0.848672	-2.636209	4.044776
H	-2.222824	0.309590	6.775505
H	-2.117222	1.731429	5.722153
H	-3.194778	0.383175	5.283919

$(Me_2As)_2PH$

20

E= -4973.435656

C	-0.086062	0.077386	0.087138
As	-0.104970	-0.136721	2.048225
P	2.228450	-0.166314	2.308946
As	2.257128	-0.386590	4.640861
C	4.197457	-0.106531	4.852042
C	-0.311720	-2.097018	2.077362
C	1.685634	1.427618	5.156935
H	-1.335475	-2.343183	1.787746
H	0.385941	-2.581881	1.392270
H	-0.140241	-2.464228	3.090733
H	-1.098407	-0.089283	-0.287783
H	0.212541	1.095644	-0.167587
H	0.597196	-0.630533	-0.384738
H	1.765059	1.530409	6.241601
H	2.302562	2.188270	4.674651
H	0.643272	1.569579	4.867167
H	4.423515	-0.040647	5.918533
H	4.735603	-0.957669	4.432846
H	4.528015	0.809480	4.358920
H	2.378245	1.249024	2.274689

$(Me_3C)_2P$•

27

E= -657.011048

C	0.073266	0.514718	-0.163384
C	0.052977	0.367950	1.362470
C	1.486766	0.413311	1.917274
P	-0.858724	-1.158188	2.002724
C	-0.340417	-2.673369	1.000093
C	1.169121	-2.808363	0.769611
C	-0.732450	1.551433	1.956989
C	-0.813855	-3.866629	1.850265
C	-1.086323	-2.712382	-0.344209
H	-0.238441	2.493768	1.694546
H	-1.755964	1.587251	1.573225
H	-0.785067	1.491647	3.047997
H	1.948096	1.374782	1.661247
H	1.495427	0.320505	3.007084
H	2.115933	-0.376879	1.505462
H	0.497557	1.489892	-0.429953
H	0.684807	-0.249761	-0.646039
H	-0.933948	0.465226	-0.586585
H	-0.889627	-3.668477	-0.844192
H	-2.167635	-2.627804	-0.202830
H	-0.769783	-1.914518	-1.017326
H	1.381321	-3.777952	0.303827
H	1.558595	-2.035250	0.104823
H	1.725922	-2.763982	1.709854
H	-0.615292	-4.803542	1.317648
H	-0.293268	-3.906465	2.811273
H	-1.887745	-3.815537	2.052229

$(Me_3C)_2PH$

28

E= -657.648982

C	0.150115	-0.033019	-0.402281
C	0.186017	-0.196739	1.127148
C	1.654644	-0.237118	1.575969
P	-0.606917	1.387922	1.784599
C	-1.246971	1.147200	3.549965
C	-0.116569	0.675995	4.472314
C	-1.683753	2.557579	3.985022
C	-2.454476	0.210305	3.674977
C	-0.504785	-1.511373	1.497421
H	-1.863103	1.219579	1.140902
H	0.701822	-0.853910	-0.873494
H	-0.874456	-0.054852	-0.784867
H	0.612541	0.906357	-0.719358
H	2.163308	-1.084314	1.102192
H	2.181952	0.676676	1.289069
H	1.751999	-0.356811	2.657239
H	-0.024613	-2.341332	0.965437
H	-0.431405	-1.726207	2.565689
H	-1.562172	-1.503968	1.219849
H	-2.807339	0.199111	4.713302
H	-3.283079	0.548629	3.046655
H	-2.218072	-0.816397	3.396540
H	-2.092349	2.522479	5.001357
H	-0.844796	3.257934	3.976551
H	-2.462668	2.957991	3.328187
H	-0.444247	0.734899	5.516179
H	0.165478	-0.361170	4.275729

H	0.776901	1.299026	4.368493

$(Me_3Si)_2P•$

27

E= -1159.891157

C	0.015259	-0.001309	0.000788
Si	0.002053	-0.000268	1.883832
C	1.772696	-0.000907	2.522603
P	-1.116662	-1.752161	2.783291
Si	-0.401558	-3.483603	1.510337
C	1.452423	-3.461618	1.181244
C	-0.864108	1.559511	2.472787
C	-0.822050	-5.057727	2.445931
C	-1.328644	-3.473692	-0.127840
H	-0.341506	2.450366	2.109468
H	-1.894702	1.601705	2.109672
H	-0.893433	1.605932	3.564799
H	2.294478	0.892223	2.162929
H	1.798253	0.014263	3.615491
H	2.329889	-0.876959	2.183497
H	0.480303	0.922367	-0.359477
H	0.580178	-0.840839	-0.411068
H	-0.999066	-0.042205	-0.405700
H	-1.053388	-4.357941	-0.712341
H	-2.410106	-3.500710	0.030261
H	-1.096155	-2.588843	-0.724426
H	1.738081	-4.376926	0.652240
H	1.755674	-2.612411	0.564433
H	2.023102	-3.425584	2.113331
H	-0.543357	-5.939648	1.860029
H	-0.290614	-5.105414	3.400424
H	-1.893140	-5.116490	2.657632

$(Me_3Si)_2PH$

28

E= -1160.528870

C	-0.016200	-0.006337	0.000530
Si	0.003764	0.000707	1.879274
C	1.777144	0.006491	2.499272
P	-0.945301	1.954661	2.523559
Si	-1.624753	1.544804	4.649826
C	-0.187621	0.829070	5.629551
C	-2.120339	3.194559	5.399143
C	-3.097828	0.379297	4.725888
C	-0.867167	-1.550597	2.493171
H	-2.208040	1.724931	1.912552
H	0.493510	-0.895819	-0.383609
H	-1.039453	-0.014097	-0.385487
H	0.489054	0.874463	-0.404419
H	2.301670	-0.891349	2.157163
H	2.319773	0.877845	2.122839
H	1.826471	0.026502	3.591014
H	-0.386302	-2.438364	2.069045
H	-0.827694	-1.640685	3.581789
H	-1.917736	-1.562285	2.189838
H	-3.426766	0.254751	5.762954
H	-3.940614	0.780271	4.155525
H	-2.860998	-0.608624	4.325491
H	-2.454912	3.064315	6.433459
H	-1.280346	3.894340	5.398087

H	-2.937762	3.655655	4.837478
H	-0.451999	0.772938	6.690297
H	0.071402	-0.180084	5.298698
H	0.706055	1.453635	5.543081

$(Me_3Ge)_2P$•

27

E= -4734.936115

C	0.001307	-0.001749	-0.058225
Ge	-0.005740	0.030104	1.904943
C	1.842656	0.021813	2.565002
P	-1.174797	-1.752827	2.852297
Ge	-0.420743	-3.514285	1.521251
C	1.512965	-3.460757	1.185090
C	-0.885599	1.677459	2.497449
C	-0.841696	-5.176165	2.469908
C	-1.381965	-3.496095	-0.189899
H	-0.347631	2.549802	2.116614
H	-1.914046	1.718119	2.132401
H	-0.905836	1.730581	3.587986
H	2.366770	0.905666	2.191133
H	1.859221	0.047186	3.656488
H	2.378323	-0.867643	2.229769
H	0.466250	0.914784	-0.431559
H	0.564112	-0.853389	-0.444813
H	-1.017453	-0.052285	-0.448490
H	-1.093268	-4.370884	-0.779026
H	-2.460440	-3.533307	-0.023271
H	-1.149742	-2.598075	-0.764429
H	1.811399	-4.368781	0.654041
H	1.791587	-2.599739	0.574756
H	2.068326	-3.415365	2.124369
H	-0.548544	-6.039227	1.866078
H	-0.308719	-5.222351	3.421969
H	-1.912796	-5.241478	2.672343

$(Me_3Ge)_2PH$

28

E= -4735.573535

C	0.015712	-0.045628	-0.112527
Ge	0.016585	0.006898	1.845940
C	1.861165	0.030286	2.504073
P	-0.983631	2.022956	2.464649
Ge	-1.647034	1.568325	4.659693
C	-0.141189	0.800727	5.654074
C	-2.144609	3.275246	5.482218
C	-3.187015	0.358126	4.729391
C	-0.898194	-1.594163	2.516783
H	-2.247481	1.726432	1.883984
H	0.533042	-0.942081	-0.464572
H	-1.006333	-0.063861	-0.497410
H	0.522879	0.831051	-0.520508
H	2.389002	-0.866617	2.169595
H	2.390162	0.905707	2.121469
H	1.889478	0.059489	3.595073
H	-0.420543	-2.488122	2.106410
H	-0.852605	-1.652805	3.606172
H	-1.947263	-1.594512	2.212789
H	-3.506293	0.221197	5.766236
H	-4.023165	0.778083	4.165688

H	-2.942701	-0.618559	4.309164
H	-2.456199	3.119415	6.518585
H	-1.298298	3.965195	5.472160
H	-2.971131	3.738703	4.938978
H	-0.394308	0.740440	6.715732
H	0.097235	-0.205127	5.302171
H	0.749743	1.424196	5.549996

$(Me_2B)_2P$•

19

E= -550.701780

C	-0.011898	-0.013716	0.007264
B	-0.006916	0.004491	1.581781
C	1.364978	0.005517	2.355572
P	-1.671609	0.103545	2.526916
B	-1.545487	-1.776897	2.876681
C	-2.402667	-2.781777	2.019414
C	-0.646974	-2.324111	4.048889
H	0.905294	-0.434287	-0.417061
H	-0.878380	-0.510452	-0.436692
H	-0.059766	1.034456	-0.322395
H	1.839243	-0.973331	2.197969
H	2.050946	0.738238	1.914033
H	1.305522	0.181178	3.430703
H	-2.001776	-3.800285	2.029545
H	-3.397087	-2.829978	2.486790
H	-2.568919	-2.467272	0.985936
H	0.173098	-2.902260	3.600392
H	-0.207842	-1.570547	4.704330
H	-1.210767	-3.039711	4.659159

$(Me_2B)_2PH$

20

E= -551.345729

C	-0.001530	0.008719	0.000685
B	-0.000226	0.005357	1.576417
C	1.365992	-0.002681	2.356618
P	-1.621711	0.089470	2.539260
B	-3.007173	-0.999815	1.863018
C	-3.276118	-1.085975	0.312796
C	-3.941370	-1.776795	2.862471
H	-1.346137	-0.362326	3.845961
H	2.018522	-0.787699	1.955223
H	1.887754	0.942279	2.154248
H	1.298196	-0.126762	3.439092
H	0.944523	0.369809	-0.413653
H	-0.118754	-1.029964	-0.338870
H	-0.820590	0.571840	-0.451608
H	-4.304107	-1.384562	0.086600
H	-3.046261	-0.172393	-0.239529
H	-2.622514	-1.867118	-0.100144
H	-4.053359	-2.817303	2.533891
H	-3.630034	-1.774230	3.908802
H	-4.948667	-1.342864	2.805911

$(Me_2Al)_2P$•

19

E= -985.896197

C	-0.129177	0.065699	-0.370397
Al	0.043456	0.117197	1.581103

C	1.794986	0.258286	2.447928
P	-1.867174	0.118179	2.872611
Al	-1.922152	-2.158623	3.235428
C	-3.019754	-3.281230	2.062470
C	-0.948223	-2.917785	4.756706
H	0.789283	-0.283768	-0.853142
H	-0.954370	-0.569183	-0.706931
H	-0.331139	1.070990	-0.760010
H	2.394571	-0.637111	2.242445
H	2.364176	1.106276	2.049936
H	1.737028	0.374115	3.533216
H	-2.704095	-4.329325	2.086924
H	-4.067659	-3.256606	2.385717
H	-3.008171	-2.945597	1.021086
H	-0.175008	-3.612165	4.405369
H	-0.457350	-2.166009	5.380006
H	-1.616094	-3.502587	5.399679

$(Me_2Al)_2PH$

20

E= -986.535641

C	0.088542	0.091205	-0.353247
Al	0.170535	0.173331	1.601967
C	1.847411	-0.044327	2.588112
P	-1.787863	0.636806	2.715760
Al	-3.236933	-0.965808	1.926781
C	-3.455961	-1.094133	-0.015354
C	-4.254887	-2.084027	3.169812
H	-1.507136	0.169898	4.023537
H	2.390273	-0.935969	2.254147
H	2.514068	0.807276	2.406380
H	1.705868	-0.123214	3.669294
H	1.013488	0.441964	-0.822404
H	-0.062377	-0.942245	-0.689350
H	-0.740746	0.675684	-0.762777
H	-4.470628	-1.391211	-0.299717
H	-3.223158	-0.155086	-0.526273
H	-2.778689	-1.851114	-0.430139
H	-4.205656	-3.140723	2.883061
H	-3.913038	-2.001141	4.204935
H	-5.316678	-1.810179	3.152665

$(Me_2Ga)_2P$•

19

E= -4350.780883

C	0.065743	0.241074	-0.341279
Ga	-0.022248	0.058385	1.619729
C	1.597948	-0.055789	2.741838
P	-2.074094	0.063399	2.676387
Ga	-1.947802	-2.171015	3.240363
C	-3.138812	-3.469404	2.355861
C	-0.684293	-2.697939	4.662720
H	1.017123	-0.122916	-0.737320
H	-0.751991	-0.287803	-0.835836
H	-0.023269	1.299490	-0.608964
H	2.008883	-1.069313	2.671115
H	2.372137	0.633241	2.393123
H	1.390186	0.146933	3.793911
H	-2.753125	-4.488890	2.435620
H	-4.120784	-3.446046	2.840640

H	-3.292286	-3.227764	1.301805
H	0.188424	-3.176380	4.203849
H	-0.329865	-1.843245	5.241513
H	-1.133374	-3.426590	5.343249

$(Me_2Ga)_2PH$

20

E= -4351.420193

C	-0.030071	0.066403	-0.363268
Ga	0.023801	0.156992	1.605527
C	1.675574	-0.138156	2.637988
P	-1.919808	0.727917	2.695943
Ga	-3.289239	-0.950530	1.921319
C	-3.527688	-1.103019	-0.029873
C	-4.233331	-2.113284	3.201217
H	-1.634497	0.245819	3.998561
H	2.184554	-1.045434	2.298602
H	2.366636	0.696173	2.478176
H	1.486890	-0.222702	3.709665
H	0.912969	0.393543	-0.808857
H	-0.197536	-0.969888	-0.677003
H	-0.842741	0.666781	-0.777699
H	-4.538425	-1.428625	-0.289276
H	-3.318460	-0.161322	-0.541794
H	-2.831806	-1.850949	-0.425760
H	-4.149118	-3.162575	2.902399
H	-3.852688	-2.007429	4.218640
H	-5.300239	-1.866586	3.208882

5) **R^AR^DP• type radicals and their corresponding phosphines $R=EH_n$**

$(H_2B)(HO)P$•

6

E= -443.180504

P	0.032199	0.001270	0.151591
O	0.034557	-0.000451	1.806329
H	0.931965	-0.000413	2.146530
B	-1.807123	0.000894	-0.183427
H	-2.144966	0.001267	-1.329123
H	-2.602215	0.000421	0.704637

$(H_2B)(HO)PH$

7

E= -443.805251

P	0.049024	0.012057	0.162786
O	0.089211	0.008194	1.817366
H	0.971453	-0.061251	2.183619
B	1.220240	-1.235647	-0.558777
H	1.464654	-1.102346	-1.720295
H	1.575843	-2.209272	0.031926
H	0.687058	1.222654	-0.212916

$(H_2B)(HS)P$•

6

E= -766.164522

P	0.108147	-0.629153	0.177260
S	-0.088164	0.512722	1.921044
B	1.786500	0.015777	-0.378869

H	-1.288475	0.037642	2.300889
H	2.223426	-0.445126	-1.389197
H	2.399078	0.840271	0.228596

(H_2B)(HS)PH

7

E= -766.788421

P	0.329653	-0.956624	0.292021
S	-0.476483	-0.175354	2.094730
B	1.607262	0.211895	-0.392379
H	-0.694604	1.095695	1.713300
H	1.810897	0.174583	-1.567340
H	2.287942	0.860003	0.338866
H	-0.735318	-0.878065	-0.630636

(H_2B)(HSe)P•

6

E= -2769.547941

P	0.003388	0.000655	-0.058427
Se	0.021537	-0.000156	2.160944
H	1.484511	-0.000265	2.300973
B	-1.865501	0.000799	-0.284679
H	-2.263472	0.001396	-1.410392
H	-2.638445	0.000560	0.625978

(H_2B)(HSe)PH

7

E= -2770.171326

P	-0.048587	-0.006198	-0.078004
Se	-0.096030	0.002415	2.181140
H	1.353002	0.003761	2.419058
B	1.208899	-1.258339	-0.652868
H	1.757995	-1.059292	-1.693546
H	1.346433	-2.283242	-0.062747
H	0.535771	1.235284	-0.409323

(H_2B)(H_2N)P•

7

E= -423.321157

P	-0.160890	-0.047361	-0.011385
N	-0.274163	0.176506	1.646062
B	1.682330	-0.088218	-0.259378
H	-1.169123	0.242169	2.102743
H	0.528043	0.255607	2.253347
H	2.093996	-0.240050	-1.370203
H	2.440319	0.033481	0.661872

(H_2B)(H_2N)PH

8

E= -423.938202

P	0.206311	-0.629339	0.298398
N	-0.243230	0.074967	1.771378
B	1.740123	0.130240	-0.385520
H	-0.520033	1.042367	1.808042
H	-0.765947	-0.503813	2.407082
H	1.943085	-0.027380	-1.551888
H	2.567269	0.603726	0.330119
H	-0.798227	-0.358635	-0.665716

(H_2B)(H_2P)P•

7

E= -709.904947

P	0.427330	-1.076069	0.219411
P	0.045917	-0.189222	2.161064
B	1.762874	0.094498	-0.386490
H	-0.194207	1.177791	1.879162
H	-1.306173	-0.560661	2.344994
H	1.929384	0.198830	-1.563871
H	2.475387	0.686966	0.368788

(H_2B)(H_2P)PH

8

E= -710.540135

P	0.101335	0.542940	-0.055696
P	-0.037897	0.424100	2.136435
B	1.817596	0.025614	-0.527747
H	-1.381650	0.864462	2.232713
H	-0.368538	-0.947938	2.275776
H	1.997390	-0.487087	-1.588826
H	2.733115	0.341050	0.167721
H	-0.766216	-0.431008	-0.593069

(H_2B)(H_2As)P•

7

E= -2604.455398

As	0.016294	-0.011153	-0.004288
P	0.011943	-0.045491	2.300494
B	1.865620	0.008158	2.562750
H	-1.249327	-0.825598	-0.182156
H	0.938308	-1.183183	-0.285114
H	2.337602	-0.571026	3.495163
H	2.576150	0.658387	1.852771

(H_2B)(H_2As)PH

8

E= -2605.091467

As	0.215093	-0.380639	-0.581961
P	0.103838	-0.385869	1.737806
B	1.847942	-0.150483	2.331406
H	-1.287234	-0.497154	-0.763987
H	0.186679	1.125465	-0.770305
H	2.048184	0.443504	3.345977
H	2.735901	-0.714109	1.768021
H	-0.577965	0.792305	2.109881

(H_2Al)(HO)P•

6

E= -660.769195

P	0.366876	0.000416	0.223491
O	0.219387	-0.000173	1.890065
H	1.085525	-0.000298	2.302481
Al	-1.918896	0.000912	-0.248003
H	-2.314987	0.001860	-1.783917
H	-2.993488	0.000270	0.912421

(H_2Al)(HO)PH

7

E= -661.392603

P	-0.116219	0.305895	0.259201
O	-0.029670	0.124949	1.918870

H	0.840249	-0.083378	2.258804
Al	1.258234	-1.333721	-0.685719
H	1.512081	-1.237699	-2.246129
H	1.810103	-2.529158	0.199199
H	0.782706	1.387501	-0.000517

(H2Al)(HS)P•

6

E= -983.759411

S	-0.160102	-0.000216	-0.069017
P	-0.323878	0.000584	2.030193
Al	1.968207	-0.000015	2.442217
H	-1.477641	0.000504	-0.344678
H	2.419227	0.000873	3.957797
H	2.985300	-0.001292	1.230226

(H2Al)(HS)PH

7

E= -984.381611

S	-0.145089	-0.055746	-0.094673
P	-0.339434	-0.030780	2.041377
Al	1.839113	0.026552	2.851964
H	0.950015	-0.819066	-0.265236
H	2.062324	-0.456521	4.341647
H	2.987577	0.606622	1.934293
H	-0.625755	-1.391095	2.328242

(H2Al)(HSe)P•

6

E= -2987.143911

P	0.332040	0.000440	0.000625
Se	0.169420	-0.000125	2.224281
H	1.618304	-0.000355	2.473987
Al	-1.963392	0.000907	-0.366418
H	-2.460184	0.001893	-1.867981
H	-2.954167	0.000228	0.869903

(H2Al)(HSe)PH

7

E= -2987.766132

P	-0.300203	0.231297	0.004573
Se	-0.105759	0.145815	2.275244
H	1.341989	-0.069113	2.411559
Al	1.244643	-1.344380	-0.720618
H	1.959193	-1.076487	-2.107528
H	1.471156	-2.652990	0.134019
H	0.446464	1.400247	-0.293538

(H2Al)(H2N)P•

7

E= -640.908127

N	-0.127738	-0.000297	-0.099677
P	-0.314107	0.000210	1.582086
Al	1.916102	0.000033	2.169038
H	-0.940378	-0.000362	-0.693798
H	0.752997	-0.000603	-0.590746
H	2.352278	0.000466	3.690552
H	2.961018	-0.000544	0.966388

(H2Al)(H2N)PH

8

E= -641.527332

N	-0.117508	0.032522	-0.080789
P	-0.304742	0.020229	1.625207
Al	1.839184	0.026338	2.494867
H	0.340011	-0.736017	-0.544535
H	-0.934846	0.331525	-0.588675
H	1.987789	-0.409582	4.012279
H	3.060608	0.585363	1.656309
H	-0.716419	-1.299375	1.985834

$(H_2Al)(H_2P)P\bullet$

7

E= -927.502581

P	-0.210090	0.071279	-0.004207
P	-0.332654	-0.232833	2.133411
Al	1.941463	-0.046247	2.568823
H	0.842659	-0.766397	-0.449196
H	-1.279902	-0.735757	-0.457562
H	2.437339	-0.215110	4.059709
H	2.940768	0.219859	1.366885

$(H_2Al)(H_2P)PH$

8

E= -928.134330

P	-0.059066	-0.076972	-0.306249
Al	-0.047267	-0.023531	2.004690
P	2.097933	-0.023583	-0.816518
H	1.930048	0.144766	-2.213602
H	2.396219	1.344940	-0.587066
H	-1.252692	0.694744	2.735784
H	1.083137	-0.812553	2.779027
H	-0.510486	1.238399	-0.579706

$(H_2Al)(H_2As)P\bullet$

7

E= -2822.053003

As	-0.005327	-0.015671	-0.018112
P	-0.000900	0.110721	2.278911
Al	2.305732	0.011745	2.529424
H	-1.244249	-0.879369	-0.147493
H	0.962528	-1.161559	-0.258312
H	2.897021	-0.357952	3.949345
H	3.241694	0.312262	1.285164

$(H_2Al)(H_2As)PH$

8

E= -2822.686439

As	-0.000000	-0.000000	-0.000000
P	-0.000000	-0.000000	2.340956
Al	2.267592	-0.000000	2.793160
H	-1.459205	0.402151	-0.105842
H	0.473235	1.426337	-0.232081
H	2.791460	0.827161	4.036653
H	3.218261	-0.915321	1.919591
H	-0.267725	1.358829	2.643152

$(H_2Ga)(HO)P\bullet$

6

E= -2343.223884

P	0.349829	0.000309	0.227772
O	0.207513	-0.000066	1.890530
H	1.074517	-0.000330	2.301631
Ga	-1.914146	0.000909	-0.257552
H	-2.318412	0.001844	-1.767518
H	-2.954884	0.000322	0.901675

(H_2Ga)(HO)PH

7

E= -2343.848678

P	-0.138280	0.315855	0.254766
O	-0.018458	0.108465	1.906656
H	0.848829	-0.147862	2.220625
Ga	1.241802	-1.302003	-0.681131
H	1.505259	-1.266217	-2.219847
H	1.818620	-2.439689	0.226987
H	0.799711	1.365839	-0.004347

(H_2Ga)(HS)P•

6

E= -2666.212570

S	-0.151996	-0.000105	-0.066715
P	-0.312300	0.000446	2.031468
Ga	1.968486	-0.000023	2.441357
H	-1.470460	0.000486	-0.339206
H	2.410575	0.000819	3.936116
H	2.966808	-0.001185	1.243718

(H_2Ga)(HS)PH

7

E= -2666.835334

S	-0.120356	-0.057659	-0.086681
P	-0.349338	-0.006472	2.046739
Ga	1.823893	0.021983	2.840685
H	0.974230	-0.828124	-0.219963
H	2.068698	-0.485598	4.295018
H	2.942136	0.607827	1.926836
H	-0.610511	-1.371991	2.334979

(H_2Ga)(HSe)P•

6

E= -4669.596722

P	0.317844	0.000383	0.002253
Se	0.169580	-0.000074	2.226523
H	1.619824	-0.000350	2.469152
Ga	-1.968783	0.000901	-0.365228
H	-2.447897	0.001927	-1.848968
H	-2.948549	0.000201	0.850665

(H_2Ga)(HSe)PH

7

E= -4670.219310

P	-0.307343	0.219743	0.002109
Se	-0.101678	0.140501	2.272635
H	1.347688	-0.069613	2.389918
Ga	1.242504	-1.339045	-0.719111
H	1.958752	-1.068498	-2.079197
H	1.459306	-2.625813	0.130213
H	0.458253	1.377113	-0.292857

(H_2Ga)(H_2N)P•

7

E= -2323.362555

N -0.123833 -0.000279 -0.096594

P -0.301911 0.000174 1.583568

Ga 1.913488 0.000024 2.166525

H -0.936811 -0.000362 -0.690406

H 0.758269 -0.000581 -0.585229

H 2.345928 0.000484 3.663456

H 2.945044 -0.000557 0.982524

(H_2Ga)(H_2N)PH

8

E= -2323.981596

N -0.113533 0.042789 -0.066865

P -0.322340 0.038957 1.634815

Ga 1.814270 0.021893 2.490538

H 0.377312 -0.711322 -0.519941

H -0.928720 0.320132 -0.590247

H 1.986961 -0.398545 3.986997

H 3.015215 0.537496 1.636039

H -0.675089 -1.300398 1.989162

(H_2Ga)(H_2P)P•

7

E= -2609.955076

P -0.187784 0.059783 -0.018534

P -0.326704 -0.178338 2.129643

Ga 1.944238 -0.034020 2.561446

H 0.835818 -0.832631 -0.422502

H -1.282169 -0.728845 -0.447535

H 2.425998 -0.289249 4.021258

H 2.930186 0.298093 1.394089

(H_2Ga)(H_2P)PH

8

E= -2610.587231

P -0.079991 -0.104350 -0.317501

Ga -0.035321 -0.019034 1.983008

P 2.086506 -0.028383 -0.794224

H 1.935111 0.159345 -2.191139

H 2.366863 1.339534 -0.541435

H -1.197494 0.722863 2.711137

H 1.079359 -0.803659 2.738468

H -0.517208 1.219894 -0.571954

(H_2Ga)(H_2As)P•

7

E= -4504.505317

As 0.036767 -0.030295 -0.019583

P -0.005459 0.075638 2.281432

Ga 2.290001 0.017877 2.579431

H -1.204679 -0.886029 -0.179736

H 0.998881 -1.185096 -0.239070

H 2.845013 -0.482860 3.948435

H 3.230735 0.484708 1.420759

(H_2Ga)(H_2As)PH

8

E= -4505.139124

As	0.140174	-0.342944	-0.432300
P	-0.097162	-0.656976	1.876433
Ga	2.031636	-0.141460	2.590026
H	-1.355896	-0.305667	-0.687196
H	0.242317	1.173387	-0.445798
H	2.198242	0.627065	3.937206
H	3.247745	-0.671298	1.768845
H	-0.724063	0.550912	2.272522

$(H_3Si)(HO)P\bullet$

7

E= -708.446430

O	0.313847	-0.000000	-0.032560
H	0.043860	-0.000000	0.887710
P	1.980380	0.000000	-0.162841
Si	1.981451	-0.000000	-2.424408
H	3.398529	0.000000	-2.870208
H	1.299139	-1.210555	-2.948342
H	1.299139	1.210555	-2.948342

$(H_3Si)(HO)PH$

8

E= -709.073143

P	0.012431	0.020573	-0.069463
O	0.022720	-0.068728	1.594692
H	0.890763	-0.162705	1.990209
Si	1.610178	1.586031	-0.492193
H	1.982981	1.495643	-1.927329
H	2.818009	1.358027	0.348914
H	1.078427	2.938854	-0.197703
H	0.872885	-1.062888	-0.427068

$(H_3Si)(HS)P\bullet$

7

E= -1031.432889

P	-0.218388	-0.000000	0.096797
S	-0.168864	0.000000	2.208301
Si	2.030199	0.000000	-0.157075
H	2.292427	0.000000	-1.617016
H	2.629639	1.209229	0.462519
H	2.629639	-1.209229	0.462519
H	-1.500886	-0.000000	2.388793

$(H_3Si)(HS)PH$

8

E= -1032.058626

P	-0.228153	0.137201	-0.179451
S	0.325313	0.381748	1.879521
Si	1.812950	0.149475	-1.159393
H	1.659052	-0.312588	-2.561842
H	2.755768	-0.744322	-0.437713
H	2.327138	1.538113	-1.127509
H	1.354555	-0.483301	1.960557
H	-0.403735	-1.270526	-0.222946

$(H_3Si)(HSe)P\bullet$

7

E= -3034.816465

Se	-0.102268	0.000000	-0.023030
H	-0.047237	-0.000000	1.443567

P	2.120352	-0.000000	-0.274281
Si	2.077546	0.000000	-2.537755
H	3.490860	-0.000000	-2.987708
H	1.388547	-1.207711	-3.059893
H	1.388547	1.207711	-3.059893

(H_3Si)(HSe)PH

8

E= -3035.442606

P	-0.006257	0.015964	-0.396616
Se	-0.240977	-0.081959	1.869715
H	1.182127	-0.085126	2.238898
Si	1.620759	1.581577	-0.543447
H	2.122915	1.602834	-1.941239
H	2.744843	1.284228	0.380901
H	1.022398	2.891717	-0.198024
H	0.842586	-1.104426	-0.590128

(H_3Si)(H_2N)P•

8

E= -688.580705

P	0.268596	-0.043585	0.085259
Si	-0.041925	0.002907	2.317503
H	1.232809	0.175118	3.064784
H	-0.675965	-1.271453	2.737483
H	-0.932280	1.144500	2.674581
N	0.482534	1.629149	-0.133900
H	0.644508	1.969984	-1.067474
H	0.148882	2.335701	0.502728

(H_3Si)(H_2N)PH

9

E= -689.206836

P	-0.278293	-0.243628	0.013948
N	0.238563	-0.124555	1.646481
Si	1.714461	0.030459	-1.013196
H	-0.766343	1.063858	-0.297996
H	1.471615	0.328810	-2.448814
H	2.492104	1.147637	-0.411207
H	2.501910	-1.220508	-0.892279
H	0.651300	0.732200	1.982811
H	-0.399687	-0.516900	2.320120

(H_3Si)(H_2P)P•

8

E= -975.176436

Si	0.010566	-0.044411	0.080785
P	-0.106802	0.198076	2.323303
P	2.013200	-0.170859	2.707159
H	-1.355583	0.079542	-0.480927
H	0.886050	1.004726	-0.505888
H	0.575646	-1.373328	-0.269093
H	2.138575	0.595699	3.890386
H	2.632778	0.781655	1.856384

(H_3Si)(H_2P)PH

9

E= -975.808849

Si	-0.003514	-0.115414	0.081318
P	0.014891	0.064135	2.334078

P 2.223029 -0.044982 2.607973
H -1.369810 0.212348 -0.394857
H 0.973952 0.794980 -0.570175
H 0.332267 -1.516508 -0.267219
H 2.219863 0.305056 3.980825
H 2.593593 1.251553 2.166897
H -0.189842 1.468147 2.368219

(H_3Si)(H_2As)P•

8

E= -2869.727534

As -0.068112 -0.647511 -0.089206
P 0.030308 0.240122 2.046107
Si 2.274648 0.095972 2.243461
H 2.772629 -1.202315 1.716654
H 2.651143 0.224702 3.672408
H 2.913138 1.191480 1.467296
H -1.210021 0.237166 -0.550500
H 0.996641 0.221353 -0.740062

(H_3Si)(H_2As)PH

9

E= -2870.361840

As -0.590206 -0.343881 0.052083
P -0.087638 0.352963 2.233469
Si 2.167108 0.238949 2.183835
H 2.650213 -1.060783 1.651107
H 2.706669 0.437762 3.553421
H 2.643892 1.331763 1.300842
H -0.253917 1.010151 -0.545308
H 0.768027 -0.916818 -0.328194
H -0.282234 -0.923251 2.822861

(H_3Ge)(HO)P•

7

E= -2495.983855

O 0.303455 0.000000 -0.003865
H 0.022881 0.000000 0.913336
P 1.970617 -0.000000 -0.114906
Ge 1.994951 -0.000000 -2.440254
H 3.454703 -0.000000 -2.909897
H 1.284869 -1.250250 -2.971703
H 1.284869 1.250250 -2.971703

(H_3Ge)(HO)PH

8

E= -2496.611561

P -0.027957 -0.004357 -0.049042
O 0.032124 -0.096165 1.613073
H 0.912984 -0.183559 1.981056
Ge 1.610577 1.601973 -0.502782
H 2.032609 1.501360 -1.973131
H 2.835434 1.364267 0.397468
H 1.059707 3.002337 -0.220089
H 0.832917 -1.081049 -0.426495

(H_3Ge)(HS)P•

7

E= -2818.970264

S -0.026078 0.000000 -0.010190

P	-0.045795	0.000000	2.101104
Ge	2.272203	0.000000	2.322863
H	2.573226	0.000000	3.823556
H	2.866634	-1.251016	1.668015
H	2.866634	1.251016	1.668015
H	-1.361136	0.000000	-0.171381

$(H_3Ge)(HS)PH$

8

E= -2819.596259

S	-0.010683	0.014416	-0.012647
P	-0.048386	-0.000429	2.133693
Ge	2.238266	-0.006706	2.568407
H	2.466036	0.318034	4.048571
H	2.934081	1.040495	1.688527
H	2.776957	-1.398665	2.237963
H	0.955444	0.927157	-0.230525
H	-0.225286	1.386892	2.377350

$(H_3Ge)(HSe)P•$

7

E= -4822.353731

Se	-0.110690	0.000000	0.007263
H	-0.067657	0.000000	1.474539
P	2.114543	-0.000000	-0.228360
Ge	2.085433	-0.000000	-2.558710
H	3.546379	-0.000000	-3.016079
H	1.374169	-1.250162	-3.088822
H	1.374169	1.250162	-3.088822

$(H_3Ge)(HSe)PH$

8

E= -4822.979885

P	-0.040250	-0.010774	-0.381918
Se	-0.247067	-0.114044	1.888367
H	1.181166	-0.105106	2.236699
Ge	1.629955	1.601821	-0.555439
H	2.152653	1.610933	-1.997033
H	2.777844	1.286917	0.411791
H	1.012079	2.956195	-0.207383
H	0.822015	-1.121134	-0.575024

$(H_3Ge)(H_2N)P•$

8

E= -2476.117886

N	-0.020804	0.009663	-0.019080
P	-0.044295	-0.067633	1.680254
Ge	2.253863	-0.017211	1.995323
H	2.889508	-1.402528	1.807707
H	2.566637	0.488278	3.406963
H	2.877125	0.936027	0.958630
H	-0.902839	0.020436	-0.505383
H	0.741179	0.393939	-0.555551

$(H_3Ge)(H_2N)PH$

9

E= -2476.744119

N	-0.023207	-0.010604	-0.010505
P	-0.040728	0.002516	1.704616
Ge	2.249644	0.005401	2.092184

H	-0.217349	-1.372170	2.055326
H	2.524078	-0.382993	3.550964
H	2.958990	-0.993772	1.164049
H	2.800201	1.408547	1.822216
H	0.379740	-0.790628	-0.506703
H	-0.873730	0.309666	-0.445235

(H_3Ge)(H_2P)P•

8

E= -2762.713827

Ge	-0.004117	-0.012460	-0.021637
P	0.017165	-0.031350	2.302698
P	2.193838	0.021455	2.502138
H	-1.459113	-0.050474	-0.493184
H	0.676147	1.262857	-0.538470
H	0.750356	-1.231636	-0.565038
H	2.262981	0.658757	3.764798
H	2.521237	1.176453	1.744995

(H_3Ge)(H_2P)PH

9

E= -2763.345995

Ge	-0.006127	-0.005428	-0.020573
P	-0.001601	-0.009530	2.305071
P	2.211750	-0.000774	2.565753
H	-1.430226	0.299954	-0.492899
H	0.959149	1.045948	-0.583536
H	0.419226	-1.395657	-0.496804
H	2.196136	0.270670	3.956843
H	2.499928	1.338946	2.198535
H	-0.279656	1.376449	2.436160

(H_3Ge)(H_2As)P•

8

E= -4657.264700

Ge	-0.011388	0.005437	-0.028598
P	0.014192	-0.014365	2.296141
As	2.320090	-0.007875	2.506200
H	1.033812	0.989226	-0.571622
H	-1.405849	0.415965	-0.508901
H	0.318599	-1.400759	-0.546867
H	2.344345	-0.869183	3.754661
H	2.623850	-1.173886	1.578968

(H_3Ge)(H_2As)PH

9

E= -4657.898831

Ge	-0.015205	-0.007123	-0.027874
P	0.009501	0.000878	2.296851
As	2.318066	-0.001063	2.706824
H	0.914968	1.073166	-0.593568
H	-1.445891	0.226562	-0.528222
H	0.471834	-1.385600	-0.484706
H	2.454584	-1.482885	2.407851
H	2.801859	0.386867	1.316709
H	-0.130087	1.407668	2.423243

6) R^AR^DP• type radicals and their corresponding phosphines R=EMe$_n$

(Me2B)(MeO)P•

15

E= -561.138006

C	0.059392	-0.648668	0.012167
B	-0.107806	0.217898	1.316442
C	1.133243	0.800393	2.077189
P	-1.889517	0.491434	1.942183
O	-1.580856	1.487433	3.227695
C	-2.671224	1.947827	4.011740
H	-2.256804	2.587491	4.790680
H	-3.375805	2.526857	3.406083
H	-3.203254	1.111586	4.476618
H	0.859129	-1.388358	0.133950
H	-0.839557	-1.163013	-0.338749
H	0.394238	0.015926	-0.796451
H	1.971850	1.026449	1.410934
H	0.900563	1.669295	2.695246
H	1.486398	0.015558	2.762593

(Me2B)(MeO)PH

16

E= -561.762801

C	-0.059331	-0.207323	0.015593
O	-0.061665	-0.382987	1.419567
P	1.382551	-0.155988	2.224970
B	0.806443	0.020758	4.038649
C	1.887602	-0.174928	5.162263
C	-0.663836	0.451285	4.354213
H	-1.100983	-0.195048	-0.307976
H	0.417370	0.736542	-0.275866
H	0.456093	-1.031992	-0.491936
H	1.590641	-1.043420	5.765286
H	2.912642	-0.341313	4.820478
H	1.884210	0.676623	5.852576
H	-0.968710	0.258371	5.386381
H	-0.725728	1.538439	4.197763
H	-1.382094	0.011412	3.657279
H	1.842911	-1.498140	2.389135

(Me2B)(MeS)P•

15

E= -884.136769

C	0.124508	-0.704859	-0.023933
B	0.035187	0.169849	1.280910
C	1.345562	0.667796	1.991401
P	-1.747828	0.578302	1.864778
S	-1.352085	1.685993	3.586774
C	-3.035928	2.092028	4.149976
H	-2.932096	2.672037	5.066781
H	-3.555892	2.688234	3.400973
H	-3.598484	1.182589	4.358228
H	0.577768	-1.669927	0.242093
H	-0.820201	-0.907881	-0.533833
H	0.817914	-0.246375	-0.739585
H	2.242975	0.152795	1.636992
H	1.471507	1.735814	1.763925
H	1.307081	0.601717	3.082840

(Me$_2$B)(MeS)PH

16

E= -884.762400

C	-0.131675	-0.021363	0.022443
S	0.047797	0.895279	1.589035
P	1.973599	0.246914	2.203139
B	1.882852	0.047450	4.101973
C	3.221667	0.220509	4.907184
C	0.554885	-0.370413	4.818532
H	-1.128852	0.210459	-0.353984
H	0.608903	0.300807	-0.708905
H	-0.052176	-1.095781	0.185371
H	3.053582	0.910559	5.743543
H	3.485197	-0.739203	5.369591
H	4.085107	0.574127	4.339000
H	0.718648	-0.860145	5.782503
H	-0.002481	0.556862	5.018364
H	-0.111261	-0.980069	4.202759
H	2.728224	1.442478	2.125818

(Me$_2$B)(MeSe)P•

15

E= -2887.521384

C	0.155630	-0.759459	-0.022646
B	0.067755	0.141781	1.264612
C	1.383862	0.632194	1.976600
P	-1.713698	0.560327	1.847543
Se	-1.293417	1.803723	3.628055
C	-3.137042	2.187341	4.159931
H	-3.090366	2.811075	5.052067
H	-3.645168	2.721544	3.359507
H	-3.654367	1.256583	4.384235
H	0.731770	-1.664375	0.211550
H	-0.795478	-1.063451	-0.465795
H	0.741005	-0.234494	-0.789061
H	2.294409	0.280905	1.483373
H	1.422018	1.727689	2.017793
H	1.413076	0.296727	3.020556

(Me$_2$B)(MeSe)PH

16

E= -2888.146554

C	-0.181709	-0.049750	-0.072893
Se	-0.013582	1.016991	1.562318
P	2.000800	0.247690	2.230563
B	1.902021	0.054383	4.130105
C	3.228101	0.250548	4.952473
C	0.576741	-0.388955	4.838588
H	-1.154296	0.195631	-0.500892
H	0.604464	0.212372	-0.778087
H	-0.143859	-1.110864	0.165268
H	3.036500	0.934161	5.789163
H	3.506515	-0.705023	5.414721
H	4.090154	0.624288	4.395432
H	0.749885	-0.937379	5.769101
H	0.040484	0.532816	5.109772
H	-0.109856	-0.949829	4.199690
H	2.801652	1.411390	2.141049

(Me$_2$B)(Me$_2$N)P•

19

E= -580.581223

N	-0.005199	0.076373	-0.120078
P	0.134964	0.243932	1.557141
B	1.947344	0.032934	2.051555
C	-1.313197	-0.208328	-0.685989
C	1.017470	0.303953	-1.123823
C	2.247206	0.442138	3.549582
C	3.121066	-0.591783	1.195079
H	3.054256	1.187118	3.563256
H	1.402578	0.857007	4.106239
H	2.632876	-0.420110	4.107577
H	3.802349	-1.167621	1.830425
H	2.800404	-1.228189	0.367502
H	3.726401	0.217748	0.762937
H	0.640536	0.999502	-1.883096
H	1.906964	0.737614	-0.674837
H	1.294001	-0.630402	-1.627984
H	-1.642972	0.608862	-1.339217
H	-1.278478	-1.129388	-1.280381
H	-2.043743	-0.338777	0.111983

(Me$_2$B)(Me$_2$N)PH

20

E= -581.205249

N	0.104957	0.105642	-0.155053
P	0.131689	0.028544	1.557112
B	1.959187	0.149318	2.073996
C	-1.162292	0.486005	-0.762139
C	0.791365	-0.933379	-0.902390
C	2.386607	-0.495851	3.443046
C	2.974326	0.998881	1.231022
H	2.964331	-1.399623	3.200556
H	3.063471	0.149184	4.012971
H	1.564608	-0.809170	4.092169
H	3.084364	1.969055	1.736770
H	3.975201	0.553575	1.226633
H	2.656099	1.201661	0.206314
H	0.847079	-0.655302	-1.959935
H	1.811430	-1.054881	-0.531538
H	0.282524	-1.909853	-0.833457
H	-0.986041	0.848053	-1.780446
H	-1.878938	-0.349690	-0.819445
H	-1.621727	1.292262	-0.188860
H	-0.106250	-1.349442	1.860988

(Me$_2$B)(Me$_2$P)P•

19

E= -867.205838

P	0.008076	-0.204182	-0.043590
P	0.017490	0.082304	2.088130
B	1.898435	0.113899	2.436598
C	1.040686	-1.694264	-0.419441
C	-1.688342	-0.849423	-0.382311
C	2.383737	-0.482749	3.812432
C	2.966825	0.768555	1.480853
H	-1.798806	-1.010955	-1.457176
H	-1.887933	-1.787184	0.140826
H	-2.425518	-0.107538	-0.070773

H	0.981473	-1.906040	-1.489819
H	2.082795	-1.488237	-0.169235
H	0.707940	-2.572729	0.137711
H	3.910216	0.210265	1.490518
H	2.655682	0.941282	0.448534
H	3.203305	1.753341	1.909245
H	3.253138	0.048196	4.214502
H	1.615176	-0.551608	4.585994
H	2.721463	-1.510656	3.611997

(Me_2B)(Me_2P)PH

20

E= -867.840667

P	0.153698	-0.259013	-0.243823
P	0.106255	0.000598	1.943421
B	1.947438	0.112616	2.428217
C	0.887564	-1.951981	-0.416810
C	-1.629608	-0.674273	-0.524073
C	2.454920	-0.678242	3.689367
C	2.929444	1.064193	1.654137
H	-1.766396	-0.940581	-1.575289
H	-1.975245	-1.506321	0.095564
H	-2.245716	0.201913	-0.314069
H	0.826799	-2.267175	-1.462208
H	1.942783	-1.917454	-0.136343
H	0.382382	-2.694395	0.207454
H	3.934746	0.632783	1.592839
H	2.610090	1.373743	0.656970
H	3.037249	1.974916	2.261018
H	3.257853	-0.158757	4.221849
H	1.677736	-0.966163	4.401584
H	2.892725	-1.614080	3.310230
H	-0.212198	-1.307784	2.396359

(Me_2B)(Me_2As)P•

19

E= -2761.752928

As	0.017991	-0.141991	-0.059129
P	-0.016710	0.285374	2.201190
B	1.872201	0.162179	2.493156
C	-1.735246	-1.021401	-0.232487
C	1.146588	-1.759222	-0.178599
C	2.393861	-0.785847	3.637870
C	2.915769	1.003932	1.665024
H	-1.855036	-1.353537	-1.265855
H	-1.820338	-1.878176	0.436621
H	-2.521658	-0.302093	-0.000878
H	1.077632	-2.154103	-1.194518
H	2.185447	-1.490295	0.020421
H	0.823698	-2.523448	0.529519
H	3.863141	0.468570	1.535931
H	2.572505	1.376175	0.697062
H	3.153056	1.887180	2.276179
H	3.327827	-0.433138	4.087777
H	1.670504	-1.003980	4.426854
H	2.630220	-1.746870	3.155539

(Me_2B)(Me_2As)PH

20

E= -2762.389266

As	-0.126799	-0.104734	0.065178
P	-0.064982	-0.373141	2.375817
B	1.774509	-0.050695	2.766325
C	-2.082435	0.092994	-0.079042
C	0.312048	1.815188	-0.055490
C	2.163579	0.952203	3.914021
C	2.902193	-0.843350	2.008827
H	-0.640647	0.852362	2.804584
H	-2.325897	0.378117	-1.104902
H	-2.561189	-0.861777	0.142035
H	-2.462143	0.855300	0.603681
H	0.133317	2.158708	-1.077341
H	-0.293197	2.405392	0.635276
H	1.368774	1.957016	0.179034
H	3.297121	-1.596581	2.705212
H	2.598093	-1.364854	1.098720
H	3.750797	-0.189439	1.776568
H	3.106680	0.697329	4.407483
H	2.320381	1.927811	3.429173
H	1.390762	1.107353	4.671031
H	1.717217	-0.093888	6.136384
H	2.944647	-0.998783	5.212785
H	2.979504	0.783900	5.233895
H	-0.669691	0.761681	-0.857629
H	0.993091	0.159913	-0.904920
H	-0.363438	-0.964798	-0.842789
H	-2.453542	-0.474689	2.095678
H	-1.451301	-0.390824	3.563191
H	-1.938591	1.081778	2.735786

(Me_2Al)(MeO)P•

15

E= -778.724263

C	0.006581	-0.004728	-0.462225
Al	0.011757	-0.001846	1.497031
P	2.090280	-0.051282	2.592298
O	1.435140	-0.064851	4.127188
C	2.326476	-0.095099	5.232254
C	-1.617793	0.045280	2.574655

(Me_2Al)(MeO)PH

16

E= -779.349089

C	-0.066018	0.159192	-0.040461
O	-0.103676	0.131269	1.372511
P	1.344069	-0.228083	2.153987
Al	0.535855	-0.045435	4.354668
C	1.855198	0.177644	5.784054
C	-1.381066	-0.241952	4.665498
H	0.487699	1.033063	-0.408065
H	0.397654	-0.746854	-0.450923
H	-1.097067	0.224186	-0.391685
H	-1.649918	-0.206989	5.725197
H	-1.940226	0.543088	4.145208
H	-1.743738	-1.191014	4.255271
H	1.584073	1.011622	6.441657
H	1.884635	-0.715515	6.420135
H	2.872101	0.355547	5.423218
H	1.958316	1.069083	2.207422

(Me_2Al)(MeS)P•

15

E= -1101.730300

C	0.025106	0.035006	-0.081628
Al	0.124345	0.023111	1.871519
C	1.819212	0.038297	2.847763
P	-1.920278	-0.010195	3.014571
S	-1.115118	-0.013204	4.940956
C	-2.610978	-0.038598	5.980958
H	-2.278611	-0.041823	7.019389
H	-3.216579	0.847613	5.793767
H	-3.194963	-0.936523	5.781309
H	0.547399	-0.832498	-0.502143
H	-0.997407	0.025314	-0.468440
H	0.526301	0.920462	-0.490246
H	2.680406	0.050455	2.172789
H	1.895978	0.912561	3.503678
H	1.915706	-0.838445	3.497688

(Me_2Al)(MeS)PH

16

E= -1102.353036

C	-0.097353	-0.065541	-0.025232
S	0.141778	0.761037	1.581648
P	1.933020	-0.189912	2.252494
Al	1.718805	0.036042	4.574517
C	3.380715	0.280566	5.576662
C	-0.035601	-0.151872	5.407031
H	-1.049962	0.292978	-0.418091
H	0.698989	0.196484	-0.721496
H	-0.143000	-1.147399	0.098046
H	-0.070094	-1.040693	6.047945
H	-0.255735	0.703952	6.055450
H	-0.843705	-0.231913	4.675639
H	3.328482	-0.163344	6.575892
H	4.250484	-0.141798	5.064117
H	3.587356	1.348904	5.716418
H	2.887483	0.843146	2.045890

(Me_2Al)(MeSe)P•

15

E= -3105.116060

C	0.042613	0.035370	-0.112820
Al	0.143128	0.022349	1.840761
C	1.850238	0.033610	2.800863
P	-1.893562	-0.006896	2.989923
Se	-1.033595	-0.012003	5.028535
C	-2.691024	-0.035362	6.071407
H	-2.410858	-0.040498	7.124833
H	-3.274080	0.854869	5.843457
H	-3.257210	-0.932976	5.830261
H	0.563463	-0.832241	-0.534910
H	-0.980580	0.026887	-0.497841
H	0.543725	0.920913	-0.521447
H	2.703459	0.046792	2.115759
H	1.938429	0.906713	3.457289
H	1.956374	-0.845994	3.445859

(Me_2Al)(MeSe)PH

16

E= -3105.738117

C	-0.151130	-0.052954	-0.104675
Se	0.126235	0.934025	1.564832
P	1.973950	-0.176403	2.278991
Al	1.745063	0.015669	4.600014
C	3.389476	0.283300	5.625937
C	-0.018554	-0.200072	5.407521
H	-1.070809	0.334180	-0.545260
H	0.682143	0.117038	-0.783769
H	-0.262409	-1.114939	0.105549
H	-0.003769	-0.939497	6.215749
H	-0.353908	0.742767	5.856327
H	-0.778532	-0.498414	4.680623
H	3.326106	-0.145783	6.630868
H	4.268359	-0.141785	5.131291
H	3.589238	1.354807	5.750907
H	2.970203	0.818698	2.092028
H	2.056788	-0.095117	4.511076
H	4.893840	0.134089	1.070118
H	3.803956	-0.350070	-0.235214
H	3.938203	1.342367	0.219726
H	-1.360866	-1.224558	-1.204818
H	-2.090466	-0.487514	0.235999
H	-1.812028	0.495302	-1.218899
H	0.399031	0.865310	-1.949522
H	1.698434	0.915820	-0.752571
H	1.289211	-0.617198	-1.549113

(Me_2Al)(Me_2N)P•

19

E= -798.169796

N	-0.093918	0.044375	-0.090098
P	0.202332	-0.113953	1.572444
Al	2.484227	0.100567	1.956169
C	-1.409612	-0.312143	-0.596139
C	0.876369	0.312493	-1.133979
C	2.931295	0.010493	3.862966
C	3.912810	0.322195	0.621795
H	3.598792	-0.834640	4.068965
H	3.470472	0.911194	4.179966

(Me_2Al)(Me_2N)PH

20

E= -798.791394

N	0.146883	-0.109201	-0.177997
P	0.211497	-0.139536	1.550019
Al	2.501623	-0.058045	2.004747
C	-1.141841	-0.485402	-0.745716
C	0.733435	1.022133	-0.872341
C	3.073389	0.463274	3.803240
C	3.746137	-0.668325	0.623302
H	3.852093	1.233664	3.763563
H	2.258551	0.847184	4.423480
H	3.513911	-0.390161	4.332518
H	4.438194	-1.429542	0.999489
H	3.213394	-1.086119	-0.235892
H	4.366295	0.158380	0.255907
H	-1.016018	-0.740947	-1.803076
H	-1.534025	-1.360627	-0.226652
H	-1.891271	0.321059	-0.677080

H	0.703304	0.852240	-1.953549
H	0.207676	1.969806	-0.661677
H	1.782248	1.145059	-0.587862
H	-0.142490	1.201854	1.923174

(Me_2Al)(Me_2P)P•

19

E= -1084.801334

P	0.006257	-0.260852	0.025910
P	0.056081	-0.167013	2.154024
Al	2.319799	0.257773	2.484465
C	1.222465	-1.508759	-0.600195
C	-1.592289	-1.092687	-0.374398
C	3.008382	0.041838	4.303366
C	3.502842	0.872560	1.045754
H	4.180699	0.069675	0.728999
H	2.968521	1.221804	0.157647
H	4.143590	1.690243	1.394456
H	3.970098	-0.483443	4.307718
H	3.192399	1.022128	4.760288
H	2.325409	-0.499009	4.963973
H	-1.715315	-1.133245	-1.459348
H	-1.637655	-2.106589	0.029711
H	-2.413151	-0.508893	0.044569
H	1.096255	-1.610314	-1.680978
H	2.239866	-1.163257	-0.410572
H	1.079117	-2.485576	-0.132630

(Me_2Al)(Me_2P)PH

20

E= -1085.432007

P	0.021066	-0.087562	0.151524
P	0.545764	-1.107444	2.070769
Al	2.538408	0.024508	2.532271
C	1.051815	-1.149587	-0.964561
C	-1.628722	-0.877166	-0.144544
C	2.926421	0.527651	4.382726
C	3.809606	0.353725	1.079292
H	4.703119	0.883841	1.422455
H	4.142495	-0.583184	0.617193
H	3.356775	0.951828	0.280043
H	3.904082	0.145545	4.698275
H	2.978296	1.618854	4.480861
H	2.180686	0.170107	5.097840
H	-1.970051	-0.617515	-1.150570
H	-1.592047	-1.965092	-0.044404
H	-2.355647	-0.482298	0.568267
H	0.774124	-0.936126	-1.999860
H	2.108815	-0.903416	-0.846687
H	0.910796	-2.217221	-0.774195
H	-0.218317	-0.265328	2.925350

(Me_2Al)(Me_2As)P•

19

E= -2979.347929

As	-0.280728	-0.123922	-0.009570
P	-0.365370	-0.156256	2.271598
Al	1.921016	0.027659	2.692619
C	-1.908814	-1.150771	-0.426214
C	1.042295	-1.512826	-0.489829

C	2.593588	-0.767122	4.350312
C	3.123581	0.956706	1.453727
H	-1.989923	-1.255731	-1.510194
H	-1.882159	-2.138249	0.035697
H	-2.776226	-0.602535	-0.057309
H	0.975232	-1.691381	-1.565284
H	2.048729	-1.155422	-0.264022
H	0.850032	-2.446242	0.040953
H	3.930597	0.290924	1.123653
H	2.625957	1.348484	0.562206
H	3.613624	1.800104	1.954594
H	3.594982	-0.409441	4.609294
H	1.931742	-0.566000	5.199117
H	2.653297	-1.858670	4.260331

(Me_2Al)(Me_2As)PH

20

E= -2979.981686

As	-0.389651	-0.260756	0.065513
P	-0.461204	-0.433850	2.392600
Al	1.783016	-0.036837	2.871288
C	-2.274503	0.258375	-0.182953
C	0.369525	1.550887	-0.156220
C	2.230984	1.054025	4.433484
C	3.162825	-0.827635	1.730326
H	-0.909848	0.879798	2.711239
H	-2.434324	0.505325	-1.235022
H	-2.916826	-0.582287	0.082198
H	-2.535071	1.120348	0.433901
H	0.240408	1.866754	-1.194480
H	-0.119910	2.272266	0.501328
H	1.441111	1.526951	0.058482
H	3.746123	-1.574735	2.281721
H	2.760808	-1.318491	0.839404
H	3.879509	-0.067212	1.398126
H	3.148294	0.713694	4.925061
H	2.406782	2.093557	4.128826
H	1.432917	1.075028	5.181370

(Me_2Ga)(MeO)P•

15

E= -2461.169270

C	0.007629	0.027517	-0.474401
Ga	0.013334	0.016572	1.497869
P	2.070307	-0.073188	2.598580
O	1.431535	-0.080811	4.136666
C	2.331787	-0.131653	5.233814
C	-1.625035	0.065959	2.585689
H	1.729891	-0.127248	6.142731
H	2.936197	-1.044322	5.204444
H	2.997763	0.737596	5.236565
H	-0.645940	0.819000	-0.853246
H	1.003035	0.165706	-0.901295
H	-0.390646	-0.922126	-0.847503
H	-2.301434	-0.747296	2.305655
H	-1.385763	-0.019394	3.646440
H	-2.162320	1.005452	2.421576

(Me_2Ga)(MeO)PH

16

E= -2461.794067

C	-0.071362	0.197396	-0.041275
O	-0.099183	0.137780	1.371034
P	1.368068	-0.151458	2.138118
Ga	0.582777	-0.055184	4.337247
C	1.905188	0.110004	5.789349
C	-1.351370	-0.242661	4.626129
H	0.425639	1.110833	-0.393331
H	0.443525	-0.669975	-0.473697
H	-1.106475	0.206736	-0.386128
H	-1.688123	0.281263	5.523840
H	-1.900161	0.126011	3.757637
H	-1.599099	-1.303016	4.746908
H	1.680418	0.987274	6.404104
H	1.849051	-0.763443	6.447091
H	2.930474	0.198674	5.425061
H	1.900565	1.179641	2.221062

(Me_2Ga)(MeS)P•

15

E= -2784.173650

C	0.029934	-0.042447	-0.515237
Ga	-0.114464	-0.002766	1.448375
P	1.907624	-0.055798	2.613501
S	1.076619	-0.031267	4.528872
C	2.558860	-0.070195	5.588193
C	-1.829811	0.058988	2.411177
H	2.213651	-0.049819	6.622203
H	3.127467	-0.982903	5.412059
H	3.185531	0.800349	5.396348
H	-0.662029	0.672228	-0.970028
H	1.040447	0.173657	-0.867104
H	-0.248337	-1.036788	-0.881240
H	-2.676838	0.037073	1.721894
H	-1.916336	-0.785143	3.101204
H	-1.894579	0.969596	3.014238

(Me_2Ga)(MeS)PH

16

E= -2784.796718

C	-0.089131	-0.041479	-0.408373
S	-0.283948	0.728443	1.232572
P	1.323758	-0.197648	2.292458
Ga	0.547893	0.009874	4.483338
C	1.912931	0.187006	5.890871
C	-1.387280	-0.084543	4.800484
H	0.841257	0.272617	-0.881129
H	-0.119949	-1.128422	-0.333828
H	-0.930723	0.300869	-1.012360
H	-1.626063	-0.240302	5.854734
H	-1.857419	0.848210	4.473247
H	-1.836538	-0.886322	4.208591
H	1.703269	-0.489415	6.724309
H	2.922573	-0.009162	5.524822
H	1.892076	1.206736	6.290264
H	2.257228	0.873413	2.353151

(Me_2Ga)(MeSe)P•

15

E= -4787.559139

C	0.064648	-0.045263	-0.490993
Ga	-0.126842	-0.008378	1.468982
P	1.861048	-0.062557	2.688117
Se	0.931461	-0.031446	4.696572
C	2.550645	-0.074426	5.796857
C	-1.877362	0.055171	2.372574
H	2.233862	-0.067460	6.839814
H	3.110724	-0.982911	5.583453
H	3.156824	0.804463	5.585467
H	-0.565484	0.719986	-0.953964
H	1.095611	0.105863	-0.816082
H	-0.273393	-1.013945	-0.874646
H	-2.698315	0.069862	1.652147
H	-2.006012	-0.811629	3.027747
H	-1.953400	0.946310	3.002932

(Me2Ga)(MeSe)PH

16

E= -4788.181529

C	-0.115845	-0.054715	0.005155
Se	0.185906	0.897172	1.690919
P	2.096966	-0.165493	2.305070
Ga	1.966417	0.001468	4.627657
C	3.666978	0.247667	5.590157
C	0.212968	-0.178814	5.493763
H	0.682939	0.165886	-0.700380
H	-0.178626	-1.125755	0.188259
H	-1.065860	0.307033	-0.390232
H	3.662750	-0.270142	6.552642
H	4.523829	-0.096747	5.006963
H	3.814362	1.314337	5.792171
H	0.280204	-0.758253	6.418239
H	-0.163792	0.815713	5.756375
H	-0.519125	-0.639799	4.828051
H	3.044561	0.874908	2.110880

(Me2Ga)(Me2N)P•

19

E= -2480.614533

C	-0.003793	-0.011700	-0.004881
Ga	-0.009515	0.004234	1.970024
C	1.706623	0.021966	2.955712
P	-2.149330	0.038114	2.839405
N	-2.045115	-0.034755	4.529941
C	-0.844664	-0.164777	5.332563
C	-3.241755	0.242049	5.308030
H	0.518286	0.870435	-0.389375
H	0.537312	-0.889280	-0.373560
H	-1.009460	-0.026873	-0.429865
H	2.528874	0.288925	2.287042
H	1.693923	0.727406	3.790296
H	1.924629	-0.968883	3.368629
H	-3.142360	1.188301	5.855605
H	-4.104950	0.314687	4.646972
H	-3.420179	-0.558911	6.034844
H	-1.075724	-0.703236	6.257294
H	-0.081059	-0.724251	4.795636
H	-0.432969	0.816974	5.603400

(Me2Ga)(Me2N)PH

20

E= -2481.236281

N	0.110582	0.156234	-0.218727
P	0.142428	0.126584	1.508189
Ga	2.416968	0.128619	1.986435
C	-1.178941	0.505810	-0.800732
C	0.759766	-0.920940	-0.942176
C	3.011674	-0.441045	3.777350
C	3.652014	0.849334	0.630811
H	4.291566	1.630255	1.052406
H	4.312575	0.057376	0.261894
H	3.102753	1.260717	-0.218488
H	3.839235	-1.152622	3.698073
H	3.385227	0.422521	4.337586
H	2.212229	-0.903406	4.359586
H	0.756012	-0.704441	-2.015437
H	1.801830	-1.017821	-0.626758
H	0.263503	-1.895095	-0.789014
H	-1.039355	0.812656	-1.842592
H	-1.896819	-0.331397	-0.785911
H	-1.618378	1.341672	-0.255021
H	-0.112925	-1.253490	1.815414

($(Me_2Ga)(Me_2P)P\bullet$)

19

E= -2767.244193

C	0.009881	-0.013458	0.064512
P	0.088837	-0.210136	1.899319
C	1.885626	0.114494	2.210479
P	-0.348382	-2.275215	2.234403
Ga	0.483393	-2.470663	4.393853
C	0.714822	-0.908053	5.576755
C	0.918120	-4.295582	4.998448
H	0.180959	1.034767	-0.192241
H	0.750250	-0.632380	-0.447567
H	-0.985434	-0.294411	-0.283229
H	2.109770	1.146963	1.930891
H	2.103297	-0.001767	3.273545
H	2.527303	-0.556896	1.635413
H	0.921777	-4.376067	6.088186
H	0.217852	-5.028997	4.591184
H	1.916311	-4.570568	4.640341
H	1.777821	-0.759864	5.796986
H	0.316975	0.012852	5.146139
H	0.215831	-1.088565	6.533810

$(Me_2Ga)(Me_2P)PH$

20

E= -2767.875169

P	0.054080	-0.139682	0.107782
P	0.470946	-1.135014	2.063911
Ga	2.547713	-0.176431	2.510733
C	1.004978	-1.321983	-0.957695
C	-1.652136	-0.802406	-0.185319
C	2.942401	0.333886	4.371752
C	3.854565	0.031598	1.050106
H	3.966940	0.068171	4.646764
H	2.851899	1.421045	4.473610
H	2.254773	-0.125939	5.084326
H	4.773478	0.513131	1.391694

H	4.117122	-0.943812	0.627816
H	3.429548	0.632101	0.239781
H	0.758880	-1.121540	-2.003540
H	2.077309	-1.160931	-0.832147
H	0.773259	-2.367251	-0.734718
H	-1.958653	-0.551023	-1.204553
H	-1.707907	-1.885664	-0.048965
H	-2.353276	-0.324386	0.502070
H	-0.223677	-0.203243	2.882912

(Me2Ga)(Me2As)P•

19

E= -4661.790846

As	0.045057	-0.207884	-0.042249
P	0.036949	0.055927	2.228977
Ga	2.340834	0.131134	2.541472
C	-1.659024	-1.167115	-0.278365
C	1.259980	-1.734100	-0.363022
C	3.046140	-0.589276	4.235469
C	3.536179	0.904876	1.174637
H	-1.783103	-1.406258	-1.336672
H	-1.680910	-2.086300	0.308102
H	-2.477647	-0.518642	0.035661
H	1.153291	-2.044689	-1.404717
H	2.292600	-1.418691	-0.202157
H	1.023046	-2.575477	0.289361
H	4.035581	-0.188605	4.469144
H	2.372476	-0.381605	5.070381
H	3.136827	-1.678361	4.158337
H	4.333285	0.196682	0.925388
H	3.012408	1.182246	0.257688
H	4.021840	1.800974	1.574676

(Me2Ga)(Me2As)PH

20

E= -4662.424567

As	-0.054218	-0.096221	0.301551
P	0.593613	-1.347417	2.181488
Ga	2.598009	-0.266882	2.663392
C	-1.738758	-1.064186	-0.041381
C	1.049364	-1.114560	-0.977725
C	3.017184	0.123724	4.548165
C	3.838506	0.161758	1.191362
H	-2.122069	-0.764593	-1.019891
H	-1.582697	-2.144024	-0.022728
H	-2.474722	-0.792469	0.717091
H	0.721877	-0.862450	-1.988706
H	2.097263	-0.830642	-0.873526
H	0.945644	-2.190284	-0.823752
H	4.068367	-0.079293	4.771190
H	2.845210	1.188436	4.741607
H	2.392646	-0.445371	5.239612
H	4.762093	0.618440	1.553855
H	4.098758	-0.741592	0.630771
H	3.367162	0.854114	0.486124
H	-0.143304	-0.603916	3.144119

(Me3Si)(MeO)P•

19

E= -865.727663

C	-0.046410	0.000000	0.044296
O	-0.013469	0.000000	1.462516
P	1.476626	-0.000000	2.216888
Si	0.546984	0.000000	4.291077
C	1.934177	-0.000000	5.558454
C	-0.516127	1.538002	4.465041
C	-0.516127	-1.538002	4.465041
H	-1.095424	0.000000	-0.252461
H	0.442886	0.891972	-0.361527
H	0.442886	-0.891972	-0.361527
H	1.516034	-0.000000	6.569952
H	2.568836	-0.885181	5.461162
H	2.568836	0.885181	5.461162
H	-1.054770	-1.534660	5.417924
H	-1.249420	-1.578161	3.655637
H	0.089606	-2.446903	4.418975
H	-1.054770	1.534660	5.417924
H	0.089606	2.446903	4.418975
H	-1.249420	1.578161	3.655637

(Me_3Si)(MeO)PH

20

E= -866.354334

C	-0.032384	-0.089717	0.046368
O	-0.033022	-0.176779	1.457680
P	1.425298	0.119432	2.242411
Si	0.517437	0.031566	4.326957
C	1.925391	-0.020851	5.569720
C	-0.516870	1.580956	4.545686
C	-0.553846	-1.501559	4.449537
H	-1.072503	-0.129210	-0.280394
H	0.418073	0.847960	-0.302114
H	0.510378	-0.929783	-0.405578
H	1.525797	-0.050123	6.588233
H	2.546959	-0.910088	5.432460
H	2.568690	0.859677	5.490182
H	-1.143643	-1.505986	5.371241
H	-1.238112	-1.538459	3.598330
H	0.057848	-2.407631	4.429795
H	-1.011866	1.584458	5.521654
H	0.095763	2.483609	4.472992
H	-1.289942	1.633754	3.774478
H	1.959380	-1.208960	2.307919

(Me_3Si)(MeS)P•

19

E= -1188.729861

C	0.060058	-0.000000	-0.445537
S	-0.481325	0.000000	1.293872
P	1.356037	-0.000000	2.300252
Si	0.487837	0.000000	4.405108
C	1.968627	-0.000000	5.559048
C	-0.558233	1.533867	4.684155
C	-0.558233	-1.533867	4.684155
H	-0.841807	0.000000	-1.057941
H	0.645763	0.892776	-0.662469
H	0.645763	-0.892776	-0.662469
H	1.630841	-0.000000	6.600220
H	2.593498	-0.884763	5.410104
H	2.593498	0.884763	5.410104

H	-0.934067	-1.548061	5.712454
H	-1.417620	-1.555290	4.009063
H	0.020793	-2.446671	4.521754
H	-0.934067	1.548061	5.712454
H	0.020793	2.446671	4.521754
H	-1.417620	1.555290	4.009063

(Me3Si)(MeS)PH

20

E= -1189.355747

C	0.033580	-0.009884	0.109830
S	0.127783	0.067362	1.928339
P	2.252271	0.037216	2.197739
Si	2.406846	1.476180	3.954697
C	1.160291	1.067953	5.294758
C	4.155769	1.310896	4.618516
C	2.101290	3.197340	3.279735
H	-1.025610	0.045617	-0.145101
H	0.555332	0.830686	-0.346914
H	0.442310	-0.948486	-0.263454
H	4.308511	2.005377	5.450668
H	4.349925	0.300846	4.990028
H	4.900387	1.539643	3.851392
H	1.247950	1.777622	6.123767
H	0.137969	1.123283	4.911568
H	1.319668	0.062017	5.692381
H	2.172382	3.938982	4.081416
H	2.831560	3.460992	2.510030
H	1.102120	3.270548	2.841881
H	2.370717	-1.157120	2.959078

(Me3Si)(MeSe)P•

19

E= -3192.114794

C	0.072543	-0.000000	-0.584865
Se	-0.612053	0.000000	1.249205
P	1.331794	-0.000000	2.325040
Si	0.482446	0.000000	4.437211
C	1.977188	-0.000000	5.573788
C	-0.558525	1.534622	4.737425
C	-0.558525	-1.534622	4.737425
H	-0.792425	0.000000	-1.247892
H	0.669210	0.894545	-0.752305
H	0.669210	-0.894545	-0.752305
H	1.653163	-0.000000	6.619388
H	2.600068	-0.884655	5.416236
H	2.600068	0.884655	5.416236
H	-0.915231	-1.550007	5.772499
H	-1.430861	-1.560176	4.078886
H	0.019280	-2.446170	4.563896
H	-0.915231	1.550007	5.772499
H	0.019280	2.446170	4.563896
H	-1.430861	1.560176	4.078886

(Me3Si)(MeSe)PH

20

E= -3192.740398

C	-0.038935	0.224010	-0.416791
Se	-0.417347	-0.864011	1.167115
P	1.443167	-0.302464	2.354304

Si	0.517752	-0.085965	4.423090
C	1.950554	0.000408	5.634893
C	-0.458811	1.513324	4.419977
C	-0.595895	-1.534851	4.850126
H	-0.883056	0.087519	-1.093619
H	0.043236	1.273709	-0.141821
H	0.875726	-0.117411	-0.897478
H	1.574573	0.138609	6.653604
H	2.542089	-0.919343	5.621902
H	2.618205	0.836023	5.408531
H	-1.016418	-1.407548	5.852902
H	-1.428864	-1.615955	4.146442
H	-0.043386	-2.478289	4.833476
H	-0.928627	1.676680	5.394803
H	0.183327	2.371982	4.206271
H	-1.249483	1.482280	3.665439
H	1.971019	-1.606442	2.554387

(Me$_3$Si)(Me$_2$N)P•

23

E= -885.169130

N	-0.089851	0.005498	-0.078045
P	0.138272	0.183082	1.601353
Si	2.376293	0.108751	1.944864
C	3.152791	-1.549592	1.497066
C	2.507827	0.343976	3.806586
C	3.326160	1.495803	1.093234
C	-1.443068	0.158437	-0.584757
C	0.919270	0.057392	-1.115126
H	4.369257	1.488868	1.427074
H	3.324991	1.402656	0.004694
H	2.898869	2.469560	1.347189
H	4.203427	-1.561977	1.806086
H	2.639034	-2.364478	2.013961
H	3.120253	-1.763931	0.425788
H	3.557615	0.312721	4.115093
H	2.095273	1.306752	4.120124
H	1.976239	-0.442559	4.349126
H	-1.668753	-0.632426	-1.309330
H	-2.157403	0.090537	0.235988
H	-1.576790	1.129254	-1.081562
H	0.599807	-0.545972	-1.971386
H	1.089727	1.084817	-1.467492
H	1.865762	-0.347261	-0.760707

(Me$_3$Si)(Me$_2$N)PH

24

E= -885.793867

N	-0.254909	-0.296770	-0.084930
P	0.010998	-0.348361	1.619011
Si	2.233735	0.071870	1.958954
C	3.402932	-1.252079	1.305984
C	2.469074	0.224228	3.818456
C	2.599839	1.711451	1.112319
C	-0.787304	0.950569	-0.603688
C	0.645820	-0.958245	-1.006006
H	3.629008	2.028971	1.307019
H	2.475089	1.632813	0.028290
H	1.931649	2.500132	1.469252
H	4.435111	-1.016734	1.586355

H	3.161021	-2.233626	1.723837
H	3.361789	-1.330993	0.217096
H	3.509889	0.468768	4.053473
H	1.834258	1.009949	4.236567
H	2.225513	-0.711387	4.330449
H	-1.297875	0.764511	-1.555002
H	-1.514393	1.364668	0.097059
H	-0.009830	1.711852	-0.783701
H	0.147241	-1.099805	-1.971134
H	1.572565	-0.387488	-1.189524
H	0.922336	-1.942392	-0.622046
H	0.077678	-1.759725	1.780563

(Me3Si)(Me2P)P•

23

E= -1171.799903

Si	-0.064341	0.154494	-0.099213
P	-0.056197	0.421711	2.156661
P	2.021398	0.044465	2.610553
C	-1.837341	-0.254789	-0.562922
C	0.431952	1.758378	-0.950218
C	1.064051	-1.242505	-0.655704
C	2.188171	0.826295	4.278977
C	3.053489	1.207560	1.598916
H	0.965281	-1.384805	-1.736961
H	2.116549	-1.045915	-0.437557
H	0.795431	-2.181802	-0.165443
H	0.346849	1.641974	-2.035841
H	-0.223838	2.580626	-0.650992
H	1.459641	2.049976	-0.723012
H	-1.934432	-0.353975	-1.648889
H	-2.157998	-1.195347	-0.107171
H	-2.525633	0.528602	-0.233792
H	4.082595	1.152391	1.962733
H	3.051665	0.897898	0.553129
H	2.707487	2.241480	1.666375
H	3.208950	0.678718	4.640017
H	1.965837	1.895744	4.263047
H	1.506456	0.335272	4.975575

(Me3Si)(Me2P)PH

24

E= -1172.432101

Si	-0.041910	0.136071	-0.163972
P	0.029507	0.548141	2.074203
P	2.125161	-0.045826	2.567755
C	-1.826872	-0.301091	-0.551714
C	0.427690	1.714680	-1.067163
C	1.083629	-1.273078	-0.686878
C	2.211026	0.678308	4.272562
C	3.009821	1.302337	1.649815
H	0.980200	-1.458471	-1.761058
H	2.135858	-1.058427	-0.482695
H	0.828148	-2.194241	-0.156505
H	0.350167	1.567776	-2.149205
H	-0.241740	2.533863	-0.790572
H	1.450119	2.030784	-0.846174
H	-1.957163	-0.436483	-1.630155
H	-2.125000	-1.230525	-0.058957
H	-2.511103	0.487965	-0.228109

H	4.038804	1.346973	2.015427
H	3.049674	1.067240	0.584279
H	2.549217	2.285728	1.781569
H	3.240010	0.603809	4.635207
H	1.893083	1.723532	4.302279
H	1.576491	0.099819	4.947459
H	-0.558008	-0.700171	2.416261

$(Me_3Si)(Me_2As)P\bullet$

23

E= -3066.347458

Si	-0.020613	-0.143281	-0.036172
P	-0.050660	-0.291754	2.230096
As	2.198818	-0.154043	2.691198
C	1.349128	0.977380	-0.673037
C	-1.683618	0.565612	-0.546779
C	0.165199	-1.862729	-0.778953
C	2.160162	-1.006167	4.468322
C	2.957252	-1.686979	1.697669
H	3.183824	-1.081256	4.841512
H	1.583031	-0.381149	5.150973
H	1.714254	-2.000594	4.425572
H	3.966646	-1.861040	2.076715
H	2.360081	-2.589448	1.833477
H	3.022504	-1.447302	0.635998
H	1.261599	1.080666	-1.759660
H	1.279773	1.976015	-0.233782
H	2.346361	0.587293	-0.454164
H	-1.756994	0.618268	-1.637936
H	-2.508189	-0.053808	-0.183322
H	-1.822890	1.574777	-0.149887
H	0.105484	-1.801974	-1.870762
H	1.120676	-2.323793	-0.518944
H	-0.632812	-2.527441	-0.437146

$(Me_3Si)(Me_2As)PH$

24

E= -3066.981547

Si	-0.003550	-0.129744	-0.096084
P	-0.023014	-0.385128	2.164817
As	2.256331	-0.126386	2.670291
C	1.365029	1.006530	-0.703045
C	-1.671739	0.600584	-0.557397
C	0.176853	-1.829160	-0.875798
C	2.166943	-0.932778	4.468856
C	2.885165	-1.752562	1.743200
H	3.185055	-1.068793	4.841643
H	1.638544	-0.256909	5.143275
H	1.652061	-1.894423	4.448507
H	3.870568	-2.006628	2.139574
H	2.210554	-2.596947	1.898359
H	2.986388	-1.558295	0.674320
H	1.289272	1.140739	-1.787089
H	1.293986	1.991689	-0.234180
H	2.359887	0.608388	-0.485448
H	-1.762236	0.682204	-1.645347
H	-2.494071	-0.024245	-0.198501
H	-1.798256	1.601039	-0.134649
H	0.136094	-1.750959	-1.966950
H	1.123565	-2.306167	-0.610498

H	-0.632059	-2.491579	-0.555688
H	-0.363662	0.974516	2.406528

(Me3Ge)(MeO)P•

19

E= -2653.250087

C	-0.056191	-0.000000	0.044919
O	-0.012968	-0.000000	1.463046
P	1.483438	0.000000	2.203128
Ge	0.534902	-0.000000	4.339928
C	1.959934	0.000000	5.684792
C	-0.580613	1.601041	4.507126
C	-0.580613	-1.601041	4.507126
H	-1.107383	-0.000000	-0.243900
H	0.429902	0.892061	-0.364640
H	0.429902	-0.892061	-0.364640
H	-1.148307	-1.575679	5.440847
H	-1.280001	-1.643923	3.670196
H	0.033547	-2.503778	4.495134
H	1.521296	0.000000	6.685837
H	2.589895	-0.886729	5.586440
H	2.589895	0.886729	5.586440
H	-1.148307	1.575679	5.440847
H	0.033547	2.503778	4.495134
H	-1.280001	1.643923	3.670196

(Me3Ge)(MeO)PH

20

E= -2653.876705

C	-0.039623	-0.109172	0.051032
O	-0.038417	-0.149835	1.464371
P	1.429923	0.139606	2.231922
Ge	0.502336	0.040406	4.376897
C	1.952276	-0.019688	5.692834
C	-0.584842	1.649757	4.607758
C	-0.616244	-1.560151	4.471695
H	-1.081062	-0.138152	-0.272464
H	0.428748	0.807391	-0.328930
H	0.485103	-0.974476	-0.374029
H	-1.238354	-1.551818	5.370023
H	-1.261021	-1.592743	3.591801
H	0.003950	-2.458961	4.481973
H	1.534607	-0.053794	6.702186
H	2.571128	-0.908089	5.549018
H	2.588521	0.864442	5.615088
H	-1.101810	1.626929	5.570291
H	0.036136	2.547140	4.567512
H	-1.331655	1.708082	3.813476
H	1.950632	-1.194609	2.295969

(Me3Ge)(MeS)P•

19

E= -2976.252163

C	0.059040	-0.000000	-0.444860
S	-0.472553	0.000000	1.297517
P	1.371153	-0.000000	2.293909
Ge	0.462784	0.000000	4.454264
C	1.990349	-0.000000	5.678752
C	-0.627632	1.602686	4.730006
C	-0.627632	-1.602686	4.730006

H	-0.846189	0.000000	-1.052347
H	0.643392	0.892806	-0.665632
H	0.643392	-0.892806	-0.665632
H	-1.026366	-1.605730	5.747990
H	-1.462982	-1.624667	4.027585
H	-0.029763	-2.504838	4.586747
H	1.634441	-0.000000	6.712183
H	2.609777	-0.886498	5.527573
H	2.609777	0.886498	5.527573
H	-1.026366	1.605730	5.747990
H	-0.029763	2.504838	4.586747
H	-1.462982	1.624667	4.027585

(Me3Ge)(MeS)PH

20

E= -2976.877778

C	-0.032715	0.171298	-0.312183
S	-0.333668	-0.751313	1.230477
P	1.442018	-0.248522	2.319982
Ge	0.491386	-0.070454	4.453278
C	1.971640	0.001700	5.733769
C	-0.537974	1.590341	4.474580
C	-0.666233	-1.600544	4.832086
H	-0.916765	0.025265	-0.934332
H	0.093310	1.235668	-0.115312
H	0.840841	-0.215960	-0.835945
H	-1.119325	-1.491342	5.820932
H	-1.464526	-1.667319	4.090315
H	-0.093844	-2.530297	4.812707
H	1.578323	0.113873	6.747344
H	2.563425	-0.915479	5.696340
H	2.627924	0.848609	5.523374
H	-1.020824	1.720054	5.446363
H	0.109238	2.450344	4.290800
H	-1.311394	1.562415	3.704302
H	1.969496	-1.556073	2.499547

(Me3Ge)(MeSe)P•

19

E= -4979.637094

C	0.071752	0.000000	-0.584176
Se	-0.599881	-0.000000	1.254633
P	1.351369	0.000000	2.319459
Ge	0.459381	-0.000000	4.487441
C	1.998746	0.000000	5.697339
C	-0.627980	1.602683	4.781422
C	-0.627980	-1.602683	4.781422
H	-0.797792	-0.000000	-1.241249
H	0.666950	0.894692	-0.756189
H	0.666950	-0.894692	-0.756189
H	-1.002593	-1.612492	5.808485
H	-1.480486	-1.621427	4.099416
H	-0.034933	-2.504483	4.617460
H	1.653303	0.000000	6.734380
H	2.616542	-0.886427	5.539471
H	2.616542	0.886427	5.539471
H	-1.002593	1.612492	5.808485
H	-0.034933	2.504483	4.617460
H	-1.480486	1.621427	4.099416

(Me_3Ge)(MeSe)PH

20

E= -4980.262468

C	-0.045314	0.229480	-0.408799
Se	-0.413483	-0.867403	1.171447
P	1.457475	-0.311264	2.347586
Ge	0.498734	-0.086350	4.473887
C	1.975490	0.008115	5.757798
C	-0.525453	1.577714	4.457326
C	-0.662738	-1.604486	4.893471
H	-0.897643	0.103507	-1.077310
H	0.046412	1.276717	-0.127286
H	0.861844	-0.113973	-0.902127
H	-1.103195	-1.475442	5.885669
H	-1.472412	-1.681177	4.164686
H	-0.095192	-2.537455	4.884635
H	1.580878	0.145177	6.767852
H	2.563216	-0.912348	5.742194
H	2.636393	0.846691	5.529167
H	-1.026084	1.721954	5.417949
H	0.128887	2.432560	4.274535
H	-1.283569	1.542210	3.672199
H	1.966087	-1.621961	2.553545

(Me_3Ge)(Me_2N)P•

23

E= -2672.691733

N	-0.105195	0.008725	-0.061393
P	0.129522	0.275387	1.606077
Ge	2.434801	0.144888	1.936493
C	3.175604	-1.635784	1.558043
C	2.628568	0.499134	3.855308
C	3.453741	1.498701	0.942038
C	-1.453033	0.188667	-0.574270
C	0.906499	-0.013646	-1.097048
H	4.493602	1.495964	1.280541
H	3.442768	1.304524	-0.132080
H	3.035790	2.492054	1.117481
H	0.556601	-0.618171	-1.940176
H	1.131894	0.995316	-1.471307
H	1.829882	-0.460824	-0.731513
H	-1.704952	-0.619830	-1.269891
H	-2.169185	0.172774	0.247804
H	-1.556109	1.145316	-1.104828
H	3.683613	0.447801	4.136328
H	2.252544	1.492833	4.107699
H	2.078986	-0.238079	4.444475
H	4.224817	-1.668518	1.864598
H	2.626597	-2.396208	2.117033
H	3.122512	-1.887371	0.496755

(Me_3Ge)(Me_2N)PH

24

E= -2673.317383

N	-0.297723	-0.279491	-0.061889
P	-0.033161	-0.264241	1.642761
Ge	2.275531	0.100940	1.948253
C	3.417767	-1.381697	1.352438
C	2.573439	0.394186	3.864617
C	2.745953	1.722967	0.948333

C	-0.729013	0.983953	-0.634733
C	0.580503	-1.026802	-0.938709
H	3.787189	1.997479	1.136214
H	2.622397	1.563091	-0.125490
H	2.110016	2.559493	1.246559
H	0.096081	-1.171655	-1.910236
H	1.544161	-0.518566	-1.116449
H	0.788574	-2.011139	-0.514337
H	-1.232494	0.800523	-1.589914
H	-1.438970	1.473112	0.034696
H	0.105069	1.680588	-0.822729
H	3.628015	0.615101	4.049360
H	1.974886	1.233570	4.224584
H	2.304649	-0.493340	4.442157
H	4.457772	-1.188064	1.629511
H	3.108343	-2.319159	1.820453
H	3.367097	-1.504501	0.268902
H	0.018939	-1.670428	1.845008

$(Me_3Ge)(Me_2P)P$•

23

E= -2959.322485

Ge	-0.066350	0.174979	-0.082466
P	-0.077393	0.500709	2.233504
P	1.984996	0.041943	2.690360
C	-1.919230	-0.208274	-0.586516
C	0.507486	1.813204	-0.998687
C	1.073543	-1.329305	-0.612115
C	2.184630	0.842766	4.346774
C	3.048979	1.157538	1.657904
H	4.078514	1.073281	2.014832
H	3.029486	0.835201	0.615940
H	2.737403	2.202997	1.714017
H	3.201343	0.666285	4.706399
H	1.998430	1.918811	4.315851
H	1.490144	0.385324	5.053624
H	0.981423	-1.491712	-1.689437
H	2.123788	-1.146231	-0.377957
H	0.764263	-2.239616	-0.095061
H	0.424021	1.674020	-2.080054
H	-0.128240	2.652992	-0.710356
H	1.541906	2.066469	-0.761156
H	-1.994059	-0.330467	-1.670328
H	-2.265872	-1.126475	-0.108174
H	-2.578261	0.608296	-0.284027

$(Me_3Ge)(Me_2P)PH$

24

E= -2959.954624

Ge	-0.044550	0.165337	-0.148849
P	0.014760	0.614926	2.149998
P	2.082347	-0.072014	2.640473
C	-1.910488	-0.246324	-0.577727
C	0.500891	1.784388	-1.106476
C	1.097851	-1.342817	-0.653556
C	2.207220	0.656118	4.341414
C	3.017581	1.233440	1.710721
H	4.051140	1.234784	2.065898
H	3.036443	0.994502	0.645325
H	2.600964	2.236111	1.843641

H	3.234010	0.541113	4.699607
H	1.933033	1.713827	4.367615
H	1.552910	0.107547	5.022422
H	0.992216	-1.543812	-1.723091
H	2.149073	-1.137028	-0.442523
H	0.811390	-2.238589	-0.099083
H	0.420328	1.624012	-2.184798
H	-0.144494	2.621620	-0.832390
H	1.532588	2.056873	-0.876030
H	-2.020988	-0.390724	-1.655617
H	-2.229979	-1.160307	-0.072612
H	-2.568198	0.569198	-0.269831
H	-0.625927	-0.607181	2.492179

(Me3Ge)(Me2As)P•

23

E= -4853.870041

Ge	-0.050628	-0.078353	-0.103948
P	-0.029384	-0.322414	2.222237
As	2.224906	-0.084861	2.619247
C	1.316811	1.174013	-0.742765
C	-1.822010	0.607805	-0.581498
C	0.199305	-1.830317	-0.952612
C	2.287589	-1.006033	4.361385
C	3.013386	-1.542380	1.539114
H	0.108769	-1.726131	-2.037312
H	1.181489	-2.247728	-0.724925
H	-0.562804	-2.532921	-0.609193
H	4.044851	-1.681983	1.869947
H	2.464611	-2.476725	1.663209
H	3.022856	-1.260946	0.485650
H	3.326061	-1.048182	4.696959
H	1.707738	-0.435312	5.087962
H	1.885607	-2.017712	4.293610
H	-1.908497	0.696764	-1.667747
H	-2.604857	-0.065325	-0.225636
H	-1.986065	1.592113	-0.138287
H	1.198147	1.319406	-1.819923
H	1.211382	2.141791	-0.248620
H	2.326320	0.803232	-0.554938

(Me3Ge)(Me2As)PH

24

E= -4854.504018

Ge	-0.020879	-0.068265	-0.119609
P	0.046213	-0.408282	2.197025
As	2.329865	-0.041675	2.605847
C	1.342157	1.192955	-0.746198
C	-1.801215	0.640651	-0.524951
C	0.208520	-1.802009	-1.001290
C	2.371541	-0.911569	4.376563
C	2.988735	-1.601949	1.590215
H	0.116971	-1.684937	-2.084303
H	1.187716	-2.232669	-0.783400
H	-0.557663	-2.502931	-0.663192
H	4.006512	-1.817543	1.922351
H	2.366756	-2.484713	1.752024
H	3.018995	-1.368462	0.524731
H	3.412676	-1.009351	4.693615
H	1.846557	-0.286012	5.100422

H	1.903260	-1.896617	4.346930
H	-1.920887	0.753740	-1.605666
H	-2.578525	-0.034094	-0.160278
H	-1.942598	1.617696	-0.057935
H	1.216503	1.369421	-1.817923
H	1.249485	2.146852	-0.222971
H	2.350772	0.809561	-0.577794
H	-0.351768	0.925739	2.489337

7) [R$_2$P•]$_2$ dimers for R=EH$_n$

(F$_2$P•)$_2$

6

E= -1082.153255

P	-0.000344	0.000160	-0.000044
P	0.000016	-0.000416	2.273259
F	1.608286	-0.000019	2.388952
F	-1.608584	0.002989	-0.118174
F	0.261226	1.587137	-0.115289
F	-0.258384	-1.587688	2.391591

(Cl$_2$P•)$_2$

6

E= -2523.601642

P	-0.011993	0.060974	0.007602
P	0.006617	-0.151755	2.285321
Cl	2.052196	0.104557	2.503623
Cl	0.698755	-1.845768	-0.387145
Cl	-2.057652	-0.195357	-0.210097
Cl	-0.704068	1.755121	2.680395

(Br$_2$P•)$_2$

6

E= -10979.4855404

P	0.163249	-0.049940	0.006699
P	-0.098285	0.015318	2.268730
Br	2.088305	-0.011682	2.757052
Br	0.726541	2.106187	-0.224583
Br	-2.024087	-0.020876	-0.478622
Br	-0.659474	-2.141613	2.498944

[(HO)$_2$P•]$_2$

10

E= -986.057559

O	0.015405	-0.085433	-0.150633
P	0.012634	0.001079	1.504427
P	2.215690	-0.012690	1.664483
O	2.374840	1.576209	1.220840
O	-0.146014	-1.588276	1.946048
O	2.212597	0.076167	3.319322
H	-0.749711	0.361025	-0.513028
H	3.202920	1.717135	0.762077
H	2.980301	-0.365143	3.682557
H	-0.976724	-1.731587	2.399304

[(HS)$_2$P•]$_2$

10

E= -2277.949984

S	-0.273250	-0.117302	-0.461869
P	0.032623	-0.013446	1.646932

S	2.157377	0.227148	1.818491
P	-0.372617	-2.174275	2.048358
S	0.486936	-2.203336	4.000709
S	1.051899	-3.266835	0.874048
H	-0.894717	1.072594	-0.520872
H	0.098581	-3.849390	0.126709
H	-0.457107	-3.017604	4.502012
H	2.051545	1.064406	2.864673

[(HSe)$_2$P•]$_2$

10

E= -10291.4815156

P	0.000347	-0.000590	-0.001430
P	-0.000623	-0.000612	2.237138
Se	2.199711	-0.000073	2.869163
Se	1.264744	-1.876870	-0.231297
Se	1.477101	1.631178	-0.631631
Se	-0.544044	2.195762	2.467340
H	0.432514	-2.360850	-1.339111
H	2.110744	-1.381759	3.356844
H	-1.461256	1.903056	3.575102
H	0.392973	2.491878	-1.120195

[(H$_2$N)$_2$P•]$_2$

14

E= -906.576018

N	-0.411495	-0.485739	0.140827
P	-0.135798	-0.799491	1.784629
P	2.084296	-1.021034	1.708214
N	2.431183	0.291415	0.637417
H	-1.059293	-1.080815	-0.346978
H	0.367305	-0.157774	-0.418127
H	3.319618	0.191219	0.167274
H	2.370294	1.217034	1.044560
N	2.445308	-0.444815	3.261913
H	1.753360	0.128691	3.729714
H	2.979485	-1.041579	3.870360
N	-0.208041	0.740809	2.566584
H	-1.090038	0.905479	3.030622
H	0.018763	1.538828	1.985257

[(H$_2$P)$_2$P•]$_2$

14

E= -2052.943934

P	0.016008	-1.270457	0.257326
P	-0.159132	-0.387825	2.290207
P	1.861501	0.386471	2.774479
P	2.627945	1.266617	0.882753
H	-1.331439	-1.701205	0.190510
H	-0.190324	-0.116256	-0.544909
H	3.175374	0.111681	0.262532
H	3.859171	1.697597	1.434025
P	3.092834	-1.440543	3.028345
H	2.299358	-1.986341	4.069381
H	2.522486	-2.253456	2.011248
P	-1.371031	1.438931	1.955599
H	-1.136880	1.985799	3.242987
H	-0.400927	2.251214	1.307785

[(H$_2$As)$_2$P•]$_2$

14

E= -9631.150458

As	-0.100400	-0.010275	-0.025229
P	-0.026472	0.005802	2.315903
P	2.175933	0.028453	2.701004
As	2.571972	-2.244157	2.218470
As	-0.352717	2.295917	2.708926
As	2.217530	-0.209831	5.038662
H	0.451737	-1.422646	-0.151100
H	1.265067	0.623291	-0.251163
H	3.952102	-2.195210	2.853163
H	3.138196	-1.945769	0.840803
H	1.561640	1.139543	5.268313
H	0.898126	-0.953465	5.179499
H	-1.771476	2.270013	2.169505
H	0.217473	2.809966	1.395793

[$(H_3C)_2P\bullet]_2$

18

E= -842.377303

C	0.004410	0.083955	0.065232
P	0.004608	-0.164717	1.903337
P	2.196048	0.022440	2.243861
C	2.196188	-0.225391	4.082092
C	-0.488280	1.542819	2.434218
C	2.688873	-1.685412	1.713900
H	-1.026675	0.208149	-0.274810
H	0.586682	0.956991	-0.241815
H	1.793300	0.662445	4.574593
H	1.613055	-1.097699	4.389585
H	0.408403	-0.803228	-0.427571
H	3.227211	-0.350613	4.421966
H	2.613711	-1.771253	0.627566
H	3.734218	-1.852072	1.985350
H	2.074365	-2.463353	2.175086
H	0.126422	2.321067	1.973790
H	-1.533507	1.709734	2.162490
H	-0.413500	1.627886	3.520643

[$(H_3Si)_2P\bullet]_2$

18

E= -1847.933067

Si	-0.161042	-0.157613	0.116032
P	-0.094088	-0.067611	2.373240
Si	2.151777	0.123824	2.547033
P	-0.540090	2.117215	2.680491
Si	-2.680697	2.175638	1.958137
Si	-1.025704	1.951307	4.882156
H	0.439407	1.049556	-0.506582
H	0.592050	-1.366593	-0.301312
H	-2.134490	0.998115	5.141347
H	0.196426	1.486011	5.582244
H	-1.574490	-0.278292	-0.316805
H	-1.405295	3.299673	5.372907
H	-3.439266	0.966702	2.368620
H	-3.311251	3.386954	2.539569
H	-2.684167	2.274921	0.478311
H	2.745690	-1.221457	2.345793
H	2.712740	1.068822	1.548302
H	2.460848	0.611697	3.913300

[$(H_3Ge)_2P$•]$_2$

18

E= -8998.082017

Ge	0.168626	0.072930	-0.199643
P	0.056571	-0.204221	2.111602
P	2.260345	0.069103	2.471255
Ge	2.207394	0.277020	4.791188
Ge	-0.641526	1.968424	2.581250
Ge	2.777106	-2.203236	2.444181
H	-1.246928	-0.062337	-0.770924
H	0.752246	1.442072	-0.569596
H	1.915016	1.738653	5.144201
H	1.129557	-0.627889	5.399286
H	1.065335	-1.035672	-0.758690
H	3.588812	-0.113868	5.327350
H	2.399658	-2.750183	1.064323
H	4.284435	-2.358214	2.673040
H	2.003648	-2.964078	3.528128
H	0.399592	2.992671	2.115013
H	-1.971155	2.199376	1.855066
H	-0.853494	2.097829	4.092791

[$(H_2B)_2P$•]$_2$

14

E= -786.869395

B	0.099232	0.093765	-0.163440
P	-0.012322	-0.268329	1.634732
B	1.389312	0.000997	2.792388
P	-1.853448	-1.056596	2.413174
B	-2.079601	-2.879687	2.462140
B	-3.130510	0.120604	3.013525
H	1.137591	0.526183	-0.553787
H	-0.832885	-0.102599	-0.875167
H	-2.936965	1.292910	2.966761
H	-4.134379	-0.357776	3.438907
H	-3.112803	-3.274082	2.903171
H	-1.222350	-3.602654	2.066676
H	2.391500	0.436145	2.319284
H	1.272324	-0.254312	3.947846

1) [R^AR^DP•]$_2$ for R=EH_n

[$(H_2B)(H_2P)P$•]$_2$

14

E= -1419.900289

B	-0.194304	0.256775	0.277019
P	-0.082100	-0.226626	2.117837
P	2.110183	-0.087611	2.447435
P	3.187172	-1.577903	1.253930
P	-0.452594	1.971507	1.957081
B	2.501216	-0.376475	4.227780
H	-1.270020	0.172797	-0.229149
H	0.789079	0.453694	-0.371033
H	2.805446	-1.072436	-0.013570
H	2.216631	-2.614996	1.244926
H	3.581015	-0.783848	4.525862
H	1.719386	-0.019950	5.054873

H	-1.857685	1.869997	1.802072
H	-0.199708	2.102724	0.532054

[(H_2B)(H_2As)P•]$_2$

14

E= -5209.004796

B	0.146436	-0.029135	0.063276
P	-0.116963	-0.022428	1.963344
As	2.183669	-0.017971	1.512865
P	-0.257660	-2.208058	2.348083
B	-0.315281	-2.494598	4.175500
As	-2.255269	-3.000778	1.487857
H	0.142836	1.035759	-0.473740
H	0.069540	-1.045415	-0.559799
H	-1.912471	-2.632296	0.056032
H	-3.060322	-1.725849	1.688770
H	-0.840326	-3.481229	4.592763
H	0.290751	-1.766239	4.901110
H	2.250412	1.488652	1.329180
H	1.933924	-0.248783	-0.017272

[(H_2Al)(H_2As)P•]$_2$

14

E= -5644.192520

As	-0.035558	0.099950	-0.039645
P	-0.009932	-0.518495	2.215557
P	2.179114	-0.452255	2.779533
Al	2.892620	0.894584	0.973848
Al	-0.582352	1.610840	3.065196
As	2.298042	1.227056	4.402752
H	0.936972	-0.969105	-0.506314
H	1.064302	1.194580	-0.042867
H	3.668276	0.217782	-0.223197
H	2.894975	2.458932	1.239195
H	1.284138	2.249167	3.823766
H	1.257564	0.623022	5.329586
H	-1.381738	1.700762	4.423693
H	-0.472449	2.815418	2.038302

[(H_2Ga)(H_2P)P•]$_2$

14

E= -5219.995813

P	-0.122113	0.708252	-0.159274
P	0.142097	0.053092	1.932640
Ga	2.423703	0.251508	2.200095
P	-0.108650	-2.172457	1.880127
P	-0.529498	-2.367736	4.080284
Ga	-2.422721	-2.227890	1.821181
H	-3.080713	-3.638591	1.844087
H	-3.181768	-0.868914	1.865021
H	-1.489576	0.341026	-0.263513
H	0.339703	-0.440892	-0.858325
H	3.168786	1.438933	1.519478
H	3.109663	-0.769507	3.157136
H	-0.815381	-3.756815	4.056230
H	-1.914615	-1.979168	4.162141

[(H_2Ga)(H_2As)P•]$_2$

14

E= -9009.103559

Ga	-0.262206	0.148328	0.056094

P	0.514929	0.116344	2.236648
P	2.756166	0.279537	1.976010
As	3.198049	-1.873747	1.170103
As	0.027156	2.420091	2.184465
Ga	2.296788	-0.287186	4.201927
H	-1.800974	0.041558	-0.127406
H	0.811483	0.367443	-1.044384
H	2.393852	-1.710082	-0.112341
H	2.058675	-2.601832	1.869781
H	2.014436	0.910125	5.156448
H	2.296189	-1.792534	4.599300
H	-1.474651	2.222617	2.319127
H	-0.066602	2.551569	0.650305

[(H₃Si)(HO)P•]₂

14

E= -1416.975624

O	0.010923	0.205113	0.075360
P	0.067626	-0.007716	1.750564
P	2.187483	0.110486	2.316145
O	2.672757	-1.501983	2.232250
Si	-0.425794	2.132087	2.304361
Si	3.082346	0.730521	0.326501
H	0.569174	3.093831	1.764891
H	-1.766521	2.438682	1.752652
H	-0.451051	2.219327	3.784463
H	2.541256	2.054855	-0.056743
H	4.546389	0.838263	0.543624
H	2.814944	-0.285409	-0.715994
H	-0.211866	-0.620683	-0.357162
H	2.840322	-1.834154	3.114855

[(H₃Si)(HS)P•]₂

14

E= -2062.940100

S	-0.292761	0.157854	-0.286647
P	0.010952	0.102505	1.843941
P	2.185449	0.098164	2.291932
S	2.719959	-1.963408	2.036871
Si	-0.385032	2.292643	2.270672
Si	3.217042	0.868839	0.425244
H	0.443597	3.185336	1.422260
H	-1.821346	2.531837	2.002659
H	-0.068185	2.520719	3.699988
H	2.607265	2.178258	0.091079
H	4.644306	1.040927	0.784142
H	3.080529	-0.076185	-0.705708
H	-0.685517	-1.122951	-0.388196
H	2.821728	-2.241317	3.347529

[(H₃Si)(HSe)P•]₂

14

E= -6069.706150

Se	0.124775	-0.095912	-0.409613
P	-0.043367	0.046693	1.868144
Si	2.193421	0.132181	2.240829
P	-0.441751	2.259355	2.042328
Si	-2.678475	2.174789	1.669208
Se	-0.610457	2.401100	4.320828
H	-3.341185	1.299368	2.668277

H	-3.202960	3.556631	1.768146
H	-2.896811	1.632478	0.306796
H	2.718671	-1.249386	2.142080
H	2.855340	1.007670	1.241288
H	2.411829	0.675023	3.603012
H	0.609826	3.203610	4.453509
H	-1.094870	-0.899401	-0.541752

$[(H_3Si)(H_2N)P\bullet]_2$

16

E= -1377.236969

Si	-0.074852	-0.101824	0.083237
P	-0.026898	0.068247	2.338007
N	1.653147	0.198896	2.692041
P	-0.894665	2.100729	2.514983
Si	0.951762	3.397454	2.453288
N	-1.257801	2.319221	4.179295
H	0.619815	1.038390	-0.576543
H	0.639337	-1.352410	-0.281383
H	-2.197490	2.060485	4.439395
H	-1.478852	-0.165833	-0.388974
H	-0.588964	2.001178	4.867131
H	1.782373	3.019279	1.279013
H	0.529816	4.811922	2.308276
H	1.751551	3.247445	3.694330
H	2.302145	0.553256	2.005091
H	2.041976	-0.567360	3.217980

$[(H_3Si)(H_2P)P\bullet]_2$

16

E= -1950.435450

P	0.297893	-0.137695	-0.145526
P	0.064679	0.152803	2.069234
P	2.239955	0.082628	2.640580
P	2.182034	0.571221	4.807348
Si	-0.363172	-2.015847	2.552762
Si	2.793524	2.162578	1.949735
H	-0.905565	-0.873336	-0.310071
H	0.958857	1.293635	4.859190
H	1.602230	-0.651945	5.226230
H	-0.305144	1.092642	-0.511565
H	1.943787	3.194096	2.598730
H	4.218834	2.400278	2.278229
H	2.589618	2.217261	0.480670
H	-1.794330	-2.272226	2.256584
H	0.482840	-2.928729	1.743854
H	-0.096010	-2.231446	3.995667

$[(H_3Si)(H_2As)P\bullet]_2$

16

E= -5739.540446

Si	-0.002258	-0.013624	0.029348
P	0.037891	-0.013035	2.290553
P	2.277203	0.033101	2.492278
Si	2.328710	0.246602	4.741641
As	-0.339530	2.308432	2.519892
As	2.798332	-2.251418	2.468659
H	-1.449897	2.341591	1.481649
H	1.441816	-2.758616	2.933597
H	2.442623	-2.426372	1.004303

H	-1.332703	2.137146	3.655740
H	1.588656	-0.852115	5.414542
H	3.743093	0.246735	5.187068
H	1.689423	1.541992	5.086607
H	-1.422793	-0.037477	-0.398405
H	0.661775	1.192971	-0.527125
H	0.693766	-1.228949	-0.460094

[(H$_3$Ge)(HO)P•]$_2$

14

E= -4992.051400

O	-0.001809	0.144155	0.087861
P	0.051625	-0.054987	1.763388
P	2.167861	0.067414	2.342800
O	2.660497	-1.539951	2.212743
Ge	-0.422710	2.156516	2.316513
Ge	3.092958	0.765728	0.319495
H	0.652124	3.113958	1.788945
H	-1.773207	2.497744	1.684234
H	-0.508799	2.262721	3.840980
H	2.499887	2.114059	-0.088284
H	4.596964	0.921579	0.574414
H	2.864501	-0.294079	-0.756486
H	-0.229322	-0.684598	-0.336632
H	2.827418	-1.897038	3.085797

[(H$_3$Ge)(HS)P•]$_2$

14

E= -5638.015436

S	-0.286551	0.116949	-0.259514
P	-0.000198	0.063988	1.875212
P	2.175609	0.058525	2.326144
S	2.700945	-1.998056	2.001562
Ge	-0.384267	2.327155	2.284098
Ge	3.221415	0.891850	0.413195
H	0.470850	3.209788	1.369436
H	-1.868840	2.575111	2.020776
H	-0.034429	2.591840	3.749151
H	2.543575	2.212342	0.042822
H	4.680408	1.131140	0.807404
H	3.129306	-0.114082	-0.732142
H	-0.657496	-1.170184	-0.365136
H	2.787663	-2.323148	3.302758

[(H$_3$Ge)(HSe)P•]$_2$

14

E= -9644.781243

Se	0.110004	-0.076831	-0.415094
P	-0.051716	0.046917	1.866229
Ge	2.251379	0.126096	2.250111
P	-0.433116	2.259055	2.044874
Ge	-2.735997	2.180622	1.659661
Se	-0.596187	2.381834	4.326330
H	-3.401475	1.279860	2.705816
H	-3.268355	3.610282	1.763669
H	-2.968349	1.617743	0.256050
H	2.783698	-1.303915	2.150925
H	2.916632	1.023203	1.200691
H	2.484022	0.693885	3.651708
H	0.632024	3.171699	4.463043

H -1.118579 -0.866250 -0.550935

[(H3Ge)(H2N)P•]2

16

E= -4952.312277

N	-0.542771	0.827945	1.056276
P	-0.247987	-0.287439	2.334572
P	1.846557	0.027990	2.988687
N	1.802935	1.433784	3.974228
Ge	0.078547	-2.322394	1.247864
Ge	2.799246	1.026801	1.120088
H	-0.170983	0.658084	0.132842
H	1.249061	2.220431	3.663263
H	1.654835	1.250240	4.955391
H	-1.485151	1.183990	1.016423
H	2.334737	2.483185	1.010346
H	4.323205	0.983060	1.262766
H	2.395180	0.266582	-0.151915
H	-1.224386	-2.652393	0.507831
H	1.212646	-2.234935	0.213101
H	0.394143	-3.423934	2.262122

[(H3Ge)(H2P)P•]2

16

E= -5525.510525

P	0.306904	-0.154031	-0.129623
P	0.060566	0.147913	2.081792
P	2.233135	0.065494	2.652500
P	2.162963	0.579826	4.813251
Ge	-0.380578	-2.089493	2.564028
Ge	2.810974	2.201319	1.929152
H	-0.951048	-0.788294	-0.308163
H	0.955148	1.328681	4.843847
H	1.551050	-0.626241	5.238057
H	-0.190888	1.122029	-0.500456
H	1.928186	3.256298	2.607675
H	4.283860	2.441914	2.262843
H	2.595378	2.251082	0.413203
H	-1.866148	-2.343988	2.289886
H	0.481205	-3.006595	1.689962
H	-0.070679	-2.329993	4.043699

[(H3Ge)(H2As)P•]2

16

E= -9314.615169

Ge	0.004394	-0.015596	-0.059027
P	0.035993	-0.021885	2.272017
P	2.273121	0.022048	2.470676
Ge	2.329371	0.268757	4.785634
As	-0.326363	2.302742	2.494036
As	2.772953	-2.267625	2.469251
H	-1.555329	2.289332	1.598688
H	1.420462	-2.757991	2.963025
H	2.390134	-2.457813	1.013064
H	-1.173004	2.154367	3.746775
H	1.562886	-0.866832	5.475589
H	3.789857	0.277217	5.242953
H	1.666303	1.609350	5.122388
H	-1.458770	-0.080748	-0.508169
H	0.661248	1.258346	-0.604612

H	0.762852	-1.246705	-0.562036

8) [$R^AR^DP\bullet]_2$ dimers for R=EMe_n

[(Me_2B)(MeO)P•]$_2$

30

E= -1122.362747

C	-0.022575	-0.011878	0.001878
B	-0.016123	-0.013388	1.571250
C	1.318244	-0.010911	2.384938
P	-1.711546	0.079811	2.475083
O	-1.226517	-0.106424	4.070231
C	-2.235346	-0.037268	5.060113
P	-2.271135	-1.979281	1.921655
B	-2.966986	-1.655773	0.176244
C	-2.447367	-2.518231	-1.022975
O	-0.837731	-2.796237	1.593701
C	-0.204235	-3.423499	2.698444
C	-4.139924	-0.626118	0.003291
H	0.652736	-3.972117	2.306370
H	0.144942	-2.683851	3.427295
H	-0.882087	-4.123433	3.200950
H	2.110820	-0.573267	1.881239
H	1.661700	1.033535	2.429018
H	1.209114	-0.347000	3.417385
H	0.220767	-1.036469	-0.308638
H	-0.969551	0.259491	-0.473891
H	0.767039	0.630877	-0.400720
H	-1.735974	-0.034629	6.029603
H	-2.835428	0.875756	4.961483
H	-2.902664	-0.906238	5.007249
H	-4.888753	-0.984393	-0.710963
H	-4.638310	-0.315348	0.924001
H	-3.705248	0.281294	-0.440969
H	-3.099272	-3.402214	-1.087024
H	-2.541171	-1.998707	-1.981583
H	-1.429038	-2.889070	-0.893050

[(Me_2B)(MeS)P•]$_2$

30

E= -1768.350705

S	0.015216	-0.183069	-0.472269
P	0.012764	0.318604	1.593213
P	2.177519	0.612044	2.047695
B	2.513554	2.482102	1.783560
B	-0.769114	2.069629	1.642247
S	2.266451	0.369424	4.157698
C	2.103762	3.112046	0.402740
C	3.267840	3.336054	2.859312
C	-0.543324	2.937111	2.933703
C	-1.692016	2.584226	0.485145
C	0.164628	-1.994047	-0.350891
C	2.559517	-1.425577	4.250914
H	2.666463	-1.669952	5.308245
H	1.711086	-1.976018	3.843121
H	3.472929	-1.696515	3.721819
H	-2.315953	3.433037	0.778248
H	-1.041712	2.921786	-0.334744
H	-2.321062	1.802176	0.051015
H	-0.197757	3.939438	2.650156

H	-1.519148	3.093365	3.411437
H	0.134906	2.529140	3.685810
H	0.134630	-2.378824	-1.370768
H	1.114842	-2.272326	0.106015
H	-0.663968	-2.413294	0.219485
H	3.671717	4.270556	2.460203
H	2.543846	3.599266	3.643896
H	4.061454	2.785626	3.372151
H	1.522613	4.027587	0.571538
H	3.020018	3.441806	-0.103872
H	1.555048	2.467231	-0.286637

[(Me$_2$B)(MeSe)P•]$_2$

30

E= -5775.118455

Se	0.088555	-0.135326	-0.654144
P	0.032084	0.344678	1.557664
P	2.181124	0.669229	2.046073
B	2.508679	2.546891	1.818594
B	-0.806853	2.069223	1.640061
Se	2.235085	0.368958	4.289485
C	2.062396	3.232300	0.475610
C	3.302871	3.369167	2.892619
C	-0.584793	2.954160	2.920760
C	-1.785520	2.551783	0.513273
C	0.371812	-2.062034	-0.463853
C	2.494381	-1.570113	4.255173
H	2.568679	-1.895701	5.292928
H	1.636954	-2.048604	3.784062
H	3.412202	-1.813307	3.723090
H	-1.170285	2.995403	-0.283829
H	-2.346160	1.743298	0.036726
H	-2.477892	3.327972	0.851401
H	-0.309882	3.973854	2.622918
H	-1.549698	3.052932	3.434737
H	0.142795	2.589949	3.648551
H	0.400044	-2.476620	-1.471718
H	1.323000	-2.247758	0.032946
H	-0.449092	-2.504997	0.096987
H	3.755071	4.278696	2.487150
H	2.580629	3.684337	3.660567
H	4.061696	2.788248	3.423481
H	1.507194	4.152448	0.697589
H	2.966077	3.560334	-0.054109
H	1.475598	2.623234	-0.214606

[(Me$_2$B)(Me$_2$N)P•]$_2$

38

E= -1161.242009

B	0.669998	1.376654	-0.525821
P	0.182217	-0.155746	0.482906
P	2.108189	-0.673343	1.446910
N	1.972765	-2.234528	2.137059
N	-1.041939	0.326284	1.583515
B	2.484749	0.543452	2.846937
C	-0.274174	2.633969	-0.555290
C	1.975734	1.336217	-1.402875
C	2.256042	-3.345413	1.240229
C	0.886135	-2.533859	3.051028
C	2.549506	2.083166	2.515269

C	2.939972	0.024898	4.261066
C	-2.030464	-0.667905	1.965449
C	-0.768207	1.283880	2.632034
H	3.759422	0.621010	4.675897
H	2.095780	0.150011	4.954447
H	3.213588	-1.032177	4.274974
H	3.607914	2.374376	2.491214
H	2.104898	2.389820	1.565347
H	2.095761	2.676708	3.316882
H	1.134860	-3.418485	3.647323
H	0.723680	-1.698549	3.734337
H	-0.059203	-2.737506	2.521611
H	2.486076	-4.239961	1.828483
H	1.406912	-3.580586	0.578457
H	3.121542	-3.110241	0.619337
H	-0.168380	0.851340	3.453283
H	-0.227338	2.144179	2.236646
H	-1.709677	1.640320	3.061894
H	-1.743399	-1.230235	2.867521
H	-2.988335	-0.176119	2.167256
H	-2.176255	-1.378495	1.150718
H	1.823490	1.809849	-2.378315
H	2.741350	1.933689	-0.887549
H	2.406264	0.343156	-1.547000
H	-0.459207	2.965154	-1.582992
H	-1.229844	2.494272	-0.046326
H	0.249027	3.468507	-0.066822

$[(Me_2B)(Me_2P)P•]_2$

38

E= -1734.512889

P	-0.707319	0.706206	0.519951
P	0.637928	-0.234170	1.985510
B	2.429959	0.039033	1.438323
P	0.146175	-2.356923	2.114122
P	-0.012544	-2.908556	4.238539
B	1.471993	-3.434347	1.297266
C	-2.325967	0.271271	1.304223
C	-0.724475	-0.497620	-0.886689
C	2.090568	-3.007686	-0.084689
C	1.862401	-4.821221	1.928220
C	1.450128	-2.077740	5.011993
C	-1.333781	-1.714428	4.741212
C	2.790388	1.232992	0.479898
C	3.581100	-0.844817	2.045118
H	1.358374	-2.149453	6.099355
H	2.363955	-2.597036	4.716182
H	1.527342	-1.023893	4.729957
H	3.785433	1.643298	0.677737
H	2.824410	0.819191	-0.539402
H	2.061033	2.045467	0.457195
H	4.162189	-0.215949	2.732778
H	3.264101	-1.733574	2.594975
H	4.281419	-1.150619	1.259426
H	2.144131	-5.561007	1.172625
H	2.761011	-4.646875	2.539388
H	1.115059	-5.252802	2.597400
H	3.175601	-3.162497	-0.080829
H	1.696521	-3.685153	-0.853962
H	1.885436	-1.984340	-0.406271

H	-1.469583	-1.780872	5.823599
H	-1.080231	-0.683225	4.478426
H	-2.275954	-1.986204	4.261444
H	-1.504372	-0.199164	-1.592802
H	0.233826	-0.462880	-1.408896
H	-0.917129	-1.521933	-0.555379
H	-3.131807	0.580282	0.633737
H	-2.416694	-0.801830	1.496439
H	-2.436236	0.815276	2.244239

[(Me$_2$B)(Me$_2$As)P•]$_2$

38

E= -5523.609060

C	0.134437	0.050168	-0.086825
As	0.071257	0.113113	1.879448
C	2.014735	-0.016440	2.175708
P	-0.579112	-2.097856	2.178816
P	0.887353	-3.377689	1.196901
B	2.043399	-4.212779	2.441525
C	2.586894	-5.661960	2.151102
B	-0.697865	-2.452705	4.034316
C	-0.633863	-3.928402	4.576384
C	-1.003411	-1.276429	5.035751
As	-0.257467	-5.021488	0.018635
C	-1.341418	-3.787491	-1.066023
C	-1.671554	-5.499183	1.304646
C	2.579805	-3.425905	3.694006
H	-2.418494	-6.103662	0.784187
H	-1.245099	-6.093470	2.114186
H	-2.149917	-4.607828	1.715568
H	-1.608929	-1.600789	5.887810
H	-0.036898	-0.952768	5.450537
H	-1.459356	-0.390352	4.588342
H	-1.647051	-4.219530	4.884619
H	-0.271614	-4.680785	3.872805
H	-0.021709	-3.975112	5.484384
H	3.603693	-5.809400	2.528257
H	1.946639	-6.359918	2.711352
H	2.543302	-5.974930	1.105471
H	2.568556	-4.068544	4.581851
H	3.636013	-3.184092	3.514389
H	2.061506	-2.494581	3.931348
H	-2.080909	-4.372579	-1.617061
H	-1.853708	-3.058154	-0.434905
H	-0.698906	-3.269099	-1.779132
H	2.475561	0.916286	1.841264
H	2.215472	-0.141803	3.240784
H	2.444664	-0.852078	1.619767
H	0.569022	0.985084	-0.447225
H	0.742220	-0.788783	-0.432429
H	-0.878514	-0.043204	-0.480894

[(Me$_2$Al)(Me$_2$As)P•]$_2$

38

E= -5958.787372

C	0.264799	0.133355	-0.534549
Al	0.310388	0.007667	1.422197
C	2.002929	-0.059504	2.396136
P	-1.757235	-0.034831	2.503903
As	-2.110858	2.280977	2.394464

C	-0.294655	3.017185	2.080845
P	-1.102205	-0.039140	4.649739
Al	-3.157303	0.302409	5.713407
C	-4.755167	0.816203	4.711303
As	-1.004753	-2.308032	5.230913
C	-2.708030	-3.008657	4.508577
C	0.128949	-2.940958	3.746313
C	-2.740374	2.361517	0.529468
C	-3.191937	-0.012042	7.645184
H	-5.672792	0.548417	5.245188
H	-4.780393	1.898273	4.539297
H	-4.779140	0.341099	3.725020
H	-3.814318	-0.884539	7.879858
H	-2.201303	-0.195241	8.069921
H	-3.637425	0.834702	8.179963
H	-2.633953	-4.096465	4.435346
H	-3.525973	-2.775044	5.195995
H	-2.930253	-2.594123	3.523022
H	0.274031	-4.017446	3.860630
H	-0.341458	-2.753335	2.777784
H	1.100268	-2.447250	3.792262
H	-0.364233	4.107405	2.063670
H	0.361462	2.733410	2.906974
H	0.137874	2.701951	1.125474
H	-2.874509	3.410475	0.256250
H	-2.035385	1.898078	-0.162214
H	-3.702671	1.853216	0.458793
H	2.534683	-1.001460	2.217016
H	2.670696	0.741401	2.057207
H	1.866643	0.048836	3.475866
H	0.972070	-0.565413	-0.995535
H	-0.724312	-0.070427	-0.954865
H	0.562991	1.134425	-0.870907

[$(Me_2Ga)(MeS)P\bullet]_2$

30

E= -5568.424788

C	-0.007951	-0.019489	0.013831
Ga	-0.017240	0.015939	1.975520
C	1.609889	0.047542	3.099047
P	-2.021309	0.143238	3.159361
S	-3.290903	-0.880445	1.785979
C	-4.925603	-0.397304	2.429024
P	-1.794278	-1.378764	4.799224
Ga	0.077491	-2.620337	4.175550
C	1.464070	-3.077609	5.486239
S	-0.923288	-0.214334	6.358537
C	-2.425463	0.410725	7.178263
C	0.077586	-3.226736	2.293226
H	-5.670191	-0.848482	1.771939
H	-5.064816	-0.776858	3.441646
H	-5.037819	0.686695	2.416081
H	2.125076	-3.869597	5.127267
H	2.069533	-2.188882	5.689644
H	1.018216	-3.385093	6.435231
H	1.011530	-2.965431	1.785798
H	0.013692	-4.320165	2.285530
H	-0.767732	-2.854046	1.708135
H	-2.096277	1.008587	8.029105
H	-2.997435	1.041609	6.497323

H	-3.042112	-0.415795	7.530997
H	2.159299	0.969531	2.880364
H	1.406007	0.045345	4.173391
H	2.282754	-0.781170	2.857187
H	0.981995	0.190282	-0.397302
H	-0.319450	-1.011944	-0.326460
H	-0.724769	0.697836	-0.393036

[(Me2Ga)(Me2N)P•]2

38

E= -4961.298002

C	0.261330	0.947721	0.689614
Ga	0.825893	-0.581716	1.812656
C	2.743406	-0.736546	2.292578
P	-0.857254	-2.076436	2.333200
N	-0.298803	-3.549691	3.025299
C	-0.811317	-4.761814	2.407563
P	-0.977420	-0.739376	4.134601
Ga	0.690812	0.749837	4.772988
C	0.149635	2.639434	4.863803
N	-2.366243	0.267538	3.970839
C	-2.488201	1.158573	2.829097
C	-3.622516	-0.405697	4.278067
C	2.330470	0.013193	5.571250
C	1.058488	-3.678744	3.512473
H	1.154250	-4.603338	4.090658
H	1.807586	-3.700683	2.702733
H	1.300850	-2.847189	4.178223
H	3.219723	0.429152	5.090301
H	2.367807	0.290612	6.629506
H	2.379269	-1.074901	5.501806
H	0.651976	3.175402	5.672339
H	0.398734	3.140545	3.922214
H	-0.931990	2.710514	5.000038
H	3.201941	0.239691	2.471716
H	2.939475	-1.379117	3.150093
H	3.249338	-1.175345	1.424704
H	0.397736	1.900504	1.210135
H	0.891623	0.977451	-0.205409
H	-0.779572	0.876188	0.370597
H	-0.769770	-5.586042	3.127746
H	-1.853414	-4.619710	2.116579
H	-0.242092	-5.064506	1.513355
H	-3.306132	1.864663	3.006049
H	-1.568513	1.729008	2.696909
H	-2.698370	0.618295	1.892440
H	-4.400233	0.344506	4.452664
H	-3.957606	-1.066863	3.463418
H	-3.512534	-1.003834	5.184286

[(Me2Ga)(Me2As)P•]2

38

E= -9323.672183

C	0.130879	1.067927	0.738878
Ga	0.759666	-0.277698	2.025396
C	2.605404	-0.975704	1.950407
P	-0.918855	-1.412408	3.263414
As	-0.403581	-3.639601	3.700845
C	0.030999	-4.102215	1.830415
P	-1.594197	-0.620901	5.234177

Ga	0.508512	0.274229	4.991904
C	0.928391	1.941987	3.972478
As	-3.299159	0.906872	4.762116
C	-2.835107	1.620266	2.988495
C	-4.586085	-0.432301	4.104495
C	1.981221	-0.553766	6.009892
C	1.450506	-3.604411	4.363300
H	1.834694	-4.627263	4.331255
H	2.090199	-2.958944	3.762698
H	1.457889	-3.262957	5.397694
H	2.739713	-0.985691	5.352547
H	2.470515	0.218833	6.612033
H	1.623896	-1.332362	6.685972
H	1.878493	1.906877	3.428561
H	0.150395	2.313885	3.306739
H	1.065955	2.691311	4.761627
H	3.210365	-0.388252	1.255209
H	3.074757	-0.934529	2.936956
H	2.629755	-2.018372	1.621060
H	0.891547	1.842234	0.605465
H	-0.020870	0.584330	-0.232232
H	-0.807384	1.541367	1.027516
H	0.369616	-5.140447	1.804017
H	-0.866243	-4.012299	1.216550
H	0.818641	-3.468134	1.418957
H	-3.715605	2.131872	2.592376
H	-2.023023	2.342608	3.072800
H	-2.552368	0.813977	2.309037
H	-5.458356	0.088129	3.702359
H	-4.138647	-1.048101	3.320982
H	-4.904163	-1.069068	4.930742

[(Me$_3$Si)(MeO)P•]$_2$

38

E= -1731.542490

Si	-0.036741	0.266388	-0.150656
P	0.317221	0.070249	2.084470
P	2.501109	-0.085802	2.097971
Si	2.906377	-2.311488	2.442971
O	-0.326588	-1.476171	2.326970
O	2.904667	0.317610	3.687015
C	0.908655	-0.953352	-1.218352
C	-1.881267	0.011103	-0.376127
C	0.477334	2.013780	-0.600604
C	2.149784	-3.350098	1.082641
C	4.778604	-2.451989	2.437796
C	2.231647	-2.801291	4.120296
C	-1.151331	-1.638801	3.469426
C	3.098271	1.691248	3.969111
H	3.488571	1.759800	4.985428
H	3.814738	2.146530	3.274339
H	2.152172	2.243135	3.915694
H	-1.466647	-2.683449	3.489885
H	-0.608605	-1.410963	4.393996
H	-2.039451	-0.999049	3.415364
H	-2.158245	0.102720	-1.430855
H	-2.173791	-0.984976	-0.033513
H	-2.456029	0.747706	0.191622
H	0.691227	-0.756782	-2.273477
H	1.988268	-0.855240	-1.075654

H	0.624179	-1.984983	-1.001887
H	0.277973	2.202077	-1.660392
H	-0.069060	2.759736	-0.017498
H	1.546712	2.162491	-0.427891
H	2.369862	-4.406476	1.267950
H	1.065516	-3.217053	1.083095
H	2.539987	-3.095319	0.094687
H	5.082641	-3.483853	2.641393
H	5.200937	-2.158074	1.473212
H	5.214914	-1.812382	3.209516
H	2.727419	-3.706466	4.484566
H	2.404447	-1.998467	4.840688
H	1.159517	-2.995171	4.061140

[(Me$_3$Si)(MeS)P•]$_2$

38

E= -2377.538398

Si	-0.134102	0.257993	-0.064242
P	0.042448	-0.143333	2.168877
P	2.240803	-0.261644	2.387608
Si	2.934466	-2.434265	2.536403
S	-0.864074	-2.075968	2.288589
S	2.554301	0.252886	4.439973
C	0.722206	-1.020775	-1.135293
C	-1.968219	0.303685	-0.445585
C	0.650917	1.939963	-0.330320
C	2.495541	-3.341036	0.957942
C	4.798870	-2.303452	2.697877
C	2.226697	-3.322246	4.027544
C	-1.456544	-2.028565	4.011889
C	2.985420	2.013478	4.263881
H	3.228390	2.378776	5.262592
H	3.850423	2.136211	3.612437
H	2.139346	2.578587	3.872640
H	-1.990196	-2.963555	4.187594
H	-0.619540	-1.954628	4.706590
H	-2.134747	-1.188943	4.159748
H	-2.125860	0.553909	-1.499553
H	-2.437539	-0.663696	-0.250517
H	-2.479112	1.056074	0.161101
H	0.558881	-0.785349	-2.192047
H	1.800195	-1.024008	-0.955494
H	0.340846	-2.028383	-0.952801
H	0.575770	2.224042	-1.384895
H	0.151641	2.711157	0.262368
H	1.709016	1.931150	-0.056055
H	2.909183	-4.353863	1.008545
H	1.414865	-3.426245	0.830505
H	2.916498	-2.854980	0.074716
H	5.234861	-3.305591	2.766047
H	5.239997	-1.800475	1.833317
H	5.080288	-1.749182	3.596587
H	2.731202	-4.287064	4.145675
H	2.379636	-2.752103	4.947390
H	1.159133	-3.509923	3.900471

[(Me$_3$Si)(MeSe)P•]$_2$

38

E= -6384.305732

Si	-0.128817	0.258039	-0.079156

P	0.026186	-0.163808	2.153614
P	2.227229	-0.291298	2.383171
Si	2.952146	-2.455683	2.513876
Se	-0.980032	-2.201081	2.265825
Se	2.533207	0.254245	4.570090
C	0.698002	-1.036605	-1.156049
C	-1.957790	0.363915	-0.475680
C	0.706162	1.918229	-0.332487
C	2.525231	-3.350986	0.924326
C	4.815107	-2.302804	2.674037
C	2.258558	-3.378137	3.992194
C	-1.530614	-2.077014	4.141388
C	2.960320	2.143616	4.290660
H	3.196606	2.562362	5.269442
H	3.820679	2.235149	3.630461
H	2.097768	2.658142	3.870541
H	-2.103410	-2.977894	4.363456
H	-0.652678	-2.029368	4.783778
H	-2.152127	-1.195324	4.283635
H	-2.097864	0.638094	-1.526238
H	-2.459867	-0.591049	-0.302392
H	-2.450989	1.120671	0.140133
H	0.552144	-0.787725	-2.212264
H	1.773526	-1.073469	-0.965950
H	0.286887	-2.035232	-0.987301
H	0.641868	2.212228	-1.385100
H	0.228011	2.699055	0.265053
H	1.762555	1.876474	-0.055050
H	2.940963	-4.363283	0.967575
H	1.445893	-3.438214	0.786457
H	2.951551	-2.854524	0.049417
H	5.265044	-3.299271	2.734740
H	5.247897	-1.787768	1.812356
H	5.090070	-1.749882	3.575557
H	2.734763	-4.362145	4.059024
H	2.455532	-2.851185	4.929600
H	1.182189	-3.527830	3.889371

[(Me$_3$Si)(Me$_2$N)P•]$_2$

46

E= -1770.417067

N	-1.121276	0.468703	0.981134
P	-0.358873	-0.346424	2.285001
P	1.748639	0.184576	2.715365
N	1.685212	1.389615	3.959076
Si	-0.085081	-2.482188	1.576390
Si	2.811998	1.357611	1.092700
C	-0.671498	0.415225	-0.393536
C	0.699766	2.455945	3.891447
C	1.818938	0.870866	5.314028
C	-1.849557	1.682884	1.298439
C	2.020862	2.994133	0.594357
C	4.485676	1.737755	1.853748
C	3.050089	0.271997	-0.422670
C	-1.698613	-2.976518	0.753631
C	1.360555	-2.802630	0.415675
C	0.202033	-3.466102	3.147845
H	-1.225481	2.588066	1.222248
H	-2.694489	1.801132	0.610286
H	-2.240672	1.624810	2.315135

H	3.669915	0.805649	-1.150667
H	3.565844	-0.656304	-0.164827
H	2.111124	0.010904	-0.913316
H	2.535571	3.390380	-0.287525
H	0.960008	2.906210	0.350889
H	2.126026	3.731984	1.394184
H	0.985899	3.266834	4.570330
H	0.643640	2.863315	2.882149
H	-0.307837	2.111856	4.175285
H	5.084008	2.364715	1.184862
H	4.354905	2.268937	2.800087
H	5.045207	0.820855	2.056524
H	2.094961	1.686578	5.991158
H	0.885940	0.418299	5.687633
H	2.605734	0.116377	5.347099
H	1.431806	-3.877007	0.215326
H	1.268284	-2.291361	-0.545174
H	2.298680	-2.486887	0.879876
H	0.078096	1.184021	-0.633082
H	-0.236259	-0.559542	-0.616661
H	-1.524121	0.562525	-1.066708
H	0.341304	-4.526518	2.914363
H	1.098902	-3.113509	3.664874
H	-0.643140	-3.374702	3.834705
H	-1.668956	-4.027572	0.449586
H	-2.538742	-2.844594	1.440493
H	-1.899060	-2.371464	-0.134278

[(Me$_3$Si)(Me$_2$P)P•]$_2$

46

E= -2343.691620

P	-0.457353	0.759645	0.104652
P	-0.111613	-0.262235	2.059327
P	1.960686	0.206093	2.692034
P	1.780305	1.618951	4.382454
Si	0.198669	-2.450787	1.510217
Si	3.138305	1.436954	1.189816
C	-1.918173	-0.209295	-0.495705
C	0.316180	2.683817	3.996935
C	0.985244	0.485531	5.611774
C	-1.362233	2.251339	0.727495
C	2.415831	3.116833	0.756425
C	4.793724	1.697500	2.037113
C	3.372137	0.411463	-0.361313
C	-1.487580	-3.257696	1.304685
C	1.227724	-2.670971	-0.044216
C	1.054674	-3.249555	2.974323
H	-1.752519	2.804922	-0.131154
H	-2.188076	1.989038	1.393496
H	-0.673980	2.906068	1.263708
H	4.004417	0.959089	-1.068094
H	3.868007	-0.536441	-0.135532
H	2.418806	0.202433	-0.851075
H	3.122105	3.652931	0.113140
H	1.473166	3.029393	0.212888
H	2.257125	3.727973	1.648925
H	0.062842	3.257200	4.893012
H	0.567246	3.391606	3.205107
H	-0.554929	2.095709	3.694795
H	5.466228	2.251560	1.373741

H	4.684252	2.266560	2.963738
H	5.267989	0.743401	2.281256
H	0.756239	1.051626	6.518483
H	0.061050	0.045183	5.226191
H	1.680899	-0.314241	5.871857
H	1.299739	-3.731579	-0.305804
H	0.802367	-2.140590	-0.900911
H	2.240353	-2.291488	0.111936
H	-2.402473	0.375686	-1.281970
H	-1.589158	-1.150632	-0.938533
H	-2.650286	-0.415061	0.289932
H	1.147862	-4.326842	2.801705
H	2.051892	-2.833404	3.130183
H	0.480973	-3.104456	3.894203
H	-1.358104	-4.340067	1.199678
H	-2.109503	-3.081086	2.186624
H	-2.035086	-2.902647	0.429947

$[(Me_3Si)(Me_2As)P\bullet]_2$

46

E= -6132.788812

C	0.043134	0.005396	-0.123953
As	-0.029694	-0.110667	1.843394
C	1.921171	-0.046912	2.117720
P	-0.434698	2.166810	2.262600
Si	-2.393698	2.572918	1.174372
C	-3.115450	4.140768	1.907595
P	-1.187085	2.365653	4.339725
Si	-1.416142	0.393645	5.437482
C	-2.763440	-0.610636	4.604740
As	0.564778	3.337571	5.539718
C	0.586146	5.033529	4.539849
C	2.172316	2.572768	4.697683
C	-2.009170	2.898932	-0.637563
C	-3.619007	1.158815	1.338784
C	0.151043	-0.631358	5.612505
C	-1.984743	0.903357	7.154105
H	2.347953	-0.986083	1.757204
H	2.368877	0.790007	1.579581
H	2.143214	0.049849	3.180598
H	-2.936918	-1.531109	5.172025
H	-3.704079	-0.053794	4.576625
H	-2.493291	-0.889841	3.584211
H	-0.058826	-1.504012	6.240470
H	0.520088	-0.997461	4.652399
H	0.946518	-0.060263	6.099074
H	3.035358	3.162039	5.017102
H	2.312839	1.543133	5.029340
H	2.097537	2.603835	3.608897
H	-2.177173	0.011984	7.760403
H	-1.230013	1.504898	7.667478
H	-2.907533	1.487465	7.108089
H	1.433391	5.631173	4.883547
H	0.682783	4.851295	3.467345
H	-0.337084	5.579927	4.736897
H	-4.533042	1.379378	0.778029
H	-3.213496	0.215529	0.961175
H	-3.892933	1.008275	2.385961
H	0.604773	-0.860821	-0.480734
H	-0.964390	-0.044080	-0.537910

H	0.537577	0.917463	-0.463068
H	-4.009491	4.430817	1.345717
H	-3.390638	4.003893	2.955118
H	-2.400518	4.966775	1.852531
H	-2.917742	3.242421	-1.143308
H	-1.255647	3.685279	-0.736024
H	-1.646709	2.017138	-1.168509

[(Me3Ge)(MeO)P•]2

38

E= -5306.588087

Ge	-0.032013	0.293876	-0.171317
P	0.310429	0.116141	2.133316
P	2.497270	-0.028099	2.110251
Ge	2.878895	-2.326763	2.435148
O	-0.300330	-1.442784	2.376846
O	2.917316	0.335105	3.704569
C	1.027584	-0.936655	-1.264388
C	-1.936648	-0.070274	-0.429307
C	0.416725	2.138092	-0.646726
C	2.052297	-3.419423	1.045455
C	4.829204	-2.501097	2.390250
C	2.214259	-2.806848	4.208812
C	-1.116699	-1.623206	3.522648
C	3.117927	1.701475	4.015961
H	3.521163	1.745656	5.028570
H	3.827058	2.170930	3.323052
H	2.172972	2.257195	3.987092
H	-1.419637	-2.671585	3.536655
H	-0.570940	-1.397070	4.445672
H	-2.012596	-0.993655	3.478462
H	-2.189397	-0.045493	-1.492147
H	-2.180038	-1.059071	-0.035408
H	-2.544956	0.671858	0.091878
H	0.867107	-0.703281	-2.320529
H	2.093046	-0.834541	-1.047598
H	0.728551	-1.970487	-1.088498
H	0.222570	2.305282	-1.709331
H	-0.180140	2.846934	-0.069249
H	1.473844	2.332140	-0.453082
H	2.230851	-4.474584	1.269320
H	0.977505	-3.233368	1.040037
H	2.466763	-3.201498	0.060035
H	5.116275	-3.534913	2.600409
H	5.224043	-2.222645	1.410991
H	5.280702	-1.854881	3.145648
H	2.782651	-3.650303	4.608566
H	2.330519	-1.952686	4.877696
H	1.160862	-3.082594	4.154578

[(Me3Ge)(MeS)P•]2

38

E= -5952.583418

S	-0.336842	-0.214849	-0.558777
P	-0.030014	-0.190214	1.558984
P	2.130394	-0.121180	2.044682
S	2.468470	1.958103	2.427272
Ge	-0.463237	-2.436554	2.059023
Ge	3.410937	-0.221874	0.072600
C	3.072052	1.301794	-1.104629

C	5.255146	-0.164950	0.724202
C	3.092934	-1.902641	-0.869079
C	-2.316697	-2.773188	1.539233
C	0.742611	-3.682012	1.154488
C	-0.236030	-2.582424	3.995878
C	2.348780	1.968489	4.244500
C	-0.757366	1.539094	-0.823791
H	2.575595	2.984953	4.568861
H	1.339773	1.708095	4.564559
H	3.069820	1.278601	4.682682
H	-0.987611	1.651468	-1.884095
H	-1.628525	1.816466	-0.231237
H	0.084667	2.182280	-0.567339
H	3.764219	-1.947809	-1.731188
H	3.296445	-2.763786	-0.230832
H	2.064409	-1.954901	-1.227194
H	3.864292	1.353871	-1.856462
H	2.114222	1.180929	-1.611215
H	3.069504	2.238127	-0.543518
H	5.943375	-0.194902	-0.124866
H	5.433358	0.753303	1.286641
H	5.462890	-1.019541	1.371235
H	-0.440084	-3.607428	4.316159
H	0.786527	-2.325304	4.279588
H	-0.923055	-1.912746	4.517165
H	-2.589176	-3.802596	1.785844
H	-2.990815	-2.097723	2.069712
H	-2.446883	-2.622347	0.466140
H	0.465988	-4.707710	1.412695
H	0.687346	-3.570309	0.070446
H	1.772780	-3.512020	1.473476

[(Me$_3$Ge)(MeSe)P•]$_2$

38

E= -9959.350890

Ge	-0.117389	0.282955	-0.283890
P	0.071081	-0.016754	2.033309
P	2.277727	-0.183187	2.193311
Ge	2.961365	-2.426408	2.403853
Se	-0.949311	-2.038972	2.260344
Se	2.657117	0.410213	4.358105
C	0.756611	-1.123177	-1.325890
C	-2.029072	0.349730	-0.686657
C	0.738246	2.003491	-0.648862
C	2.442177	-3.450025	0.822917
C	4.911975	-2.299340	2.496902
C	2.273339	-3.276114	4.025920
C	-1.418920	-1.841516	4.151813
C	3.080538	2.290954	4.021101
H	3.355078	2.730595	4.980534
H	3.915924	2.363456	3.327198
H	2.205000	2.800306	3.622010
H	-1.993012	-2.725940	4.429738
H	-0.514682	-1.782941	4.755590
H	-2.023633	-0.947136	4.287704
H	-2.177025	0.541310	-1.752599
H	-2.509560	-0.595895	-0.429203
H	-2.510323	1.148560	-0.118888
H	0.598054	-0.939125	-2.391856
H	1.830979	-1.125155	-1.132440

H 0.355075 -2.108322 -1.081756
H 0.676852 2.229693 -1.716551
H 0.244407 2.804611 -0.095117
H 1.790047 1.973728 -0.357352
H 2.842231 -4.463174 0.920018
H 1.356876 -3.513346 0.739697
H 2.846888 -3.007471 -0.088566
H 5.339539 -3.298299 2.618363
H 5.313860 -1.857012 1.583127
H 5.212837 -1.683624 3.346451
H 2.762407 -4.245939 4.152993
H 2.484014 -2.669937 4.909289
H 1.197680 -3.433917 3.943675

[(Me$_3$Ge)(Me$_2$N)P•]$_2$

46

E= -5345.462987

N -0.814702 0.839380 1.045489
P -0.298426 -0.189694 2.334995
P 1.842165 0.113162 2.807074
N 1.843618 1.284432 4.078048
Ge -0.198484 -2.379270 1.525402
Ge 2.909628 1.353471 1.129599
C -0.502129 0.539785 -0.336945
C 1.053438 2.497143 3.952331
C 1.765680 0.721629 5.419223
C -2.129829 1.439296 1.207496
C 1.890020 2.865282 0.393867
C 4.509425 2.022933 2.036470
C 3.464145 0.140856 -0.308373
C -1.983985 -2.827564 0.846839
C 1.138721 -2.702459 0.127403
C 0.228514 -3.487876 3.080381
H -2.189290 2.361284 0.617622
H -2.946174 0.774947 0.880872
H -2.295396 1.692877 2.255466
H 4.114016 0.687898 -0.996937
H 4.029031 -0.699667 0.100517
H 2.623215 -0.256504 -0.878436
H 2.076862 2.957598 -0.679006
H 0.822065 2.720085 0.559368
H 2.201027 3.794511 0.877175
H 1.348077 3.208533 4.731015
H 1.220363 2.967778 2.982622
H -0.027438 2.306572 4.054083
H 5.071140 2.687797 1.375079
H 4.218685 2.570412 2.934863
H 5.155312 1.194377 2.334211
H 2.112582 1.463444 6.146609
H 0.740836 0.425276 5.695328
H 2.409394 -0.156376 5.489821
H 1.180357 -3.771383 -0.099234
H 0.893841 -2.167253 -0.792164
H 2.126799 -2.384541 0.465953
H -0.630319 1.437924 -0.951434
H 0.536170 0.221266 -0.432046
H -1.143394 -0.250384 -0.760936
H 0.262369 -4.541667 2.791324
H 1.201432 -3.204521 3.487089
H -0.524913 -3.363909 3.860906

H	-2.022137	-3.880590	0.555499
H	-2.737153	-2.654858	1.618589
H	-2.239189	-2.221999	-0.025630

[(Me3Ge)(Me2P)P•]2

46

E= -5918.737007

P	-0.451536	0.765943	0.142325
P	-0.139882	-0.253756	2.102833
P	1.938706	0.190061	2.740968
P	1.763882	1.628644	4.412067
Ge	0.193520	-2.494025	1.501109
Ge	3.135393	1.467069	1.187922
C	-1.887881	-0.221843	-0.486861
C	0.311748	2.702905	4.006933
C	0.953844	0.518820	5.653502
C	-1.386022	2.248619	0.743011
C	2.374938	3.221115	0.757858
C	4.863775	1.737584	2.068433
C	3.384506	0.411341	-0.437633
C	-1.554783	-3.345803	1.247100
C	1.293802	-2.680011	-0.107188
C	1.068910	-3.344672	3.027831
H	-1.767849	2.794492	-0.124332
H	-2.220483	1.978464	1.395067
H	-0.715684	2.913924	1.288893
H	3.994774	0.979829	-1.144815
H	3.902678	-0.522808	-0.210821
H	2.424562	0.187599	-0.904255
H	3.074543	3.756544	0.109496
H	1.425259	3.124954	0.230608
H	2.230934	3.815825	1.662222
H	0.064773	3.295571	4.892139
H	0.571567	3.393187	3.202556
H	-0.566380	2.120224	3.714719
H	5.524835	2.309456	1.411424
H	4.738225	2.285896	3.003999
H	5.335824	0.778160	2.289232
H	0.730585	1.097911	6.553412
H	0.025060	0.084329	5.272387
H	1.640061	-0.285746	5.923862
H	1.364378	-3.733022	-0.392214
H	0.874627	-2.121011	-0.946757
H	2.299734	-2.303274	0.085077
H	-2.366496	0.355802	-1.281903
H	-1.537496	-1.158565	-0.923207
H	-2.630983	-0.439330	0.285208
H	1.182717	-4.414307	2.832024
H	2.051965	-2.905760	3.202508
H	0.467235	-3.219595	3.930936
H	-1.417164	-4.428630	1.181956
H	-2.203912	-3.133791	2.099404
H	-2.054841	-3.008887	0.338454

[(Me3Ge)(Me2As)P•]2

46

E= -9707.834239

C	0.006285	0.018006	-0.102033
As	-0.024865	-0.083051	1.867489
C	1.931922	-0.046751	2.102678

P	-0.391795	2.204060	2.261820
Ge	-2.411584	2.590370	1.135888
C	-3.196912	4.205808	1.907614
P	-1.164767	2.420047	4.331542
Ge	-1.409092	0.384221	5.454537
C	-2.819495	-0.661157	4.593216
As	0.601668	3.345400	5.548000
C	0.664020	5.050410	4.564744
C	2.199588	2.555716	4.710024
C	-2.013298	2.948239	-0.750860
C	-3.653056	1.084374	1.302609
C	0.226944	-0.680670	5.641632
C	-2.000811	0.921472	7.243430
H	2.338512	-0.994204	1.740529
H	2.381362	0.780038	1.550418
H	2.175652	0.053697	3.160536
H	-2.988219	-1.578852	5.163302
H	-3.752226	-0.093232	4.572662
H	-2.542551	-0.930872	3.573415
H	0.012976	-1.550690	6.269025
H	0.590131	-1.037694	4.677122
H	1.012287	-0.097964	6.127866
H	3.072488	3.121729	5.044355
H	2.314390	1.519365	5.030396
H	2.136392	2.601089	3.621000
H	-2.185218	0.031724	7.851671
H	-1.240874	1.530385	7.737334
H	-2.924488	1.500565	7.183888
H	1.520691	5.627684	4.919760
H	0.763948	4.877533	3.491045
H	-0.249221	5.613481	4.761539
H	-4.568399	1.288776	0.740979
H	-3.213569	0.159935	0.920433
H	-3.915346	0.929994	2.350735
H	0.535579	-0.864128	-0.468910
H	-1.011914	-0.004895	-0.492030
H	0.516102	0.915488	-0.457217
H	-4.102639	4.469788	1.354880
H	-3.449016	4.050330	2.957185
H	-2.494245	5.039549	1.840923
H	-2.920946	3.301181	-1.248195
H	-1.252634	3.727771	-0.831698
H	-1.656912	2.061634	-1.276257

9) Phosphinyl radicals of the formula $(R'_3Si)(R_2N)P\bullet$ and their dimers $[(R'_3Si)(R_2N)P\bullet]_2$

$(Ph_3Si)(Ph_2N)P\bullet$

58

E= -1843.731219

C	0.161415	-0.604264	-2.397651
C	-0.562015	0.286019	-1.592533
C	-0.712863	1.600832	-2.038276
C	-0.177046	2.009964	-3.253737
C	0.530810	1.112436	-4.041139
C	0.703419	-0.198139	-3.608902
Si	-1.210932	-0.308869	0.065490
C	-2.202801	1.017424	0.964251
C	-3.237171	1.676590	0.287788
C	-4.031057	2.618174	0.929538

C	-3.817300	2.908602	2.272085
C	-2.814221	2.246816	2.968368
C	-2.017952	1.311145	2.317954
P	0.479428	-1.217501	1.298961
N	1.866221	-0.324080	0.794470
C	1.857936	1.030348	0.359678
C	2.551543	1.392012	-0.797189
C	2.517041	2.701827	-1.242050
C	1.788513	3.664804	-0.548420
C	1.105539	3.307241	0.604653
C	1.151150	1.997355	1.066627
C	-2.376317	-1.770456	-0.170664
C	-2.687986	-2.301101	-1.425894
C	-3.560651	-3.376658	-1.551362
C	-4.134987	-3.943815	-0.421485
C	-3.840494	-3.427582	0.835600
C	-2.973735	-2.350340	0.956208
C	3.135273	-0.961959	0.890053
C	4.232158	-0.265412	1.403783
C	5.465772	-0.888592	1.506203
C	5.622993	-2.212778	1.110190
C	4.533077	-2.906284	0.601593
C	3.296515	-2.285657	0.482159
H	3.092610	0.634556	-1.351075
H	3.042778	2.968780	-2.151478
H	1.755792	4.686732	-0.907974
H	0.538943	4.046530	1.159119
H	0.644088	1.723385	1.982208
H	-1.232213	2.325264	-1.420784
H	-0.302114	3.037040	-3.578365
H	0.952733	1.432735	-4.987456
H	1.261886	-0.902575	-4.215491
H	0.310375	-1.629304	-2.069307
H	-2.252553	-1.867052	-2.319958
H	-3.793074	-3.770729	-2.534696
H	-4.814001	-4.783694	-0.518959
H	-4.290125	-3.862419	1.721346
H	-2.767747	-1.949552	1.945295
H	-3.431997	1.448144	-0.756123
H	-4.821908	3.120758	0.383776
H	-4.437564	3.641597	2.775877
H	-2.652309	2.456832	4.019895
H	-1.244913	0.795582	2.880977
H	4.109121	0.763039	1.721918
H	6.308358	-0.337934	1.908934
H	6.588346	-2.697676	1.195854
H	4.643978	-3.936054	0.281498
H	2.450237	-2.820044	0.067856

[(Ph$_3$Si)(Ph$_2$N)P•]$_2$

128

E= -3244.850445

C	3.039121	-1.808398	2.921532
C	1.930102	-1.834056	2.069729
C	1.484359	-3.071132	1.609401
C	2.136940	-4.244076	1.959377
C	3.244740	-4.210506	2.795103
C	3.682575	-2.984211	3.279399
N	1.298941	-0.627610	1.627814
C	1.170960	0.449235	2.539961

C	1.040608	0.206372	3.913422
C	0.877308	1.254232	4.805226
C	0.835260	2.569353	4.359261
C	0.940173	2.812718	2.998256
C	1.091335	1.769057	2.097122
P	0.971728	-0.516822	-0.090242
P	-1.017082	0.159813	-0.706035
Si	-2.628210	-1.557498	-0.877174
C	-3.212545	-2.433287	0.761597
C	-3.910908	-3.780357	0.476099
N	-1.704427	1.543282	0.087714
C	-2.337981	1.558109	1.360582
C	-3.421097	2.415661	1.587754
C	-4.046734	2.446124	2.824022
C	-3.611028	1.627654	3.861397
C	-2.546960	0.768043	3.635229
C	-1.925824	0.729197	2.395369
C	-1.585545	2.809525	-0.566128
C	-1.938044	2.976863	-1.903282
C	-1.781449	4.209079	-2.523617
C	-1.281409	5.294874	-1.818443
C	-0.943346	5.136789	-0.478806
C	-1.093876	3.907135	0.142857
C	-4.126669	-0.676109	-1.772996
C	-4.612915	0.593698	-1.048940
C	-1.781086	-2.837556	-2.091943
C	-2.815269	-3.725718	-2.812446
C	-3.751331	-0.259453	-3.208183
C	-5.344889	-1.619431	-1.872177
C	-0.940852	-2.142289	-3.176999
C	-0.813581	-3.768665	-1.341516
C	-2.019406	-2.733295	1.687221
C	-4.230603	-1.574623	1.536744
Si	2.863852	0.363911	-1.257234
C	3.630558	-1.334115	-1.883612
C	2.570442	-2.319807	-2.407292
C	4.179969	1.322895	-0.164176
C	5.569520	1.278999	-0.838809
C	2.281701	1.489877	-2.730656
C	3.468470	2.095113	-3.507225
C	3.842950	2.813210	0.033966
C	4.346062	0.719089	1.239048
C	1.417440	0.709062	-3.735701
C	1.428513	2.659221	-2.217454
C	4.625288	-1.120968	-3.043371
C	4.360108	-2.070308	-0.742971
H	4.686408	-3.052570	-1.106067
H	5.251229	-1.546604	-0.395109
H	3.710633	-2.249981	0.117698
H	4.116636	-0.793823	-3.953337
H	5.416522	-0.405099	-2.821649
H	5.108345	-2.078380	-3.274676
H	1.889108	-2.638587	-1.619983
H	1.975715	-1.912595	-3.224609
H	3.080897	-3.212007	-2.790903
H	1.961402	-0.108131	-4.213623
H	0.515347	0.310380	-3.268978
H	1.094451	1.391708	-4.531777
H	4.140268	1.345149	-3.926564
H	3.071079	2.681640	-4.344933

H	4.060703	2.778289	-2.896187
H	1.007170	3.204896	-3.069664
H	0.590518	2.312924	-1.618874
H	1.994731	3.375625	-1.625662
H	5.216524	1.179470	1.723728
H	3.482270	0.930533	1.866069
H	4.508981	-0.357173	1.228104
H	3.937709	3.388036	-0.888425
H	2.844862	2.977344	0.439657
H	4.555582	3.237704	0.751671
H	6.001854	0.278158	-0.841688
H	5.564827	1.650468	-1.864615
H	6.250682	1.919446	-0.265096
H	-1.504558	-1.428533	-3.776094
H	-0.094960	-1.617684	-2.739794
H	-0.535885	-2.902444	-3.857127
H	-1.315556	-4.425210	-0.629753
H	-0.297150	-4.410126	-2.066122
H	-0.049592	-3.195035	-0.817285
H	-3.421825	-3.162579	-3.523500
H	-2.277645	-4.489889	-3.387264
H	-3.488077	-4.248005	-2.130536
H	-1.360333	-1.877776	1.833006
H	-2.387989	-3.038509	2.673797
H	-1.413097	-3.554909	1.306645
H	-4.423521	-2.043485	2.509310
H	-3.882667	-0.563359	1.732782
H	-5.188680	-1.511587	1.018287
H	-4.263520	-4.198882	1.426897
H	-4.781982	-3.676427	-0.173614
H	-3.242095	-4.518435	0.031280
H	-6.114154	-1.132874	-2.484614
H	-5.117826	-2.577771	-2.339624
H	-5.793475	-1.816266	-0.897390
H	-4.548462	0.371821	-3.619250
H	-2.820268	0.310395	-3.249815
H	-3.643696	-1.116600	-3.874992
H	-5.513831	0.962593	-1.555237
H	-4.871759	0.422302	-0.005184
H	-3.875070	1.391860	-1.077810
H	1.051500	-0.811033	4.282825
H	0.772445	1.034580	5.861989
H	0.709644	3.386555	5.058919
H	0.904220	3.830171	2.624363
H	1.118961	1.975289	1.035263
H	3.402785	-0.865750	3.306555
H	4.544557	-2.934250	3.935284
H	3.757263	-5.124984	3.069184
H	1.768187	-5.188759	1.575736
H	0.627173	-3.105879	0.957570
H	-3.769639	3.061999	0.791483
H	-4.885620	3.116341	2.975489
H	-4.098421	1.660526	4.828636
H	-2.186245	0.116279	4.422568
H	-1.114770	0.042751	2.228542
H	-2.308656	2.133237	-2.468803
H	-2.054762	4.316214	-3.567337
H	-1.158523	6.255391	-2.304930
H	-0.553251	5.975939	0.086480
H	-0.840832	3.784679	1.188273

(Ph$_3$Si)(PFP$_2$N)P•

58

E= -2836.029988

C	0.468303	-2.115541	-1.021748
C	0.996498	-1.135560	-0.186767
C	1.372977	-1.520356	1.098562
C	1.167576	-2.808651	1.556024
C	0.572203	-3.746598	0.727572
C	0.223547	-3.399530	-0.566552
N	1.154620	0.195909	-0.618729
C	2.471829	0.710457	-0.601580
C	3.471245	0.154616	-1.396285
C	4.764606	0.648697	-1.376508
C	5.071753	1.729770	-0.564392
C	4.090563	2.303450	0.230082
C	2.805407	1.787954	0.215778
F	3.184448	-0.869092	-2.193534
F	5.704469	0.105701	-2.140708
F	6.304934	2.214384	-0.546414
F	4.393631	3.330044	1.014946
F	1.890709	2.330689	1.007916
F	1.916313	-0.628624	1.921470
F	1.500411	-3.139643	2.797276
F	0.326591	-4.968775	1.179317
F	-0.331179	-4.298642	-1.371824
F	0.168356	-1.822906	-2.285747
P	-0.056871	1.194988	-1.367287
Si	-1.990855	0.764723	-0.201389
C	-1.742994	-0.341020	1.293066
C	-2.277513	-1.632309	1.344484
C	-2.105137	-2.431066	2.469464
C	-1.399013	-1.947702	3.563960
C	-0.863304	-0.665028	3.530869
C	-1.033126	0.129412	2.405094
C	-2.569554	2.462279	0.368491
C	-3.188491	2.633290	1.611767
C	-3.683291	3.872770	2.000919
C	-3.569423	4.965331	1.151150
C	-2.961352	4.814901	-0.089828
C	-2.467601	3.575837	-0.474761
C	-3.298594	0.045393	-1.342368
C	-4.590249	0.585016	-1.347984
C	-5.593960	0.029945	-2.132769
C	-5.324397	-1.076899	-2.927440
C	-4.047093	-1.624793	-2.938361
C	-3.045279	-1.065907	-2.155620
H	-3.286630	1.789609	2.287164
H	-4.158114	3.984102	2.969346
H	-3.952897	5.933072	1.454639
H	-2.869393	5.664304	-0.757255
H	-1.996501	3.482745	-1.449093
H	-4.815629	1.451964	-0.735295
H	-6.587373	0.464345	-2.124383
H	-6.106743	-1.509748	-3.540914
H	-3.828005	-2.484742	-3.561222
H	-2.053763	-1.501552	-2.190848
H	-2.842202	-2.016877	0.501032
H	-2.521862	-3.431910	2.490587
H	-1.259133	-2.571884	4.438886

H	-0.302360	-0.288277	4.378108
H	-0.601663	1.125385	2.388921

[(Ph$_3$Si)(PFP$_2$N)P•]$_2$

116

E= -5672.147297

C	-3.752816	2.627334	0.518368
C	-3.176846	2.446479	-0.747922
C	-4.030324	2.322263	-1.846257
C	-5.411499	2.371007	-1.687891
C	-5.963262	2.535045	-0.425389
C	-5.129521	2.667067	0.681011
Si	-1.309417	2.247011	-0.890455
C	-0.756709	2.274884	-2.674015
C	-1.194239	3.310016	-3.510865
C	-0.817205	3.354858	-4.846732
C	-0.001591	2.359006	-5.371410
C	0.438114	1.324920	-4.556342
C	0.065337	1.289511	-3.219495
H	-1.846985	4.085809	-3.120578
H	-1.165909	4.162870	-5.479930
H	0.286923	2.387853	-6.416003
H	1.071043	0.543134	-4.958433
H	0.405867	0.465756	-2.602084
H	-3.618830	2.159076	-2.835306
H	-6.056291	2.256254	-2.551358
H	-7.040068	2.554569	-0.300971
H	-5.554414	2.800076	1.669754
H	-3.117829	2.728905	1.393065
P	-1.161373	0.331383	0.332357
P	0.722086	-0.736718	0.514986
N	2.041342	-0.060027	-0.433469
C	2.486775	1.271333	-0.569403
C	2.923902	1.737212	-1.812915
C	3.399813	3.023334	-1.996159
C	3.499273	3.882433	-0.915331
C	3.119601	3.437256	0.337203
C	2.601079	2.165223	0.491943
F	2.928384	0.918864	-2.865387
F	3.776758	3.425446	-3.203281
F	3.960831	5.115838	-1.076297
F	3.276851	4.218901	1.397381
F	2.233625	1.805144	1.719453
N	-2.250738	-0.801389	-0.467832
C	-1.916870	-1.675831	-1.526449
C	-1.969057	-1.296187	-2.865185
C	-1.573907	-2.157444	-3.875906
C	-1.164418	-3.443908	-3.565940
C	-1.154546	-3.865362	-2.246257
C	-1.538212	-2.988165	-1.248358
F	-2.410796	-0.095862	-3.206580
F	-1.601010	-1.758322	-5.142872
F	-0.782200	-4.269521	-4.529268
F	-0.762447	-5.099064	-1.943620
F	-1.541903	-3.418756	0.008243
C	-3.502246	-0.985457	0.159943
C	-3.619043	-1.276922	1.519782
C	-4.846559	-1.292737	2.159852
C	-6.008595	-1.098344	1.432076
C	-5.928672	-0.890327	0.065975

C	-4.692635	-0.842930	-0.554512
F	-2.537214	-1.525472	2.251394
F	-4.662677	-0.645352	-1.867693
F	-7.037453	-0.709532	-0.643884
F	-7.189797	-1.121839	2.037555
F	-4.911869	-1.524491	3.467047
C	-0.432946	3.580406	0.093169
C	0.126328	4.680127	-0.569799
C	0.669753	5.742976	0.140872
C	0.672456	5.721224	1.529933
C	0.146712	4.627651	2.205347
C	-0.403178	3.570270	1.492887
H	0.146216	4.708568	-1.654217
H	1.100513	6.583488	-0.391296
H	1.105640	6.545893	2.084219
H	0.170095	4.583900	3.287741
H	-0.798214	2.724649	2.045243
Si	1.227948	-0.730071	2.766716
C	0.569641	0.679943	3.827511
C	1.364380	1.537501	4.593812
C	0.799648	2.534358	5.380985
C	-0.579051	2.694603	5.422020
C	-1.390117	1.839280	4.685519
C	-0.821829	0.840918	3.905641
H	2.440925	1.428561	4.598495
H	1.440962	3.183960	5.966173
H	-1.020351	3.472925	6.034762
H	-2.468972	1.942132	4.723949
H	-1.474163	0.176178	3.353633
C	0.317378	-2.279500	3.334581
C	-0.164193	-2.350248	4.648122
C	-0.795969	-3.492684	5.121010
C	-0.962702	-4.591989	4.287855
C	-0.489567	-4.544330	2.983396
C	0.145265	-3.401080	2.516121
H	-0.054440	-1.499097	5.311691
H	-1.164257	-3.521705	6.140372
H	-1.464006	-5.481241	4.653651
H	-0.623566	-5.392331	2.321414
H	0.483674	-3.376625	1.486961
C	3.083212	-1.061431	2.831244
C	3.509703	-2.374677	2.585432
C	4.857552	-2.710199	2.585431
C	5.814913	-1.743763	2.864319
C	5.415240	-0.440467	3.125000
C	4.069388	-0.101372	3.089297
H	2.780273	-3.155478	2.398813
H	5.158575	-3.730993	2.378370
H	6.866971	-2.004262	2.869610
H	6.155378	0.324115	3.332014
H	3.799438	0.935375	3.235386
C	2.880587	-1.061694	-0.980903
C	4.217968	-1.180740	-0.613237
C	5.006246	-2.223106	-1.072967
C	4.470920	-3.165964	-1.935107
C	3.155193	-3.041596	-2.356323
C	2.392570	-1.981806	-1.904252
F	4.764921	-0.278233	0.190088
F	6.268525	-2.329074	-0.674912
F	5.216320	-4.173994	-2.367042

F	2.639737	-3.916906	-3.212260
F	1.156712	-1.845395	-2.378711

(Ph3Si)(t*B*u2N)P•

62

E= -1696.154660

C	0.641202	-0.922806	2.192556
C	-0.476877	-1.170054	1.384746
C	-1.207670	-2.338809	1.624574
C	-0.836920	-3.225295	2.629750
C	0.273937	-2.960072	3.419726
C	1.012384	-1.802730	3.200489
Si	-0.922201	-0.029707	-0.049372
P	0.700575	-0.385237	-1.633864
N	2.220819	0.029147	-0.937893
C	3.207598	-1.102064	-0.810296
C	2.464093	-2.443972	-0.633986
C	-2.486254	-0.669075	-0.885512
C	-2.448838	-1.811883	-1.696748
C	-3.602839	-2.315048	-2.284223
C	-4.823048	-1.684904	-2.071618
C	-4.882935	-0.552741	-1.268900
C	-3.726176	-0.051745	-0.683569
C	-1.260955	1.713404	0.577280
C	-1.126073	2.070270	1.922145
C	-1.383438	3.368736	2.349475
C	-1.790094	4.334385	1.438560
C	-1.946165	3.996356	0.098472
C	-1.684431	2.700370	-0.323080
C	2.619681	1.469134	-0.768619
C	2.538859	1.910707	0.702628
C	4.018115	1.783989	-1.333798
C	1.673971	2.381541	-1.570816
C	4.095087	-1.252807	-2.061394
C	4.066696	-0.963762	0.458689
H	4.200527	2.854759	-1.212050
H	4.832984	1.263975	-0.835449
H	4.060740	1.558482	-2.400988
H	2.010525	3.416618	-1.467617
H	1.683831	2.126128	-2.633526
H	0.650123	2.345175	-1.205875
H	2.845154	2.956751	0.798058
H	1.510429	1.843554	1.062017
H	3.179304	1.313314	1.350989
H	4.630779	-2.205934	-2.023329
H	3.470539	-1.253367	-2.958827
H	4.837812	-0.463417	-2.158144
H	3.201417	-3.217436	-0.401376
H	1.748126	-2.402727	0.189339
H	1.939688	-2.748991	-1.540061
H	4.751062	-1.814886	0.509868
H	4.673468	-0.061686	0.492592
H	3.432086	-0.990617	1.347167
H	-0.820409	1.325934	2.650048
H	-1.269765	3.623990	3.397282
H	-1.990513	5.346875	1.771010
H	-2.271599	4.743293	-0.617074
H	-1.813270	2.450711	-1.373045
H	-2.078562	-2.563209	1.017595
H	-1.418904	-4.125212	2.795647

H	0.564299	-3.651699	4.202790
H	1.882633	-1.588106	3.811464
H	1.243659	-0.038139	2.023813
H	-3.791529	0.833338	-0.059140
H	-5.832611	-0.058193	-1.096639
H	-5.724771	-2.076035	-2.529878
H	-3.548785	-3.199222	-2.909718
H	-1.505454	-2.320345	-1.871712

[(Ph$_3$Si)(t*B*u$_2$N)P•]$_2$

124

E= -3392.391979

C	2.487257	-2.766912	-1.178657
C	3.181142	-2.016885	-0.229771
C	4.454972	-2.462124	0.150805
C	5.007188	-3.613451	-0.395511
C	4.300354	-4.339727	-1.347426
C	3.038641	-3.912759	-1.739503
Si	2.512565	-0.431850	0.550958
P	0.738327	0.764115	-0.247882
P	-1.087980	-0.259125	-1.097836
N	-1.059080	-1.966157	-1.426210
C	-1.329688	-2.408357	-2.855129
C	-2.609266	-3.260888	-2.994083
C	2.130559	-0.758337	2.372478
C	1.674100	0.278188	3.196948
C	1.542635	0.105288	4.568723
C	1.835063	-1.125519	5.144767
C	2.253027	-2.177569	4.340562
C	2.406739	-1.991005	2.971482
C	3.987596	0.756376	0.515281
C	4.293863	1.610009	1.579862
C	5.390448	2.463686	1.531484
C	6.211431	2.481278	0.412304
C	5.937614	1.628638	-0.650798
C	4.843470	0.776264	-0.593378
N	1.363909	1.884887	-1.408587
C	1.615329	1.521080	-2.843966
C	1.940415	0.027206	-2.950362
C	1.435304	3.338057	-0.974175
C	0.601921	4.264040	-1.876403
C	0.900453	3.526404	0.456318
C	2.886852	3.865475	-0.923447
Si	-2.713725	0.391412	0.398515
C	-4.409017	0.192624	-0.415498
C	-5.517479	0.746096	0.240483
C	-6.788007	0.681637	-0.314364
C	-6.980000	0.067418	-1.547762
C	-5.893732	-0.473466	-2.221015
C	-4.625086	-0.405382	-1.655533
C	-2.813217	-0.365700	2.123204
C	-3.915234	-1.126469	2.527478
C	-3.975057	-1.692365	3.795129
C	-2.927414	-1.509810	4.688121
C	-1.822888	-0.757767	4.307486
C	-1.771362	-0.191573	3.041190
C	-2.542456	2.259873	0.568830
C	-2.442901	2.900832	1.807422
C	-2.431657	4.288371	1.897967
C	-2.529305	5.062208	0.749444

C	-2.645853	4.442826	-0.489289	H	0.110192	-4.088424	-2.871991
C	-2.652165	3.057685	-0.576188	H	-1.694054	-1.621888	-4.811533
C	-1.208057	-2.968845	-0.298331	H	-2.349426	-0.570855	-3.553053
C	-0.383339	-2.505537	0.907865	H	-0.620954	-0.599507	-3.866528
C	-2.686328	-3.146345	0.114951	H	-2.811252	-3.417251	-4.056811
C	-0.650587	-4.376438	-0.595286	H	-2.524504	-4.246331	-2.541139
C	-1.512172	-1.218050	-3.812124	H	-3.476188	-2.765704	-2.557914
C	-0.140427	-3.198380	-3.441411	H	2.902923	4.795860	-0.348644
C	0.387283	1.806964	-3.740469	H	3.536799	3.151838	-0.423569
C	2.832504	2.232077	-3.461602	H	3.307222	4.090875	-1.899729
H	2.116062	-0.230708	-3.997597	H	0.931829	4.594370	0.686031
H	2.840927	-0.225268	-2.391309	H	-0.123229	3.183811	0.572815
H	1.133424	-0.602840	-2.586879	H	1.524477	3.009603	1.188811
H	3.014295	1.798719	-4.448992	H	0.550779	5.256520	-1.420483
H	2.671661	3.298376	-3.612217	H	1.041243	4.390385	-2.866073
H	3.727210	2.089035	-2.853779	H	-0.415261	3.889686	-1.984578
H	0.490702	1.274958	-4.691662	H	-4.738623	-1.292287	1.841179
H	-0.529081	1.474866	-3.253018	H	-4.840062	-2.280162	4.082392
H	0.286421	2.864303	-3.976307	H	-2.968620	-1.955319	5.676052
H	-0.811827	-4.977780	0.303364	H	-0.992245	-0.622178	4.988882
H	-1.151000	-4.893808	-1.410502	H	-0.890848	0.375611	2.755699
H	0.422197	-4.362124	-0.784874	H	-5.384725	1.241336	1.198165
H	-0.559035	-3.173254	1.754089	H	-7.630009	1.116252	0.213052
H	0.678218	-2.549778	0.672979	H	-7.971989	0.018215	-1.982976
H	-0.623429	-1.504506	1.247726	H	-6.029502	-0.946213	-3.187726
H	-2.752724	-3.480858	1.152865	H	-3.786155	-0.821828	-2.194679
H	-3.249671	-2.219031	0.035351	H	-2.379080	2.314825	2.717241
H	-3.195002	-3.883898	-0.506303	H	-2.349256	4.764293	2.868975
H	-0.389618	-3.519052	-4.457200	H	-2.521843	6.144395	0.818301
H	0.747930	-2.568147	-3.505811	H	-2.727879	5.041047	-1.390009

H	-2.748935	2.588073	-1.551045
H	3.678781	1.608731	2.471766
H	5.602164	3.115342	2.371925
H	7.064885	3.148814	0.370884
H	6.579316	1.624390	-1.525056
H	4.667102	0.103972	-1.425931
H	5.029173	-1.896086	0.878146
H	5.993253	-3.939791	-0.083834
H	4.733795	-5.233377	-1.782736
H	2.480156	-4.470200	-2.484043
H	1.496429	-2.450734	-1.480219
H	2.748948	-2.821043	2.361697
H	2.469702	-3.144749	4.780549
H	1.730121	-1.265431	6.215154
H	1.201584	0.929453	5.186310
H	1.412556	1.236993	2.758468

(Ph_3Si)(TMS_2N)P•

62

E= -2199.095163

C	-1.896728	2.702380	-0.372852
C	-1.440644	1.744435	0.543286
C	-1.195916	2.158146	1.855713
C	-1.377376	3.484205	2.235382
C	-1.819262	4.419712	1.309502
C	-2.086650	4.024846	0.002914
Si	-1.189721	-0.026827	-0.039309
P	0.376829	-0.224607	-1.703126
N	1.884396	-0.009470	-0.873860
Si	2.550321	1.610948	-0.617178
C	4.300263	1.709312	-1.299533
C	-0.680757	-1.142440	1.385172
C	0.462578	-0.865645	2.145872
C	0.883315	-1.727018	3.150603
C	0.172802	-2.893289	3.409812
C	-0.963653	-3.185928	2.666434
C	-1.386730	-2.317089	1.667043
C	-2.791358	-0.654663	-0.801487
C	-4.020179	-0.035825	-0.545920
C	-5.200527	-0.536406	-1.082138
C	-5.174367	-1.668276	-1.887534
C	-3.964741	-2.298448	-2.154097
C	-2.787037	-1.794729	-1.616276
Si	2.881251	-1.470018	-0.715946
C	3.940382	-1.713736	-2.246558
C	3.970207	-1.318999	0.807060
C	1.808538	-2.993382	-0.508871
C	1.535534	2.885840	-1.538928
C	2.583737	2.072506	1.201916
H	4.681973	2.724338	-1.147572
H	5.004370	1.021703	-0.826074
H	4.305457	1.509840	-2.374850
H	2.035406	3.856625	-1.453130
H	1.448300	2.648148	-2.602845
H	0.531222	2.998809	-1.126668
H	3.117860	3.019651	1.331274
H	1.571353	2.214245	1.590798
H	3.090618	1.319393	1.811427
H	4.475319	-2.667705	-2.197718
H	3.312395	-1.729452	-3.142685

H	4.679711	-0.918954	-2.369169
H	2.460987	-3.863132	-0.375165
H	1.156866	-2.925352	0.365093
H	1.186182	-3.182205	-1.387957
H	4.608903	-2.205594	0.876509
H	4.623951	-0.444251	0.800001
H	3.357109	-1.281077	1.710810
H	-0.864561	1.438278	2.596748
H	-1.176672	3.784419	3.257870
H	-1.960631	5.453375	1.604913
H	-2.438389	4.748862	-0.723550
H	-2.104369	2.410213	-1.398757
H	-2.276308	-2.561059	1.095553
H	-1.523665	-4.093047	2.864875
H	0.503860	-3.570812	4.188965
H	1.771021	-1.491482	3.727967
H	1.046131	0.023244	1.938116
H	-4.056540	0.850064	0.080120
H	-6.142211	-0.041946	-0.870592
H	-6.094816	-2.058460	-2.307655
H	-3.937886	-3.180922	-2.783596
H	-1.850625	-2.300844	-1.834166

[(Ph$_3$Si)(TMS$_2$N)P•]$_2$

124

E= -4398.277199

C	2.018649	0.302663	3.295657
C	2.243330	-0.839736	2.517777
C	2.453777	-2.049981	3.184286
C	2.444842	-2.118315	4.572547
C	2.245536	-0.968577	5.325785
C	2.034232	0.245737	4.683236
Si	2.450961	-0.682507	0.647773
C	4.060100	0.282514	0.393441
C	4.743550	0.901467	1.444551
C	5.983271	1.501793	1.245200
C	6.567497	1.495176	-0.013504
C	5.907645	0.880711	-1.071590
C	4.673671	0.282104	-0.864788
P	0.765285	0.707832	-0.042978
N	1.449364	1.715694	-1.281350
Si	1.855466	3.380910	-0.784460
C	1.239263	3.761318	0.940486
P	-1.141156	-0.086304	-0.938205
Si	-2.631709	0.760730	0.585236
C	-2.602879	0.129297	2.357737
C	-3.653345	-0.610274	2.909847
C	-3.589566	-1.093063	4.211729
C	-2.470210	-0.838407	4.992218
C	-1.420626	-0.093441	4.468121
C	-1.488812	0.385911	3.167910
N	-1.390305	-1.795245	-1.161853
Si	-1.702308	-2.931805	0.183832
C	-3.534406	-2.966868	0.607148
Si	-1.805281	-2.350774	-2.809543
C	-1.895457	-0.947808	-4.046397
C	-0.554477	-3.581427	-3.480115
C	-3.481358	-3.210278	-2.810904
C	-1.272617	-4.704542	-0.280379
C	-0.621433	-2.524322	1.650655

C	-2.333408	2.619995	0.603721
C	-2.390672	3.349975	1.795809
C	-2.311413	4.738119	1.792651
C	-2.182775	5.424201	0.592903
C	-2.136729	4.716626	-0.602541
C	-2.207898	3.330179	-0.596330
C	-4.367348	0.557928	-0.127640
C	-5.459223	1.042729	0.604340
C	-6.751564	0.970205	0.101708
C	-6.980959	0.417485	-1.153393
C	-5.910748	-0.051246	-1.903434
C	-4.620588	0.024044	-1.391979
Si	1.587102	1.282752	-2.991286
C	0.132259	1.958610	-3.971259
C	3.142503	2.005590	-3.764902
C	1.689510	-0.575284	-3.209542
C	2.815276	-2.377342	-0.090845
C	4.074696	-2.933502	0.180733
C	4.438081	-4.175092	-0.321623
C	3.551213	-4.883698	-1.123748
C	2.303476	-4.347055	-1.408956
C	1.937260	-3.109234	-0.890095
C	3.701863	3.704708	-0.770392
C	1.104575	4.612777	-1.994170
H	1.631900	-0.810976	-4.277394
H	2.620312	-0.999902	-2.828164
H	0.873293	-1.094913	-2.711266
H	3.207017	1.641839	-4.796309
H	3.133348	3.097051	-3.807550
H	4.047944	1.694902	-3.239536
H	0.108966	1.529701	-4.978587
H	-0.812232	1.722969	-3.477142
H	0.203858	3.043096	-4.078304
H	-1.502789	-5.311349	0.603213
H	-1.857190	-5.112095	-1.107026
H	-0.211931	-4.839951	-0.496463
H	-1.111511	-2.821529	2.582812
H	0.319325	-3.074417	1.572215
H	-0.384303	-1.467552	1.756184
H	-3.671831	-3.247474	1.655421
H	-4.037627	-2.013510	0.445526
H	-4.044691	-3.710412	-0.010943
H	-0.853486	-3.856629	-4.497360
H	0.453951	-3.165748	-3.533476
H	-0.517291	-4.499094	-2.890600
H	-2.266001	-1.371809	-4.986433
H	-2.561125	-0.131882	-3.759653
H	-0.914059	-0.517122	-4.242354
H	-3.770507	-3.394287	-3.851149
H	-3.468171	-4.177951	-2.304895
H	-4.262756	-2.604175	-2.347007
H	3.861979	4.764533	-0.540818
H	4.204141	3.118230	0.000348
H	4.189874	3.494120	-1.722757
H	1.396281	4.829980	1.124271
H	0.180954	3.542669	1.079086
H	1.806882	3.209990	1.694745
H	1.181445	5.617050	-1.564639
H	1.627330	4.628244	-2.954249
H	0.050244	4.408145	-2.180132

H	-4.536599	-0.821858	2.319006
H	-4.416297	-1.668428	4.613917
H	-2.414295	-1.217714	6.006678
H	-0.539202	0.100340	5.065933
H	-0.652685	0.952229	2.767585
H	-5.298875	1.489376	1.581355
H	-7.581028	1.349677	0.688128
H	-7.989698	0.359910	-1.546899
H	-6.076880	-0.475195	-2.887883
H	-3.797723	-0.340468	-1.992709
H	-2.497746	2.831807	2.742554
H	-2.351230	5.282502	2.729672
H	-2.123702	6.506890	0.586556
H	-2.046433	5.249233	-1.542525
H	-2.169473	2.794262	-1.540356
H	4.318419	0.904601	2.441454
H	6.492764	1.971938	2.079061
H	7.533196	1.962542	-0.170176
H	6.357823	0.863708	-2.058288
H	4.191438	-0.214986	-1.698519
H	4.787904	-2.382304	0.786173
H	5.416583	-4.584263	-0.095992
H	3.834300	-5.848666	-1.529427
H	1.613924	-4.890817	-2.044254
H	0.958442	-2.704293	-1.121091
H	2.629664	-2.955900	2.613089
H	2.603750	-3.070510	5.066452
H	2.252878	-1.018300	6.409107
H	1.874453	1.149215	5.262230
H	1.834830	1.255179	2.807979

$(Ph_3Si)(PFS_2N)P$•

62

E= -3985.374706

C	3.151086	2.684578	-0.886502
C	2.970758	1.837105	0.213301
C	3.288829	2.324917	1.484075
C	3.761070	3.621166	1.651675
C	3.920413	4.451895	0.550232
C	3.616678	3.981000	-0.721991
Si	2.368731	0.079551	-0.055086
C	3.671284	-0.830469	-1.053611
C	5.018580	-0.526400	-0.816111
C	6.031890	-1.185347	-1.498931
C	5.716182	-2.153004	-2.445577
C	4.386503	-2.458738	-2.704946
C	3.374422	-1.803795	-2.013884
P	0.552147	0.269927	-1.470679
N	-0.898436	0.022630	-0.400586
Si	-1.798706	1.499234	-0.290981
C	-0.697006	3.048050	0.099381
F	0.167151	2.804153	1.099960
C	1.998121	-0.737483	1.582154
C	2.649725	-1.920216	1.949733
C	2.453458	-2.478931	3.204954
C	1.604061	-1.862380	4.115878
C	0.947017	-0.689956	3.767625
C	1.140596	-0.133670	2.510012
Si	-1.471198	-1.591572	-0.144490
C	-3.071093	-1.929513	-1.196838

F	-4.174895	-1.333697	-0.731986
C	-0.271133	-2.957788	-0.830199
F	-0.151765	-2.885039	-2.170977
C	-1.792005	-2.107687	1.702209
F	-0.815786	-2.915717	2.142255
F	0.968641	-2.919901	-0.323282
F	-0.777110	-4.165121	-0.541045
C	-2.781739	1.929957	-1.907434
F	-2.130147	1.565217	-3.018988
C	-3.084924	1.507439	1.158387
F	-3.762655	0.353608	1.272800
F	-3.976183	1.314857	-1.920420
F	-3.011171	3.251145	-1.988788
F	-2.477783	1.736112	2.334714
F	-3.987109	2.481221	0.975094
F	0.009828	3.479866	-0.953214
F	-1.481870	4.063263	0.495619
F	-1.859280	-1.067780	2.542064
F	-2.947530	-2.785261	1.805639
F	-2.866777	-1.472156	-2.447993
F	-3.333241	-3.241136	-1.287872
H	5.279064	0.241187	-0.093714
H	7.068183	-0.938549	-1.297608
H	6.506170	-2.664364	-2.984143
H	4.134495	-3.207172	-3.447566
H	2.345094	-2.066112	-2.229197
H	3.316826	-2.407951	1.246531
H	2.964183	-3.396729	3.473062
H	1.449370	-2.300013	5.095560
H	0.273521	-0.212470	4.469329
H	0.627932	0.787920	2.258669
H	3.163042	1.691014	2.355248
H	4.001588	3.983127	2.644933
H	4.282077	5.465545	0.681705
H	3.740174	4.625068	-1.585045
H	2.924056	2.334099	-1.889442

[(Ph$_3$Si)(PFS$_2$N)P•]$_2$

124

E= -7970.796026

C	-2.474811	-3.639843	-1.612159
F	-3.661230	-4.265521	-1.624313
Si	-2.724096	-1.678462	-1.647609
C	-1.904035	-1.200267	-3.349205
F	-0.657516	-1.654636	-3.482272
F	-1.825697	-4.024585	-2.723137
F	-1.789911	-4.129820	-0.573015
N	-2.341146	-0.668305	-0.251231
P	-0.893928	0.442589	-0.587146
P	1.033838	0.360639	0.526367
Si	1.341453	-0.891818	2.490373
C	3.180796	-1.173954	2.710919
C	-4.606383	-1.481693	-2.108995
F	-4.829069	-0.281529	-2.668202
Si	-3.447757	-0.529427	1.107809
C	-4.910375	0.767743	1.000271
F	-5.401822	0.989379	-0.213974
C	-4.293753	-2.235843	1.590376
F	-3.830461	-3.224644	0.791213
C	-2.602828	0.019611	2.762448

F	-1.735671	1.024782	2.568106
F	-3.989973	-2.580925	2.848016
F	-5.621309	-2.252213	1.496250
F	-5.041094	-2.400866	-2.971175
F	-5.395672	-1.563012	-1.013344
F	-2.643058	-1.796834	-4.304345
F	-1.889224	0.107968	-3.642956
Si	-1.403111	2.737699	-0.396403
C	-1.050200	3.666606	1.201365
C	-0.327446	3.412090	-1.785001
C	-3.147389	3.062331	-0.999987
N	2.397835	-0.121906	-0.653130
Si	3.681453	1.080705	-0.643634
C	3.349727	2.827470	0.142038
F	2.894535	2.842614	1.401255
Si	2.495366	-1.519607	-1.690137
C	2.247790	-1.187459	-3.602862
F	3.425705	-0.916730	-4.188625
C	4.174169	-2.508939	-1.712287
F	4.422388	-3.157973	-0.567309
C	1.246420	-2.892944	-1.156594
F	0.011273	-2.367897	-0.953696
F	4.070112	-3.444822	-2.676926
F	5.247442	-1.763609	-1.997630
C	0.528115	-2.566495	2.725126
C	0.810488	0.305744	3.834917
F	-3.540603	0.457666	3.620455
F	-1.945454	-0.964768	3.374556
F	-5.923832	0.367397	1.784440
F	-4.506151	1.957471	1.495020
C	5.337032	0.584295	0.264153
F	5.471848	-0.726720	0.473064
C	4.288008	1.600381	-2.430773
F	3.258488	1.709622	-3.284150
F	6.399100	0.987621	-0.454092
F	5.426671	1.196899	1.457167
F	4.894690	2.794800	-2.395692
F	5.160840	0.735813	-2.962215
F	4.518400	3.491208	0.159917
F	2.506247	3.544019	-0.617112
F	1.121815	-3.863710	-2.054464
F	1.616147	-3.459924	-0.000603
F	1.759121	-2.272207	-4.217933
F	1.428018	-0.161959	-3.867944
C	0.344446	-0.219311	5.045976
C	0.083597	0.606148	6.130770
C	0.292002	1.975569	6.028132
C	0.751256	2.513130	4.833630
C	1.001070	1.687703	3.745255
H	0.164593	-1.283750	5.144380
H	-0.283882	0.177704	7.056414
H	0.093030	2.622026	6.875681
H	0.909706	3.579435	4.738880
H	1.335898	2.137275	2.819618
C	3.936164	-0.317301	3.519158
C	5.230505	-0.651416	3.896837
C	5.790406	-1.849672	3.477057
C	5.056782	-2.712163	2.671301
C	3.765729	-2.376979	2.294942
H	3.506333	0.611739	3.876690

H	5.799696	0.026788	4.521783
H	6.798866	-2.112264	3.776330
H	5.489783	-3.646636	2.334288
H	3.198307	-3.075822	1.694206
C	1.061380	-3.358184	3.753524
C	0.489881	-4.576907	4.093516
C	-0.623593	-5.035608	3.403249
C	-1.159949	-4.271417	2.376313
C	-0.589252	-3.050111	2.046651
H	1.939125	-3.025037	4.297344
H	0.920049	-5.167999	4.893988
H	-1.071173	-5.988793	3.660801
H	-2.022120	-4.625901	1.828517
H	-1.016933	-2.477492	1.233254
C	-0.245088	4.809424	-1.883730
C	0.376341	5.415650	-2.964236
C	0.916799	4.637985	-3.982616
C	0.825949	3.256756	-3.912332
C	0.211321	2.650266	-2.822563
H	-0.677889	5.437589	-1.111681
H	0.432407	6.497133	-3.015917
H	1.400616	5.110005	-4.830198
H	1.239319	2.640350	-4.700820
H	0.143802	1.570525	-2.802484
C	0.134108	4.402389	1.316278
C	0.364770	5.241890	2.399314
C	-0.592117	5.368112	3.395938
C	-1.766492	4.629919	3.315717
C	-1.989962	3.791734	2.233718
H	0.881691	4.342240	0.540730
H	1.291134	5.802634	2.453871
H	-0.422134	6.032435	4.236304
H	-2.514360	4.707294	4.096411
H	-2.913496	3.233472	2.201524
C	-3.948597	4.064694	-0.441142
C	-5.148742	4.431660	-1.033908
C	-5.568386	3.811007	-2.203449
C	-4.774688	2.832777	-2.786128
C	-3.574476	2.471297	-2.192098
H	-3.638855	4.572814	0.463752
H	-5.756261	5.206908	-0.580902
H	-6.507448	4.096121	-2.664234
H	-5.088175	2.347822	-3.702802
H	-2.958182	1.728741	-2.681105

(PFP_3Si)(Ph_2N)P•

58

E= -3332.205929

C	4.506424	-0.977733	-2.391773
C	3.244241	-1.385291	-1.949394
C	2.955177	-2.749408	-1.874641
C	3.903236	-3.685809	-2.261172
C	5.155607	-3.280384	-2.702738
C	5.451413	-1.922943	-2.758978
N	2.262777	-0.427513	-1.579536
C	2.734934	0.828313	-1.088663
C	3.366872	0.893641	0.151273
C	3.853997	2.108127	0.610994
C	3.720870	3.256924	-0.163707
C	3.094359	3.187845	-1.401144

C	2.602160	1.974549	-1.866216
P	0.631794	-0.717215	-2.022928
Si	-0.580477	-0.086910	-0.187515
C	-0.738661	-1.610254	0.935351
C	-1.937468	-2.151874	1.382586
C	-1.994037	-3.313431	2.139257
C	-0.820729	-3.964959	2.479480
C	0.398969	-3.447441	2.068920
C	0.412908	-2.289373	1.316280
F	-3.104855	-1.563014	1.111945
F	-3.161127	-3.799481	2.544024
F	-0.861262	-5.072688	3.202461
F	1.528218	-4.058819	2.404690
F	1.608973	-1.802244	0.965059
C	0.069297	1.374918	0.812992
C	0.502130	1.332268	2.131310
C	1.000290	2.449008	2.784344
C	1.068895	3.657411	2.114092
C	0.631628	3.744739	0.802417
C	0.145847	2.611282	0.183440
F	0.466387	0.198554	2.835862
F	1.427665	2.363544	4.039737
F	1.575569	4.724053	2.714229
F	0.715815	4.897779	0.149650
F	-0.268953	2.727116	-1.082868
C	-2.312843	0.322808	-0.842807
C	-3.098507	1.389043	-0.422748
C	-4.357076	1.639364	-0.946347
C	-4.877375	0.791391	-1.910068
C	-4.138649	-0.300662	-2.337381
C	-2.884981	-0.513062	-1.794008
F	-2.678379	2.219304	0.534671
F	-5.071511	2.675127	-0.522724
F	-6.080214	1.017560	-2.413676
F	-4.640111	-1.129398	-3.244229
F	-2.219192	-1.602527	-2.197250
H	3.464655	-0.008337	0.743000
H	4.335733	2.159976	1.580788
H	4.097765	4.205008	0.201990
H	2.983109	4.079629	-2.005990
H	2.120105	1.905442	-2.834239
H	1.993294	-3.071370	-1.496266
H	3.663289	-4.740844	-2.196965
H	5.897633	-4.014392	-2.993438
H	6.425450	-1.592862	-3.101602
H	4.742521	0.077467	-2.451335

[(PFP$_3$Si)(Ph$_2$N)P•]$_2$

116

E= -6664.493595

C	3.241658	1.157486	2.366133
C	3.498811	0.839989	1.034109
C	4.228825	1.794884	0.333692
C	4.654203	2.989038	0.889052
C	4.353460	3.267191	2.210021
C	3.649954	2.339097	2.958448
Si	2.559553	-0.594766	0.197652
C	3.486031	-1.147106	-1.352086
C	4.875559	-1.178178	-1.409428
C	5.565975	-1.536088	-2.552533

C	4.856207	-1.891181	-3.691539	C	2.310979	1.320526	-4.537734
C	3.472422	-1.891607	-3.670153	C	1.178328	1.698662	-5.246230
C	2.819810	-1.524259	-2.506286	C	0.074157	2.188169	-4.559210
F	5.597349	-0.859617	-0.335711	C	0.092305	2.265193	-3.173645
F	6.892597	-1.551214	-2.570710	H	3.227390	1.156362	-2.606063
F	5.503250	-2.227710	-4.795745	H	3.189693	0.966183	-5.064876
F	2.784686	-2.210904	-4.759470	H	1.162996	1.628727	-6.327692
F	1.489269	-1.497169	-2.545714	H	-0.810603	2.505856	-5.098416
F	4.531605	1.617179	-0.957570	H	-0.765992	2.647101	-2.645496
F	5.324005	3.870721	0.159013	C	1.419833	3.201030	-0.433632
F	4.727572	4.413443	2.752894	C	1.032720	3.492912	0.880110
F	3.345264	2.602620	4.223591	C	1.320106	4.727395	1.444816
F	2.528748	0.326107	3.130592	C	1.995464	5.702536	0.723714
P	0.652746	0.644687	0.031138	C	2.369426	5.422633	-0.583545
P	-1.166162	-0.064258	-0.978275	C	2.088368	4.192289	-1.160761
N	-1.154698	-1.707884	-1.424684	H	0.514215	2.749027	1.469611
C	-0.750882	-2.818000	-0.625037	H	2.403189	3.997044	-2.177128
C	0.133023	-3.755841	-1.165090	H	2.900060	6.165442	-1.167823
C	0.507082	-4.872227	-0.432082	H	2.223429	6.661659	1.171341
C	-0.003931	-5.080179	0.843437	H	1.007297	4.921175	2.465135
C	-0.883838	-4.150443	1.381300	C	2.448709	-2.093508	1.357579
C	-1.246939	-3.022453	0.658800	C	3.375946	-3.133753	1.363052
H	0.505240	-3.612613	-2.170696	C	3.331398	-4.175454	2.275982
H	1.195387	-5.588210	-0.866675	C	2.339701	-4.199345	3.241068
H	0.279012	-5.958677	1.412020	C	1.397330	-3.188220	3.271443
H	-1.294522	-4.298839	2.373339	C	1.459245	-2.182470	2.327391
H	-1.930831	-2.310345	1.093776	F	4.375750	-3.175582	0.482298
N	1.196424	1.922663	-1.019404	F	4.232441	-5.149864	2.229303
C	1.209877	1.846574	-2.456027	F	2.272400	-5.200381	4.103709
C	2.332061	1.409242	-3.151085	F	0.409100	-3.222700	4.160442

F	0.496214	-1.265703	2.368753
Si	-2.870238	0.481514	0.476050
C	-2.957478	-0.279723	2.205659
C	-3.954957	-1.142725	2.650809
C	-3.869376	-1.816639	3.859523
C	-2.772211	-1.613236	4.680929
C	-1.781198	-0.723224	4.298876
C	-1.897843	-0.075814	3.084548
F	-5.053173	-1.361860	1.926642
F	-4.834448	-2.645496	4.239621
F	-2.679777	-2.254724	5.833512
F	-0.741011	-0.504275	5.089063
F	-0.930267	0.778967	2.757055
C	-2.660615	2.365467	0.574351
C	-2.540942	3.109433	1.745232
C	-2.175299	4.446633	1.746616
C	-1.953864	5.102438	0.548896
C	-2.141525	4.423007	-0.643211
C	-2.509791	3.091864	-0.605733
F	-2.796365	2.570697	2.937553
F	-2.023290	5.100264	2.893089
F	-1.586503	6.370758	0.544082
F	-1.977848	5.047067	-1.802923
F	-2.756840	2.505916	-1.782861
C	-4.408574	0.039477	-0.535935
C	-5.316259	0.960284	-1.046371
C	-6.321520	0.601115	-1.931634
C	-6.454921	-0.722622	-2.316175
C	-5.599711	-1.680429	-1.793954
C	-4.613314	-1.282731	-0.913551
F	-5.270460	2.244425	-0.688697
F	-7.161234	1.514024	-2.404851
F	-7.410717	-1.075834	-3.159859
F	-5.742936	-2.956611	-2.128313
F	-3.843586	-2.239704	-0.386925
C	-1.593152	-1.992107	-2.762544
C	-2.463921	-3.058306	-2.986950
C	-2.924708	-3.321475	-4.269271
C	-2.529198	-2.525903	-5.337731
C	-1.655308	-1.469620	-5.112583
C	-1.181275	-1.205140	-3.835817
H	-2.783519	-3.673680	-2.155675
H	-3.608774	-4.147286	-4.426956
H	-2.895452	-2.730698	-6.336844
H	-1.323718	-0.847380	-5.936242
H	-0.490468	-0.388126	-3.675717

[(PFP$_3$Si)(PFP$_2$N)P•]

58

E= -4324.502586

C	3.497633	2.204200	-0.233938
C	3.120676	1.111133	-1.009624
C	3.988212	0.678152	-2.009374
C	5.202146	1.306138	-2.228141
C	5.555400	2.399231	-1.450488
C	4.701891	2.852153	-0.455766
N	1.882954	0.449551	-0.791669
C	1.980808	-0.891737	-0.364659
C	2.648712	-1.203819	0.819449
C	2.760907	-2.507761	1.264305

C	2.193198	-3.539455	0.530372
C	1.532231	-3.255926	-0.653162
C	1.447704	-1.946989	-1.099987
P	0.487388	1.328917	-1.324949
Si	-1.219276	0.385248	-0.146918
C	-2.359592	1.825785	0.307920
C	-2.909503	2.077667	1.558406
C	-3.762061	3.146775	1.787410
C	-4.097622	3.991531	0.740832
C	-3.584443	3.762571	-0.527276
C	-2.736525	2.687093	-0.714554
C	-0.560223	-0.446987	1.409362
C	0.080647	0.344585	2.355523
C	0.641442	-0.169325	3.510332
C	0.572263	-1.534842	3.740964
C	-0.055415	-2.359415	2.820360
C	-0.602849	-1.809504	1.673743
C	-2.333407	-0.767102	-1.142999
C	-3.390446	-1.387708	-0.485658
C	-4.295281	-2.212400	-1.124823
C	-4.162935	-2.417663	-2.491189
C	-3.142375	-1.795055	-3.190459
C	-2.252046	-0.973605	-2.514183
F	3.159397	-0.228252	1.566337
F	3.359665	-2.769266	2.418682
F	2.273393	-4.784650	0.967365
F	0.991471	-4.234947	-1.362005
F	0.828170	-1.710363	-2.249466
F	-1.148043	-2.660875	0.799918
F	-0.088436	-3.670837	3.021053
F	1.114712	-2.051677	4.828878
F	1.246412	0.621110	4.383693
F	0.168274	1.661592	2.158078
F	-2.645159	1.285474	2.596886
F	-4.265860	3.362378	2.995284
F	-4.912479	5.012006	0.949822
F	-3.913149	4.560818	-1.533710
F	-2.267650	2.467928	-1.951864
F	-3.551199	-1.186377	0.827078
F	-5.281665	-2.796429	-0.457462
F	-5.018948	-3.200130	-3.126349
F	-3.032017	-1.977609	-4.499547
F	-1.318769	-0.369039	-3.244844
F	3.648478	-0.356363	-2.770211
F	6.018108	0.879977	-3.182654
F	6.710350	3.010806	-1.658502
F	5.050253	3.891640	0.289775
F	2.708410	2.634150	0.737830

[(PFP$_3$Si)(PFP$_2$N)P•]$_2$

116

E= -8649.064680

C	1.861528	3.645654	0.414411
C	2.382454	2.758903	-0.528220
C	3.674466	3.011237	-0.987225
C	4.447710	4.037762	-0.473916
C	3.929092	4.861149	0.513005
C	2.629831	4.664133	0.954294
N	1.660017	1.643372	-1.022305
C	1.304522	1.709369	-2.392468

C	1.892522	0.909610	-3.363145
C	1.502623	0.973784	-4.689614
C	0.547529	1.897187	-5.081900
C	-0.013589	2.751994	-4.145113
C	0.376665	2.654255	-2.823163
P	0.986465	0.617472	0.240310
Si	2.445780	-1.158226	0.303704
C	2.091476	-2.202471	1.865881
C	2.427304	-3.553731	1.994877
C	2.346051	-4.246727	3.191646
C	1.878290	-3.606600	4.326105
C	1.461432	-2.291584	4.234264
C	1.558964	-1.631433	3.022695
P	-1.085164	0.331964	-0.423153
Si	-2.618891	1.125502	1.121576
C	-2.540355	2.981238	0.759063
C	-2.416395	3.963976	1.739169
C	-2.291429	5.310294	1.438935
C	-2.322567	5.720718	0.117499
C	-2.489131	4.783676	-0.888268
C	-2.592404	3.447222	-0.554202
N	-1.520152	-1.308606	-0.857918
C	-2.419822	-1.401888	-1.952768
C	-3.546315	-2.224658	-1.898520
C	-4.498328	-2.245412	-2.901937
C	-4.334480	-1.455570	-4.027631
C	-3.196720	-0.675198	-4.139724
C	-2.244492	-0.681093	-3.134279
C	-1.100213	-2.512611	-0.257355
C	-0.786245	-3.634075	-1.035925
C	-0.438175	-4.852813	-0.480656
C	-0.420525	-5.008488	0.894299
C	-0.742833	-3.925092	1.688839
C	-1.038473	-2.698683	1.122950
C	-4.282615	0.420660	0.533205
C	-5.273509	1.144702	-0.129136
C	-6.461972	0.569809	-0.554520
C	-6.719186	-0.762453	-0.280262
C	-5.788481	-1.505731	0.426892
C	-4.599552	-0.908405	0.800384
C	-2.400741	0.819081	2.970701
C	-3.332393	0.243138	3.827621
C	-3.046606	-0.032216	5.155649
C	-1.808114	0.305963	5.675718
C	-0.875352	0.941760	4.872314
C	-1.194270	1.189550	3.552567
C	2.716987	-2.305337	-1.168118
C	3.846259	-3.116318	-1.225305
C	4.110221	-3.959862	-2.285509
C	3.232963	-3.989725	-3.361481
C	2.118808	-3.169062	-3.365783
C	1.888723	-2.347884	-2.275856
C	4.048787	-0.150797	0.561207
C	5.048595	-0.009986	-0.401729
C	6.157589	0.804288	-0.227782
C	6.295552	1.536548	0.937047
C	5.331868	1.428015	1.925955
C	4.249875	0.589907	1.727182
F	-0.851175	-3.572722	-2.364823
F	-0.131348	-5.870800	-1.270771

F	-0.092386	-6.167023	1.442534
F	-0.756123	-4.053108	3.011183
F	-1.248754	-1.685373	1.961189
F	2.807646	-4.284179	0.946725
F	2.683689	-5.526949	3.245707
F	1.779015	-4.260875	5.467193
F	0.939616	-1.683224	5.291444
F	1.075328	-0.393746	2.998530
F	-4.568272	-0.049010	3.417395
F	-3.957847	-0.601416	5.935501
F	-1.526763	0.045485	6.940511
F	0.296551	1.306969	5.369903
F	-0.297829	1.845033	2.815517
F	4.984049	-0.639482	-1.570947
F	7.065429	0.910036	-1.186474
F	7.331042	2.338827	1.103943
F	5.449010	2.126879	3.047532
F	3.367949	0.529007	2.723605
F	4.739600	-3.075770	-0.233849
F	5.192689	-4.725407	-2.293567
F	3.475327	-4.785303	-4.388027
F	1.299202	-3.154161	-4.407190
F	0.833061	-1.533920	-2.352675
F	-2.427530	3.655212	3.036473
F	-2.158429	6.206840	2.407760
F	-2.209146	7.003500	-0.182380
F	-2.541878	5.171466	-2.154318
F	-2.777591	2.595633	-1.566519
F	-5.148522	2.448579	-0.365619
F	-7.364414	1.296985	-1.199348
F	-7.852213	-1.315171	-0.672573
F	-6.037753	-2.771286	0.724741
F	-3.731032	-1.668655	1.468349
F	-3.731006	-3.035179	-0.856697
F	-5.573020	-3.012373	-2.772473
F	-5.245325	-1.458911	-4.987525
F	-2.996394	0.059737	-5.224774
F	-1.150156	0.040790	-3.335063
F	2.854032	0.057910	-3.032904
F	2.056011	0.164913	-5.584503
F	0.172198	1.965497	-6.348652
F	-0.919082	3.645286	-4.516753
F	-0.140807	3.498875	-1.936197
F	4.196462	2.246390	-1.942943
F	5.687637	4.218249	-0.910965
F	4.663708	5.838831	1.018237
F	2.110587	5.472537	1.867598
F	0.604357	3.529597	0.825082

(PFP$_3$Si)(t*B*u$_2$N)P•

62

E= -3184.636997

C	2.226399	-1.522604	1.106335
C	1.520874	-1.398701	-0.085034
C	1.882485	-2.270694	-1.102510
C	2.855482	-3.245087	-0.937398
C	3.514935	-3.349637	0.275924
C	3.202125	-2.480320	1.310579
Si	0.267795	0.015709	-0.262289
C	1.410409	1.532927	-0.205766

C	2.217304	1.754217	-1.314821
C	3.106706	2.808826	-1.409319
C	3.216064	3.685959	-0.341081
C	2.447184	3.490402	0.795018
C	1.567814	2.419766	0.851255
F	2.152293	0.901825	-2.347082
F	3.853837	2.982061	-2.492705
F	4.060655	4.704014	-0.403027
F	2.566671	4.322781	1.822746
F	0.881214	2.269321	1.984841
F	1.304949	-2.200387	-2.303918
F	3.165663	-4.067997	-1.932131
F	4.447636	-4.273393	0.446459
F	3.841807	-2.570892	2.469988
F	1.965359	-0.684339	2.115165
P	-0.902579	0.204638	-2.214353
N	-2.524995	-0.233664	-1.866976
C	-3.519239	0.714597	-2.517847
C	-3.529008	0.553093	-4.048482
C	-2.930162	-1.633880	-1.476354
C	-1.696231	-2.489766	-1.157779
C	-3.819266	-1.638503	-0.214046
C	-3.626939	-2.375168	-2.633410
C	-0.924426	0.055080	1.207445
C	-1.172613	-0.988800	2.087822
C	-2.156652	-0.929057	3.061742
C	-2.933711	0.211978	3.175505
C	-2.715909	1.280730	2.319063
C	-1.718390	1.183565	1.367302
F	-0.485209	-2.132845	2.004699
F	-2.369370	-1.958263	3.872745
F	-3.881829	0.281511	4.096476
F	-3.456210	2.378004	2.420516
F	-1.523114	2.244163	0.576963
C	-4.944907	0.564856	-1.966234
C	-3.129407	2.168719	-2.189400
H	-3.880437	2.841207	-2.611587
H	-3.095777	2.324480	-1.111216
H	-2.161655	2.446845	-2.606580
H	-5.562312	1.334549	-2.434699
H	-5.414010	-0.391643	-2.184292
H	-4.967841	0.734319	-0.888476
H	-4.186985	1.303683	-4.495103
H	-2.526634	0.703771	-4.457178
H	-3.880334	-0.429718	-4.360198
H	-3.735737	-2.602769	0.294066
H	-3.492630	-0.861127	0.476361
H	-4.873154	-1.479110	-0.431853
H	-2.029757	-3.512331	-0.968989
H	-0.990766	-2.519958	-1.988300
H	-1.178101	-2.172622	-0.255400
H	-3.862189	-3.392271	-2.308546
H	-4.560011	-1.915766	-2.950456
H	-2.961855	-2.440462	-3.498020

[(PFP$_3$Si)(t*B*u$_2$N)P•]$_2$

124

E= -6369.326864

C	-3.388476	1.337560	-2.507600
C	-3.732090	1.057461	-1.199623

C	-4.990725	1.498553	-0.802444
C	-5.862997	2.152261	-1.653879
C	-5.474936	2.403972	-2.961870
C	-4.223884	1.998879	-3.392035
Si	-2.623647	0.137836	0.053795
P	-0.657122	-1.031140	-0.289508
P	1.048126	-0.330994	-1.670895
N	0.825068	0.968863	-2.757680
C	1.056620	0.689315	-4.244823
C	2.189790	1.559394	-4.822276
C	-2.255720	1.421605	1.409979
C	-1.767141	1.017411	2.648846
C	-1.609008	1.871124	3.722828
C	-1.900535	3.214567	3.566576
C	-2.338812	3.678682	2.339238
C	-2.511949	2.786520	1.294033
C	-3.905793	-1.126745	0.721115
C	-4.342036	-1.295016	2.034274
C	-5.273441	-2.254415	2.405871
C	-5.844414	-3.072692	1.447076
C	-5.495933	-2.895709	0.120420
C	-4.562862	-1.929763	-0.206116
N	-1.062382	-2.641545	-0.747484
C	-1.233595	-3.083818	-2.185779
C	-1.700328	-1.898402	-3.034229
C	-0.945173	-3.689937	0.352531
C	0.006225	-4.837777	-0.026132
C	-0.400779	-3.096510	1.661454
C	-2.315865	-4.277826	0.742824
Si	2.737126	0.054147	-0.097742
C	4.522940	-0.102583	-0.769397
C	5.556341	-0.172900	0.163458
C	6.881986	-0.356172	-0.183155
C	7.221797	-0.473667	-1.522522
C	6.230928	-0.402461	-2.484814
C	4.915005	-0.220762	-2.088710
C	2.800514	1.654511	0.937968
C	3.750264	2.660339	0.782916
C	3.788057	3.788946	1.585726
C	2.856419	3.936953	2.597822
C	1.887614	2.964134	2.782389
C	1.868471	1.866511	1.942948
C	2.781267	-1.490723	1.021590
C	2.831409	-1.528575	2.411672
C	2.967099	-2.710162	3.124833
C	3.103517	-3.909525	2.447880
C	3.127584	-3.911174	1.063955
C	2.962034	-2.717150	0.386542
C	0.732881	2.394450	-2.268240
C	-0.043557	2.430148	-0.947453
C	2.140114	2.996937	-2.068748
C	-0.059036	3.348807	-3.182266
C	1.449932	-0.776058	-4.502082
C	-0.214752	0.902316	-5.090977
C	0.084151	-3.608221	-2.804916
C	-2.304380	-4.172416	-2.376262
H	-1.823610	-2.228805	-4.068223
H	-2.654068	-1.512003	-2.693312
H	-0.990774	-1.078806	-3.038732
H	-2.391716	-4.368925	-3.447976

H	-2.045410	-5.119017	-1.905112
H	-3.281867	-3.849513	-2.020167
H	-0.012901	-3.618630	-3.894291
H	0.925838	-2.969129	-2.543300
H	0.314637	-4.626742	-2.502356
H	-0.082362	4.325247	-2.691504
H	0.394535	3.498861	-4.158512
H	-1.086878	3.017446	-3.317451
H	-0.087660	3.455621	-0.574323
H	-1.065428	2.101176	-1.125245
H	0.385720	1.815537	-0.162299
H	2.120396	3.779101	-1.305041
H	2.862921	2.241892	-1.762418
H	2.524490	3.441770	-2.985764
H	0.031035	0.683054	-6.133803
H	-1.007236	0.221897	-4.786653
H	-0.608164	1.912605	-5.057610
H	1.650746	-0.876404	-5.571275
H	2.336891	-1.092820	-3.963045
H	0.641902	-1.460973	-4.261947
H	2.405174	1.218261	-5.837515
H	1.925974	2.612775	-4.896359
H	3.102863	1.467719	-4.239412
H	-2.160206	-5.078818	1.471308
H	-2.923176	-3.513576	1.224688
H	-2.876844	-4.694558	-0.087204
H	-0.320128	-3.909620	2.387455
H	0.577577	-2.643321	1.537071
H	-1.060900	-2.340791	2.081462
H	0.213400	-5.436810	0.863922

H	-0.413452	-5.513130	-0.770423
H	0.952019	-4.457541	-0.406258
F	4.679277	2.601452	-0.175033
F	4.710239	4.723418	1.391745
F	2.886052	5.000992	3.381904
F	1.005472	3.101002	3.765637
F	0.898410	0.966092	2.115388
F	5.292622	-0.035656	1.468652
F	7.826260	-0.411951	0.747697
F	8.485287	-0.646435	-1.875450
F	6.540285	-0.506164	-3.771589
F	4.006955	-0.149966	-3.062603
F	2.788933	-0.416300	3.141371
F	2.987243	-2.695947	4.451251
F	3.228376	-5.044386	3.116738
F	3.287541	-5.051643	0.402653
F	2.997463	-2.779874	-0.950656
F	-3.920639	-0.509471	3.022297
F	-5.639035	-2.374232	3.676020
F	-6.730930	-3.991652	1.791477
F	-6.041604	-3.653773	-0.824283
F	-4.319028	-1.784507	-1.513885
F	-5.392913	1.333482	0.462969
F	-7.054120	2.555771	-1.229077
F	-6.292436	3.038004	-3.787441
F	-3.824899	2.253816	-4.634297
F	-2.185749	0.991621	-2.964635
F	-2.932402	3.324814	0.143009
F	-2.588342	4.973276	2.168685
F	-1.727359	4.055394	4.571928

F	-1.146481	1.422286	4.881143
F	-1.419409	-0.252934	2.850225

(PFP$_3$Si)(TMS$_2$N)P•

62

E= -3687.568604

C	-1.896178	-2.039689	-1.399354
C	-1.936668	-0.707738	-0.996365
C	-3.155928	-0.061258	-1.162382
C	-4.257189	-0.681984	-1.731329
C	-4.163246	-2.002224	-2.140127
C	-2.974493	-2.693399	-1.968654
Si	-0.356064	0.013906	-0.224030
C	-0.622049	1.833281	0.233511
C	-0.447740	2.405168	1.486711
C	-0.586750	3.766402	1.711968
C	-0.921928	4.601573	0.658910
C	-1.106195	4.071661	-0.609242
C	-0.950134	2.711891	-0.792691
F	-0.115845	1.661368	2.546483
F	-0.409012	4.272074	2.926610
F	-1.061698	5.901762	0.861789
F	-1.411638	4.868346	-1.624829
F	-1.100061	2.240200	-2.035597
F	-3.330346	1.196252	-0.754803
F	-5.402657	-0.026708	-1.876075
F	-5.209769	-2.605542	-2.681247
F	-2.884633	-3.964976	-2.338800
F	-0.779528	-2.755762	-1.215624
P	1.152411	-0.279450	-1.927659
N	2.748780	-0.059915	-1.368701
Si	3.272331	1.346244	-0.414694
C	5.090481	1.713852	-0.699757
Si	3.906256	-1.344841	-1.846539
C	5.057742	-0.722718	-3.188136
C	4.881729	-1.903582	-0.343732
C	2.977119	-2.830915	-2.503895
C	-0.172427	-1.019040	1.354132
C	-1.188022	-0.983853	2.302728
C	-1.163461	-1.740792	3.459354
C	-0.085872	-2.584979	3.687701
C	0.938910	-2.663265	2.759472
C	0.872426	-1.891312	1.610810
F	-2.240706	-0.182652	2.110473
F	-2.149029	-1.670182	4.345241
F	-0.040855	-3.317042	4.789255
F	1.969021	-3.475077	2.970216
F	1.874268	-2.022014	0.739333
C	2.346235	2.891263	-0.965817
C	3.005273	0.995680	1.405702
H	5.349481	2.570875	-0.068388
H	5.776929	0.904773	-0.447859
H	5.270216	2.008373	-1.736949
H	1.832599	3.373631	-0.130420
H	3.058329	3.613809	-1.374218
H	1.611437	2.688360	-1.747953
H	3.360304	1.837083	2.009373
H	1.957594	0.834795	1.666205
H	3.562721	0.103842	1.705128
H	5.634087	-1.559805	-3.595192

H	4.487119	-0.281346	-4.010398
H	5.767768	0.023954	-2.828121
H	3.703358	-3.629750	-2.690179
H	2.242505	-3.205285	-1.787193
H	2.457826	-2.625890	-3.442686
H	5.638971	-2.627492	-0.662612
H	5.401901	-1.097720	0.177166
H	4.226485	-2.399933	0.374883

$[(PFP_3Si)(TMS_2N)P\bullet]_2$

124

E= -7375.214341

C	-3.071531	1.934357	-2.085843
C	-3.588361	1.351466	-0.944085
C	-4.926331	1.627660	-0.679683
C	-5.714536	2.398355	-1.515228
C	-5.156556	2.945672	-2.660948
C	-3.821579	2.718371	-2.946330
Si	-2.589057	0.232497	0.230349
P	-0.619999	-0.880896	-0.231328
P	1.081234	-0.064661	-1.517082
N	0.989807	1.428142	-2.348534
Si	1.232906	1.418935	-4.139018
C	2.647027	2.580122	-4.574432
C	-2.353693	1.226207	1.832689
C	-1.838385	0.609637	2.969140
C	-1.696434	1.246941	4.186127
C	-2.068633	2.574464	4.300096
C	-2.568781	3.240400	3.195314
C	-2.708489	2.563544	1.995460
C	-3.865431	-1.153748	0.556329
C	-4.432715	-1.521366	1.772880
C	-5.470210	-2.437828	1.872741
C	-5.992102	-3.017809	0.729442
C	-5.473482	-2.665948	-0.504192
C	-4.438950	-1.752577	-0.558901
N	-1.088410	-2.347093	-1.017374
Si	-1.227457	-2.613909	-2.781634
C	-1.715872	-1.043980	-3.672279
Si	-1.088422	-3.805926	0.027417
C	0.037329	-5.121522	-0.699060
C	-0.544183	-3.416750	1.773010
C	-2.786189	-4.588828	0.207113
Si	2.736942	-0.005076	0.112505
C	4.478414	-0.158706	-0.648721
C	5.541526	-0.473664	0.194279
C	6.832097	-0.675561	-0.257497
C	7.101697	-0.552951	-1.612933
C	6.079125	-0.227795	-2.486170
C	4.799898	-0.038608	-1.987626
C	2.866215	1.439744	1.345746
C	3.909113	2.359446	1.406864
C	3.941197	3.398388	2.324003
C	2.911631	3.536114	3.237806
C	1.859592	2.634685	3.221450
C	1.861541	1.619921	2.285738
C	2.674694	-1.666027	1.045042
C	2.713825	-1.843043	2.424595
C	2.826276	-3.090696	3.018595
C	2.943141	-4.218627	2.226403

C	2.963152	-4.085562	0.848430
C	2.813485	-2.828615	0.290796
Si	0.948669	3.031001	-1.567821
C	-0.031234	2.963497	0.025401
C	2.718121	3.615901	-1.309905
C	0.097217	4.343028	-2.604673
C	1.619130	-0.282648	-4.807908
C	-0.272542	2.022489	-5.082337
C	0.386585	-3.261293	-3.490094
C	-2.527454	-3.914109	-3.168569
H	-1.555774	-1.187051	-4.745405
H	-2.767245	-0.799380	-3.524092
H	-1.126535	-0.184755	-3.362028
H	-2.567859	-4.010031	-4.259335
H	-2.286163	-4.902758	-2.772084
H	-3.525350	-3.635003	-2.827448
H	0.357238	-3.204424	-4.583107
H	1.243192	-2.683691	-3.142022
H	0.548270	-4.307278	-3.225114
H	0.076478	5.253986	-1.995364
H	0.615772	4.590159	-3.532156
H	-0.933557	4.080518	-2.845016
H	0.321563	3.727916	0.724434
H	-1.076682	3.187096	-0.196641
H	0.004283	2.006111	0.541020
H	2.813709	4.233029	-0.411699
H	3.430783	2.793576	-1.234534
H	3.031829	4.224791	-2.161564
H	-0.030590	1.953654	-6.149137
H	-1.156642	1.409886	-4.905517
H	-0.535795	3.058823	-4.873133
H	1.939172	-0.144613	-5.846690
H	2.410855	-0.809493	-4.279315
H	0.735102	-0.917755	-4.821288
H	2.853636	2.468593	-5.644054
H	2.409313	3.632211	-4.404694
H	3.562948	2.342572	-4.032761
H	-2.647477	-5.599827	0.605592
H	-3.389240	-4.039973	0.932579
H	-3.349085	-4.676989	-0.722166
H	-0.323896	-4.364267	2.277090
H	0.331644	-2.774586	1.830829
H	-1.339669	-2.921424	2.332076
H	0.226984	-5.881066	0.066328
H	-0.419516	-5.631333	-1.551086
H	0.999388	-4.728031	-1.026253
F	4.940625	2.305586	0.562069
F	4.948121	4.262557	2.325788
F	2.927853	4.521956	4.118364
F	0.871709	2.761299	4.100646
F	0.830072	0.771019	2.292650
F	5.343276	-0.560853	1.514837
F	7.811020	-0.970769	0.588228
F	8.331473	-0.737327	-2.064320
F	6.323654	-0.094688	-3.784125
F	3.859173	0.288502	-2.875026
F	2.677654	-0.807068	3.260792
F	2.832132	-3.208234	4.339631
F	3.049120	-5.415778	2.779698
F	3.132437	-5.156666	0.082198

F	2.847529	-2.756751	-1.045738
F	-4.037627	-0.978965	2.923167
F	-5.973192	-2.753019	3.059714
F	-6.972876	-3.900349	0.815191
F	-5.950131	-3.219438	-1.614177
F	-4.005537	-1.419829	-1.780333
F	-5.500365	1.177198	0.441082
F	-6.987427	2.633621	-1.222931
F	-5.892578	3.693454	-3.466878
F	-3.268800	3.263411	-4.025323
F	-1.781420	1.775811	-2.381572
F	-3.191517	3.288356	0.979287
F	-2.907718	4.521709	3.289623
F	-1.919003	3.211570	5.448696
F	-1.185069	0.608044	5.229472
F	-1.446993	-0.662287	2.917923

(PFP$_3$Si)(PFS$_2$N)P•

62

E= -5473.844152

C	2.973500	-1.887747	-1.848802
C	2.994382	-1.071321	-0.728265
C	4.201968	-0.989533	-0.043712
C	5.334866	-1.676151	-0.435867
C	5.272480	-2.482763	-1.563840
C	4.088612	-2.591892	-2.275574
Si	1.551303	-0.046571	-0.080969
P	-0.176190	-0.173914	-1.616993
N	-1.683454	-0.101022	-0.621849
Si	-2.468294	1.436616	-0.403802

C	-1.350693	2.982324	-0.808599
F	-2.185748	4.027968	-0.948108
F	4.279501	-0.234355	1.057221
F	6.466384	-1.576806	0.247507
F	6.344075	-3.148263	-1.957953
F	4.032205	-3.362191	-3.352588
F	1.856996	-2.021113	-2.567896
C	1.053536	-0.608892	1.637585
C	0.172131	0.178813	2.361550
C	-0.231423	-0.110867	3.648904
C	0.251714	-1.266141	4.246001
C	1.127466	-2.090868	3.557980
C	1.514172	-1.756902	2.270697
F	-0.287850	1.305663	1.796586
F	2.346367	-2.594305	1.654109
F	1.580643	-3.196279	4.132329
F	-0.121476	-1.578585	5.474031
F	-1.050339	0.689065	4.312481
C	2.172491	1.734599	-0.079460
C	2.470256	2.505197	1.037057
C	2.871700	3.826777	0.933733
C	3.002799	4.405927	-0.319214
C	2.744758	3.660534	-1.459737
C	2.348832	2.344815	-1.314581
F	2.383298	1.994185	2.264301
F	3.138333	4.539111	2.019613
F	3.385770	5.666417	-0.426977
F	2.885569	4.205523	-2.659359
F	2.125207	1.633976	-2.430054
Si	-2.437307	-1.668190	-0.623582

C -4.255953 -1.695401 0.082549
F -5.141510 -2.118803 -0.819549
C -2.476980 -2.501559 -2.381921
F -3.369565 -3.501947 -2.407293
C -1.448996 -2.921807 0.468029
F -0.152735 -3.005500 0.098912
F -2.803863 -1.633940 -3.347312
F -1.284950 -3.035838 -2.700403
C -3.175587 1.754521 1.387936
F -4.488775 2.005222 1.377722
C -3.852021 1.599651 -1.762802
F -3.287946 2.008111 -2.914001
F -2.966484 0.696936 2.192275
F -2.581285 2.813847 1.953304
F -4.433042 0.410628 -2.026854
F -4.820974 2.462860 -1.456415
F -0.458686 3.314572 0.129636
F -0.672172 2.854145 -1.963216
F -1.469892 -2.580475 1.767385
F -1.965962 -4.152138 0.363518
F -4.337743 -2.506900 1.144350
F -4.658557 -0.473899 0.489022

[(PFP$_3$Si)(PFS$_2$N)P•]$_2$

124

E= -10947.7079441

C -1.439939 -3.900890 -3.015720
F -2.181597 -4.988962 -2.744608
Si -2.312586 -2.302589 -2.269526
C -2.281877 -0.986957 -3.730199
F -1.135807 -1.022723 -4.417058
F -1.384683 -3.787845 -4.351763
F -0.206086 -4.158910 -2.582011
N -1.820557 -1.835877 -0.624148
P -1.114247 -0.153228 -0.647551
P 0.982763 0.457288 -0.118333
Si 2.482549 -0.649414 1.296591
C 4.258612 -0.083518 0.968282
C -4.175263 -2.884775 -2.303720
F -5.041425 -1.866381 -2.209660
Si -2.140677 -2.922481 0.729355
C -3.902048 -2.722548 1.569475
F -4.352929 -3.920624 1.961913
C -2.005586 -4.862890 0.462531
F -2.992595 -5.464237 -0.189483
C -0.832446 -2.761614 2.163295
F -0.120949 -1.612251 2.021787
F -0.854050 -5.207105 -0.124212
F -1.993061 -5.381620 1.709507
F -4.448910 -3.535565 -3.438389
F -4.450876 -3.730438 -1.292090
F -3.252767 -1.367626 -4.579850
F -2.519699 0.284786 -3.414749
Si -2.388926 1.341956 0.646912
C -1.812094 1.964485 2.330152
C -2.648780 2.826870 -0.503499
C -4.163059 0.701246 0.800972
N 1.791885 1.054894 -1.636640
Si 2.147068 2.774546 -1.381348
C 1.070540 3.891924 -0.172684

F	0.344636	3.267106	0.754668
Si	2.144571	0.250298	-3.165699
C	1.173877	0.994999	-4.698555
F	1.879775	1.982508	-5.272403
C	3.972615	0.297243	-3.841687
F	4.854580	-0.410841	-3.133515
C	1.668871	-1.628849	-3.128824
F	0.466002	-1.718835	-2.495909
F	3.914925	-0.261459	-5.068563
F	4.471650	1.527057	-3.982487
C	2.712425	-2.518255	1.245974
C	1.966192	-0.036871	3.005506
F	-1.374857	-2.753350	3.376771
F	0.056139	-3.760612	2.147609
F	-3.875659	-1.938977	2.656702
F	-4.823448	-2.205487	0.742799
C	3.981026	2.993008	-0.753318
F	4.767318	2.010092	-1.242490
C	2.154804	3.974099	-2.929642
F	1.020565	3.961829	-3.630438
F	4.512367	4.150551	-1.156212
F	4.090080	2.957762	0.583093
F	2.310565	5.220946	-2.445860
F	3.170748	3.760854	-3.770492
F	1.908755	4.718104	0.468085
F	0.215562	4.662276	-0.867444
F	1.520585	-2.151033	-4.339431
F	2.518936	-2.411362	-2.466709
F	0.979422	0.072375	-5.646840
F	-0.019118	1.491529	-4.360170
C	1.740160	-0.877945	4.093783
C	1.531969	-0.400235	5.376232
C	1.551316	0.965517	5.607126
C	1.751392	1.837791	4.550237
C	1.949324	1.326252	3.281965
F	1.723787	-2.201169	3.949033
F	1.316311	-1.236363	6.379399
F	1.355120	1.434431	6.824368
F	1.749599	3.148496	4.760154
F	2.190783	2.203202	2.310738
C	5.078913	0.658266	1.814058
C	6.435732	0.813951	1.583426
C	7.028881	0.185070	0.502041
C	6.260494	-0.597772	-0.344468
C	4.906667	-0.699732	-0.095911
F	4.607538	1.250709	2.906935
F	7.173123	1.547928	2.404496
F	8.323686	0.320505	0.285430
F	6.824386	-1.231294	-1.360483
F	4.183520	-1.464046	-0.918590
C	2.069198	-3.381536	0.377655
C	2.461369	-4.694744	0.192141
C	3.511323	-5.193394	0.946770
C	4.156568	-4.377249	1.864368
C	3.751377	-3.062286	1.993476
F	1.004329	-2.953585	-0.305538
F	1.844191	-5.476969	-0.677435
F	3.891644	-6.449892	0.800431
F	5.150715	-4.857985	2.597398
F	4.404123	-2.299542	2.878067

C	-3.723049	3.669254	-0.222992
C	-4.144272	4.666646	-1.080974
C	-3.472662	4.853594	-2.279717
C	-2.381041	4.060107	-2.588123
C	-1.980612	3.088257	-1.687564
F	-4.393963	3.536691	0.927110
F	-5.174886	5.439371	-0.769303
F	-3.862519	5.798498	-3.116046
F	-1.725641	4.251931	-3.720021
F	-0.870415	2.404929	-1.993733
C	-1.659984	3.303894	2.687808
C	-1.405686	3.694648	3.993545
C	-1.335974	2.742611	4.996856
C	-1.502141	1.402735	4.685206
C	-1.722201	1.051486	3.369879
F	-1.778554	4.285355	1.800345
F	-1.249467	4.975325	4.290622
F	-1.109127	3.108210	6.243723
F	-1.456044	0.482165	5.640641
F	-1.942248	-0.235458	3.105179
C	-4.931822	0.656044	1.960899
C	-6.293782	0.407816	1.941277
C	-6.944178	0.230972	0.731964
C	-6.226740	0.300208	-0.451277
C	-4.865456	0.520866	-0.386236
F	-4.400369	0.874713	3.161200
F	-6.981039	0.356109	3.073385
F	-8.244209	0.004122	0.706014
F	-6.843829	0.158825	-1.613271
F	-4.199385	0.587341	-1.544229

(t*B*u$_3$Si)(Ph$_2$N)P•

64

E= -1622.391954

C	-1.521029	2.204136	-1.245460
C	-1.771977	1.281190	-0.234089
C	-2.220760	1.731257	1.008984
C	-2.368166	3.089212	1.244032
C	-2.075926	4.012572	0.244138
C	-1.658915	3.565186	-1.001813
N	-1.610038	-0.111011	-0.474090
P	-0.214340	-0.820290	-1.217676
Si	1.695548	-0.264993	-0.030992
C	1.443928	-0.874959	1.803715
C	1.327284	-2.408209	1.872032
C	-2.765563	-0.923439	-0.333800
C	-4.037967	-0.415836	-0.616374
C	-5.156564	-1.222659	-0.479002
C	-5.030442	-2.546353	-0.071021
C	-3.768584	-3.053422	0.208044
C	-2.643649	-2.248909	0.088472
C	2.962400	-1.408442	-0.991291
C	2.367270	-2.789524	-1.342803
C	2.246660	1.602629	-0.108106
C	2.004317	2.198363	-1.507586
C	3.373391	-0.772803	-2.332985
C	4.242966	-1.655523	-0.170780
C	1.456677	2.468241	0.890365
C	3.742966	1.775343	0.220196
C	2.607790	-0.451275	2.719098

C	0.138690	-0.316314	2.399233
H	1.988582	-3.331378	-0.476726
H	1.554149	-2.716091	-2.068116
H	3.153731	-3.405669	-1.797057
H	3.977040	0.127104	-2.205411
H	3.980385	-1.490017	-2.899310
H	2.507143	-0.519883	-2.952175
H	4.050359	-2.259655	0.718267
H	4.963611	-2.210009	-0.785228
H	4.730605	-0.733207	0.150050
H	0.953621	2.149194	-1.791214
H	2.292998	3.257168	-1.501554
H	2.585842	1.706875	-2.286793
H	1.735692	3.519931	0.750099
H	0.378944	2.399090	0.744633
H	1.677958	2.214395	1.928693
H	3.982579	2.845976	0.239572
H	4.013031	1.365056	1.195693
H	4.390254	1.316887	-0.529587
H	2.458707	-0.884378	3.716418
H	3.578021	-0.796578	2.355671
H	2.662144	0.631626	2.845445
H	1.066048	-2.702955	2.896011
H	0.544356	-2.791384	1.211222
H	2.260846	-2.913762	1.619519
H	0.059804	-0.628531	3.448476
H	0.087488	0.772206	2.375857
H	-0.736806	-0.704814	1.874959
H	-2.445706	1.009077	1.784209
H	-2.705068	3.430023	2.216333
H	-2.184067	5.073869	0.434577
H	-1.448152	4.274560	-1.793817
H	-1.229207	1.843667	-2.225092
H	-1.663173	-2.640932	0.329929
H	-3.654371	-4.080201	0.536547
H	-5.908162	-3.173496	0.031432
H	-6.135510	-0.814990	-0.704870
H	-4.144803	0.609786	-0.948183

[(t*B*u$_3$Si)(Ph$_2$N)P•]$_2$

128

E= -3244.850445

C	3.039121	-1.808398	2.921532
C	1.930102	-1.834056	2.069729
C	1.484359	-3.071132	1.609401
C	2.136940	-4.244076	1.959377
C	3.244740	-4.210506	2.795103
C	3.682575	-2.984211	3.279399
N	1.298941	-0.627610	1.627814
C	1.170960	0.449235	2.539961
C	1.040608	0.206372	3.913422
C	0.877308	1.254232	4.805226
C	0.835260	2.569353	4.359261
C	0.940173	2.812718	2.998256
C	1.091335	1.769057	2.097122
P	0.971728	-0.516822	-0.090242
P	-1.017082	0.159813	-0.706035
Si	-2.628210	-1.557498	-0.877174
C	-3.212545	-2.433287	0.761597
C	-3.910908	-3.780357	0.476099

N	-1.704427	1.543282	0.087714
C	-2.337981	1.558109	1.360582
C	-3.421097	2.415661	1.587754
C	-4.046734	2.446124	2.824022
C	-3.611028	1.627654	3.861397
C	-2.546960	0.768043	3.635229
C	-1.925824	0.729197	2.395369
C	-1.585545	2.809525	-0.566128
C	-1.938044	2.976863	-1.903282
C	-1.781449	4.209079	-2.523617
C	-1.281409	5.294874	-1.818443
C	-0.943346	5.136789	-0.478806
C	-1.093876	3.907135	0.142857
C	-4.126669	-0.676109	-1.772996
C	-4.612915	0.593698	-1.048940
C	-1.781086	-2.837556	-2.091943
C	-2.815269	-3.725718	-2.812446
C	-3.751331	-0.259453	-3.208183
C	-5.344889	-1.619431	-1.872177
C	-0.940852	-2.142289	-3.176999
C	-0.813581	-3.768665	-1.341516
C	-2.019406	-2.733295	1.687221
C	-4.230603	-1.574623	1.536744
Si	2.863852	0.363911	-1.257234
C	3.630558	-1.334115	-1.883612
C	2.570442	-2.319807	-2.407292
C	4.179969	1.322895	-0.164176
C	5.569520	1.278999	-0.838809
C	2.281701	1.489877	-2.730656
C	3.468470	2.095113	-3.507225
C	3.842950	2.813210	0.033966
C	4.346062	0.719089	1.239048
C	1.417440	0.709062	-3.735701
C	1.428513	2.659221	-2.217454
C	4.625288	-1.120968	-3.043371
C	4.360108	-2.070308	-0.742971
H	4.686408	-3.052570	-1.106067
H	5.251229	-1.546604	-0.395109
H	3.710633	-2.249981	0.117698
H	4.116636	-0.793823	-3.953337
H	5.416522	-0.405099	-2.821649
H	5.108345	-2.078380	-3.274676
H	1.889108	-2.638587	-1.619983
H	1.975715	-1.912595	-3.224609
H	3.080897	-3.212007	-2.790903
H	1.961402	-0.108131	-4.213623
H	0.515347	0.310380	-3.268978
H	1.094451	1.391708	-4.531777
H	4.140268	1.345149	-3.926564
H	3.071079	2.681640	-4.344933
H	4.060703	2.778289	-2.896187
H	1.007170	3.204896	-3.069664
H	0.590518	2.312924	-1.618874
H	1.994731	3.375625	-1.625662
H	5.216524	1.179470	1.723728
H	3.482270	0.930533	1.866069
H	4.508981	-0.357173	1.228104
H	3.937709	3.388036	-0.888425
H	2.844862	2.977344	0.439657
H	4.555582	3.237704	0.751671

H	6.001854	0.278158	-0.841688
H	5.564827	1.650468	-1.864615
H	6.250682	1.919446	-0.265096
H	-1.504558	-1.428533	-3.776094
H	-0.094960	-1.617684	-2.739794
H	-0.535885	-2.902444	-3.857127
H	-1.315556	-4.425210	-0.629753
H	-0.297150	-4.410126	-2.066122
H	-0.049592	-3.195035	-0.817285
H	-3.421825	-3.162579	-3.523500
H	-2.277645	-4.489889	-3.387264
H	-3.488077	-4.248005	-2.130536
H	-1.360333	-1.877776	1.833006
H	-2.387989	-3.038509	2.673797
H	-1.413097	-3.554909	1.306645
H	-4.423521	-2.043485	2.509310
H	-3.882667	-0.563359	1.732782
H	-5.188680	-1.511587	1.018287
H	-4.263520	-4.198882	1.426897
H	-4.781982	-3.676427	-0.173614
H	-3.242095	-4.518435	0.031280
H	-6.114154	-1.132874	-2.484614
H	-5.117826	-2.577771	-2.339624
H	-5.793475	-1.816266	-0.897390
H	-4.548462	0.371821	-3.619250
H	-2.820268	0.310395	-3.249815
H	-3.643696	-1.116600	-3.874992
H	-5.513831	0.962593	-1.555237
H	-4.871759	0.422302	-0.005184
H	-3.875070	1.391860	-1.077810
H	1.051500	-0.811033	4.282825
H	0.772445	1.034580	5.861989
H	0.709644	3.386555	5.058919
H	0.904220	3.830171	2.624363
H	1.118961	1.975289	1.035263
H	3.402785	-0.865750	3.306555
H	4.544557	-2.934250	3.935284
H	3.757263	-5.124984	3.069184
H	1.768187	-5.188759	1.575736
H	0.627173	-3.105879	0.957570
H	-3.769639	3.061999	0.791483
H	-4.885620	3.116341	2.975489
H	-4.098421	1.660526	4.828636
H	-2.186245	0.116279	4.422568
H	-1.114770	0.042751	2.228542
H	-2.308656	2.133237	-2.468803
H	-2.054762	4.316214	-3.567337
H	-1.158523	6.255391	-2.304930
H	-0.553251	5.975939	0.086480
H	-0.840832	3.784679	1.188273

(t*B*u$_3$Si)(PFP$_2$N)P•

64

E= -2614.689730

C	-0.400638	2.153382	-1.027407
C	-0.728273	1.218406	-0.046987
C	-1.021261	1.701984	1.227401
C	-0.892963	3.045474	1.538629
C	-0.491674	3.943359	0.561184
C	-0.247711	3.495850	-0.727820

N	-0.818383	-0.157031	-0.345112
P	0.410458	-1.155772	-1.086373
Si	2.501590	-0.870692	-0.106954
C	2.368521	-1.367963	1.765437
C	2.001117	-2.855444	1.918103
F	-1.438627	0.869415	2.175369
F	-1.150569	3.474954	2.767400
F	-0.354729	5.227456	0.855920
F	0.111559	4.355256	-1.672080
F	-0.220887	1.761591	-2.283354
C	-2.118407	-0.715619	-0.298031
C	-3.158634	-0.209144	-1.076529
C	-4.433731	-0.746373	-1.022938
C	-4.688193	-1.830215	-0.197458
C	-3.668992	-2.361281	0.577104
C	-2.403717	-1.800369	0.529496
F	-2.934791	0.812004	-1.897782
F	-1.458556	-2.317632	1.306230
F	-3.913488	-3.393248	1.374746
F	-5.903006	-2.356691	-0.148405
F	-5.405733	-0.241429	-1.772390
C	3.434699	-2.229475	-1.157373
C	2.587367	-3.502617	-1.373663
C	3.310438	0.884554	-0.340148
C	3.013246	1.471470	-1.732098
C	3.784839	-1.705879	-2.563004
C	4.744648	-2.659216	-0.468672
C	2.782145	1.888930	0.700271
C	4.842862	0.826210	-0.178755
C	3.686448	-1.122594	2.523408

C	1.252831	-0.573906	2.465277
H	2.215472	-3.939518	-0.447519
H	1.728449	-3.321917	-2.023906
H	3.210573	-4.258624	-1.867193
H	4.539304	-0.917938	-2.542609
H	4.196072	-2.529229	-3.159501
H	2.906908	-1.326338	-3.094882
H	4.560830	-3.197579	0.463249
H	5.291464	-3.341206	-1.131522
H	5.407026	-1.820273	-0.247290
H	1.947271	1.569312	-1.926621
H	3.453385	2.474011	-1.800612
H	3.435871	0.877217	-2.541146
H	3.165373	2.888858	0.463568
H	1.693756	1.949226	0.705098
H	3.108085	1.650264	1.713757
H	5.245595	1.845126	-0.233343
H	5.154251	0.404490	0.779116
H	5.321405	0.250884	-0.973073
H	3.581550	-1.493206	3.550572
H	4.537238	-1.640579	2.075766
H	3.935214	-0.061663	2.591183
H	1.824556	-3.071379	2.978782
H	1.085448	-3.110226	1.378280
H	2.797594	-3.522030	1.582781
H	1.223562	-0.851059	3.526233
H	1.396448	0.505974	2.420280
H	0.276493	-0.809333	2.045827

[(t*B*u$_3$Si)(PFP$_2$N)P•]$_2$

128

E= -5229.430518

P	-0.845969	-1.394376	0.014918
P	0.885293	-0.165325	-0.752250
Si	2.551630	-1.375233	-1.990466
C	1.500351	-2.551331	-3.147451
C	0.258418	-1.848064	-3.721275
N	1.664521	1.109327	0.215430
C	1.138749	2.425819	0.191324
C	0.919134	3.128167	1.382280
C	0.414495	4.414866	1.403067
C	0.090371	5.048379	0.214729
C	0.302276	4.386702	-0.980069
C	0.839855	3.109616	-0.985647
F	1.186252	2.556198	2.556698
F	0.220556	5.035311	2.559911
F	-0.413319	6.275319	0.224750
F	0.009255	4.980885	-2.129927
F	1.055736	2.557529	-2.175662
Si	-1.641559	-2.558326	2.046024
C	-0.382719	-4.041752	2.244968
C	0.862074	-3.676176	3.073074
N	-2.156786	-0.281222	-0.459968
C	-2.333033	-0.130866	-1.859539
C	-2.808798	-1.157474	-2.670856
C	-2.904804	-1.016251	-4.044565
C	-2.541082	0.175086	-4.647557
C	-2.099305	1.228354	-3.863932
C	-2.013504	1.069502	-2.493286
F	-3.145606	-2.331441	-2.149336

F	-3.317405	-2.036695	-4.788564
F	-2.610569	0.305613	-5.964968
F	-1.751517	2.378639	-4.426495
F	-1.622599	2.113977	-1.766148
C	3.430783	-0.008918	-3.068984
C	2.472610	0.567523	-4.129933
C	3.868097	-2.366235	-0.940693
C	4.972671	-1.451728	-0.381838
C	2.755051	0.925824	1.086629
C	3.874181	1.762007	1.080494
C	4.947698	1.546161	1.929805
C	4.928735	0.490124	2.827540
C	3.810719	-0.324316	2.888578
C	2.740735	-0.071641	2.052345
F	3.934338	2.810024	0.261946
F	1.646430	-0.812554	2.186642
F	3.751498	-1.324629	3.760602
F	5.965247	0.269349	3.623995
F	6.004139	2.347565	1.875583
C	-1.834261	-1.587105	3.727917
C	-3.089517	-0.695307	3.740458
C	-3.374341	-3.245300	1.437981
C	-4.116896	-3.955844	2.592869
C	-1.998051	-2.583197	4.899608
C	-0.618971	-0.708384	4.069353
C	-2.781924	0.716215	0.307639
C	-4.122150	1.054732	0.090793
C	-4.756111	2.054873	0.808033
C	-4.070634	2.754364	1.788898
C	-2.747282	2.437375	2.034566

C	-2.128882	1.446606	1.293514
F	-4.832257	0.417468	-0.839733
F	-6.028396	2.339762	0.561087
F	-4.679367	3.701875	2.488139
F	-2.068494	3.066730	2.986322
F	-0.864150	1.162468	1.585185
C	-4.339313	-2.165840	0.917532
C	-3.200067	-4.268890	0.298619
C	-1.024982	-5.261946	2.938531
C	0.118480	-4.507072	0.872655
C	3.951379	1.175114	-2.239999
C	4.648043	-0.596359	-3.817111
C	3.219948	-3.079219	0.253299
C	4.570546	-3.446974	-1.788245
C	2.323292	-3.052158	-4.352663
C	0.980441	-3.783074	-2.388465
H	5.619437	-2.039177	0.280461
H	5.608841	-1.032972	-1.161242
H	4.572752	-0.626638	0.202305
H	3.883650	-4.226611	-2.121288
H	5.075762	-3.038893	-2.665430
H	5.334226	-3.934604	-1.170134
H	2.588573	-2.417815	0.844423
H	2.609994	-3.923434	-0.061327
H	3.998602	-3.475191	0.916235
H	1.771291	-4.374132	-1.923830
H	0.258477	-3.495893	-1.624917
H	0.458526	-4.441100	-3.093582
H	3.237160	-3.576636	-4.072365
H	1.704701	-3.757923	-4.919815
H	2.588228	-2.245171	-5.036873
H	-0.349532	-1.411940	-2.934341
H	0.500465	-1.060396	-4.432662
H	-0.360036	-2.587302	-4.245544
H	4.495672	1.860066	-2.901030
H	3.142220	1.741471	-1.792838
H	4.641528	0.876304	-1.452368
H	2.284333	-0.140807	-4.938261
H	1.515141	0.876649	-3.709178
H	2.934049	1.453229	-4.581625
H	5.467255	-0.854854	-3.144800
H	4.412404	-1.477114	-4.414155
H	5.030092	0.168201	-4.504159
H	-0.677525	-4.845504	0.211135
H	0.651912	-3.708736	0.365662
H	0.821360	-5.339513	1.002856
H	0.627651	-3.457146	4.115222
H	1.546214	-4.533292	3.072844
H	1.405842	-2.829650	2.666962
H	-1.819613	-5.716745	2.347603
H	-0.248521	-6.025260	3.068952
H	-1.420481	-5.034255	3.929361
H	-0.445068	0.072200	3.335058
H	-0.799912	-0.222399	5.035834
H	0.298423	-1.287698	4.163919
H	-3.072785	-0.065428	4.637494
H	-3.151091	-0.032237	2.880419
H	-4.010400	-1.279296	3.773442
H	-2.196411	-2.006212	5.810782
H	-2.830388	-3.274905	4.769675

H	-1.095435	-3.165608	5.084542
H	-4.999292	-4.452571	2.172190
H	-3.528705	-4.720427	3.098061
H	-4.479657	-3.252336	3.343853
H	-4.187300	-4.512396	-0.110986
H	-2.595682	-3.879742	-0.520985
H	-2.756945	-5.204601	0.641460
H	-5.321733	-2.625002	0.756004
H	-4.481834	-1.341535	1.616255
H	-4.014416	-1.764889	-0.035478

(t*B*u$_3$Si)(t*B*u$_2$N)P•

68

E= -1474.811224

C	-1.899802	2.450403	0.223903
C	-2.864389	1.306888	-0.129345
C	-3.532544	1.668897	-1.473211
C	-3.917356	1.317338	0.988544
N	-2.077543	0.025565	-0.171599
P	-0.655729	0.152713	-1.205407
Si	1.365816	-0.001885	-0.014814
C	1.229880	-0.479904	1.866360
C	0.911617	-1.972041	2.063108
C	-2.770204	-1.307371	-0.087744
C	-1.855930	-2.412172	-0.635421
C	-3.069494	-1.680069	1.377322
C	-4.059757	-1.405100	-0.925823
C	2.198469	1.764845	-0.231534
C	1.706667	2.777862	0.819140
C	2.372829	-1.334715	-1.037028
C	2.720207	-0.809115	-2.443502
C	3.733382	1.703440	-0.103827
C	1.864010	2.377590	-1.607480
C	3.682125	-1.742573	-0.335806
C	1.555944	-2.620221	-1.266150
C	2.534724	-0.198345	2.635913
C	0.089356	0.314695	2.531005
H	-2.457131	3.384325	0.332120
H	-1.390015	2.242097	1.164903
H	-1.152318	2.608830	-0.557074
H	2.137141	3.761264	0.591777
H	0.623253	2.892040	0.815125
H	2.017998	2.521452	1.832649
H	-4.378703	2.308120	1.014972
H	-4.720034	0.596615	0.842890
H	-3.452860	1.136551	1.960343
H	-3.914305	2.693382	-1.432081
H	-2.806853	1.614503	-2.288808
H	-4.369284	1.016009	-1.715191
H	2.393010	3.333393	-1.712227
H	2.161102	1.747341	-2.445654
H	0.796273	2.581912	-1.716436
H	4.137436	2.722864	-0.149010
H	4.060357	1.267064	0.842710
H	4.200450	1.140293	-0.912887
H	-0.035860	-0.024946	3.567420
H	0.296112	1.385201	2.566608
H	-0.856904	0.169859	2.005538
H	2.411221	-0.515715	3.679333
H	3.388040	-0.744962	2.228164

H	2.793123	0.861112	2.653425
H	0.788941	-2.172981	3.134870
H	-0.017506	-2.269676	1.575932
H	1.710507	-2.623434	1.704306
H	3.182909	-1.617184	-3.024027
H	1.830269	-0.484531	-2.991449
H	3.430188	0.018362	-2.425434
H	2.179078	-3.348346	-1.800708
H	1.228672	-3.095306	-0.340775
H	0.675783	-2.432020	-1.884410
H	4.245004	-2.423027	-0.987655
H	4.331254	-0.894751	-0.112458
H	3.495725	-2.276962	0.598522
H	-3.462094	-2.700459	1.425824
H	-2.156411	-1.639648	1.973726
H	-3.804349	-1.026928	1.841694
H	-2.332779	-3.382174	-0.474615
H	-1.682718	-2.303182	-1.708695
H	-0.893920	-2.433685	-0.129558
H	-4.437180	-2.430402	-0.874303
H	-4.858355	-0.752660	-0.576256
H	-3.855373	-1.174835	-1.973982

[(t*B*u$_3$Si)(t*B*u$_2$N)P•]$_2$

136

E= -2949.625266

C	0.475520	-2.815503	3.305721
C	1.729816	-2.158674	2.667334
C	2.929805	-3.078189	2.990187
C	2.024377	-0.874827	3.449885
N	1.562545	-1.872241	1.180090
C	1.625984	-3.060432	0.245046
C	0.969717	-2.657235	-1.066048
P	1.234770	-0.213828	0.686612
Si	2.948187	0.694282	-0.802202
C	4.600221	0.563297	0.256145
C	4.518348	1.456055	1.510726
P	-1.000942	0.674596	0.017009
N	-1.540507	1.763019	1.311162
C	-1.514054	1.368399	2.766370
C	-1.657280	-0.147974	2.854059
Si	-2.935363	-0.637313	-0.844357
C	-2.505739	-0.724552	-2.769183
C	-1.890801	0.595164	-3.279162
C	-4.566802	0.427790	-0.626999
C	-4.737257	0.810144	0.853026
C	-3.347709	-2.427918	-0.126252
C	-2.058156	-3.172896	0.202989
C	-4.546193	1.709123	-1.479409
C	-5.850511	-0.323460	-1.044427
C	3.061688	-3.500375	-0.094539
C	0.902109	-4.327066	0.753483
C	3.196923	0.012059	-2.633648
C	3.933208	-1.339543	-2.710249
C	2.482075	2.584368	-0.951410
C	1.458944	2.804671	-2.081184
C	1.819911	-0.133469	-3.333977
C	4.055357	0.970400	-3.492625
C	1.869398	3.104017	0.356780
C	3.684768	3.504681	-1.254022

C	4.888402	-0.859179	0.749502
C	5.843193	0.997922	-0.546663
C	-1.779047	3.236116	0.983828
C	-0.958678	4.213786	1.857874
C	-1.424615	3.577033	-0.469548
C	-3.258006	3.673477	1.139526
C	-0.199678	1.786063	3.467419
C	-2.663501	1.922988	3.636007
C	-4.171316	-2.430843	1.177313
C	-4.130131	-3.294207	-1.135647
C	-1.493328	-1.828994	-3.109873
C	-3.758064	-0.992355	-3.631560
H	-1.644725	-0.466368	3.898889
H	-2.600500	-0.457732	2.408152
H	-0.852675	-0.658459	2.335541
H	-4.286770	-3.471521	1.504546
H	-3.680258	-1.898773	1.991281
H	-5.174851	-2.026447	1.060762
H	-2.536672	1.510245	4.640716
H	-2.644214	3.005952	3.740809
H	-3.646896	1.615876	3.278419
H	-0.060498	1.180720	4.367615
H	0.655864	1.640714	2.808782
H	-0.216400	2.823828	3.792217
H	-2.289226	-4.094370	0.752610
H	-1.513094	-3.467723	-0.692045
H	-1.395073	-2.566663	0.824019
H	-4.325374	-4.272837	-0.678776
H	-5.095756	-2.866495	-1.408158
H	-3.570920	-3.478896	-2.053328
H	0.827116	-5.028034	-0.081766
H	1.452722	-4.840437	1.539695
H	-0.099998	-4.134361	1.114229
H	0.844883	-3.523634	-1.720369
H	-0.001381	-2.218169	-0.879431
H	1.561815	-1.920412	-1.593593
H	3.018857	-4.255827	-0.885621
H	3.645823	-2.668514	-0.462855
H	3.593648	-3.938463	0.746067
H	0.392854	-2.510801	4.352430
H	-0.441308	-2.518366	2.803465
H	0.525597	-3.903812	3.291610
H	2.219365	-1.145441	4.490901
H	2.905926	-0.362451	3.064762
H	1.192131	-0.185208	3.433761
H	2.976903	-3.178664	4.077729
H	2.836887	-4.084446	2.591658
H	3.878128	-2.655512	2.658891
H	-5.541193	1.549445	0.959489
H	-5.005118	-0.047156	1.468830
H	-3.827362	1.241470	1.254971
H	-6.697998	0.363801	-0.925387
H	-5.843986	-0.649303	-2.084303
H	-6.061282	-1.191538	-0.421825
H	-5.381673	2.356016	-1.183487
H	-3.631284	2.285778	-1.373621
H	-4.675879	1.492049	-2.540386
H	-1.358774	-1.868650	-4.198034
H	-0.517472	-1.625674	-2.681343
H	-1.805479	-2.823577	-2.786774

H	-1.657783	0.489510	-4.346490
H	-2.567052	1.444222	-3.177140
H	-0.968654	0.846604	-2.757002
H	-3.442171	-1.094250	-4.677271
H	-4.282121	-1.909800	-3.363072
H	-4.473447	-0.171262	-3.599485
H	4.135986	-1.568973	-3.763366
H	4.896473	-1.323715	-2.195677
H	3.353259	-2.168920	-2.318093
H	1.596812	0.723844	-3.970160
H	1.802352	-1.023098	-3.972486
H	0.990567	-0.219924	-2.631834
H	4.143767	0.542610	-4.498755
H	3.614451	1.958129	-3.609167
H	5.067552	1.094421	-3.104953
H	1.132079	3.851210	-2.074348
H	1.884225	2.622391	-3.066427
H	0.574594	2.176008	-1.966105
H	1.593914	4.158349	0.230224
H	0.974481	2.553703	0.636154
H	2.558447	3.049841	1.198060
H	3.302299	4.519839	-1.419236
H	4.386141	3.565042	-0.421453
H	4.243995	3.223175	-2.145693
H	5.729078	-0.836977	1.454545
H	4.030715	-1.292203	1.255857
H	5.176677	-1.525486	-0.064089
H	5.371162	1.234275	2.163737
H	4.570967	2.518124	1.271645
H	3.605669	1.286109	2.084793
H	6.712397	0.984776	0.123345
H	6.063116	0.308247	-1.364190
H	5.768921	2.002483	-0.961464
H	-3.293773	4.763214	1.050691
H	-3.886156	3.268003	0.354059
H	-3.704343	3.414399	2.093047
H	-1.804731	4.579604	-0.682978
H	-0.356796	3.586227	-0.631013
H	-1.855772	2.888409	-1.192116
H	-1.015005	5.205515	1.401125
H	-1.360798	4.310992	2.866330
H	0.089477	3.940496	1.926687

(t*B*u$_3$Si)(TMS$_2$N)P•

68

E= -1977.749337

C	-4.269990	-1.494802	-0.910722
Si	-2.588947	-1.514006	-0.062472
C	-2.836019	-1.902767	1.757381
N	-1.756794	0.036458	-0.280538
Si	-2.702446	1.535411	-0.156629
C	-3.923799	1.407631	1.265706
P	-0.324121	0.183470	-1.266380
Si	1.654899	0.009562	-0.017911
C	2.531649	1.755421	-0.166927
C	4.058154	1.654906	0.015487
C	2.668692	-1.329061	-1.023288
C	3.938495	-1.786463	-0.282030
C	1.394915	-0.497527	1.835774
C	2.664749	-0.305566	2.685535

C	1.826192	-2.584950	-1.308842
C	3.079748	-0.781976	-2.404425
C	-1.662158	-2.926384	-0.871743
C	-1.607100	2.997521	0.251497
C	-3.592672	1.901029	-1.766689
C	0.255710	0.327894	2.466747
C	0.992912	-1.977899	1.938443
C	2.249015	2.405326	-1.536750
C	2.023004	2.748555	0.892334
H	-2.231148	3.894994	0.315667
H	-1.120713	2.862665	1.220567
H	-0.840377	3.184344	-0.503798
H	2.468496	3.732996	0.701337
H	0.941676	2.872067	0.857700
H	2.303313	2.463241	1.907409
H	-4.453347	2.362770	1.345979
H	-4.676970	0.625937	1.154794
H	-3.397267	1.242612	2.210136
H	-4.095688	2.871996	-1.717056
H	-2.878473	1.935977	-2.594817
H	-4.342967	1.143344	-2.003422
H	2.792257	3.356484	-1.603403
H	2.562358	1.791067	-2.381089
H	1.186303	2.624729	-1.669272
H	4.487453	2.664886	0.010132
H	4.334999	1.189882	0.965009
H	4.544253	1.097525	-0.786243
H	0.086021	-0.017680	3.494922
H	0.489761	1.391549	2.519240
H	-0.677802	0.213341	1.913353
H	2.474063	-0.664233	3.705221
H	3.518345	-0.865683	2.297119
H	2.960493	0.741815	2.764964
H	0.718601	-2.211075	2.975095
H	0.131228	-2.215832	1.312851
H	1.808583	-2.648984	1.662520
H	3.552447	-1.586196	-2.982003
H	2.216913	-0.434219	-2.981090
H	3.800234	0.034251	-2.343657
H	2.430767	-3.301109	-1.879621
H	1.497404	-3.092980	-0.402437
H	0.943826	-2.352681	-1.909276
H	4.506705	-2.471244	-0.924587
H	4.602150	-0.961792	-0.017778
H	3.700538	-2.332291	0.634152
H	-3.288892	-2.894505	1.861229
H	-1.892077	-1.906849	2.306530
H	-3.499774	-1.183548	2.242067
H	-2.298775	-3.815429	-0.808487
H	-1.470910	-2.732813	-1.930721
H	-0.713357	-3.170686	-0.395825
H	-4.748884	-2.469055	-0.766405
H	-4.958927	-0.735089	-0.536902
H	-4.151529	-1.340623	-1.987390

[(t*B*u$_3$Si)(TMS$_2$N)P•]$_2$

136

E= -3955.526146

C	0.714168	-3.099315	3.324216
Si	1.972206	-1.869588	2.633962

C	1.977197	-0.305562	3.649217
N	1.763658	-1.561175	0.863403
Si	2.444496	-2.923576	-0.093311
C	4.338769	-3.025477	-0.097416
C	3.648426	-2.618113	3.087023
P	1.111672	0.037071	0.430362
Si	2.597116	1.367342	-0.913492
C	3.076939	0.719302	-2.690048
C	1.815956	0.235915	-3.432993
P	-1.089392	0.455072	-0.259547
N	-1.821716	1.464562	1.006067
Si	-1.639372	1.231464	2.757691
C	-0.241725	2.290592	3.439403
Si	-2.678720	-1.214343	-0.967008
C	-4.489039	-0.552712	-0.681381
C	-4.766232	0.668876	-1.573005
C	-2.548274	-3.003169	-0.200133
C	-3.243261	-3.108192	1.168733
C	-2.273495	-1.243089	-2.881692
C	-0.991238	-2.059522	-3.085138
C	-3.159663	-4.095102	-1.101681
C	-1.086997	-3.376860	0.036547
C	1.963738	-4.580718	0.677990
C	1.962964	-3.080816	-1.901982
C	1.757358	3.113717	-1.112792
C	0.961287	3.535349	0.127195
C	4.164249	1.540646	0.244994
C	4.678562	0.184576	0.744301
C	2.792094	4.229146	-1.373777
C	0.786565	3.122945	-2.304931
C	5.351154	2.217639	-0.470198
C	3.821581	2.373688	1.495038
C	3.775355	1.769445	-3.577725
C	4.056884	-0.455848	-2.576063
Si	-2.602448	3.019898	0.554727
C	-2.403813	3.471090	-1.257507
C	-4.456566	3.102759	0.903489
C	-1.964675	4.476138	1.575405
C	-3.167507	1.749723	3.734296
C	-1.456633	-0.583457	3.154972
C	-3.384259	-1.878780	-3.739500
C	-1.996278	0.155652	-3.469430
C	-5.547049	-1.610047	-1.064444
C	-4.748331	-0.175107	0.788167
H	-0.933652	-0.728739	4.102822
H	-2.452076	-1.020387	3.262541
H	-0.923826	-1.134357	2.382019
H	-3.004602	-4.083127	1.611637
H	-2.899289	-2.344683	1.863805
H	-4.329378	-3.048619	1.100587
H	-2.962958	1.490779	4.779859
H	-3.389171	2.817653	3.706884
H	-4.062470	1.200044	3.431944
H	0.022322	1.973772	4.453231
H	0.651916	2.240572	2.815546
H	-0.555101	3.333996	3.500442
H	-1.047537	-4.347679	0.544189
H	-0.530043	-3.475942	-0.891508
H	-0.564662	-2.647875	0.656224
H	-3.098136	-5.056674	-0.576531

H	-4.210895	-3.920879	-1.334855
H	-2.619857	-4.212653	-2.042240
H	2.173442	-5.339468	-0.084523
H	2.562960	-4.837463	1.552943
H	0.914853	-4.668407	0.949855
H	2.800168	-3.601709	-2.379747
H	1.078808	-3.703012	-2.037015
H	1.817000	-2.153389	-2.445007
H	4.574481	-4.092465	-0.187163
H	4.807734	-2.527588	-0.945689
H	4.820059	-2.663264	0.807654
H	0.456410	-2.810822	4.347983
H	-0.215630	-3.165945	2.760464
H	1.150192	-4.100310	3.365200
H	2.101642	-0.602175	4.696839
H	2.822121	0.333485	3.383674
H	1.072141	0.280845	3.565838
H	3.602250	-2.762241	4.173074
H	3.884289	-3.586063	2.646562
H	4.478299	-1.935595	2.889167
H	-5.737260	0.291662	0.876451
H	-4.744693	-1.046550	1.441348
H	-4.012248	0.528513	1.164778
H	-6.539998	-1.152425	-0.970618
H	-5.450227	-1.963763	-2.091572
H	-5.535393	-2.477476	-0.404375
H	-5.747511	1.089926	-1.321969
H	-4.026904	1.460030	-1.460480
H	-4.800524	0.396106	-2.628739
H	-0.602219	-1.911692	-4.099938
H	-0.213328	-1.748829	-2.389642
H	-1.147803	-3.130921	-2.950129
H	-1.804901	0.052463	-4.545050
H	-2.833393	0.844173	-3.352084
H	-1.119624	0.619175	-3.018202
H	-3.010642	-2.000714	-4.764075
H	-3.692565	-2.864130	-3.388222
H	-4.272994	-1.247574	-3.799526
H	4.203615	-0.915533	-3.561202
H	5.037613	-0.139122	-2.215876
H	3.691892	-1.228776	-1.909968
H	1.255596	1.059097	-3.874208
H	2.093779	-0.442388	-4.248897
H	1.127010	-0.293633	-2.776487
H	4.058859	1.291014	-4.523817
H	3.134536	2.614077	-3.829212
H	4.689354	2.160614	-3.127042
H	0.274526	4.090492	-2.340170
H	1.298212	3.009889	-3.259389
H	0.026418	2.345860	-2.217376
H	0.507875	4.515565	-0.063873
H	0.160868	2.836479	0.358763
H	1.577967	3.631013	1.020729
H	2.247376	5.154812	-1.597924
H	3.416564	4.433189	-0.503386
H	3.446232	4.027411	-2.222637
H	5.534881	0.345379	1.412046
H	3.919826	-0.358408	1.300868
H	5.016953	-0.456198	-0.067128
H	4.652836	2.310748	2.208250

H	3.676467	3.430575	1.268265
H	2.923329	2.007913	1.997557
H	6.158628	2.367636	0.257532
H	5.757443	1.594424	-1.269436
H	5.110437	3.192661	-0.892182
H	-4.719178	4.164622	0.825960
H	-5.068094	2.565232	0.182320
H	-4.742430	2.770901	1.900969
H	-3.338461	3.927115	-1.598862
H	-1.610921	4.208931	-1.379485
H	-2.174929	2.629574	-1.910040
H	-2.334633	5.381962	1.081361
H	-2.371837	4.481308	2.589366
H	-0.881146	4.553499	1.640587

(t*B*u$_3$Si)(PFS$_2$N)P•

68

E= -3764.025626

C	2.091649	-2.060224	-1.849818
F	3.350615	-2.514246	-1.852162
F	1.313789	-3.049955	-2.323273
F	2.021451	-1.050463	-2.731454
Si	1.488770	-1.502804	-0.077425
C	0.531791	-3.055693	0.585135
F	1.410885	-4.078966	0.585551
N	0.599118	-0.007654	-0.148139
P	-0.911846	-0.309016	-1.128058
Si	-2.984946	-0.029961	-0.024851
C	-3.525333	1.821306	-0.211451
C	-3.103753	2.404171	-1.575616
C	2.932601	-1.398854	1.245853
F	4.058227	-1.981762	0.834936
Si	1.386054	1.527796	0.008213
C	1.396277	2.151700	1.856360
F	2.534793	2.765889	2.193481
C	3.175934	1.474434	-0.799215
F	4.086624	2.167132	-0.116112
C	0.653416	3.005880	-1.025199
F	0.414798	2.662952	-2.300780
F	3.646430	0.208340	-0.924831
F	3.136281	1.967744	-2.043850
F	3.238454	-0.117469	1.570718
F	2.568884	-1.990066	2.390910
F	0.094908	-2.905205	1.844195
F	-0.518501	-3.440668	-0.153681
C	-4.008280	-1.201800	-1.199162
C	-3.340147	-2.575315	-1.409981
C	-2.980618	-0.625614	1.815852
C	-1.729454	-0.098146	2.541139
C	-5.411932	-1.445296	-0.608605
C	-4.189052	-0.580551	-2.597346
F	-0.461493	3.566690	-0.549169
F	1.591332	3.975074	-1.026965
F	0.397167	3.038861	2.047694
F	1.194085	1.154776	2.728691
C	-4.235683	-0.140138	2.567871
C	-2.969532	-2.159908	1.925929
C	-5.051465	2.000978	-0.092950
C	-2.870401	2.663997	0.898128
H	-3.035757	3.727859	0.694588

H	-3.294114	2.453437	1.880413
H	-1.793758	2.518763	0.964017
H	-3.673332	1.979384	-2.400841
H	-3.284918	3.485124	-1.576561
H	-2.047328	2.258634	-1.799833
H	-5.283482	3.072877	-0.105126
H	-5.586581	1.548712	-0.930369
H	-5.459449	1.590783	0.832929
H	-4.669982	-1.315616	-3.253354
H	-4.830543	0.301579	-2.580119
H	-3.238889	-0.304294	-3.065134
H	-3.996877	-3.192527	-2.034892
H	-2.382682	-2.497570	-1.930816
H	-3.170947	-3.117885	-0.481730
H	-6.001896	-2.032317	-1.323039
H	-5.378438	-2.014158	0.322003
H	-5.959919	-0.520957	-0.417387
H	-1.738463	-0.443262	3.581675
H	-0.809623	-0.470293	2.088609
H	-1.674822	0.990515	2.562661
H	-2.831413	-2.438403	2.977117
H	-3.909752	-2.604270	1.596141
H	-2.162288	-2.621527	1.364306
H	-4.224383	-0.562300	3.579851
H	-4.278683	0.943937	2.674200
H	-5.160787	-0.471344	2.088804

(TMS$_3$Si)(Ph$_2$N)P•

64

E= -2376.690724

C	3.103665	-2.059611	0.171500
C	3.109034	-0.850103	-0.519714
C	4.289897	-0.402041	-1.114412
C	5.447713	-1.157078	-1.011960
C	5.440086	-2.368728	-0.327522
C	4.264940	-2.816927	0.260122
N	1.909038	-0.086174	-0.628701
P	0.550832	-0.914150	-1.314616
Si	-1.286772	-0.225363	-0.151718
Si	-0.847908	0.426573	2.065461
C	-0.612060	2.286426	2.208668
C	1.985710	1.294810	-0.339659
C	1.256905	2.221071	-1.084689
C	1.340556	3.577195	-0.797903
C	2.160080	4.032647	0.225672
C	2.894553	3.112428	0.967558
C	2.808394	1.756796	0.694984
Si	-2.370424	-2.322515	-0.068795
C	-1.442881	-3.446603	1.128764
Si	-2.801639	1.262348	-1.168442
C	-3.590848	0.450907	-2.675474
C	-4.161111	-2.156424	0.501096
C	-2.361191	-3.133555	-1.769921
C	-4.143782	1.625112	0.109033
C	-2.066101	2.915426	-1.689930
C	-2.342103	-0.043434	3.120742
C	0.654218	-0.498209	2.722416
H	-1.888434	-4.446630	1.141430
H	-0.396450	-3.550137	0.826409
H	-1.462509	-3.059065	2.151160

H	-2.865069	-4.104947	-1.727114
H	-2.877539	-2.520058	-2.513120
H	-1.340199	-3.300585	-2.124333
H	-4.615893	-3.147760	0.598669
H	-4.238207	-1.655043	1.469375
H	-4.756467	-1.589478	-0.220448
H	0.859025	-0.213336	3.759447
H	0.482963	-1.578459	2.697604
H	1.545721	-0.287244	2.129043
H	-2.190417	0.292915	4.151922
H	-3.259224	0.422581	2.749677
H	-2.500257	-1.125006	3.144144
H	-0.441877	2.566874	3.253517
H	0.237794	2.637986	1.621636
H	-1.503580	2.818789	1.863079
H	-4.278357	1.145227	-3.169410
H	-2.835431	0.147767	-3.406255
H	-4.160068	-0.439816	-2.395122
H	-2.871843	3.582019	-2.015415
H	-1.540738	3.395668	-0.860813
H	-1.365961	2.810842	-2.522915
H	-4.930575	2.243392	-0.335720
H	-4.608371	0.711046	0.487123
H	-3.735821	2.172073	0.964195
H	4.289737	0.537879	-1.654141
H	6.359396	-0.803184	-1.479752
H	6.346534	-2.958112	-0.253562
H	4.249788	-3.756372	0.800659
H	2.187928	-2.392784	0.645519
H	3.383716	1.047683	1.278032
H	3.534418	3.451704	1.774182
H	2.228463	5.091677	0.443634
H	0.767613	4.279704	-1.392364
H	0.644801	1.872438	-1.907119

$[(TMS_3Si)(Ph_2N)P•]_2$

128

E= -4753.465456

C	0.286574	-3.521867	1.354821
C	1.248928	-2.574807	1.681041
C	2.506102	-2.995404	2.112404
C	2.792004	-4.350098	2.206283
C	1.832697	-5.296126	1.861277
C	0.578953	-4.876553	1.434393
N	0.961568	-1.182836	1.517163
C	1.146049	-0.324517	2.620871
C	1.133859	-0.839959	3.924227
C	1.262244	0.002345	5.017393
C	1.400307	1.374607	4.848494
C	1.396105	1.888422	3.560674
C	1.264600	1.055889	2.459382
P	0.774380	-0.711796	-0.149880
Si	2.796994	0.123446	-0.871352
Si	2.545026	1.655360	-2.672148
C	2.410604	3.383021	-1.942932
P	-0.915943	0.571733	-0.642842
Si	-2.745928	-0.776468	-0.818332
Si	-2.113150	-2.405086	-2.416020
C	-1.405045	-3.962017	-1.630408
N	-1.314846	1.892465	0.405194
C	-1.981129	1.784221	1.652523

C	-2.795126	2.827773	2.108078
C	-3.410887	2.749849	3.347963
C	-3.239285	1.632211	4.157610
C	-2.463356	0.580699	3.692694
C	-1.852269	0.649163	2.448784
C	-0.892780	3.189026	-0.015307
C	-0.210036	4.024835	0.870979
C	0.189976	5.293046	0.474892
C	-0.075290	5.745131	-0.812474
C	-0.743861	4.912791	-1.700487
C	-1.151877	3.645453	-1.307517
Si	-3.990670	-1.751049	0.938108
C	-5.154454	-0.473786	1.686832
Si	-4.229986	0.685546	-1.933366
C	-5.893286	-0.150945	-2.243580
C	-5.068142	-3.056001	0.093258
C	-3.029276	-2.664757	2.280297
Si	3.840316	-1.901465	-1.586847
C	5.190543	-2.472045	-0.398014
Si	4.271176	1.205690	0.638365
C	4.704062	0.173613	2.153573
C	2.588496	-3.298478	-1.745900
C	4.660953	-1.686225	-3.271266
C	5.859240	1.444869	-0.364444
C	3.727655	2.928027	1.213397
C	4.086127	1.657386	-3.764567
C	1.075820	1.323176	-3.795899
C	-4.565384	2.243340	-0.931121
C	-3.475374	1.153037	-3.599668
C	-3.643934	-2.899775	-3.405336

C	-0.840634	-1.720799	-3.619758
H	5.672014	-3.365318	-0.810242
H	5.965435	-1.715969	-0.247596
H	4.778060	-2.740200	0.576480
H	3.927324	-1.457067	-4.048855
H	5.412274	-0.892920	-3.271727
H	5.158387	-2.622087	-3.547497
H	2.178760	-3.589857	-0.775878
H	1.750911	-3.024364	-2.392557
H	3.082687	-4.174091	-2.180826
H	1.166569	0.359579	-4.303927
H	0.137584	1.324314	-3.237837
H	1.031693	2.104907	-4.562183
H	4.097722	0.815111	-4.458363
H	4.082524	2.578264	-4.358185
H	5.017090	1.634936	-3.193658
H	2.148300	4.098394	-2.728911
H	1.648870	3.453116	-1.165667
H	3.364213	3.695959	-1.509079
H	5.481687	0.694649	2.722779
H	3.844188	0.040510	2.813783
H	5.096675	-0.807619	1.877863
H	4.356765	3.693819	0.750452
H	2.691584	3.169172	0.969994
H	3.843325	3.006043	2.297849
H	6.192543	0.529678	-0.861465
H	5.722682	2.212505	-1.131360
H	6.666689	1.779194	0.295737
H	-1.194654	-0.815288	-4.118496
H	0.087580	-1.472583	-3.099754

H	-0.614260	-2.468880	-4.387200
H	-2.026986	-4.339941	-0.813147
H	-1.330490	-4.751641	-2.385562
H	-0.402338	-3.774351	-1.241343
H	-4.028015	-2.066245	-3.999455
H	-3.380803	-3.705615	-4.098750
H	-4.452990	-3.261133	-2.765631
H	-2.143644	-2.139049	2.638451
H	-3.690471	-2.828655	3.137820
H	-2.712916	-3.647994	1.920521
H	-5.684354	-0.905638	2.542335
H	-4.633972	0.420860	2.031436
H	-5.905593	-0.169011	0.952460
H	-5.742809	-3.507621	0.828557
H	-5.683230	-2.626669	-0.701874
H	-4.466352	-3.859346	-0.341722
H	-6.529255	0.513069	-2.838653
H	-5.792928	-1.093231	-2.786769
H	-6.419081	-0.355821	-1.307133
H	-4.040125	1.965395	-4.068327
H	-2.434651	1.474583	-3.499505
H	-3.485919	0.300411	-4.285029
H	-5.318255	2.854962	-1.439882
H	-4.942513	1.999620	0.065251
H	-3.668536	2.851578	-0.802264
H	1.015062	-1.904096	4.082104
H	1.247217	-0.424249	6.014276
H	1.499359	2.030090	5.705048
H	1.500299	2.956130	3.402774
H	1.226186	1.485592	1.467790
H	3.251739	-2.253877	2.373907
H	3.775048	-4.667146	2.535916
H	2.062848	-6.353265	1.923484
H	-0.176615	-5.603079	1.157454
H	-0.681197	-3.181925	1.012953
H	-2.934005	3.707682	1.492206
H	-4.032878	3.573177	3.681090
H	-3.712620	1.579970	5.130754
H	-2.320559	-0.306254	4.299365
H	-1.253435	-0.179224	2.101848
H	-1.671650	3.002172	-2.006066
H	-0.955771	5.249363	-2.708954
H	0.241881	6.734029	-1.121379
H	0.720072	5.927210	1.176464
H	-0.019322	3.678577	1.879360

(TMS$_3$Si)(PFP$_2$N)P•

64

E= -3368.987598

C	2.755622	-1.801087	0.470704
C	2.416600	-0.743750	-0.370537
C	3.405594	-0.237404	-1.211397
C	4.691070	-0.751381	-1.207580
C	5.002597	-1.809232	-0.367324
C	4.032276	-2.338103	0.469801
N	1.106379	-0.208427	-0.378330
P	-0.122328	-1.242297	-1.070997
Si	-2.126266	-0.677220	-0.160188
Si	-2.087777	-0.003972	2.092934
C	-2.008426	1.878212	2.182265

F	3.115879	0.759927	-2.042059
F	5.617844	-0.250949	-2.015363
F	6.227584	-2.314285	-0.364238
F	4.336184	-3.344525	1.279793
F	1.855130	-2.307260	1.303227
C	0.998068	1.159733	-0.069662
C	0.482948	2.076562	-0.985172
C	0.393924	3.426269	-0.688819
C	0.859178	3.898053	0.529008
C	1.415520	3.014646	1.440571
C	1.481530	1.665662	1.137025
F	0.100121	1.662567	-2.188013
F	2.029075	0.843534	2.029744
F	1.861889	3.459402	2.608017
F	0.778513	5.188742	0.816884
F	-0.100019	4.276593	-1.579310
Si	-3.035549	-2.858602	-0.248714
C	-2.218357	-3.891766	1.097714
Si	-3.545005	0.760412	-1.377623
C	-3.905395	0.010218	-3.065185
C	-4.899715	-2.835678	0.023623
C	-2.690155	-3.645141	-1.925129
C	-5.144184	0.882205	-0.382317
C	-2.905646	2.516471	-1.582987
C	-3.706088	-0.551553	2.892541
C	-0.670165	-0.809825	3.020750
H	-2.540032	-4.936050	1.030568
H	-1.128766	-3.867312	1.003903
H	-2.476680	-3.527273	2.096063
H	-3.138069	-4.643413	-1.968821
H	-3.110786	-3.052642	-2.742332
H	-1.617419	-3.752543	-2.107542
H	-5.276644	-3.862127	0.080992
H	-5.174655	-2.325405	0.950298
H	-5.415885	-2.340104	-0.803511
H	-0.642740	-0.455565	4.056325
H	-0.801216	-1.895344	3.038871
H	0.299189	-0.608255	2.566249
H	-3.754782	-0.181980	3.922260
H	-4.578713	-0.167563	2.357814
H	-3.785053	-1.641557	2.928371
H	-1.791880	2.217969	3.199849
H	-1.244852	2.287745	1.519073
H	-2.967453	2.311449	1.882303
H	-4.555582	0.668720	-3.649959
H	-2.984176	-0.145695	-3.633642
H	-4.407810	-0.956607	-2.968198
H	-3.719168	3.151064	-1.950064
H	-2.566033	2.935838	-0.631859
H	-2.086762	2.579725	-2.299688
H	-5.889573	1.445907	-0.953229
H	-5.571951	-0.093701	-0.143602
H	-4.978445	1.417119	0.557573

[(TMS$_3$Si)(PFP$_2$N)P•]$_2$

128

E= -6738.046812

C	2.892520	-0.775187	1.422895
C	2.938075	0.393042	0.663586
C	4.168728	1.061715	0.651957

C	5.273728	0.592274	1.342126	C	-1.634141	3.810810	0.336664
C	5.195076	-0.585051	2.065660	C	-1.788090	2.446631	0.496601
C	3.995165	-1.269591	2.096590	F	-2.113554	1.415296	-2.932047
N	1.813726	0.891121	-0.018409	F	-1.746750	4.051622	-3.258302
C	1.618617	2.292227	0.031937	F	-1.466439	5.666992	-1.090030
C	1.578821	2.957157	1.255754	F	-1.477127	4.593950	1.400709
C	1.504384	4.334797	1.353156	F	-1.771019	1.960338	1.732995
C	1.437799	5.099448	0.200743	Si	-1.270500	-1.097228	2.498241
C	1.448223	4.467235	-1.031941	Si	-0.806923	-3.420378	2.795402
C	1.542662	3.088308	-1.109797	C	-0.791576	-4.272450	1.121903
F	1.651653	2.254930	2.385659	Si	-3.631342	-0.968740	2.784176
F	1.470317	4.915241	2.543843	C	-4.374089	0.737201	2.523121
F	1.340970	6.416329	0.275981	Si	-0.232582	0.014121	4.321522
F	1.362747	5.183794	-2.144405	C	1.641585	-0.132660	4.394282
F	1.588397	2.545374	-2.321897	C	-4.475001	-2.233317	1.649232
F	4.313266	2.206995	-0.014822	C	-4.100238	-1.380040	4.566350
F	6.415499	1.264159	1.290142	Si	3.307462	-3.296980	-1.221394
F	6.255187	-1.057364	2.704533	C	4.989129	-2.496050	-0.894718
F	3.896422	-2.391858	2.800431	Si	0.183298	-2.746508	-3.430951
F	1.743737	-1.440617	1.551863	C	-0.507871	-4.245349	-2.535897
P	0.658967	-0.050628	-0.963420	C	2.713854	-4.272534	0.269594
Si	1.827600	-1.671746	-2.102026	C	3.550361	-4.567993	-2.595250
Si	3.099954	-0.397500	-3.642763	C	-3.491178	-0.228163	-0.963954
C	1.926539	0.527092	-4.788619	C	-4.621586	0.608345	-1.007685
P	-1.020844	-0.919302	0.183086	C	-5.845440	0.212469	-1.517888
N	-2.269277	0.222505	-0.419453	C	-6.017502	-1.065906	-2.013400
C	-1.984451	1.597105	-0.586578	C	-4.936123	-1.922774	-1.985248
C	-1.946521	2.175135	-1.855152	C	-3.709888	-1.510664	-1.488543
C	-1.780553	3.536887	-2.036665	F	-4.573683	1.862690	-0.539772
C	-1.641109	4.361959	-0.932937	F	-6.858715	1.069721	-1.522273

F	-7.187682	-1.456651	-2.501117
F	-5.058781	-3.162142	-2.446249
F	-2.758580	-2.441916	-1.509258
C	-1.159981	-1.535362	-3.941423
C	0.988224	-3.369096	-5.020347
C	4.291628	0.796116	-2.814433
C	4.168641	-1.560028	-4.676263
C	-0.759435	1.802839	4.556511
C	-0.855616	-0.947983	5.826914
C	0.824425	-3.743492	3.680558
C	-2.132564	-4.261717	3.841817
H	-0.376031	2.163434	5.516871
H	-0.382438	2.460476	3.774160
H	-1.848980	1.892532	4.576439
H	1.971831	0.252868	5.364891
H	1.960324	-1.175063	4.326260
H	2.149309	0.443685	3.623307
H	-0.272128	-0.631094	6.698238
H	-1.905942	-0.752539	6.044448
H	-0.729622	-2.028655	5.725833
H	-5.452287	0.678084	2.708540
H	-3.952145	1.451081	3.235119
H	-4.227468	1.143169	1.526746
H	-5.180130	-1.564328	4.590890
H	-3.602483	-2.257208	4.978184
H	-3.903618	-0.529428	5.223887
H	-5.245144	-1.748240	1.041848
H	-3.782038	-2.737326	0.972019
H	-4.968022	-3.004874	2.247609
H	0.994402	-4.824455	3.726463
H	1.678680	-3.293429	3.177188
H	0.807450	-3.370113	4.708069
H	-0.533661	-5.330336	1.236660
H	-1.770408	-4.207397	0.639442
H	-0.066018	-3.814363	0.448413
H	-1.915940	-5.335124	3.869710
H	-2.134768	-3.901847	4.873924
H	-3.137498	-4.137129	3.432108
H	5.549341	-3.047775	-0.133374
H	5.586519	-2.495949	-1.810953
H	4.903500	-1.459367	-0.567960
H	1.873653	-4.911689	-0.013475
H	3.526096	-4.922251	0.612202
H	2.396456	-3.661298	1.111593
H	4.306373	-5.292513	-2.273407
H	2.630378	-5.121482	-2.800575
H	3.895547	-4.120401	-3.529234
H	-1.267829	-4.732653	-3.155331
H	0.279184	-4.977898	-2.333120
H	-0.970828	-3.973541	-1.588951
H	-1.997678	-2.077123	-4.392747
H	-1.544183	-0.951770	-3.106577
H	-0.774380	-0.828182	-4.680225
H	0.234896	-3.920769	-5.593276
H	1.342906	-2.549802	-5.650267
H	1.824030	-4.047268	-4.835260
H	4.903426	1.283156	-3.581077
H	3.776217	1.577091	-2.259576
H	4.970489	0.276766	-2.132467
H	2.472878	1.277503	-5.368748

H	1.453429	-0.159995	-5.495722
H	1.135427	1.037598	-4.235508
H	4.668885	-0.973966	-5.454840
H	4.948329	-2.030056	-4.070920
H	3.596969	-2.348019	-5.169442

(TMS$_3$Si)(t*B*u$_2$N)P•

68

E= -2229.114855

C	0.504214	-2.263149	2.548221
Si	0.745880	-0.426045	2.185452
C	-0.654826	0.581855	2.938764
Si	0.950021	0.006092	-0.117973
Si	2.381051	-1.586068	-1.102415
C	1.593686	-3.275190	-1.384568
P	-0.951800	0.494634	-1.297051
N	-2.347015	0.027377	-0.374636
C	-3.360606	1.145516	-0.302429
C	-2.647939	2.423495	0.188398
Si	2.043937	2.089442	-0.326058
C	1.865908	2.750270	-2.081640
C	2.356648	0.085941	3.027079
C	-2.755178	-1.416660	-0.275167
C	-3.903933	-1.765850	-1.240402
C	-1.584147	-2.322038	-0.681112
C	-3.131621	-1.830120	1.161672
C	-3.997128	1.453088	-1.671900
C	-4.472159	0.888929	0.724856
C	1.256028	3.324778	0.862476
C	3.884727	1.953997	0.068985
C	3.004625	-0.935267	-2.757980
C	3.853494	-1.843992	0.051333
H	-3.388384	3.207565	0.369094
H	-2.112962	2.232122	1.120833
H	-1.936042	2.800810	-0.547267
H	1.691366	4.320263	0.726823
H	0.179045	3.401933	0.688987
H	1.407327	3.034776	1.906229
H	-5.105921	1.778268	0.762997
H	-5.118627	0.050356	0.471737
H	-4.059039	0.731864	1.723197
H	-4.614690	2.353293	-1.600307
H	-3.223945	1.635567	-2.422085
H	-4.631340	0.639622	-2.023432
H	2.368626	3.718778	-2.174506
H	2.308714	2.069740	-2.814147
H	0.814058	2.885028	-2.347704
H	4.348798	2.945382	0.035663
H	4.061734	1.533994	1.062490
H	4.401439	1.322897	-0.659915
H	-0.811568	0.311752	3.988040
H	-0.419848	1.649446	2.895930
H	-1.588713	0.427327	2.394661
H	2.310720	-0.151434	4.095262
H	3.219424	-0.436223	2.604905
H	2.535839	1.160424	2.933585
H	0.524607	-2.433107	3.629878
H	-0.441398	-2.655287	2.168480
H	1.312282	-2.853574	2.105253
H	3.645760	-1.675813	-3.247166

H	2.173905	-0.709465	-3.432987
H	3.588556	-0.019394	-2.630440
H	2.353705	-3.970037	-1.757561
H	1.182770	-3.692908	-0.461364
H	0.788935	-3.232880	-2.122172
H	4.586578	-2.508895	-0.417513
H	4.357493	-0.904623	0.291734
H	3.541117	-2.307484	0.992008
H	-3.175168	-2.921324	1.224154
H	-2.377506	-1.483564	1.868659
H	-4.099218	-1.453701	1.482706
H	-1.906483	-3.364405	-0.623364
H	-1.259200	-2.131120	-1.706344
H	-0.725614	-2.207063	-0.021298
H	-4.101180	-2.840684	-1.192865
H	-4.836021	-1.255863	-1.000770
H	-3.627006	-1.515010	-2.267469

[(TMS$_3$Si)(t*B*u$_2$N)P•]$_2$

136

E= -4458.257904

C	0.314537	3.185586	-2.978429
C	1.607430	2.867780	-2.214336
N	1.300194	2.211987	-0.875902
C	1.233006	3.018467	0.395530
C	0.649597	4.443592	0.240245
C	2.404005	4.189231	-2.127372
C	2.477956	1.956218	-3.100061
P	1.139904	0.480049	-0.880407
Si	2.852126	-0.429481	0.456504
Si	2.984178	-2.742965	-0.065012
C	4.701250	-3.496507	0.181602
P	-0.865393	-0.669195	-0.468820
N	-1.464715	-1.263741	-1.998389
C	-1.795745	-0.349989	-3.148717
C	-2.915170	-0.845805	-4.084113
Si	-2.743496	0.175507	0.688733
Si	-3.773797	2.323607	0.512302
C	-4.564611	2.699835	2.185379
Si	-2.345025	-0.363027	2.977620
C	-3.912045	-0.575416	4.013992
Si	-4.669071	-1.192030	0.319312
C	-6.110552	-0.505781	1.341843
C	-1.501870	-2.048682	2.983484
C	-1.396070	0.938672	3.964049
C	0.336908	2.291560	1.402343
C	2.613384	3.174662	1.046497
Si	4.932315	0.287310	-0.473132
C	6.361635	-0.369958	0.574435
Si	3.092655	-0.419844	2.832459
C	4.458664	-1.635044	3.312679
C	5.111446	-0.435624	-2.211185
C	5.321999	2.130119	-0.560532
C	3.546626	1.236384	3.613351
C	1.573027	-1.062884	3.722832
C	1.848345	-3.735785	1.072554
C	2.617594	-2.975516	-1.902394
C	-1.377747	-2.757855	-2.243549
C	-2.767189	-3.413237	-2.368315
C	-0.558429	-3.130598	-3.495083

C	-0.715129	-3.480647	-1.064961
C	-2.329165	0.973436	-2.590917
C	-0.565383	-0.078331	-4.038612
C	-5.395841	-1.213328	-1.423430
C	-4.414246	-2.966834	0.920580
C	-2.640885	3.758625	0.090173
C	-5.144169	2.417244	-0.788597
H	-2.559517	1.654744	-3.413844
H	-3.249829	0.787884	-2.037477
H	-1.625477	1.474163	-1.928105
H	-5.658922	3.375140	-0.653398
H	-4.740804	2.406228	-1.803984
H	-5.894121	1.629629	-0.713954
H	-3.079415	-0.069190	-4.836149
H	-2.659754	-1.753353	-4.626795
H	-3.854348	-0.995465	-3.556315
H	-0.746879	0.788582	-4.680618
H	0.311022	0.111082	-3.424144
H	-0.344586	-0.918468	-4.693943
H	-3.251083	4.636655	-0.146093
H	-1.982704	4.020685	0.918099
H	-2.024253	3.532587	-0.782232
H	-5.049155	3.681275	2.143844
H	-5.324183	1.964899	2.459264
H	-3.819656	2.730607	2.984789
H	0.196526	4.742707	1.187737
H	1.421731	5.178560	0.025209
H	-0.121577	4.511902	-0.520827
H	0.259184	2.893399	2.310354
H	-0.666387	2.141063	1.003954
H	0.739568	1.317762	1.681437
H	2.509717	3.651263	2.025751
H	3.088669	2.207466	1.192044
H	3.279980	3.795110	0.452239
H	0.546430	3.724552	-3.902594
H	-0.209523	2.276077	-3.247149
H	-0.359580	3.808159	-2.388301
H	2.603820	2.431200	-4.076392
H	3.467699	1.822710	-2.663466
H	2.047361	0.972067	-3.258761
H	2.816875	4.388010	-3.118687
H	1.782661	5.043535	-1.874811
H	3.242688	4.144358	-1.436864
H	-6.450832	-1.495303	-1.334890
H	-5.358869	-0.233918	-1.904524
H	-4.916694	-1.935084	-2.079601
H	-6.919419	-1.243596	1.285379
H	-5.887252	-0.344552	2.394405
H	-6.499082	0.428561	0.931460
H	-5.180191	-3.619924	0.489244
H	-3.437098	-3.379489	0.663645
H	-4.515942	-3.014449	2.008327
H	-1.228041	0.557299	4.977007
H	-0.431874	1.224761	3.552577
H	-2.001440	1.846136	4.050813
H	-1.115464	-2.298638	3.975748
H	-2.229009	-2.814274	2.699976
H	-0.680352	-2.093803	2.267388
H	-3.587621	-0.793848	5.037778
H	-4.522010	0.330287	4.051667

H	-4.538453	-1.406990	3.687464
H	3.825265	1.055294	4.657061
H	4.391269	1.725396	3.120729
H	2.705089	1.932305	3.608801
H	1.397108	-2.114517	3.488405
H	1.723657	-0.977067	4.804495
H	0.671227	-0.512471	3.468071
H	4.579546	-1.579515	4.400405
H	4.178052	-2.662850	3.070857
H	5.427589	-1.428028	2.858923
H	1.667863	-4.735952	0.665486
H	2.330835	-3.858013	2.047368
H	0.881838	-3.256742	1.234560
H	2.169593	-3.950206	-2.114389
H	1.950855	-2.197392	-2.277059
H	3.551684	-2.904032	-2.465489
H	4.616509	-4.570323	-0.021830
H	5.436815	-3.091121	-0.516442
H	5.090574	-3.383223	1.194498
H	6.306455	2.232531	-1.030570
H	4.610997	2.708879	-1.145812
H	5.382833	2.572369	0.436634
H	5.883960	0.107715	-2.765054
H	5.415826	-1.484445	-2.164701
H	4.183535	-0.385622	-2.784374
H	7.295123	-0.153046	0.043039
H	6.408715	0.145648	1.538012
H	6.325339	-1.442903	0.760345
H	-2.639090	-4.495195	-2.467320
H	-3.352616	-3.233132	-1.470002
H	-3.337928	-3.079181	-3.229663
H	-0.683139	-4.549783	-1.291162
H	0.298934	-3.136600	-0.904326
H	-1.262056	-3.344730	-0.130953
H	-0.392088	-4.211171	-3.498453
H	-1.070262	-2.888636	-4.425683
H	0.414182	-2.644161	-3.498455

(TMS$_3$Si)(TMS$_2$N)P•

68

E= -2732.048121

P	0.680570	-0.399221	-1.385472
Si	-1.204171	-0.015722	-0.131523
Si	-1.022274	-1.088361	1.960378
C	-1.077117	-2.959626	1.724570
N	2.098485	0.000314	-0.469636
Si	2.616889	1.672379	-0.208757
C	1.528116	2.839400	-1.184187
Si	3.245153	-1.342327	-0.268128
C	4.340985	-1.022898	1.226389
C	4.282446	-1.562951	-1.813762
C	2.334370	-2.938766	0.095802
Si	-2.140833	2.135377	0.147113
C	-1.246859	3.124123	1.482410
Si	-2.794030	-1.265594	-1.356623
C	-3.436753	-0.270503	-2.823996
C	-2.218417	3.117179	-1.461236
C	-3.930781	1.912674	0.705213
C	2.555569	2.128318	1.607321
C	4.357468	1.936519	-0.872931

C	-2.020667	-2.860849	-1.993867
C	-4.265783	-1.714772	-0.262232
C	0.569684	-0.614465	2.847506
C	-2.476237	-0.611966	3.064982
H	4.635251	2.984729	-0.719415
H	5.127998	1.323428	-0.402650
H	4.383829	1.743210	-1.949389
H	1.885061	3.865678	-1.049505
H	1.552153	2.610284	-2.253276
H	0.490706	2.806014	-0.858104
H	2.896870	3.158275	1.753808
H	1.536174	2.053841	1.992711
H	3.187717	1.473914	2.212719
H	4.934486	-2.437567	-1.725145
H	3.637096	-1.712594	-2.684541
H	4.911502	-0.690764	-2.007854
H	3.061333	-3.752598	0.187320
H	1.795679	-2.869683	1.044211
H	1.622477	-3.210595	-0.687302
H	5.031646	-1.866249	1.331886
H	4.943244	-0.115003	1.167997
H	3.743919	-0.973565	2.141549
H	-1.790121	4.052522	1.687389
H	-1.194417	2.556431	2.416666
H	-0.227089	3.392091	1.197694
H	-2.840978	2.596070	-2.193342
H	-2.676145	4.093748	-1.269799
H	-1.243848	3.288780	-1.920866
H	-4.363038	2.891681	0.938446
H	-4.536589	1.466390	-0.088283
H	-4.021578	1.285208	1.594421
H	-4.085004	-0.890962	-3.451417
H	-4.022625	0.591975	-2.493140
H	-2.617707	0.098594	-3.447850
H	-2.764235	-3.450322	-2.540647
H	-1.184542	-2.657174	-2.668200
H	-1.642865	-3.473986	-1.171166
H	-5.015151	-2.253361	-0.852017
H	-3.970374	-2.363290	0.566711
H	-4.747258	-0.830448	0.162142
H	0.757345	-1.289457	3.688931
H	1.426221	-0.650080	2.171976
H	0.506962	0.403071	3.243175
H	-0.847046	-3.465721	2.668113
H	-2.071749	-3.285283	1.408446
H	-0.362802	-3.304546	0.974451
H	-2.411245	-1.147525	4.018092
H	-2.481908	0.459294	3.285824
H	-3.433716	-0.867362	2.602437

[(TMS$_3$Si)(TMS$_2$N)P•]$_2$

136

E= -5464.142594

C	0.025665	-3.341337	-3.528713
Si	-1.459554	-2.390500	-2.858700
C	-2.878478	-3.612775	-3.090766
C	-1.813011	-0.949674	-3.994902
N	-1.283118	-1.868276	-1.154750
P	-1.121618	-0.150883	-0.805163
P	0.839905	0.770638	0.089817

Si	2.619607	-0.644230	0.721015
Si	2.554304	-0.707587	3.109258
C	1.999267	0.979056	3.743263
Si	-1.706718	-3.077144	0.082961
C	-0.771482	-2.652950	1.636801
C	-3.559841	-3.161437	0.354055
C	-1.289254	-4.863716	-0.390568
Si	-2.820866	0.507563	0.663031
Si	-4.755686	0.545097	-0.719270
C	-4.543367	1.901393	-2.016635
Si	-3.206131	-0.168787	2.927035
C	-3.888143	-1.884002	3.307988
Si	-2.546034	2.838250	1.027449
C	-1.512961	3.173629	2.567034
C	-1.610091	0.031706	3.926391
C	-4.493929	1.009664	3.653704
N	1.423512	1.851307	-1.190537
Si	1.903790	3.525457	-0.777962
C	1.055633	4.827284	-1.856206
Si	1.327696	1.479867	-2.924503
C	1.686512	-0.340964	-3.133450
C	-0.294737	2.093995	-3.645409
C	2.634971	2.338829	-3.981143
Si	4.689633	0.483220	0.403015
C	4.935760	0.923451	-1.408171
Si	3.207636	-2.789449	-0.117649
C	1.756190	-3.911462	-0.480719
C	4.864946	1.982964	1.539600
C	6.161865	-0.625358	0.830945
C	4.228678	-2.745948	-1.704153

C	4.237189	-3.648153	1.213177
C	1.509500	-2.059779	3.906039
C	4.276560	-1.018116	3.827649
C	1.546348	3.921824	1.015441
C	3.713560	3.940447	-1.099815
C	-1.765900	3.631976	-0.478806
C	-4.192363	3.730726	1.296442
C	-5.148134	-0.993585	-1.724905
C	-6.273408	0.894013	0.345589
H	1.461170	-0.690119	-4.144472
H	2.756829	-0.482441	-2.959585
H	1.151200	-0.967805	-2.421093
H	4.541743	-3.771733	-1.928342
H	3.638461	-2.391773	-2.552224
H	5.129609	-2.135027	-1.635530
H	2.439516	2.009075	-5.008637
H	2.566884	3.427539	-3.983646
H	3.661021	2.054937	-3.742285
H	-0.403278	1.786015	-4.690152
H	-1.156538	1.738272	-3.081451
H	-0.299024	3.186915	-3.629994
H	2.070669	-4.738969	-1.124678
H	1.342394	-4.338621	0.434020
H	0.965916	-3.364914	-0.993017
H	4.508444	-4.647863	0.856493
H	5.159754	-3.117667	1.453119
H	3.665772	-3.771511	2.137619
H	-0.796376	-5.343189	0.460377
H	-2.210195	-5.418996	-0.585700
H	-0.629341	-4.989581	-1.246725

H	-0.954265	-3.393524	2.420944	H	4.175640	-1.045609	4.918321
H	0.299853	-2.641511	1.431776	H	4.691667	-1.978175	3.512726
H	-1.030168	-1.672247	2.030799	H	4.998848	-0.236998	3.587953
H	-3.776194	-3.893259	1.138467	H	-4.053893	-1.924387	4.390516
H	-4.019407	-2.216702	0.636537	H	-4.844364	-2.080377	2.817744
H	-4.035733	-3.515624	-0.562578	H	-3.197704	-2.686020	3.047283
H	-0.061821	-3.361719	-4.621063	H	-1.678637	0.886812	4.603306
H	0.997075	-2.913924	-3.286048	H	-1.428336	-0.859176	4.534148
H	0.021437	-4.380035	-3.189239	H	-0.734701	0.190779	3.295179
H	-2.149848	-1.355106	-4.954891	H	-4.737379	0.659547	4.662792
H	-2.593582	-0.287936	-3.612956	H	-4.127429	2.033331	3.743013
H	-0.927825	-0.345101	-4.179946	H	-5.421262	1.027277	3.076656
H	-2.894079	-3.850200	-4.160737	H	-1.288250	4.242751	2.641458
H	-2.733306	-4.555330	-2.561808	H	-2.051548	2.890219	3.473819
H	-3.862422	-3.220954	-2.832814	H	-0.568551	2.627561	2.547229
H	5.801885	1.579388	-1.540884	H	-1.458666	4.657656	-0.253420
H	5.104352	0.017900	-1.996117	H	-0.892992	3.071254	-0.809831
H	4.061694	1.427893	-1.808270	H	-2.466060	3.664516	-1.316104
H	7.067170	-0.019818	0.706550	H	-3.965866	4.781643	1.509564
H	6.152155	-0.994645	1.856702	H	-4.830611	3.709283	0.410844
H	6.252295	-1.482243	0.160434	H	-4.769323	3.342613	2.136988
H	5.735675	2.578799	1.245835	H	-5.956008	-0.745369	-2.421617
H	3.994007	2.638635	1.546072	H	-4.286135	-1.302579	-2.315408
H	5.032621	1.649927	2.566885	H	-5.473146	-1.838731	-1.118085
H	1.745611	-2.079763	4.975530	H	-5.352972	1.827521	-2.750108
H	0.439790	-1.894255	3.802059	H	-4.566627	2.908704	-1.598062
H	1.739111	-3.047681	3.496675	H	-3.596028	1.783722	-2.551794
H	1.792363	0.932229	4.817306	H	-7.156766	0.983399	-0.295536
H	2.782851	1.724832	3.583158	H	-6.453989	0.069566	1.042442
H	1.099295	1.335137	3.239342	H	-6.184640	1.814279	0.925502

H	3.809882	5.012253	-0.889499
H	4.431917	3.420568	-0.472504
H	4.001227	3.791049	-2.141608
H	2.112061	4.819086	1.287346
H	0.488876	4.135020	1.165026
H	1.818652	3.122386	1.706335
H	1.204513	5.791318	-1.355840
H	1.527564	4.908511	-2.838204
H	-0.013959	4.696688	-2.001234

(TMS3Si)(PFS2N)P•

68

E= -2732.048121

P	0.680570	-0.399221	-1.385472
Si	-1.204171	-0.015722	-0.131523
Si	-1.022274	-1.088361	1.960378
C	-1.077117	-2.959626	1.724570
N	2.098485	0.000314	-0.469636
Si	2.616889	1.672379	-0.208757
C	1.528116	2.839400	-1.184187
Si	3.245153	-1.342327	-0.268128
C	4.340985	-1.022898	1.226389
C	4.282446	-1.562951	-1.813762
C	2.334370	-2.938766	0.095802
Si	-2.140833	2.135377	0.147113
C	-1.246859	3.124123	1.482410
Si	-2.794030	-1.265594	-1.356623
C	-3.436753	-0.270503	-2.823996
C	-2.218417	3.117179	-1.461236
C	-3.930781	1.912674	0.705213
C	2.555569	2.128318	1.607321
C	4.357468	1.936519	-0.872931
C	-2.020667	-2.860849	-1.993867
C	-4.265783	-1.714772	-0.262232
C	0.569684	-0.614465	2.847506
C	-2.476237	-0.611966	3.064982
H	4.635251	2.984729	-0.719415
H	5.127998	1.323428	-0.402650
H	4.383829	1.743210	-1.949389
H	1.885061	3.865678	-1.049505
H	1.552153	2.610284	-2.253276
H	0.490706	2.806014	-0.858104
H	2.896870	3.158275	1.753808
H	1.536174	2.053841	1.992711
H	3.187717	1.473914	2.212719
H	4.934486	-2.437567	-1.725145
H	3.637096	-1.712594	-2.684541
H	4.911502	-0.690764	-2.007854
H	3.061333	-3.752598	0.187320
H	1.795679	-2.869683	1.044211
H	1.622477	-3.210595	-0.687302
H	5.031646	-1.866249	1.331886
H	4.943244	-0.115003	1.167997
H	3.743919	-0.973565	2.141549
H	-1.790121	4.052522	1.687389
H	-1.194417	2.556431	2.416666
H	-0.227089	3.392091	1.197694
H	-2.840978	2.596070	-2.193342
H	-2.676145	4.093748	-1.269799
H	-1.243848	3.288780	-1.920866

H	-4.363038	2.891681	0.938446
H	-4.536589	1.466390	-0.088283
H	-4.021578	1.285208	1.594421
H	-4.085004	-0.890962	-3.451417
H	-4.022625	0.591975	-2.493140
H	-2.617707	0.098594	-3.447850
H	-2.764235	-3.450322	-2.540647
H	-1.184542	-2.657174	-2.668200
H	-1.642865	-3.473986	-1.171166
H	-5.015151	-2.253361	-0.852017
H	-3.970374	-2.363290	0.566711
H	-4.747258	-0.830448	0.162142
H	0.757345	-1.289457	3.688931
H	1.426221	-0.650080	2.171976
H	0.506962	0.403071	3.243175
H	-0.847046	-3.465721	2.668113
H	-2.071749	-3.285283	1.408446
H	-0.362802	-3.304546	0.974451
H	-2.411245	-1.147525	4.018092
H	-2.481908	0.459294	3.285824
H	-3.433716	-0.867362	2.602437

[(TMS$_3$Si)(PFS$_2$N)P•]$_2$

136

E= -5464.142594

C	0.025665	-3.341337	-3.528713
Si	-1.459554	-2.390500	-2.858700
C	-2.878478	-3.612775	-3.090766
C	-1.813011	-0.949674	-3.994902
N	-1.283118	-1.868276	-1.154750
P	-1.121618	-0.150883	-0.805163
P	0.839905	0.770638	0.089817
Si	2.619607	-0.644230	0.721015
Si	2.554304	-0.707587	3.109258
C	1.999267	0.979056	3.743263
Si	-1.706718	-3.077144	0.082961
C	-0.771482	-2.652950	1.636801
C	-3.559841	-3.161437	0.354055
C	-1.289254	-4.863716	-0.390568
Si	-2.820866	0.507563	0.663031
Si	-4.755686	0.545097	-0.719270
C	-4.543367	1.901393	-2.016635
Si	-3.206131	-0.168787	2.927035
C	-3.888143	-1.884002	3.307988
Si	-2.546034	2.838250	1.027449
C	-1.512961	3.173629	2.567034
C	-1.610091	0.031706	3.926391
C	-4.493929	1.009664	3.653704
N	1.423512	1.851307	-1.190537
Si	1.903790	3.525457	-0.777962
C	1.055633	4.827284	-1.856206
Si	1.327696	1.479867	-2.924503
C	1.686512	-0.340964	-3.133450
C	-0.294737	2.093995	-3.645409
C	2.634971	2.338829	-3.981143
Si	4.689633	0.483220	0.403015
C	4.935760	0.923451	-1.408171
Si	3.207636	-2.789449	-0.117649
C	1.756190	-3.911462	-0.480719
C	4.864946	1.982964	1.539600

C	6.161865	-0.625358	0.830945	H	-2.210195	-5.418996	-0.585700
C	4.228678	-2.745948	-1.704153	H	-0.629341	-4.989581	-1.246725
C	4.237189	-3.648153	1.213177	H	-0.954265	-3.393524	2.420944
C	1.509500	-2.059779	3.906039	H	0.299853	-2.641511	1.431776
C	4.276560	-1.018116	3.827649	H	-1.030168	-1.672247	2.030799
C	1.546348	3.921824	1.015441	H	-3.776194	-3.893259	1.138467
C	3.713560	3.940447	-1.099815	H	-4.019407	-2.216702	0.636537
C	-1.765900	3.631976	-0.478806	H	-4.035733	-3.515624	-0.562578
C	-4.192363	3.730726	1.296442	H	-0.061821	-3.361719	-4.621063
C	-5.148134	-0.993585	-1.724905	H	0.997075	-2.913924	-3.286048
C	-6.273408	0.894013	0.345589	H	0.021437	-4.380035	-3.189239
H	1.461170	-0.690119	-4.144472	H	-2.149848	-1.355106	-4.954891
H	2.756829	-0.482441	-2.959585	H	-2.593582	-0.287936	-3.612956
H	1.151200	-0.967805	-2.421093	H	-0.927825	-0.345101	-4.179946
H	4.541743	-3.771733	-1.928342	H	-2.894079	-3.850200	-4.160737
H	3.638461	-2.391773	-2.552224	H	-2.733306	-4.555330	-2.561808
H	5.129609	-2.135027	-1.635530	H	-3.862422	-3.220954	-2.832814
H	2.439516	2.009075	-5.008637	H	5.801885	1.579388	-1.540884
H	2.566884	3.427539	-3.983646	H	5.104352	0.017900	-1.996117
H	3.661021	2.054937	-3.742285	H	4.061694	1.427893	-1.808270
H	-0.403278	1.786015	-4.690152	H	7.067170	-0.019818	0.706550
H	-1.156538	1.738272	-3.081451	H	6.152155	-0.994645	1.856702
H	-0.299024	3.186915	-3.629994	H	6.252295	-1.482243	0.160434
H	2.070669	-4.738969	-1.124678	H	5.735675	2.578799	1.245835
H	1.342394	-4.338621	0.434020	H	3.994007	2.638635	1.546072
H	0.965916	-3.364914	-0.993017	H	5.032621	1.649927	2.566885
H	4.508444	-4.647863	0.856493	H	1.745611	-2.079763	4.975530
H	5.159754	-3.117667	1.453119	H	0.439790	-1.894255	3.802059
H	3.665772	-3.771511	2.137619	H	1.739111	-3.047681	3.496675
H	-0.796376	-5.343189	0.460377	H	1.792363	0.932229	4.817306

H	2.782851	1.724832	3.583158
H	1.099295	1.335137	3.239342
H	4.175640	-1.045609	4.918321
H	4.691667	-1.978175	3.512726
H	4.998848	-0.236998	3.587953
H	-4.053893	-1.924387	4.390516
H	-4.844364	-2.080377	2.817744
H	-3.197704	-2.686020	3.047283
H	-1.678637	0.886812	4.603306
H	-1.428336	-0.859176	4.534148
H	-0.734701	0.190779	3.295179
H	-4.737379	0.659547	4.662792
H	-4.127429	2.033331	3.743013
H	-5.421262	1.027277	3.076656
H	-1.288250	4.242751	2.641458
H	-2.051548	2.890219	3.473819
H	-0.568551	2.627561	2.547229
H	-1.458666	4.657656	-0.253420
H	-0.892992	3.071254	-0.809831
H	-2.466060	3.664516	-1.316104
H	-3.965866	4.781643	1.509564
H	-4.830611	3.709283	0.410844
H	-4.769323	3.342613	2.136988
H	-5.956008	-0.745369	-2.421617
H	-4.286135	-1.302579	-2.315408
H	-5.473146	-1.838731	-1.118085
H	-5.352972	1.827521	-2.750108
H	-4.566627	2.908704	-1.598062
H	-3.596028	1.783722	-2.551794
H	-7.156766	0.983399	-0.295536
H	-6.453989	0.069566	1.042442
H	-6.184640	1.814279	0.925502
H	3.809882	5.012253	-0.889499
H	4.431917	3.420568	-0.472504
H	4.001227	3.791049	-2.141608
H	2.112061	4.819086	1.287346
H	0.488876	4.135020	1.165026
H	1.818652	3.122386	1.706335
H	1.204513	5.791318	-1.355840
H	1.527564	4.908511	-2.838204
H	-0.013959	4.696688	-2.001234

(PFS$_3$Si)(Ph$_2$N)P•

64

E= -5056.124737

C	4.010333	1.524109	0.055082
C	3.048431	1.265105	-0.920776
C	2.397326	2.323751	-1.550075
C	2.687391	3.632309	-1.182902
C	3.628050	3.889611	-0.196950
C	4.290894	2.831230	0.414843
N	2.777948	-0.078897	-1.302962
P	1.271657	-0.704461	-1.800904
Si	-0.253429	-0.095702	-0.204898
Si	-1.139217	2.113632	-0.252811
C	-1.023102	2.953833	-2.022741
F	-2.226397	3.348961	-2.449559
C	3.895033	-0.928488	-1.581532
C	3.867751	-2.262650	-1.181118
C	4.935620	-3.096410	-1.486013

C	6.035257	-2.603977	-2.174828
C	6.063111	-1.268473	-2.565757
C	4.997735	-0.431093	-2.278465
Si	-2.003890	-1.549402	-0.912439
C	-1.558762	-3.407102	-1.317077
F	-2.407733	-3.861879	-2.255691
Si	0.438152	-0.745137	1.973733
C	2.288970	-0.465447	2.491520
F	2.485292	-0.978621	3.717503
C	-3.575304	-1.615516	0.230229
F	-4.248492	-2.762764	0.073811
C	-2.627188	-0.868083	-2.620823
F	-1.742369	-1.144587	-3.593775
F	-3.259738	-1.505930	1.529828
F	-4.417161	-0.613143	-0.062140
C	-0.569357	-0.055994	3.486965
F	-0.713283	-0.987944	4.439877
C	0.284454	-2.678720	2.041331
F	0.912722	-3.238308	3.075660
F	0.061430	0.991385	4.040300
F	-1.794538	0.353194	3.135022
C	-2.994002	2.405990	0.270365
F	-3.171995	3.721909	0.498675
C	-0.158483	3.164303	1.067151
F	0.085534	4.426084	0.707579
F	-3.878791	2.043315	-0.660921
F	-3.307714	1.754630	1.397087
F	-1.005467	-3.048669	2.102613
F	0.803950	-3.225238	0.918197
F	3.126767	-1.112202	1.655175
F	2.666627	0.812128	2.538604
F	-0.847636	3.199723	2.220258
F	1.026861	2.586012	1.349718
F	-0.234903	4.041025	-2.024378
F	-0.530227	2.113009	-2.952957
F	-3.807632	-1.372482	-2.987779
F	-2.763765	0.474340	-2.583383
F	-0.318857	-3.553492	-1.802154
F	-1.677949	-4.211745	-0.255905
H	5.011593	0.605681	-2.592757
H	6.915981	-0.878647	-3.108981
H	6.870168	-3.255106	-2.404879
H	4.908520	-4.132182	-1.169186
H	3.022940	-2.636624	-0.616500
H	4.522211	0.698952	0.531902
H	5.026474	3.023031	1.186952
H	3.848753	4.910405	0.090391
H	2.179854	4.448324	-1.679839
H	1.714583	2.119761	-2.364987

[(PFS$_3$Si)(Ph$_2$N)P•]$_2$

128

E= -10112.2784652

P	0.782653	-0.372639	0.731277
Si	2.976788	-0.069749	-0.229957
Si	4.703767	-0.560999	1.394853
C	4.858939	-2.318749	2.219803
P	-1.140621	0.990436	0.758908
Si	-2.970085	-0.211490	-0.053124
Si	-3.303076	-1.166077	-2.239924

C	-1.578177	-1.756860	-2.946495	F	2.510194	4.248382	-1.947914
N	1.048454	-0.090538	2.402856	F	1.787735	2.535109	-3.040209
C	1.150279	-1.222016	3.288382	F	1.353328	2.743623	-0.921572
N	-1.296778	2.461135	-0.075734	F	4.579331	2.811232	-3.791662
C	-1.454900	2.609936	-1.488756	F	6.109696	1.558553	-2.939247
C	-1.128221	3.651559	0.731585	F	4.319909	0.666302	-3.767249
Si	-3.282439	-2.065562	1.450752	F	6.022217	0.532177	3.518906
C	-2.806225	-1.810127	3.325466	F	4.704096	1.942458	2.555334
Si	-4.797002	1.295202	0.433250	F	3.898956	0.397999	3.833768
C	-5.009949	3.145519	-0.161444	F	7.061278	-1.541032	0.342282
C	-5.119224	-2.681159	1.585622	F	6.230789	0.112369	-0.774010
C	-2.319357	-3.638034	0.796245	F	7.266334	0.461743	1.091946
C	1.006788	1.230866	2.954539	F	5.947366	-2.297562	3.014345
Si	3.843277	1.882286	-1.365183	F	3.818520	-2.647912	2.981058
C	4.872767	3.183899	-0.351164	F	5.041757	-3.296120	1.327051
Si	3.092701	-1.877581	-1.798563	F	1.291206	-2.505055	-3.772697
C	2.178881	-1.542855	-3.491652	F	1.515446	-0.365212	-3.461637
C	4.790643	1.705897	-3.057536	F	3.026345	-1.488053	-4.522490
C	2.300387	2.928978	-1.869963	F	2.721468	-4.583318	-1.501095
C	4.839647	-2.528953	-2.355015	F	2.531262	-3.471335	0.344143
C	2.262164	-3.440473	-0.977062	F	0.932378	-3.447906	-1.102252
C	6.428304	-0.375226	0.480888	F	4.693200	-3.468831	-3.300626
C	4.835922	0.656956	2.912671	F	5.632454	-1.577264	-2.856809
C	-6.462135	0.529311	-0.237203	F	5.486920	-3.094962	-1.325074
C	-4.894288	1.524866	2.380923	F	-3.592389	0.219134	-4.632304
C	-4.394971	-2.758806	-2.559220	F	-5.445929	-0.191347	-3.616095
C	-4.157079	0.128679	-3.426771	F	-4.144794	1.355384	-2.872444
F	5.323095	4.140788	-1.174824	F	-1.519504	-3.079322	-3.096265
F	4.089530	3.784927	0.566769	F	-1.270195	-1.211200	-4.127089
F	5.933405	2.681865	0.285370	F	-0.594453	-1.416584	-2.075891

F	-4.258521	-3.029054	-3.876619	H	2.299641	2.090902	1.469268
F	-3.975668	-3.834967	-1.885608	C	1.598887	-1.070873	4.602155
F	-5.696066	-2.619625	-2.321924	C	1.680277	-2.166929	5.447206
F	-1.352603	-4.061473	1.615756	C	1.330890	-3.436096	5.008028
F	-3.138655	-4.673549	0.596304	C	0.903410	-3.591269	3.699091
F	-1.730069	-3.350670	-0.383888	C	0.812402	-2.499521	2.849914
F	-2.419152	-2.958861	3.889171	H	1.893439	-0.102082	4.973931
F	-1.815632	-0.920428	3.489311	H	2.031751	-2.018330	6.461827
F	-3.862576	-1.366810	4.023456	H	1.397670	-4.288765	5.672576
F	-5.217592	-3.633751	2.524838	H	0.628310	-4.568875	3.323068
F	-5.942883	-1.680559	1.945407	H	0.492569	-2.658542	1.831994
F	-5.581380	-3.193259	0.441564	C	-0.390110	4.737055	0.262371
F	-6.219740	3.525662	0.304477	C	-0.306073	5.898220	1.015758
F	-4.109606	3.987103	0.360500	C	-0.945800	5.992082	2.244920
F	-5.024442	3.334143	-1.477844	C	-1.658831	4.902251	2.721586
F	-5.051826	2.805933	2.737721	C	-1.747063	3.738607	1.973926
F	-5.867913	0.826816	2.960540	H	0.095734	4.690033	-0.700194
F	-3.725696	1.118071	2.937805	H	0.263451	6.736027	0.630490
F	-7.450080	0.522664	0.656898	H	-0.887632	6.905728	2.824632
F	-6.895121	1.198467	-1.314423	H	-2.161549	4.951179	3.680131
F	-6.260396	-0.745293	-0.634138	H	-2.306034	2.900003	2.363972
C	0.230145	1.506659	4.083804	C	-2.104014	3.730173	-2.009886
C	0.259622	2.766176	4.655279	C	-2.237831	3.881293	-3.379881
C	1.027794	3.782004	4.094361	C	-1.726121	2.930982	-4.253794
C	1.740936	3.532054	2.935184	C	-1.082535	1.818594	-3.738762
C	1.727024	2.263732	2.369522	C	-0.955062	1.651900	-2.365413
H	-0.379088	0.725947	4.517593	H	-2.500935	4.483988	-1.346335
H	-0.336104	2.959664	5.540065	H	-2.754676	4.752936	-3.763601
H	1.042782	4.768044	4.541926	H	-1.827674	3.056654	-5.324427
H	2.319972	4.315154	2.463224	H	-0.650582	1.080106	-4.399267

H	-0.385196	0.816566	-1.985608

(PFS3Si)(PFP2N)P•

64

E= -6048.409016

C	-3.296122	-1.438815	1.017112
C	-2.661881	-1.160491	-0.191709
C	-2.452809	-2.218491	-1.074864
C	-2.776867	-3.518653	-0.734135
C	-3.370170	-3.776422	0.491829
C	-3.638081	-2.733938	1.365005
N	-2.367112	0.172767	-0.562362
P	-0.959147	0.752511	-1.351964
Si	0.942405	0.166898	-0.168663
Si	2.291643	-0.885127	-1.806377
C	1.283953	-2.260907	-2.736626
F	0.404060	-1.702979	-3.582295
F	-1.961947	-1.977212	-2.282518
F	-2.547276	-4.508932	-1.582448
F	-3.681616	-5.016594	0.828526
F	-4.210945	-2.978948	2.533862
F	-3.598847	-0.452112	1.850659
C	-3.473554	1.063437	-0.639696
C	-3.517130	2.209779	0.149260
C	-4.590755	3.082047	0.079338
C	-5.647807	2.805394	-0.774575
C	-5.625985	1.665520	-1.565565
C	-4.539221	0.811246	-1.501401
F	-2.527000	2.478765	0.983387
F	-4.514946	-0.263364	-2.281292
F	-6.633009	1.410285	-2.388248
F	-6.678403	3.631125	-0.838168
F	-4.619489	4.169089	0.835567
Si	1.033280	-1.052543	1.859655
C	-0.455670	-0.556269	3.018793
F	-1.123035	0.479237	2.479019
Si	1.874785	2.290748	0.337605
C	1.219713	3.700874	-0.827680
F	1.463968	3.431902	-2.118249
C	0.865976	-2.954109	1.535353
F	0.949793	-3.662354	2.666351
C	2.725205	-0.870873	2.829135
F	2.546062	-0.612921	4.125586
F	-0.311421	-3.254744	0.957419
F	1.832918	-3.385533	0.708100
C	1.315685	2.859228	2.109544
F	1.453458	1.863349	3.016023
C	3.824205	2.382393	0.259501
F	4.351459	1.206102	-0.132118
F	2.018201	3.901804	2.557043
F	0.024191	3.208105	2.110113
C	2.730776	0.381839	-3.208287
F	1.608684	0.948236	-3.687801
C	3.943942	-1.711200	-1.184231
F	3.952291	-3.030341	-1.406278
F	3.518900	1.375899	-2.771690
F	3.353455	-0.211215	-4.233378
F	4.236271	3.308586	-0.613485
F	4.359314	2.679458	1.447829
F	-0.104228	3.880902	-0.697071

F	1.807467	4.869133	-0.533318
F	5.011699	-1.189340	-1.799346
F	4.111720	-1.533744	0.137392
F	2.069677	-3.066156	-3.458137
F	0.591473	-3.032260	-1.877906
F	3.440057	-1.999084	2.744415
F	3.480921	0.121630	2.322821
F	-1.323332	-1.581313	3.126423
F	-0.107549	-0.209594	4.254133

[(PFS$_3$Si)(PFP$_2$N)P•]$_2$

128

E= -12096.8384585

C	0.052981	3.592360	-0.147340
C	-0.927194	3.114948	0.712154
C	-1.880534	4.023184	1.152325
C	-1.891689	5.331323	0.699149
C	-0.940021	5.755268	-0.212268
C	0.046161	4.879309	-0.639838
N	-0.915058	1.753627	1.132382
C	-0.680117	1.466755	2.500786
C	-1.004352	0.235331	3.066876
C	-0.718717	-0.099903	4.374814
C	-0.104298	0.817708	5.205848
C	0.207011	2.063078	4.695237
C	-0.066800	2.372971	3.372264
F	-1.625809	-0.703868	2.328072
F	-1.064818	-1.291768	4.841361
F	0.167358	0.510942	6.463499
F	0.794295	2.964932	5.463234
F	0.308986	3.581491	2.962172
F	-2.778400	3.654328	2.053764
F	-2.820610	6.171045	1.126930
F	-0.959878	6.997092	-0.663721
F	0.979902	5.292084	-1.476953
F	1.071370	2.776161	-0.475402
P	-0.873549	0.640832	-0.236256
Si	-3.165385	-0.149121	-0.309608
Si	-3.604060	-2.111614	-1.645176
C	-2.467090	-3.493070	-0.897620
F	-3.022958	-4.340911	-0.043919
P	1.007036	-0.427695	0.683581
Si	2.918780	0.351577	-0.494654
Si	3.897612	2.127346	0.905021
C	5.827519	2.283141	0.552187
F	6.121182	3.400701	-0.113914
N	1.078786	-2.157487	0.668710
C	1.187145	-2.604830	2.025675
C	2.326620	-2.262981	2.741855
C	2.495084	-2.572350	4.075927
C	1.502343	-3.293564	4.721642
C	0.369154	-3.682979	4.025652
C	0.209302	-3.331942	2.695563
F	3.282988	-1.572480	2.119771
F	3.584771	-2.194440	4.723735
F	1.634940	-3.606592	5.997996
F	-0.575382	-4.374736	4.641763
F	-0.874380	-3.747003	2.065823
C	1.127186	-3.091567	-0.396314
C	0.972988	-2.695889	-1.723445

C	0.969143	-3.576571	-2.788242	F	-3.882566	3.669594	-3.278147
C	1.128797	-4.928948	-2.560929	F	-4.885271	2.439865	0.962824
C	1.290321	-5.364472	-1.260767	F	-4.452747	4.088472	-0.368149
C	1.294998	-4.467958	-0.205760	C	-4.329693	0.401410	3.355597
F	0.788160	-1.394167	-2.008411	F	-3.644424	-0.407856	4.184776
F	1.504376	-4.995051	1.000471	C	-6.427712	-0.105640	1.084012
F	1.465450	-6.652957	-1.011519	F	-6.456292	0.199244	-0.246354
F	1.128143	-5.786922	-3.567522	F	-3.674037	1.563965	3.306512
F	0.796266	-3.130540	-4.018933	F	-5.514117	0.626313	3.932651
Si	4.784758	-1.261613	-0.505499	C	-5.352890	-2.944246	-1.944029
C	5.891394	-1.217559	1.128129	F	-6.349969	-2.422750	-1.227228
F	5.785277	-2.350308	1.831677	C	-2.816111	-1.810924	-3.391779
Si	2.618600	1.068922	-2.783530	F	-1.493764	-1.608435	-3.226969
C	2.969308	-0.432851	-3.997070	F	-5.695178	-2.873855	-3.235856
F	3.258030	-1.547523	-3.271330	F	-5.267497	-4.246905	-1.634103
C	4.537843	-3.193368	-0.632815	F	-7.213686	-1.169360	1.249126
F	5.770300	-3.732358	-0.653275	F	-6.996635	0.917043	1.715571
C	5.959281	-0.929049	-2.048747	F	-3.426619	-2.823463	2.460503
F	7.239295	-0.766002	-1.730191	F	-5.452151	-2.548179	3.157574
F	3.900133	-3.719202	0.426725	C	0.904001	1.679370	-3.497589
F	3.906108	-3.621121	-1.729990	F	1.128854	1.942953	-4.801160
Si	-4.240454	1.622010	-1.558009	C	3.788171	2.570730	-3.213155
C	-5.587881	1.040456	-2.860095	F	3.084984	3.710696	-3.250555
F	-5.210980	1.344661	-4.105816	F	-0.048837	0.745492	-3.436466
Si	-4.575264	-0.469130	1.622754	F	0.408997	2.797572	-2.962207
C	-4.627083	-2.342740	2.125326	C	3.441461	4.023524	0.731294
F	-5.081344	-3.086620	1.094227	F	2.245625	4.366395	1.231821
C	-5.054635	2.891208	-0.308444	C	3.597712	1.866248	2.842317
F	-6.355021	3.093008	-0.478313	F	4.708886	1.645102	3.537716
C	-3.116311	2.745948	-2.673823	F	4.355814	4.708467	1.443219

F	3.489005	4.466635	-0.525845
F	4.741693	2.727477	-2.273455
F	4.404424	2.440581	-4.387863
F	1.897240	-0.727184	-4.733633
F	3.983817	-0.238841	-4.834407
F	3.021293	2.959036	3.369714
F	2.754044	0.842980	3.093724
F	6.574081	2.271908	1.653545
F	6.251870	1.257114	-0.232393
F	5.899473	-1.931135	-2.932514
F	5.569213	0.194286	-2.700743
F	7.186542	-1.036416	0.885254
F	5.497641	-0.215004	1.949438
F	-2.965662	-2.845273	-4.219437
F	-3.301260	-0.720937	-4.003177
F	-1.851612	-4.225649	-1.840279
F	-1.501257	-2.822162	-0.219601
F	-2.188222	3.413646	-1.969398
F	-2.481064	2.055967	-3.622360
F	-5.756132	-0.299132	-2.821829
F	-6.783286	1.591796	-2.659972

(TMS$_3$Si)(Pyr^{Me})P•

66

E= -2227.932291

C	-4.685585	-0.078548	0.198003
C	-3.538145	-0.991944	-0.287998
N	-2.373531	-0.058935	-0.421889
C	-2.800664	1.345845	-0.223405
C	-3.994499	1.173730	0.720462
C	-3.211930	-2.053057	0.768424
C	-3.907872	-1.676172	-1.608211
P	-0.970485	-0.651461	-1.181827
Si	0.939835	-0.009008	-0.105496
Si	0.866430	-0.776741	2.117064
C	0.666916	-2.650381	2.130133
C	-1.721366	2.219400	0.390189
C	-3.231069	1.966566	-1.564262
Si	1.926033	2.135233	-0.177262
C	1.421441	3.255796	1.257526
Si	2.420267	-1.450055	-1.260814
C	2.917221	-0.664209	-2.903189
C	3.795261	1.916355	-0.018530
C	1.561094	2.986093	-1.818769
C	3.968475	-1.788746	-0.234874
C	1.610214	-3.111536	-1.625609
C	2.482635	-0.326643	2.983270
C	-0.557806	-0.019891	3.093749
H	-2.153879	3.181725	0.679174
H	-1.285624	1.756668	1.274935
H	-0.927707	2.417971	-0.329941
H	2.082977	4.128288	1.283264
H	0.395469	3.618158	1.178598
H	1.521478	2.739450	2.216827
H	-3.555586	3.001590	-1.419981
H	-2.387637	1.961873	-2.260029
H	-4.050107	1.412928	-2.027413
H	2.073959	3.951782	-1.874640
H	1.905584	2.371107	-2.655142
H	0.491811	3.165424	-1.961145

H	4.280536	2.898090	-0.009301
H	4.056775	1.407413	0.913871
H	4.219914	1.342713	-0.845737
H	-0.640635	-0.499945	4.074297
H	-0.409577	1.051421	3.258260
H	-1.505944	-0.154492	2.566715
H	2.485965	-0.710688	4.008784
H	3.347509	-0.748239	2.464240
H	2.620374	0.757397	3.034445
H	0.570909	-3.021018	3.155896
H	-0.224177	-2.954992	1.574613
H	1.529476	-3.144951	1.674670
H	3.571024	-1.335233	-3.469916
H	2.033085	-0.462479	-3.515534
H	3.448777	0.281172	-2.763686
H	2.333035	-3.781574	-2.103561
H	1.254481	-3.592960	-0.710731
H	0.751878	-3.000095	-2.292442
H	4.671669	-2.396485	-0.814155
H	4.483682	-0.873309	0.064465
H	3.720455	-2.347983	0.672163
H	-4.049285	-2.747862	0.881300
H	-2.328012	-2.623741	0.472436
H	-3.013180	-1.589582	1.738190
H	-4.826637	-2.255437	-1.476687
H	-4.075006	-0.936431	-2.394951
H	-3.119103	-2.355042	-1.939271
H	-5.292609	-0.577815	0.955509
H	-3.624687	1.017641	1.739193
H	-4.642798	2.052942	0.727335
H	-5.348467	0.171105	-0.634615

[(TMS$_3$Si)(Pyr^{Me})P•]$_2$

132

E= -4455.921865

C	-1.924813	-2.849317	1.329658
N	-1.143321	-1.642294	1.683221
C	-0.670368	-1.710052	3.095848
C	-1.029247	-3.150929	3.533172
C	-1.293344	-3.911649	2.238123
P	-0.839302	-0.241503	0.758531
Si	-2.808451	0.546186	-0.198421
Si	-2.270712	2.589939	-1.287266
C	-1.711860	3.828101	0.021922
C	-1.365822	-0.678710	3.985864
C	0.839203	-1.493336	3.182690
C	-1.823727	-3.189340	-0.146382
C	-3.406892	-2.683780	1.694349
P	0.838954	-0.240103	-0.758223
Si	2.808707	0.545939	0.199176
Si	4.068844	1.406738	-1.654653
C	5.168092	0.138223	-2.521092
N	1.142786	-1.639351	-1.685107
C	0.669288	-1.705529	-3.097555
C	1.027349	-3.146164	-3.536342
C	1.291369	-3.908339	-2.242109
C	1.923386	-2.847189	-1.332622
C	1.365019	-0.673611	-3.986675
C	-0.840210	-1.487932	-3.183792
C	3.405564	-2.682280	-1.697171

C	1.821974	-3.188288	0.143230
Si	4.381090	-0.474294	1.705196
C	3.915844	-0.236294	3.522813
Si	2.270552	2.587119	1.292629
C	1.710508	3.827432	-0.013996
C	4.859220	-2.293277	1.536768
C	6.006008	0.456599	1.449523
Si	-4.378119	-0.471471	-1.708968
C	-4.849996	-2.292909	-1.549874
Si	-4.069756	1.403370	1.656444
C	-2.959561	2.227737	2.948866
C	-6.005139	0.454473	-1.448698
C	-3.913939	-0.223644	-3.525727
C	-5.258862	2.771640	1.110171
C	-5.168705	0.133905	2.521912
C	-0.962702	2.426115	-2.628624
C	-3.789828	3.316075	-2.147279
C	0.963266	2.420081	2.634257
C	3.789443	3.312403	2.153680
C	2.958499	2.232209	-2.946192
C	5.257193	2.774951	-1.106575
H	3.973300	-3.571825	-1.407472
H	3.544752	-2.526592	-2.768045
H	3.829151	-1.823739	-1.177145
H	4.836135	-0.145581	4.109618
H	3.311211	0.652473	3.711245
H	3.365796	-1.098679	3.902702
H	2.318896	-2.426060	0.738338
H	0.790979	-3.234047	0.478674
H	2.308404	-4.147376	0.341805
H	6.741228	0.049505	2.152314
H	6.406212	0.326763	0.440838
H	5.914020	1.525392	1.648849
H	5.168952	-2.563653	0.525737
H	5.715469	-2.454845	2.201455
H	4.069747	-2.979270	1.846710
H	1.122077	-0.538123	2.733632
H	1.150310	-1.476053	4.230970
H	1.382833	-2.291016	2.673259
H	-2.451330	-0.749153	3.886647
H	-1.103228	-0.843301	5.035667
H	-1.059873	0.333066	3.715109
H	-2.314628	-4.145822	-0.346402
H	-2.316498	-2.424088	-0.741013
H	-0.792689	-3.240009	-0.480926
H	-3.830396	-1.825303	1.174089
H	-3.974913	-3.573326	1.405187
H	-3.545848	-2.527666	2.765168
H	5.801520	3.097366	-2.001630
H	4.732513	3.649480	-0.716969
H	5.994229	2.459058	-0.368540
H	3.026598	1.720298	-3.908924
H	1.909098	2.253062	-2.653766
H	3.280592	3.267329	-3.094284
H	4.594964	-0.565396	-3.125998
H	5.852186	0.672432	-3.189087
H	5.773808	-0.441551	-1.818679
H	0.949176	3.408991	-0.674466
H	1.290878	4.720359	0.459856
H	2.549691	4.141621	-0.639488

H	4.624224	3.497936	1.476559
H	3.502018	4.272314	2.597065
H	4.145819	2.672624	2.964915
H	1.350709	1.871870	3.498302
H	0.650148	3.411382	2.978791
H	0.092507	1.877811	2.264150
H	-4.624847	3.499815	-1.469976
H	-3.502829	4.277052	-2.588636
H	-4.145789	2.677831	-2.959875
H	-0.950894	3.408760	0.682255
H	-1.292113	4.721924	-0.450135
H	-2.551591	4.141034	0.647311
H	-0.092492	1.882274	-2.259523
H	-1.349804	1.880644	-3.494542
H	-0.648779	3.418229	-2.970067
H	-3.306693	0.664228	-3.709675
H	-3.366928	-1.085524	-3.911172
H	-4.834456	-0.126620	-4.111156
H	-5.153163	-2.571815	-0.539203
H	-5.709103	-2.452965	-2.211221
H	-4.059504	-2.973523	-1.869096
H	-6.740292	0.047914	-2.151862
H	-6.404011	0.321264	-0.439928
H	-5.915346	1.524017	-1.645268
H	-3.028667	1.715587	3.911409
H	-1.909953	2.247879	2.657139
H	-3.280931	3.263068	3.097030
H	-5.853424	0.667807	3.189518
H	-5.773759	-0.446200	1.819227
H	-4.595600	-0.569364	3.127225
H	-5.803549	3.092320	2.005628
H	-4.734764	3.647130	0.721968
H	-5.995583	2.456262	0.371585
H	1.101944	-0.836779	-5.036580
H	1.059756	0.338020	-3.714559
H	2.450524	-0.744848	-3.887897
H	-1.384116	-2.285578	-2.674608
H	-1.122343	-0.532749	-2.734190
H	-1.151623	-1.469937	-4.231962
H	-1.951254	-4.773071	2.377574
H	-0.226455	-3.592055	4.127242
H	1.922153	-3.137717	-4.164147
H	1.949034	-4.769787	-2.382577
H	-0.358981	-4.272598	1.801570
H	-1.924195	-3.142675	4.160776
H	0.356968	-4.269584	-1.805937
H	0.224209	-3.586317	-4.130665

(TMS$_3$Si)(Pip^{Me})P•

69

E= -2267.234328

C	-2.572723	1.313806	-0.223038
N	-2.339389	-0.093249	-0.666506
C	-3.355329	-1.164210	-0.380071
C	-4.792722	-0.623833	-0.479019
C	-5.009382	0.689516	0.246974
C	-4.056516	1.703902	-0.353151
P	-0.829425	-0.679071	-1.235176
Si	1.036309	0.002459	-0.085362
Si	2.594099	-1.241853	-1.372624

C	1.867396	-2.889655	-1.927020
C	-3.263596	-2.303993	-1.409352
C	-3.116003	-1.764990	1.016410
C	-2.106520	1.541655	1.221195
C	-1.805367	2.262618	-1.148572
Si	1.136102	-0.982259	2.064497
C	-0.219561	-0.465857	3.268164
Si	1.903462	2.201553	-0.022957
C	1.785111	3.104307	-1.675745
C	1.038624	-2.854669	1.857772
C	2.768550	-0.534965	2.900747
C	1.088272	3.257811	1.309997
C	3.744283	2.069776	0.378261
C	3.086064	-0.266173	-2.910749
C	4.161657	-1.614599	-0.387969
H	-2.045420	3.298774	-0.892900
H	-0.730659	2.134052	-1.054195
H	-2.078799	2.086208	-2.191432
H	1.629522	4.203262	1.420198
H	0.051198	3.492009	1.059219
H	1.092110	2.754247	2.281453
H	-2.197415	2.599277	1.485237
H	-2.690755	0.967061	1.940272
H	-1.060967	1.257675	1.336443
H	-4.180624	2.689034	0.106743
H	-4.294934	1.816198	-1.417309
H	2.390898	4.015773	-1.628131
H	2.169989	2.490510	-2.493708
H	0.765189	3.398459	-1.930806
H	4.161425	3.074290	0.506979
H	3.937266	1.506277	1.293695
H	4.292114	1.587574	-0.436115
H	-0.046117	-0.971628	4.224300
H	-0.216992	0.611219	3.455827
H	-1.213854	-0.744974	2.916293
H	2.864860	-1.091887	3.838974
H	3.637480	-0.766652	2.281399
H	2.801496	0.530939	3.145904
H	0.994932	-3.346285	2.835085
H	0.148439	-3.145003	1.291914
H	1.909897	-3.245280	1.325028
H	3.722518	-0.877144	-3.559316
H	2.205889	0.031647	-3.488031
H	3.643956	0.639072	-2.654910
H	2.631464	-3.470946	-2.454504
H	1.516585	-3.481737	-1.077713
H	1.019487	-2.746671	-2.601123
H	4.890343	-2.110402	-1.038175
H	4.629324	-0.710791	0.010126
H	3.958536	-2.286137	0.450836
H	-3.777046	-2.621355	1.178860
H	-2.082495	-2.110372	1.100264
H	-3.300680	-1.044727	1.815008
H	-4.129888	-2.958084	-1.274406
H	-3.283947	-1.909455	-2.427873
H	-2.363416	-2.907052	-1.294140
H	-6.043915	1.022326	0.125278
H	-4.842846	0.578760	1.323572
H	-5.035672	-0.480045	-1.538429
H	-5.464406	-1.400487	-0.101267

[(TMS$_3$Si)(Pip^{Me})P•]$_2$

138

E= -4534.508517

C	1.248789	-1.841400	-3.064094
N	1.625619	-1.663639	-1.604538
C	1.950126	-2.943926	-0.877934
C	0.845543	-3.990445	-1.119499
C	0.489728	-4.212941	-2.580855
C	0.113260	-2.873396	-3.193307
P	0.925500	-0.282628	-0.792630
Si	2.659993	0.786342	0.365119
Si	4.006744	-0.048883	2.141765
C	4.925915	1.425415	2.884873
C	3.322907	-3.547374	-1.260995
C	2.056603	-2.686879	0.624408
C	0.773954	-0.537280	-3.697993
C	2.435983	-2.272143	-3.953531
P	-1.104584	-0.953064	0.051167
Si	-2.594510	0.803210	-0.426959
Si	-2.355066	1.486463	-2.703476
C	-3.712383	2.706038	-3.222180
N	-1.333278	-1.569164	1.660505
C	-1.748775	-3.007569	1.889349
C	-2.623988	-3.096334	3.153707
C	-1.890496	-2.652766	4.421919
C	-0.879172	-1.541554	4.117366
C	-1.249182	-0.707248	2.879912
C	-2.587376	-3.555455	0.734233
C	-0.561307	-3.968623	2.110275
C	-2.572713	0.023650	3.169699
C	-0.148327	0.328452	2.699428
Si	-4.650322	-0.393600	-0.589451
C	-5.996906	0.628487	-1.438928
Si	-3.080002	2.802940	0.836749
C	-3.155403	4.280735	-0.334273
C	-4.442539	-1.946965	-1.654145
C	-5.350163	-0.888445	1.091192
Si	2.002542	2.999755	0.955901
C	3.406707	4.240502	0.706877
Si	4.248003	1.236463	-1.345854
C	5.796129	2.098036	-0.683013
C	0.623076	3.539117	-0.203894
C	1.553147	3.281575	2.768252
C	4.906302	-0.327622	-2.146203
C	3.468328	2.343005	-2.661353
C	3.095699	-0.905092	3.554550
C	5.362538	-1.208569	1.512198
C	-4.781776	2.667297	1.649067
C	-1.930835	3.355700	2.223091
C	-0.742083	2.374385	-3.106749
C	-2.623578	-0.016801	-3.822110
H	2.224563	-3.635472	1.139361
H	2.911647	-2.048280	0.824736
H	1.170241	-2.224574	1.054210
H	5.936366	-1.580268	2.368086
H	4.993834	-2.074277	0.958634
H	6.057028	-0.669938	0.862015
H	3.658412	-4.197412	-0.448422
H	3.296078	-4.168432	-2.154076

H	4.072335	-2.769267	-1.397873	H	1.305297	4.341185	2.896734
H	1.138693	-4.930952	-0.639369	H	0.711053	2.700807	3.131702
H	-0.056878	-3.631177	-0.625261	H	2.412402	3.071325	3.409582
H	3.830492	-1.277499	4.276278	H	0.218924	4.516089	0.080056
H	2.433031	-0.212749	4.079692	H	1.026724	3.622830	-1.215853
H	2.497916	-1.754877	3.216398	H	-0.198091	2.820129	-0.246977
H	5.675953	1.057190	3.593202	H	3.021515	5.227353	0.987373
H	5.448572	2.008441	2.123285	H	4.260882	4.028333	1.354732
H	4.261340	2.098786	3.429534	H	3.760042	4.307175	-0.322948
H	-0.004977	0.867339	3.638647	H	-5.044346	3.649867	2.056332
H	0.791235	-0.154941	2.434050	H	-5.571985	2.375293	0.954728
H	-0.392562	1.055564	1.924941	H	-4.780127	1.956809	2.479455
H	-2.422445	0.780935	3.944092	H	-0.938850	3.617848	1.863004
H	-2.941097	0.518258	2.273901	H	-2.374552	4.262245	2.650413
H	-3.359339	-0.642025	3.518221	H	-1.823620	2.630595	3.027774
H	-0.047253	-4.193765	1.184056	H	-3.239877	5.191689	0.268136
H	0.172832	-3.576383	2.812216	H	-2.255451	4.369266	-0.948398
H	-0.937143	-4.915422	2.510222	H	-4.020287	4.239071	-0.997891
H	-2.843588	-4.597416	0.945478	H	-0.573889	2.359823	-4.188862
H	-3.514839	-2.995309	0.622640	H	-0.809718	3.419101	-2.791633
H	-2.059370	-3.524398	-0.217717	H	0.118871	1.925776	-2.612393
H	5.488376	-0.083259	-3.040997	H	-2.187311	0.172922	-4.808272
H	5.559378	-0.869703	-1.456809	H	-2.201942	-0.946057	-3.438789
H	4.090236	-0.987003	-2.425951	H	-3.697459	-0.171871	-3.959686
H	6.454846	2.300120	-1.535295	H	-3.930104	2.534434	-4.282137
H	5.610236	3.044380	-0.176665	H	-4.649513	2.602364	-2.674922
H	6.347546	1.447690	0.001380	H	-3.373181	3.738117	-3.119086
H	4.119448	2.406473	-3.539269	H	-6.224724	-1.531421	0.945145
H	2.496260	1.966498	-2.987190	H	-4.625187	-1.435995	1.692485
H	3.313686	3.359175	-2.289241	H	-5.666613	-0.014781	1.664825

H	-5.052485	-2.765513	-1.259472
H	-4.773639	-1.751081	-2.676734
H	-3.407472	-2.289432	-1.698773
H	-6.953458	0.107586	-1.320913
H	-6.115966	1.633681	-1.027652
H	-5.805604	0.723375	-2.510593
H	2.050425	-2.462752	-4.959111
H	3.169599	-1.470534	-4.036703
H	2.951913	-3.169585	-3.635640
H	0.586005	-0.718210	-4.759895
H	-0.140859	-0.172135	-3.251200
H	1.530680	0.243958	-3.614029
H	-0.349536	-4.911587	-2.649644
H	1.318000	-4.672368	-3.130821
H	-0.765649	-2.480568	-2.669165
H	-0.154831	-2.983848	-4.249048
H	-3.532815	-2.513328	3.002713
H	-2.952603	-4.134035	3.255518
H	-2.619960	-2.313937	5.162384
H	-1.370574	-3.502111	4.872656
H	0.123751	-1.950177	3.973437
H	-0.804775	-0.857834	4.967945

(TMS$_3$Si)(Pyr^{TFM})P•

66

E= -3418.854376

C	-3.811272	-0.447925	0.640653
C	-2.502510	-1.080161	0.128414
N	-1.607237	0.040252	-0.138597
C	-2.202288	1.306277	0.261518
C	-3.389175	0.917030	1.159707
C	-1.894881	-1.978149	1.232189
F	-1.816087	-1.302780	2.388794
C	-2.832510	-1.947599	-1.123789
F	-1.863945	-2.807952	-1.447338
P	-0.185943	-0.171889	-1.186818
Si	1.830970	-0.033709	-0.106242
Si	2.389795	-0.922159	2.012999
C	2.359008	-2.805190	2.018967
C	-1.232197	2.186192	1.083520
F	-0.345888	2.829005	0.312410
C	-2.714427	2.117559	-0.962897
F	-3.744060	1.489952	-1.543972
Si	2.840964	2.087782	-0.390155
C	2.719799	3.150133	1.161922
Si	2.874468	-1.469829	-1.683838
C	2.898910	-0.646962	-3.379787
C	4.678668	1.842666	-0.743242
C	2.077846	2.963354	-1.870379
F	-1.892992	3.118783	1.779415
F	-0.552255	1.447110	1.957714
F	-3.150100	3.329686	-0.592536
F	-1.779185	2.290784	-1.896762
F	-2.639637	-3.065245	1.460163
F	-0.660547	-2.388225	0.930809
F	-3.938994	-2.674040	-0.902821
F	-3.070315	-1.172285	-2.179379
C	4.645867	-1.867729	-1.172503
C	1.917795	-3.087533	-1.808461
C	4.185014	-0.399946	2.285446

C	1.381171	-0.280763	3.460590
H	3.249956	4.094961	1.001548
H	1.688847	3.384347	1.426642
H	3.183880	2.653143	2.018277
H	2.554139	3.937326	-2.023285
H	2.220574	2.373552	-2.780501
H	1.005855	3.124331	-1.744670
H	5.163255	2.820447	-0.836810
H	5.180709	1.298048	0.060496
H	4.845710	1.300101	-1.677278
H	1.792487	-0.691808	4.388706
H	1.422781	0.808953	3.525134
H	0.333640	-0.576612	3.392733
H	4.550526	-0.838888	3.220022
H	4.838179	-0.743194	1.479394
H	4.290744	0.684824	2.366345
H	2.725330	-3.165211	2.986404
H	1.355557	-3.205107	1.869050
H	3.009059	-3.220611	1.244598
H	3.333111	-1.322840	-4.123830
H	1.887645	-0.392324	-3.710321
H	3.492262	0.271665	-3.376475
H	2.438606	-3.782904	-2.475097
H	1.812763	-3.569653	-0.833035
H	0.913280	-2.924888	-2.206904
H	5.115628	-2.484918	-1.945695
H	5.253553	-0.968703	-1.047171
H	4.678982	-2.431630	-0.236456
H	-4.286345	-1.063692	1.402443
H	-3.044317	0.847582	2.189968
H	-4.188902	1.654767	1.118585
H	-4.507316	-0.344353	-0.188765

[(TMS$_3$Si)(Pyr^{TFM})P•]$_2$

132

E= -6837.673806

C	2.225420	1.446969	-2.344469
N	1.729843	1.632467	-0.968503
C	1.922865	3.053426	-0.604878
C	2.969950	3.621530	-1.598790
C	3.390130	2.441862	-2.460644
P	1.047001	0.519172	0.262185
Si	2.889045	-0.994176	0.991742
Si	2.541385	-1.258199	3.367646
C	2.477626	0.449668	4.175529
C	2.460345	3.271348	0.841579
F	1.548500	3.152780	1.804533
C	0.595141	3.846721	-0.718100
F	0.290231	4.185258	-1.970936
C	2.816921	0.048722	-2.586354
F	3.738964	-0.207936	-1.654655
C	1.151528	1.773641	-3.462309
F	-0.080346	1.779484	-2.981840
P	-1.261260	0.039758	-0.505727
Si	-2.901719	0.901234	1.069658
Si	-5.234582	0.473388	0.611404
C	-5.751237	1.394912	-0.952980
N	-1.746932	-1.652547	-0.876801
C	-2.093784	-2.043880	-2.263286
C	-2.409624	-3.555729	-2.212303

C	-1.659008	-4.040441	-0.984140
C	-1.784103	-2.854770	-0.021605
C	-0.942426	-1.884369	-3.279701
F	-0.517407	-0.643914	-3.404539
C	-3.340972	-1.285890	-2.786798
F	-4.305497	-1.313590	-1.862751
C	-3.089710	-3.049649	0.795988
F	-3.436182	-1.965029	1.473406
C	-0.612788	-2.848667	0.961873
F	0.500456	-2.505795	0.327850
Si	-2.520738	0.654017	3.397023
C	-2.998927	-0.998324	4.167469
Si	-3.024942	3.296605	0.769313
C	-1.779191	4.217998	1.857630
C	-0.705378	0.985224	3.714848
C	-3.495669	1.949461	4.366119
F	-4.116681	-3.389733	0.010359
F	-2.952320	-4.046554	1.684793
F	-0.804487	-1.969932	1.949936
F	-0.395474	-4.047504	1.514444
F	0.093507	-2.654841	-2.918527
F	-1.319857	-2.298185	-4.500078
F	-3.109793	-0.009384	-3.087879
F	-3.836353	-1.873212	-3.883743
Si	5.139054	-0.021985	1.215229
C	5.877515	0.995121	-0.195241
Si	3.501436	-3.077282	-0.085266
C	2.230235	-4.172639	-0.943177
C	5.452906	1.024409	2.759798
C	6.380403	-1.441335	1.424823

F	3.440450	2.400938	1.093203
F	2.993022	4.495231	0.972251
F	-0.398971	3.099886	-0.252962
F	0.632404	4.991620	-0.022512
F	1.933781	-0.942021	-2.545083
F	3.449118	-0.009420	-3.764635
F	1.371759	2.985666	-4.005059
F	1.201927	0.911788	-4.483816
C	4.807437	-2.841296	-1.436089
C	4.192095	-4.199546	1.267162
C	1.107074	-2.284590	4.035062
C	4.037517	-2.136918	4.124193
C	-2.956908	3.737352	-1.069405
C	-4.645388	4.083591	1.360455
C	-6.081994	-1.209700	0.543127
C	-6.104232	1.190137	2.133117
H	5.390289	-3.765920	-1.511076
H	4.313986	-2.686945	-2.399129
H	5.501753	-2.015699	-1.288500
H	2.800193	-4.957971	-1.452285
H	1.551833	-4.657830	-0.239917
H	1.653088	-3.638966	-1.695433
H	4.510784	-5.136193	0.796611
H	5.044941	-3.788236	1.804981
H	3.413460	-4.446994	1.994816
H	6.908549	1.220616	0.097779
H	5.912641	0.466739	-1.147983
H	5.364982	1.947840	-0.329100
H	7.358399	-0.961315	1.546506
H	6.206140	-2.042074	2.317206

H	6.453011	-2.116737	0.573092
H	6.445974	1.469827	2.631819
H	4.742781	1.833744	2.914620
H	5.488508	0.420337	3.667582
H	1.353109	-2.500048	5.080857
H	0.153716	-1.760161	4.023642
H	0.983674	-3.241696	3.524664
H	1.820598	0.410398	5.048268
H	3.468387	0.744317	4.522249
H	2.101942	1.231185	3.514262
H	3.890385	-2.099785	5.209776
H	4.098806	-3.187812	3.837940
H	4.995962	-1.665331	3.914503
H	-2.869073	-0.916260	5.252162
H	-4.046739	-1.244535	3.976184
H	-2.389139	-1.831011	3.817088
H	-0.457058	2.034396	3.538742
H	-0.453250	0.748107	4.752723
H	-0.067043	0.391156	3.061072
H	-3.117890	1.946591	5.394726
H	-3.370661	2.960475	3.975935
H	-4.562780	1.725048	4.405617
H	-1.536157	5.183779	1.404710
H	-2.231604	4.422103	2.831567
H	-0.844907	3.687030	2.024299
H	-2.502617	4.723515	-1.208619
H	-2.394566	3.021072	-1.668968
H	-3.973287	3.783879	-1.468637
H	-4.539583	5.147945	1.115979
H	-5.551757	3.726351	0.872479

H	-4.784076	4.018141	2.441702
H	-7.152566	-0.974650	0.511029
H	-5.852136	-1.820458	-0.323790
H	-5.906503	-1.798947	1.444165
H	-6.766809	1.091522	-1.227484
H	-5.752136	2.477666	-0.823682
H	-5.094484	1.163054	-1.793021
H	-7.163059	1.323548	1.883955
H	-6.052485	0.461277	2.948186
H	-5.726722	2.138236	2.502857
H	-3.479082	-3.711208	-2.107175
H	-0.608376	-4.210338	-1.212375
H	3.820635	4.048582	-1.074652
H	4.280868	1.974984	-2.051061
H	-2.090708	-4.057607	-3.122400
H	-2.073213	-4.955667	-0.564097
H	3.594845	2.724842	-3.489811
H	2.525215	4.411755	-2.198241

(TMS$_3$Si)(Pip^{TFM})P•

69

E= -3458.147204

C	-2.262034	1.319943	0.048298
N	-1.647956	0.013526	-0.222001
C	-2.360375	-1.255313	-0.017012
C	-3.840832	-1.138404	0.430314
C	-4.130323	0.079258	1.274122
C	-3.758432	1.284528	0.440716
P	-0.132672	-0.044439	-1.220686
Si	1.835634	-0.008467	-0.048305

Si	2.965312	-1.745288	-1.200832	H	2.759386	3.194512	1.399261
C	2.057872	-3.385044	-1.040163	H	-3.997701	2.221127	0.942014
C	-2.435883	-2.114661	-1.332099	H	-4.348252	1.259825	-0.478559
F	-1.283826	-2.689504	-1.688641	H	2.811238	3.319879	-2.886179
C	-1.612429	-2.069271	1.075361	H	2.470859	1.619853	-3.237016
F	-1.648338	-1.422229	2.246859	H	1.209363	2.645669	-2.552712
C	-1.511787	2.026983	1.213638	H	5.213168	2.691728	-1.171616
F	-0.189312	2.050989	1.044483	H	5.164646	1.444279	0.079989
C	-2.206747	2.255387	-1.212098	H	5.012833	0.997541	-1.625522
F	-2.590285	1.588239	-2.298039	H	1.530113	0.421571	4.463417
Si	2.269901	-0.345009	2.250069	H	1.164258	1.659911	3.251530
C	1.161770	0.585209	3.444977	H	0.130294	0.235296	3.397493
Si	2.869927	1.991210	-0.796976	H	4.341345	0.091029	3.537024
C	2.279321	2.431221	-2.529858	H	4.747868	-0.231386	1.847221
C	2.236772	-2.172557	2.704787	H	4.114837	1.351548	2.319986
C	4.035165	0.276240	2.501907	H	2.590608	-2.293759	3.734122
F	-3.319422	-3.115787	-1.197709	H	1.229723	-2.587444	2.642902
F	-2.851579	-1.352818	-2.343599	H	2.886441	-2.767473	2.057850
F	-2.162351	-3.275316	1.269481	H	3.580479	-2.146290	-3.574592
F	-0.326091	-2.274869	0.780420	H	2.136174	-1.129545	-3.477611
F	-1.909487	3.300372	1.355521	H	3.734014	-0.428338	-3.187942
F	-1.750199	1.411799	2.376396	H	2.634014	-4.174730	-1.533956
F	-3.032856	3.302178	-1.079778	H	1.919524	-3.671172	0.005298
F	-0.990061	2.768803	-1.441511	H	1.072048	-3.342994	-1.506376
C	2.516804	3.435066	0.360284	H	5.232718	-2.730714	-1.066191
C	4.739501	1.748410	-0.879318	H	5.285349	-1.051125	-0.517347
C	3.116673	-1.317073	-3.030273	H	4.663651	-2.314183	0.551735
C	4.697651	-1.971287	-0.486019	H	-5.199574	0.112701	1.494313
H	3.121641	4.299355	0.066545	H	-3.600666	0.055198	2.223764
H	1.466401	3.728824	0.321825	H	-4.458667	-1.079050	-0.467830

H	-4.115059	-2.062519	0.935734

$[(TMS_3Si)(Pip^{TFM})P\bullet]_2$

138

E= -6916.267512

C	1.979801	-0.548430	-2.677491
N	1.829505	-1.222677	-1.371414
C	2.374368	-2.614367	-1.279261
C	3.213457	-3.047475	-2.512773
C	2.610649	-2.741705	-3.877776
C	1.720670	-1.503508	-3.859359
P	1.183941	-0.471586	0.168440
Si	2.560480	1.323844	1.096825
Si	3.488251	3.270821	-0.064864
C	2.745936	3.865059	-1.687447
C	3.382533	-2.752348	-0.085089
C	1.319920	-3.750109	-1.091536
C	3.371675	0.169988	-2.822612
C	0.899155	0.560468	-2.841193
P	-1.051397	0.058526	0.499355
Si	-2.541254	1.665928	-0.609834
Si	-4.253213	2.307541	0.980603
C	-3.500511	3.189699	2.465667
N	-1.865969	-1.544405	0.875326
C	-1.796277	-2.008439	2.305117
C	-2.064218	-3.525366	2.552610
C	-3.070638	-4.197310	1.656237
C	-2.713917	-3.868699	0.224253
C	-2.688792	-2.350884	-0.072985
C	-0.372859	-1.878345	2.961570
C	-2.780539	-1.185505	3.202266
C	-4.184621	-1.894590	-0.169914
C	-2.152861	-2.230562	-1.545912
Si	-3.659115	1.583090	-2.751540
C	-5.225590	0.536570	-2.795634
Si	-1.366708	3.741974	-0.853131
C	-0.701437	4.030043	-2.598263
C	-4.291276	3.323594	-3.155610
C	-2.585906	1.130440	-4.231263
Si	1.592922	1.971655	3.244289
C	2.106785	0.780585	4.624474
Si	4.611642	0.408953	1.965565
C	6.040137	0.086460	0.756189
C	2.270816	3.634625	3.828689
C	-0.277573	2.198971	3.347960
C	5.293578	1.767168	3.102418
C	4.329101	-1.135011	3.023328
C	3.478113	4.796074	1.046651
C	5.324213	3.048877	-0.456460
C	-2.521872	5.217766	-0.598060
C	-0.038665	3.998850	0.443637
C	-5.430806	3.518392	0.128030
C	-5.495796	1.044280	1.623430
F	-2.436492	-3.318920	-2.274341
F	-2.679981	-1.198501	-2.217459
F	-0.845432	-2.079320	-1.573129
H	-5.770104	0.791434	-3.711274
H	-5.030477	-0.534447	-2.805831
H	-5.881623	0.753378	-1.948508
F	-4.772101	-2.465843	-1.239377

F	-4.932445	-2.237176	0.877140
F	-4.295911	-0.579378	-0.301515
H	-3.413598	-4.335424	-0.466526
H	-1.723264	-4.265145	0.000095
H	-3.184892	1.300299	-5.132786
H	-1.697265	1.760928	-4.299820
H	-2.265969	0.089701	-4.228304
H	-4.773668	3.243221	-4.136675
H	-5.042238	3.684369	-2.454252
H	-3.514946	4.082619	-3.242858
F	0.905387	1.087677	-4.073740
F	-0.319748	0.078080	-2.653250
F	1.079376	1.569349	-1.985470
F	3.349119	1.163768	-3.720498
F	3.763921	0.688600	-1.660605
F	4.355492	-0.645231	-3.228558
F	0.462381	-3.559005	-0.095861
F	0.618564	-3.959288	-2.214205
F	1.945126	-4.912898	-0.824677
F	4.251768	-3.760448	-0.261557
F	4.114192	-1.640503	-0.012416
F	2.807202	-2.966774	1.091746
H	-6.112752	1.573475	2.359009
H	-6.157254	0.709872	0.821190
H	-5.080318	0.167746	2.108247
H	-6.123573	3.866438	0.902894
H	-4.972637	4.394857	-0.323349
H	-6.030037	3.006253	-0.629445
H	-4.302091	3.709909	3.001329
H	-3.032389	2.490198	3.158222

H	-2.748782	3.931421	2.188552
H	-0.228496	5.018132	-2.621155
H	0.031766	3.305816	-2.940892
H	-1.514045	4.049449	-3.326040
H	0.502838	4.925185	0.231928
H	-0.496878	4.092490	1.429631
H	0.679391	3.180974	0.487836
H	-1.892564	6.110000	-0.697665
H	-3.307830	5.289617	-1.350669
H	-2.981746	5.252188	0.390509
H	5.650571	3.993302	-0.906877
H	5.941367	2.882020	0.426904
H	5.528797	2.253322	-1.171534
H	1.685697	4.091396	-1.620738
H	3.269864	4.795861	-1.932315
H	2.899375	3.173777	-2.513690
H	3.954438	5.598428	0.471861
H	2.477939	5.131229	1.319964
H	4.055593	4.661885	1.962047
H	-0.669698	1.629540	4.194538
H	-0.508067	3.253431	3.520295
H	-0.809923	1.862829	2.461210
H	1.501924	1.041586	5.500209
H	1.935816	-0.271344	4.409411
H	3.153479	0.901282	4.906760
H	1.936517	3.738308	4.867839
H	3.356983	3.719375	3.821432
H	1.852303	4.469558	3.265881
H	6.456095	-0.906210	0.948961
H	5.755599	0.124251	-0.293451

H	6.835956	0.818311	0.912816
H	4.951000	-1.961542	2.670713
H	4.592714	-0.934613	4.065054
H	3.293057	-1.472614	2.999058
H	6.293204	1.451617	3.421977
H	5.404858	2.728044	2.593418
H	4.699709	1.931216	4.000926
F	-2.469367	-1.233603	4.503226
F	-2.818550	0.102123	2.864982
F	-4.027460	-1.664114	3.106496
F	-0.351733	-2.450583	4.179734
F	0.543011	-2.518826	2.252786
F	0.016908	-0.621078	3.146528
H	-2.989262	-5.277493	1.799277
H	-4.090030	-3.918310	1.903212
H	-1.113394	-4.038767	2.408561
H	-2.328503	-3.642757	3.602849
H	4.182527	-2.569467	-2.423753
H	3.401201	-4.114342	-2.420600
H	3.431988	-2.608957	-4.582539
H	2.020628	-3.586914	-4.230499
H	0.677817	-1.806316	-3.762182
H	1.812758	-0.948212	-4.792636

$(PFS_3Si)(Pip^{Me})P$•

69

E= -4946.671687

C	-5.977212	0.825838	-1.325601
C	-4.893032	1.844973	-1.612982
C	-3.500161	1.436009	-1.104239
N	-3.178250	0.017237	-1.473822
C	-4.252821	-1.052570	-1.535431
C	-5.586632	-0.464304	-2.020168
C	-3.416524	1.627345	0.410782
C	-2.501722	2.367001	-1.792632
P	-1.620408	-0.570809	-1.705379
Si	0.011677	0.014715	-0.164556
Si	-0.297681	-1.078829	1.934362
C	-0.715306	-2.973326	1.672802
F	-1.973734	-3.250871	2.058736
C	-3.871177	-2.155953	-2.536558
C	-4.423036	-1.695448	-0.153570
Si	0.922001	2.204532	0.149822
C	-0.062389	3.485975	1.234022
F	0.655215	4.622032	1.312646
Si	1.749230	-1.189961	-1.319641
C	3.047020	-0.078667	-2.256426
F	4.229727	-0.694560	-2.364393
C	2.650916	2.149232	1.065918
F	2.519221	2.531951	2.344063
C	1.082218	3.109605	-1.583802
F	2.277550	3.650186	-1.809263
F	3.140043	0.892727	1.085882
F	3.576959	2.932352	0.510875
C	2.839538	-2.354881	-0.202204
F	3.395930	-3.347851	-0.903967
C	1.141887	-2.359633	-2.760952
F	2.207011	-2.897415	-3.378603
F	3.827281	-1.687589	0.402210
F	2.093516	-2.934231	0.763163

C	1.126529	-0.905663	3.249647
F	0.919981	-1.694220	4.311689
C	-1.822454	-0.428207	2.960345
F	-1.851263	-0.998184	4.173655
F	2.349414	-1.168105	2.787284
F	1.129658	0.370432	3.689696
F	0.385403	-3.372827	-2.315310
F	0.442440	-1.708064	-3.701821
F	2.633498	0.217857	-3.494934
F	3.272559	1.094282	-1.625485
F	-1.783923	0.900361	3.145831
F	-2.996486	-0.709163	2.368595
F	-0.628055	-3.325345	0.375353
F	0.092450	-3.779186	2.367064
F	0.825539	2.250864	-2.594356
F	0.178629	4.101960	-1.685615
F	-1.263561	3.817205	0.732354
F	-0.262241	3.047370	2.481202
H	-2.784240	3.406099	-1.615821
H	-1.497652	2.241030	-1.398611
H	-2.469231	2.186097	-2.868862
H	-3.568550	2.676740	0.667604
H	-4.151026	1.029094	0.946688
H	-2.428830	1.349336	0.780911
H	-5.126958	2.815740	-1.167370
H	-4.838582	1.997944	-2.696889
H	-5.138965	-2.519120	-0.218177
H	-3.474294	-2.103320	0.198260
H	-4.786628	-0.991261	0.593674
H	-4.740909	-2.804158	-2.670366
H	-3.608320	-1.735119	-3.510260
H	-3.045850	-2.779699	-2.191173
H	-6.937803	1.181903	-1.706060
H	-6.104637	0.673819	-0.249205
H	-5.516592	-0.275828	-3.097744
H	-6.348934	-1.235889	-1.881669

$[(PFS_3Si)(Pip^{Me})P\bullet]_2$

138

E= -9893.324162

C	0.928596	1.577103	3.014217
N	1.056562	2.423024	1.777553
C	1.218904	3.932094	2.022348
C	1.880290	4.171503	3.393068
C	1.085004	3.609452	4.573151
C	0.289924	2.366701	4.167394
P	1.058317	1.781060	0.207287
Si	2.770738	-0.039371	0.032679
Si	3.126411	-1.912448	1.661506
C	1.955176	-2.441057	3.157566
C	2.116647	4.598324	0.984011
C	-0.134957	4.664031	2.041695
C	2.315744	1.109391	3.459644
C	0.018114	0.392852	2.725923
P	-0.924813	0.906800	-0.937935
Si	-2.935308	0.004746	-0.000917
Si	-4.463384	-0.310210	-1.855249
C	-3.643597	-1.178535	-3.405971
N	-1.417619	2.215809	-1.936996
C	-0.731272	2.264594	-3.302782

C	0.440407	3.260309	-3.258194	H	-1.651913	2.939114	0.680844
C	0.049240	4.644170	-2.766856	F	-6.488866	2.450853	2.500418
C	-0.636992	4.520962	-1.415158	F	-5.288820	3.436654	1.009566
C	-1.843859	3.570399	-1.412387	F	-6.700854	1.900741	0.431384
C	-0.175024	0.901922	-3.689118	H	-3.659179	4.748853	-1.550801
C	-1.715004	2.636338	-4.424494	H	-2.595322	4.986544	-2.922742
C	-2.977892	4.236276	-2.231960	H	-3.558886	3.525999	-2.800857
C	-2.370052	3.403635	0.006210	H	-0.963318	5.501316	-1.051965
Si	-4.460449	0.864893	1.706808	H	0.093029	4.133404	-0.709560
C	-5.808444	2.249812	1.359896	F	-4.480688	1.944351	4.235967
Si	-2.402796	-2.284140	0.641785	F	-2.762081	0.706270	3.869347
C	-2.386028	-2.804236	2.525461	F	-2.882398	2.683604	2.985011
C	-5.564052	-0.606738	2.364867	F	-6.869727	-0.340142	2.352771
C	-3.602143	1.580440	3.299896	F	-5.380801	-1.702587	1.588624
Si	3.061373	-1.160585	-2.178306	F	-5.233930	-0.946593	3.616779
C	3.369292	0.264413	-3.505614	H	-0.180836	-0.159640	3.640324
Si	4.813790	1.356138	0.018531	H	-0.932297	0.748376	2.326771
C	5.380497	2.035853	1.765946	H	0.445084	-0.301491	2.005787
C	4.689374	-2.268468	-2.182194	H	2.258829	0.549633	4.389224
C	1.859914	-2.327586	-3.220582	H	2.768746	0.457226	2.711011
C	6.533179	0.716082	-0.679995	H	3.003763	1.933002	3.605089
C	4.688208	2.990219	-1.080431	H	-0.506578	4.846810	1.041346
C	3.131390	-3.628354	0.715353	H	-0.901082	4.128836	2.597014
C	4.825618	-1.913635	2.618209	H	0.001255	5.640406	2.514177
C	-3.378129	-3.769602	-0.171107	H	2.127832	5.673049	1.181682
C	-0.635903	-2.697540	-0.064532	H	3.138835	4.239484	1.055539
C	-6.121446	-1.282485	-1.487970	H	1.777216	4.458347	-0.037151
C	-5.259171	1.383716	-2.440194	F	-6.584874	1.322834	-2.576754
H	-2.636637	4.375834	0.421189	F	-5.010940	2.335900	-1.515315
H	-3.283277	2.812352	-0.014205	F	-4.776240	1.831898	-3.607306

F	-6.851456	-1.347867	-2.611301
F	-5.997259	-2.523189	-1.029646
F	-6.839542	-0.594324	-0.577558
F	-3.481651	-0.342092	-4.441616
F	-2.427225	-1.657910	-3.100854
F	-4.383783	-2.203951	-3.840498
F	-1.657593	-3.914671	2.685588
F	-1.862434	-1.848638	3.312254
F	-3.606511	-3.075000	2.995063
F	-0.141755	-3.856842	0.347902
F	-0.700759	-2.728693	-1.397592
F	0.296112	-1.745284	0.250046
F	-2.614773	-4.872787	-0.137621
F	-4.516789	-4.068385	0.452222
F	-3.653619	-3.529977	-1.466628
F	5.203351	-3.157867	2.935529
F	5.817887	-1.372849	1.898015
F	4.732452	-1.230646	3.772700
F	0.730356	-2.793249	2.746436
F	2.519003	-3.526911	3.711470
F	1.792574	-1.568469	4.158382
F	2.441648	-4.592974	1.322049
F	2.543561	-3.451003	-0.495695
F	4.358365	-4.101038	0.492667
F	2.562132	-2.581075	-4.349116
F	1.547813	-3.506195	-2.696192
F	0.716754	-1.744189	-3.590746
F	2.667829	0.102069	-4.634744
F	2.965270	1.443095	-2.968346
F	4.640599	0.426983	-3.861727
F	5.527769	-1.964571	-3.172219
F	5.359882	-2.109707	-1.014009
F	4.420363	-3.572096	-2.303105
F	6.651619	2.443513	1.743827
F	4.641609	3.095171	2.160033
F	5.279647	1.121651	2.740092
F	5.318936	4.004883	-0.458972
F	5.292314	2.818460	-2.262921
F	3.445097	3.415749	-1.343235
F	7.361875	1.769742	-0.788517
F	7.145355	-0.179557	0.094959
F	6.417718	0.190057	-1.905577
H	-1.210277	2.470132	-5.378875
H	-2.595292	1.998006	-4.397654
H	-2.033476	3.673824	-4.411545
H	0.282342	0.979903	-4.675398
H	0.594364	0.564013	-3.001130
H	-0.945575	0.134373	-3.727823
H	0.944927	5.263122	-2.663287
H	-0.590119	5.154097	-3.493557
H	1.206333	2.860536	-2.591878
H	0.886019	3.313435	-4.255925
H	2.894380	3.781971	3.379840
H	1.987955	5.253785	3.497502
H	1.773885	3.373462	5.387828
H	0.395680	4.362321	4.962693
H	-0.728772	2.629950	3.889576
H	0.199944	1.681055	5.013502
